# Deficiency of MIF Accentuates Overloaded Compression-Induced Nucleus Pulposus Cell Oxidative Damage via Depressing Mitophagy

**DOI:** 10.1155/2021/6192498

**Published:** 2021-07-01

**Authors:** Yiyang Wang, Yanzhu Hu, Haoming Wang, Ningyuan Liu, Lei Luo, Chen Zhao, Dandan Zhou, Hang Tong, Pei Li, Qiang Zhou

**Affiliations:** ^1^Department of Orthopedics, The Third Affiliated Hospital of Chongqing Medical University, Chongqing 401120, China; ^2^Tissue Repairing and Biotechnology Research Center, The Third Affiliated Hospital of Chongqing Medical University, Chongqing 401120, China; ^3^Department of Orthopedics, Three Gorges Central Hospital of Chongqing University, Chongqing 404000, China; ^4^Department of Gastroenterology, The People's Hospital of Jiulongpo District, Chongqing 400050, China; ^5^Department of Urology, The First Affiliated Hospital of Chongqing Medical University, Chongqing 400016, China

## Abstract

Established studies proved that mechanical compression loading had multiple effects on the biological behavior of the intervertebral disc (IVD). However, the regulating mechanism involved in this process remains unclear. The current study is aimed at exploring the potential bioregulators and signaling pathways involved in the compression-associated biological changes of nucleus pulposus (NP) cells. Tandem mass tag- (TMT-) based quantitative proteomics was exerted to analyze the differentially expressed proteins (DEPs) and signal pathways among the different groups of NP cells cultured under noncompression, low-compression (LC), and high-compression (HC) loading. Eight potential protective bioregulators for the NP cell survival under different compression loading were predicted by the proteomics, among which macrophage migration inhibitory factor (MIF) and oxidative stress-related pathways were selected for further evaluation, due to its similar function in regulating the fate of the cartilage endplate- (CEP-) derived cells. We found that deficiency of MIF accentuates the accumulation of ROS, mitochondrial dysfunction, and senescence of NP cells under overloaded mechanical compression. The potential molecular mechanism involved in this process is related to the mitophagy regulating role of MIF. Our findings provide a better understanding of the regulatory role of mechanical compression on the cellular fate commitment and matrix metabolism of NP, and the potential strategies for treating disc degenerative diseases via using MIF-regulating agents.

## 1. Introduction

Intervertebral disc (IVD) degeneration, which can result in low back pain (LBP), instability, and deformity of the spine, has been recognized as the leading cause of degenerative spine disease [1]. The human IVD consists of three compartments: nucleus pulposus (NP), annulus fibrosus (AF), and cartilage endplate (CEP), which connects the adjacent bony vertebral bodies. NP tissue is a type of gelatinous structure, containing collagen fibrils and proteoglycan molecules [2]. NP cell is responsible for the synthesis of the functional extracellular matrix (ECM) components (mainly aggrecan and collagen type II) in the core region of the disc, which firstly reveals apoptosis- and/or senescence-like changes during the process of IVD degeneration [2]. In the degenerative intervertebral disc, the senescent NP cells accumulate and result in weakened proliferation, compromised self-repair, increased organellar dysfunction, and enhanced breakdown of functional ECM [3, 4]. Hence, a potential strategy for treating IVD degeneration is to alleviate or retard the apoptosis- and/or senescence-like changes of NP cells by regulating the potential signaling molecules or pathways involved in the degeneration process.

It is commonly held that the overloaded compressive force applied to the IVD is the leading cause of IVD degeneration [4–6]. The prior studies reported that the physiological intensity (0.35-0.75 MPa) of compression loading acted as an anabolic factor for the NP and AF cells, owing to the stimulation of proteoglycan synthesis. In contrast, excessive compression (≥1 MPa) aggravated the catabolic metabolism of proteoglycan of NP and AF cells [7, 8]. Noticeably, our previous studies also elucidated the function of graded compression loading in controlling NP cell survival and proved that overloaded mechanical compression markedly exacerbated degenerative changes of the NP [5, 6, 9, 10]. Although the molecular mechanism involved in the compression loading-associated biological behavior changes of NP cell remains unclear, most studies point that the overloaded compression-induced apoptotic and senescent changes of NP cell are related to the intracellular oxidative damage triggered by mechanosensing factors, such as the integrins and cadherins [5, 6, 11–13]. Therefore, it is meaningful to explore the potential bioregulators and signaling pathways involved in the compression-associated biological changes of NP cell.

Hence, in the present study, we used the human NP cells isolated from a surgical excisional IVD in a patient who suffered lumbar vertebral fracture (LVF) as the investigated subject. The NP cells, seeding in the methacrylamide-modified gelatin (GelMA) hydrogels, commonly used scaffolds for 3D cell culturing, were cultured in our self-developed compression loading bioreactor under the dynamic graded compression loading for 2 weeks, referring to our previous study [5]. After that, we analyzed the apoptosis- and senescence-like changes of the NP cells and determined 5% deformation of the hydrogel as the low-compression (LC) loading and 20% deformation of the hydrogel as the high-compression (HC) loading for the NP cells. We further used tandem mass tag- (TMT-) based quantitative proteomics to analyze the differentially expressed proteins (DEPs) among the different NP cells cultured under noncompression, LC, and HC loading compression. Analysis of the DEPs, Gene Ontology (GO), and Kyoto Encyclopedia of Genes and Genomes (KEGG) enrichment predicted eight potential protective bioregulators for the NP cell survival under overloaded compression, among which macrophage migration inhibitory factor (MIF) and oxidative stress-related pathways were further evaluated in vivo and in vitro, due to its similar function in regulating the fate of the human CEP-derived cells [14]. Moreover, the recombinant lentiviral vector construct containing MIF short hairpin RNA (shRNA) was used to establish the MIF knockout (MIF-KO) NP cells to further determine the protective function of MIF in NP cells that suffered overloaded compression.

## 2. Material and Methods

### 2.1. Source of the Human NP Sample

For the better homogeneity of the samples prepared for the proteomics analysis, the experimental NP samples in this study were obtained from a 32-year-old, female patient who suffered lumbar vertebral fracture (LVF) and admitted to the orthopedics department of our hospital. The patient received the discectomy surgery and donated the excisional IVD tissue for experimental research. The patient declared no documented medical history of LBP before suffering the LVF. The preoperative MRI scans of the patient revealed that the excisional IVD was graded in Pfirrmann II (normal or mild degeneration), according to the Pfirrmann classification system (Supplemental Figure 1) [15]. Informed consent was obtained from the patient, and the researching procedure complied with approval from the ethics committee of the Third Affiliated Hospital of Chongqing Medical University.

### 2.2. Isolation of the Primary Human NP Cells

The primary human NP cells were isolated as follows [5]. The NP tissue was carefully separated from the excisional IVD and washed by phosphate-buffered saline (PBS) five times in the tissue culture dishes (Jet Biofil, China). Then, the tissue was minced into flocculent pieces and digested in 0.2% type II collagenase (Sigma, USA) for 4 hours, following 0.25% trypsin (Gibco, USA) digestion for 30 minutes. The DMEM/F-12 medium (Gibco, USA) containing 10% fetal bovine serum (FBS, Gibco, USA) and 1% penicillin/streptomycin (Gibco, USA) was used to neutralize the digesting solution. The collected suspension was centrifuged at 1200 rpm for 8-10 minutes. Finally, the collected NP cells were resuspended by the completed culture medium and seeded into flasks (Jet Biofil, China), incubated at 37°C with 5% CO_2_. Culturing media were replaced every four days.

### 2.3. GelMA Hydrogel Synthesis and Preparation

In brief, 10% (*w*/*v*) of gelatin from porcine skin (Sigma, USA) was dissolved in 1x PBS. The methacrylic anhydride (Aladdin, China) was added to the gelatin solution in a dropwise manner when the solution was heated to 60°C. The reaction was allowed to proceed for 4 hours at 60°C before being stopped using a 5x PBS dilution. The resulting solution was dialyzed against distilled water using a 12-14 kDa dialysis tubing (Thermo Fisher, USA) at 60°C for 5-7 days with two water changes per day. The solution was then stored at −20°C for 24 hours and lyophilized for 72 hours before use.

### 2.4. Establishment of the 3D NP Cell Culturing Network

The NP cells were encapsulated in GelMA hydrogel for 3D culturing and compression loading. The cell encapsulation procedure was performed according to our previous study [5]. In brief, the cells at passage II were trypsinized with 0.25% trypsin (Gibco, USA) for 2 minutes, and the collected cell suspension was then centrifuged at 1200 rpm for 5 minutes. The centrifuged cell pellets were then mixed with the GelMA hydrogel precursor (1 × 10^7^ cells/mL). After that, the mixture suspension was pipette into a self-made mold (diameter = 1 cm, thickness = 0.5 cm) and exposed to the UV light (360 ± 5 nm, 850 mW) for about 1 minute at a distance of 8-10 cm to construct the 3D NP cell culturing network. The cell-encapsulated GelMA hydrogels were incubated in the dishes (Jet Biofil, China) for three days before the compression performing regime in the bioreactor.

### 2.5. Mechanical Compression Exerting Protocol

During the compression performing regime, the cell-encapsulated hydrogel was moved to the culturing chamber of our bioreactor and cultured for two weeks. Simultaneously, we set the pressure apparatus to provide intermittent compression load. Particularly, the cell-encapsulated GelMA hydrogel was exerted bionic compression (1.0 Hz, 4 hours/day) in the chamber at 0%, 5%, 10%, and 20% compressive deformation. The compression loading-treated cells used for the following tests were washed out from the hydrogel scaffold via type II collagenase (0.2%, Sigma, USA) digestion for 5 minutes at 37°C.

### 2.6. Senescence-Associated *β*-Galactosidase (SA-*β*-Gal) Staining

SA-*β*-Gal staining was usually used for detecting the cell senescence. Briefly, treated samples were fixed with paraformaldehyde (concentration = 0.2%) for 20 minutes at room temperature. The paraformaldehyde was removed from the cells, and then, the cells were stained with X-gal solution overnight at 37°C. The percentage of the senescent cells (green stained) was observed by optical microscopy (Olympus, Japan).

### 2.7. Live/Dead Assay

The cell viability was detected by the LIVE/DEAD Assay Kit (Invitrogen, USA). In brief, the samples were washed with PBS thrice and incubated with 1 mL of PBS containing 4 mM EthD-1 and 2 mM calcein AM for 30 minutes at 37°C. Then, the washed samples were observed and imaged via fluorescence microscopy (Leica, Germany).

### 2.8. Determination of Cell Apoptosis

The apoptosis of the NP cells was detected using flow cytometry-based Annexin V/PI double-staining. In brief, the cells were washed thrice with PBS and resuspended in 100 *μ*L binding buffer with 5 *μ*L Annexin V-FITC and 5 *μ*L PI for 30 minutes protected from light. The early apoptotic cells contained Annexin V+/PI−, the late apoptotic cells contained Annexin V+/PI+, and the normal cells contained Annexin V−/PI−. The early- and late-stage apoptotic cells were counted, and the results were expressed as a percentage of the total apoptotic cell count.

### 2.9. Protein Extraction and Western Blotting

After being ground in liquid nitrogen, the samples were lysed with RIPA lysis buffer containing 1% PMSF (Beyotime, China) for 30 minutes at 4°C. The lysates were centrifuged (12,000 rpm, 8 minutes) at 4°C. After determining protein concentration, the qualified protein samples spread in SDS-polyacrylamide gel electrophoresis and transferred to a PVDF membrane band by electroblotting. The PVDF membrane bands were then incubated with the primary antibodies (anti-nitrotyrosine (NT), anti-MIF, anti-LC3, anti-P62, anti-PINK1 (1 : 1000; Abcam, UK), anti-P53, anti-P21, anti-P16, anti-aggrecan, anti-collagen II, anti-*β*-actin (1 : 500; Proteintech, China), and anti-Parkin (1 : 1000; Cell Signaling, USA)) overnight at 4°C. After the bands were washed with Tris-Buffered Saline with Tween (TBST) thrice, they were then incubated with the fluorescent secondary antibody for 80 minutes. Being washed with TBST thrice, the intensity of the proteins was determined and analyzed by Image Lab software (Bio-Rad, USA).

### 2.10. TMT-Based Quantitative Proteomics

The TMT-based quantitative proteomics for this work was commissioned by Majorbio Bio-pharm Technology (Shanghai, China) for testing. In brief, we exerted mechanical compression to the NP cell-encapsulated hydrogels for two weeks using our self-developed bioreactor according to the compression performing regime. The treated samples were then divided into three groups based on the compressive deformation of the NP cell-encapsulated hydrogels: the control group—cultured in chambers but unloaded throughout, the LC loading group—loaded with 5% deformation, and the HC loading group—loaded with 20% deformation. Four copies of the samples (test pair *n* = 3, backup pair *n* = 1) were collected. After quick freezing in liquid nitrogen, the samples were stored in drikold and sent to the testing laboratory of Majorbio Bio-pharm Technology. After finishing the quality assessment, proteolysis, peptide labeling, peptide separation, Nano Liquid Chromatography-Mass Spectrometry/Mass Spectrometry (LC-MS/MS) analysis, and other steps of TMT proteomics, the analyzed data were uploaded on the online Majorbio Cloud Platform (https://cloud.majorbio.com). The thresholds of fold change (>1.2 or <0.83) and *P* value < 0.05 were used to identify DEPs. Annotation of all identified proteins was performed using GO (http://www.blast2go.com/b2ghome, http://geneontology.org/) and KEGG pathway (http://www.genome.jp/kegg/). DEPs were further used for GO and KEGG enrichment analysis.

### 2.11. Establishment of the Rat Tail IVD Compressed Model

All animal procedures were performed under the approval of the Animal Care and Use Committee at Chongqing Medical University. Twenty 12-week-old female Sprague-Dawley (SD) rats from 500 ± 5 g were used in the study. Radiographs were taken to confirm the rat tail IVD levels and heights under intraperitoneal anesthesia. Rat tail was then fixed with a self-developed compression loading apparatus between the 8th and 10th caudal vertebrae. An axial force from the distal side was exerted to produce a compression loading of 1.3 MPa, according to the previous study [16]. The rats were then divided into three groups based on the compression loading duration: the sham group—unloaded throughout, the 2 weeks group—loaded for 2 weeks, and the 4 weeks group—loaded for 4 weeks.

### 2.12. Histological Hematoxylin and Eosin (HE) Staining

The treated samples were harvested and fixed with 4% paraformaldehyde, embedded in paraffin, and then cut into 5 *μ*m per section. Then, the sections were stained with hematoxylin and eosin. The stained sections were observed and scanned under an optical microscope (Olympus, Japan).

### 2.13. Alcian Blue Staining

Alcian blue staining was used to detect the ECM glycosaminoglycan deposition. Briefly, each tissue section was incubated in 0.2% Alcian blue solution before rinsing with deionized water. Then, the stained sections were mounted and observed under an optical microscope (Olympus, Japan).

### 2.14. MIF Immunohistochemistry (IHC)

After rehydration, tissue sections were blocked by goat serum, treated with hyaluronidase (0.8%) for 20 minutes at 37°C, and then incubated with MIF antibody (1 : 100, Abcam, UK) for 60 minutes. After washing in PBS, biotinylated secondary antibody (1 : 100, Dako, Denmark) was applied for 30 minutes, washed in PSB, and treated with avidin-biotin complex reagents. Colour was developed using 3,3-diaminobenzidine reagents (Dako, Denmark), and the sections were counterstained with Harris's hematoxylin. The average optical density (AOD) of five randomly selected visual fields (per immunohistochemical slice) under high magnification (400x) was measured using the ImageJ analysis system.

### 2.15. NP Cell Transfection

For knockout of the endogenous MIF in NP cells, shRNA targeting MIF (MIF-shRNA (Santa Cruz Biotechnology, Dallas, TX, U.S.A.)) was transfected into cells by using a recombinant lentiviral vector (GeneChem, Shanghai, China). For transfection, cells were seeded into a 6-well plate (Jet Biofil, China), incubated for 24 h, and then transfected according to the manufacturer's instructions. In brief, the cells were transfected with lentivirus (multiplicity of infection = 50) for three days. After determining the efficacy of transfection by a fluorescent microscope, the transfected cells were subcultured and seeded in the hydrogels for the subsequent experiments.

### 2.16. Detection of Intracellular Reactive Oxygen Species (ROS) Content

Intracellular ROS content was detected by fluorescent DCFH-DA molecular probe staining (Beyotime, China). Briefly, 10 mg DCFH-DA molecular probes were added into a 1 mL basic medium without FBS to prepare the working solution. After that, the cells were incubated in a 6-well plate (Jet Biofil, China) with molecular probe working solution at 37°C for 20 minutes. Then, the cells were collected and the fluorescent intensity was determined by a flow cytometer under 488 nm excitation wavelength and 525 nm emission wavelength.

### 2.17. Determination of Mitochondrial Membrane Potential (*ΔΨ*m)

JC-1 fluorescent molecular probe staining (Beyotime, China) was performed to determine the cell *ΔΨ*m, according to the manufacturer's protocol. In brief, after being washed with PBS thrice, 1 mL basic medium was added to each 6-well plate (Jet Biofil, China). Alive cells were immersed in 1 mL of prepared JC-1 molecular probe working solution and incubated for 30 minutes at 37°C. After being washed with precooled JC-1 1x washing buffer thrice, *ΔΨ*m was detected by laser scanning confocal microscopy (LSCM. Zeiss 780, Germany). The JC-1 molecular probes' fluorescence intensity ratio was then quantified by the ImageJ system.

### 2.18. Colocalization of Mitochondria and Autophagosomes

The intracellular mitophagy initiation was evaluated by the fluorescent colocalization of mitochondria and autophagosomes. NP cells were transiently transfected with GFP-LC3 (Beyotime, China). MitoTracker Red (Beyotime, China) was used to label the mitochondria in cells, which carries a thiol-reactive chloromethyl group that covalently binds to the reduced thiols that present mitochondrial matrix protein. The numbers of total colocalizing GFP-LC3 puncta per cell (mitophagosomes) were counted using ImageJ.

### 2.19. Transmission Electron Microscopy (TEM) Observation

TEM is the most reliable approach for monitoring autophagy [17]. The NP cells were collected and immersed in 0.1 M sodium cacodylate buffer at room temperature for 12 hours. The ultrathin 50 nm sections were cut by using an ultramicrotome, stained with 2% (*w*/*v*) uranyl acetate and lead citrate, and then visualized and captured with a TEM machine (Hitachi, Japan).

### 2.20. Statistical Analysis

All data were analyzed using GraphPad Prism (version 6.0, GraphPad Software, USA) and presented as mean ± standard deviation with *n* = 3. The thresholds of fold change (>1.2 or <0.83) and *P* value < 0.05 were used to identify DEPs. A two-tailed Student's *t*-test was used to assess the statistical significance of the measurement data (*P* value < 0.05).

## 3. Results

### 3.1. Effects of Graded Mechanical Compression on Cell Senescence, Viability, and ECM Synthesis of the Human NP Cells Cultured in a Bioreactor

**Figure 1:**
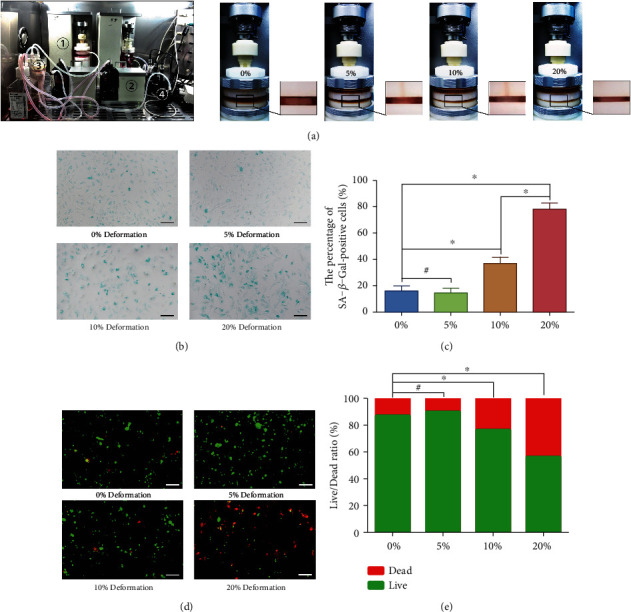
Effects of graded mechanical compression on cell senescence and viability of the human NP cells cultured in a bioreactor: (a) NP cell-encapsulated hydrogels cultured in chambers were dynamically compressed with 0%, 5%, 10%, and 20% deformation for 2 weeks, and the units of our self-developed bioreactor include (1) tissue culture chambers and compression loading application device; (2) substance exchanger peristaltic pumps; (3) medium container; and (4) pH, PO_2_, and PCO_2_ sensor. (b) The cell senescence degree of the NP cells that exerted graded compression loading was analyzed by SA-*β*-Gal staining (100x). (c) Statistic analysis of the SA-*β*-Gal positive rate of the NP cells that exerted graded compression loading. (d) The NP cell viability was analyzed by fluorescent Live/Dead staining (100x). (e) Statistic analysis of the cell Live/Dead rate of the NP cells that exerted graded compression loading. ^∗^*P* < 0.05. ^#^*P* > 0.05. Scale bar = 100 *μ*m.

**Figure 2:**
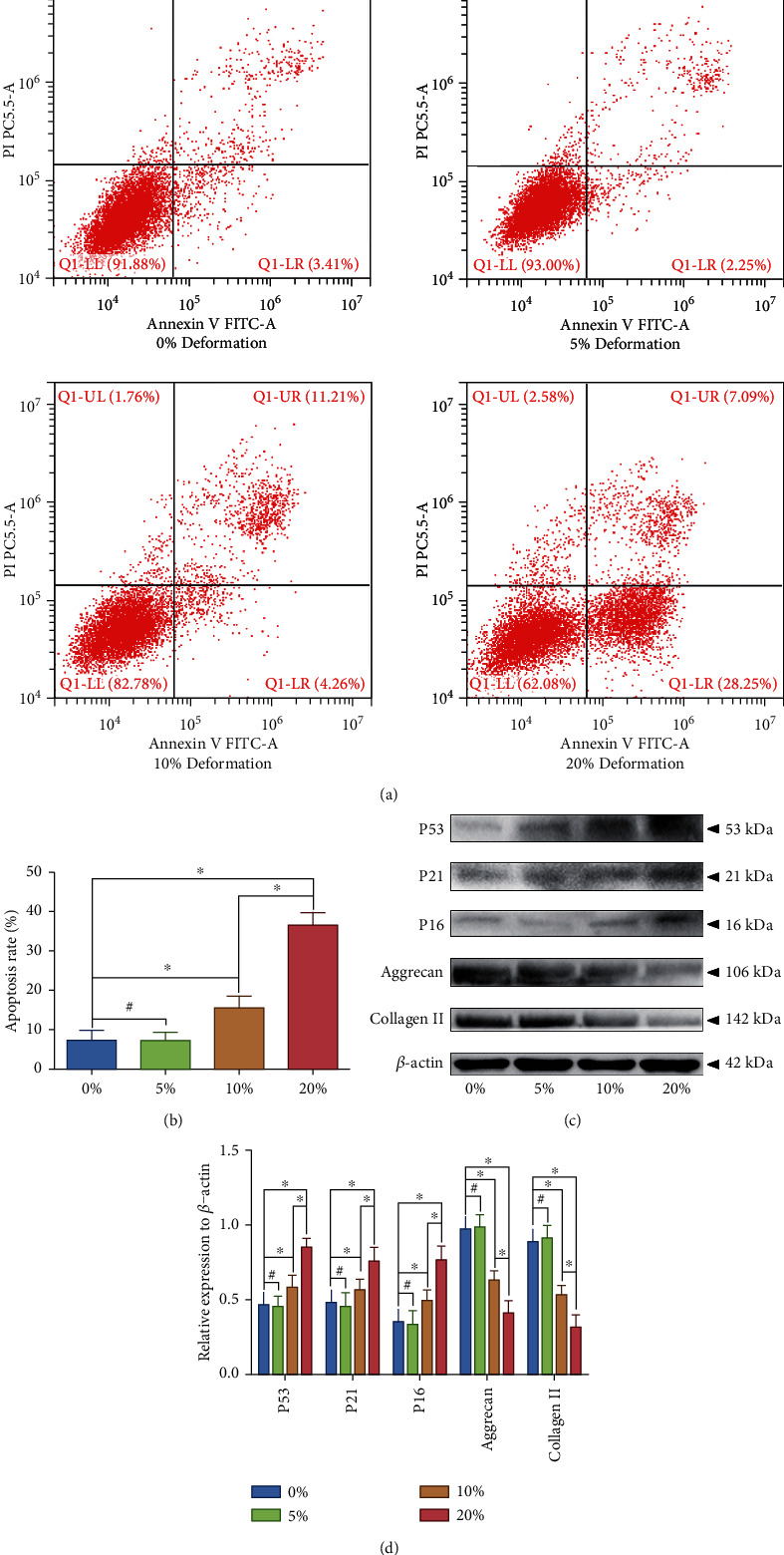
Effects of graded mechanical compression on cell apoptosis, senescence, and ECM homeostasis of the human NP cells cultured in a bioreactor: (a) the early- and late-apoptosis rate of the NP cells that exerted graded compression loading was analyzed by flow cytometry. (b) Statistic analysis of the apoptosis rate of the NP cells that exerted graded compression loading. (c) Western blotting analysis of the expression level senescence-associated markers (P53, P21, and P16) and main ECM functional components (aggrecan and collagen type II) of the NP cells that exerted graded compression loading. (d) Statistic analysis of the western blots of the NP cells that exerted graded compression loading. ^∗^*P* < 0.05. ^#^*P* > 0.05.

Our previous studies evaluated that overloaded compressive force applied to IVD could lead to disc degeneration [5, 6, 9, 10], whereas proper physiological pressure is beneficial for maintaining the cell viability of NP [18]. Our previous studies verified that mechanical compression-induced hydrogel deformation could effectively imitate the in vivo loading situation of the NP cell or mesenchymal stem cell (MSC) [5, 19]. Thus, we cultured the NP cell-encapsulated GelMA hydrogels and imitated the in vivo compression situation using our self-developed compression loading bioreactor (Figure 1(a)). The SA-*β*-Gal staining was used to determine the degree of NP cell senescence in each group. The result revealed that the proportion of positive cell was significantly increased in the group that exerted HC loading (≥10% deformation), but not in the LC loading group (≤5% deformation) (Figures 1(b) and 1(c)). The result of the fluorescent Live/Dead staining also indicated that the death ratio of the NP cells was markedly enhanced by over 10% gel deformation (Figures 1(d) and 1(e)). However, LC loading did not increase the death ratio of the NP cells (Figures 1(d) and 1(e)). Flow cytometry was utilized to detect the cell apoptosis rate, and the result also indicated that apoptosis of the NP cell was significantly enhanced by HC loading, but not in the LC loading group (Figures 2(a) and 2(b)). Additionally, western blotting results revealed that the senescence-associated markers (P53, P21, and P16) were strongly overexpressed in NP cell that suffered HC loading, which was consistent with the results of SA-*β*-Gal staining (Figures 2(c) and 2(d)), whereas the synthesis of functional ECM components (aggrecan and collagen type II) was significantly inhibited by HC loading, but not in the LC loading group (Figures 2(c) and 2(d)).

### 3.2. DEPs and Pathways of the Human NP Cells That Exerted Graded Mechanical Compression Loading Were Analyzed by TMT-Based Quantitative Proteomics

**Figure 3:**
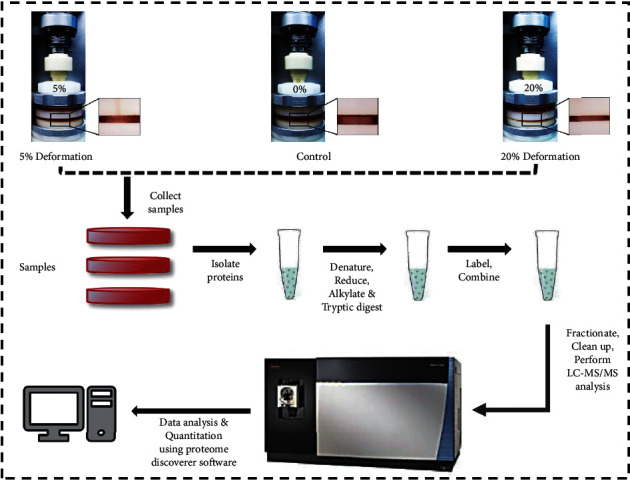
Schematic workflow of the TMT quantitative proteomics procedure: NP cell-encapsulated hydrogels cultured under dynamical compression with 0% (control), 5% (low-compression loading), and 20% deformation (high-compression loading) for 2 weeks, and protein extracts were labeled with 10-plex TMT reagent. Peptides were analyzed via LC-MS/MS, and the raw data for protein identification and relative quantification were analyzed by Proteome Discoverer.

**Figure 4:**
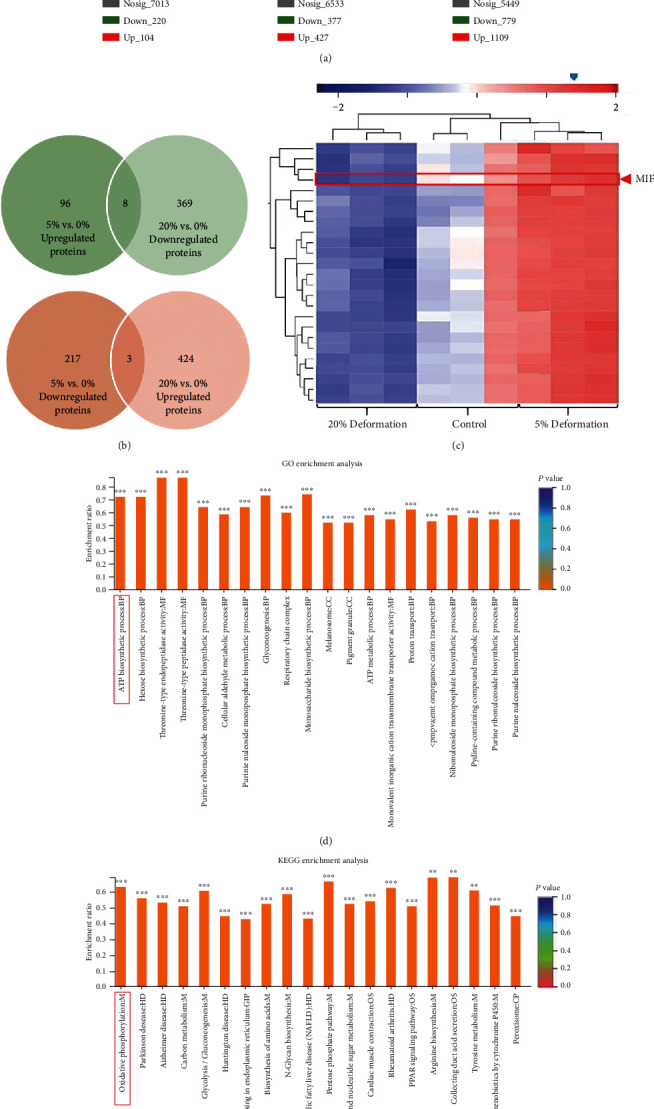
TMT-based quantitative proteomics analyzed the differentially expressed proteins and pathways of the human NP cells that exerted graded mechanical compression loading: (a) the volcano plots revealed the DEPs of the NP cell-encapsulated hydrogels cultured under dynamical compression with 0% (control), 5% (low-compression loading), and 20% deformation (high-compression loading). (b) The Venn diagrams revealed the potential protective proteins (upregulated under low-compression loading but downregulated under high-compression loading) and adverse proteins (downregulated under low-compression loading but upregulated under high-compression loading) of the NP cells that suffered mechanical compression. (c) The heat map revealed part of the DEPs among the 0% (control), 5% (low-compression loading), and 20% deformation (high-compression loading) groups. (d) GO analysis of the DEPs between low-compression loading and high-compression loading groups revealed that the top enriched biological process was ATP biosynthetic process. (e) KEGG enrichment analysis of the DEPs between low-compression loading and high-compression loading groups revealed that the top enriched pathway was related to oxidative phosphorylation. ^∗∗^*P* < 0.01 and ^∗∗∗^*P* < 0.001.

For further analysis of the potential functional factors involved in the progress of compression-associated NP cell survival and senescence, the DEPs of the samples, respectively, that suffered nonloading (control), LC loading (5% deformation), and HC loading (20% deformation) were evaluated by TMT-based proteomics analysis (Figure 3). Among the 324 DEPs, 104 proteins were upregulated and 220 proteins were downregulated in the LC loading group compared with the control group (Figure 4(a), Supplemental Table 1). There were 427 overexpressed proteins and 377 underexpressed proteins in the HC loading group compared with the control group (Figure 4(a), Supplemental Table 2). Moreover, there were 1109 overexpressed proteins and 779 underexpressed proteins in the HC loading compression group compared with the LC loading group (Figure 4(a), Supplemental Table 3). Depending on the aforementioned experimental results, we speculated that the intersection of the overexpressed proteins in the LC loading group and the underexpressed proteins in the HC loading group might play as protective factors for NP cell survival. In contrast, the intersection of the underexpressed proteins in the LC loading group and the overexpressed proteins in the HC loading group might play as adverse factors for NP cell survival. Thus, we further analyzed the protein clusters and predicted 8 potential protective factors and 3 potential adverse factors for NP cell survival (Figures 4(b) and 4(c)). Among the 8 potential protective factors, MIF was selected for further study due to its reported role in regulating the fate of the human CEP cells [14]. In addition, the GO and KEGG enrichment analysis indicated that compression loading affected the fate commitment of NP cell via regulating oxidative stress-associated pathways (ATP biosynthetic process and oxidative phosphorylation) (Figures 4(d) and 4(e)).

### 3.3. Effects of Overloaded Compression on Biological Behavior and MIF Expression of the Rat Tail NP Tissue

**Figure 5:**
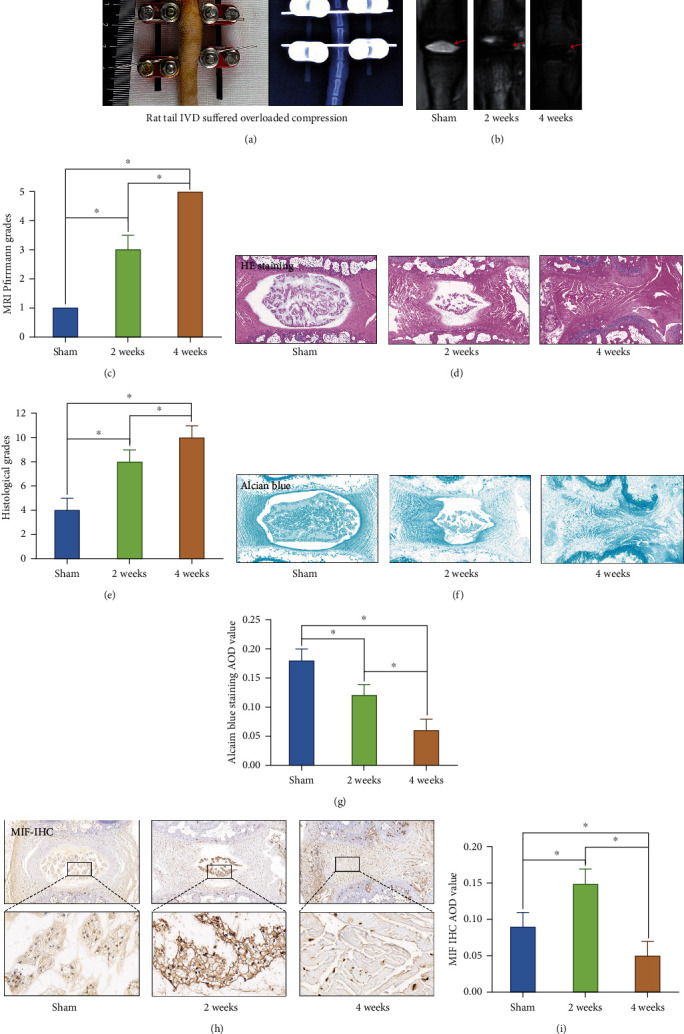
Effects of overloaded compression on biological behavior and MIF expression of the rat tail NP: (a) the close-up and X-ray views of the experimental rat tail IVD under the condition of overloaded compression. (b) The MRI image of the experimental rat tail IVD under the different duration of overloaded compression. (c) Statistic analysis of the MRI Pfirrmann grade changes of the experimental rat tail IVD under the different duration of overloaded compression. (d) The HE staining showed the histomorphological changes of the experimental rat tail IVD under the different duration of overloaded compression. (e) Statistic analysis of the Masuda degenerative score changes of the experimental rat tail IVD under the different duration of overloaded compression. (f) The Alcian blue staining showed the glycosaminoglycan content of the experimental rat tail IVD under the different duration of overloaded compression. (g) Statistic analysis of Alcian blue staining AOD value of the experimental rat tail IVD under the different duration of overloaded compression. (h) MIF IHC staining of the experimental rat tail IVD under the different duration of overloaded compression. (i) Statistic analysis of MIF IHC staining AOD value of the experimental rat tail IVD under the different duration of overloaded compression. ^∗^*P* < 0.05. ^#^*P* > 0.05.

To further verify the relationship between the expression of MIF and compression loading-associated NP degeneration, we conducted a rat tail IVD overloaded compression model (Figure 5(a)) to detect the classification of IVD degeneration and MIF expression referring to the previous study [16]. The MRI T2-weighted image of the compressed rat tail IVD indicated that the NP tissue that revealed the sign of degeneration (darkened tissue signal) occurred under 2-week overloaded compression and deteriorated under 4-week overloaded compression (Figures 5(b) and 5(c)). Additionally, the morphology analysis by HE staining also showed moderate and severe degenerative signs (loss of NP and increase in waviness of the fibrocartilage lamellas of AF) of the compressed rat tail IVD in 2 weeks and 4 weeks according to the Masuda classification [20] (Figures 5(d) and 5(e)). The result of Alcian blue staining also revealed that the content of glycosaminoglycans was gradually declined by prolonging the duration of IVD compression (Figures 5(f) and 5(g)). Noticeably, the MIF expression was upregulated in the moderate degenerative NP that suffered 2-week compression but downregulated in the severe degenerative NP that suffered 4-week compression (Figures 5(h) and 5(i)).

### 3.4. Deficiency of MIF Exacerbated Oxidative Stress-Induced Senescence of the Human NP Cell That Suffered Overloaded Compression

**Figure 6:**
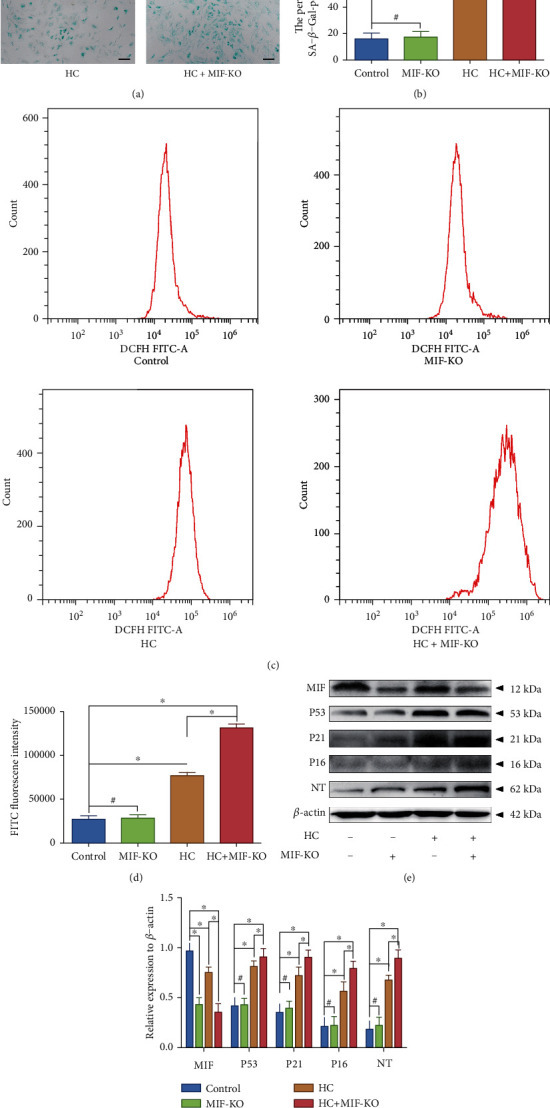
MIF-KO exacerbated oxidative stress-induced senescence of the NP cell that suffered overloaded compression: (a) after MIF-KO and high-compression loading treatment, the cell senescence degree of the NP cells was analyzed by SA-*β*-Gal staining (100x). (b) Statistic analysis of the SA-*β*-Gal-positive rate of the NP cells. (c) After MIF-KO and high-compression loading treatment, the ROS content of the NP cells was measured by flow cytometry following DCFH-DA staining. (d) Statistic analysis of the ROS content of the NP cells. (e) Western blotting analysis of the expression levels of MIF, oxidative stress biomarker NT, and senescence-associated markers (P53, P21, and P16) of the NP cells. (f) Statistic analysis of the western blots of the biomarkers. ^∗^*P* < 0.05. ^#^*P* > 0.05. Scale bar = 100 *μ*m.

Based on the proteomics data, we further figured out the molecular mechanism involved in the MIF-related NP degenerative changes via analyzing the oxidative stress-associated biomarkers. The cells were transfected with lentiviral MIF-shRNA to knock out the endogenous MIF before the analysis of oxidative stress-associated biomarkers. The SA-*β*-Gal staining result indicated that MIF-KO did not affect the senescence rate of the NP cells, but HC loading significantly aggravated the senescence of NP cells (Figures 6(a) and 6(b)). However, MIF-KO further exacerbated the HC loading-induced senescence of NP cells (Figures 6(a) and 6(b)). Flow cytometry was then used to analyze the content of intracellular ROS in NP cells marked by DCFH-DA molecular probes, and the result revealed that knockout of MIF alone did not induce the accumulation of ROS. However, HC loading did increase the content of ROS in NP cells, and MIF-KO markedly exacerbated the ROS content of the HC loading-treated NP cells (Figures 6(c) and 6(d)). In addition, the western blot analysis revealed that the expression of MIF was markedly eliminated by MIF-KO, but the expression of senescence-associated markers (P53, P21, and P16) and oxidative stress-related marker (NT) was not affected by MIF-KO (Figures 6(e) and 6(f)). HC loading depressed the expression of MIF compared with the control group and markedly enhanced the P53, P21, P16, and NT expression. Similarly, knockout of MIF in HC loading-treated NP cells further aggravated the expression of senescence-related and oxidative stress-related markers (Figures 6(e) and 6(f)).

### 3.5. Deficiency of MIF Accentuates Oxidative Stress-Induced Senescence of the NP Cell under Overloaded Compression via Depressing Mitophagy

**Figure 7:**
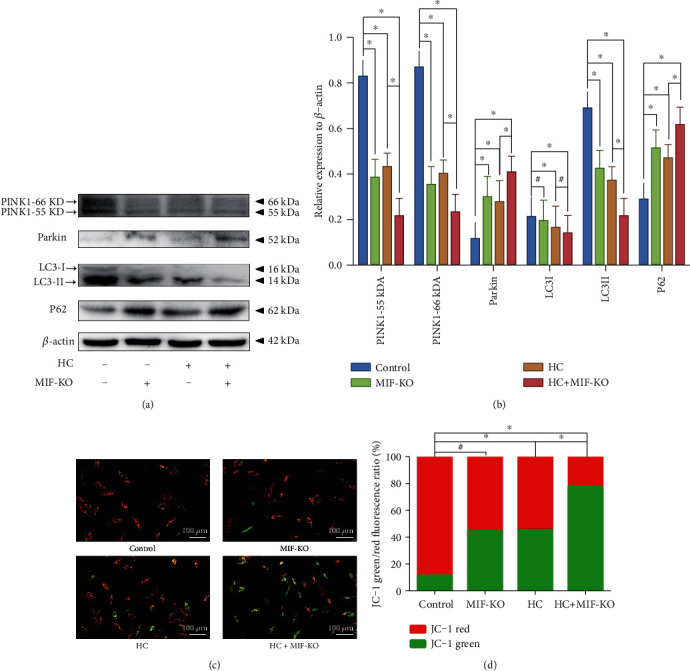
MIF-KO accentuates oxidative stress-induced senescence and mitochondrial dysfunction of the NP cell: (a) western blotting analysis of the expression levels of the mitophagy-related markers (PINK1, Parkin, LC3II/I, and P62) of the NP cells. (b) Statistic analysis of the western blots of the mitophagy-related biomarkers. (c) The mitochondrial membrane potential was observed and measured by fluorescence microscopy after JC-1 fluorescent molecular probe staining. (d) Statistic analysis of the JC-1 green/red fluorescence intensity ratio. ^∗^*P* < 0.05. ^#^*P* > 0.05.

**Figure 8:**
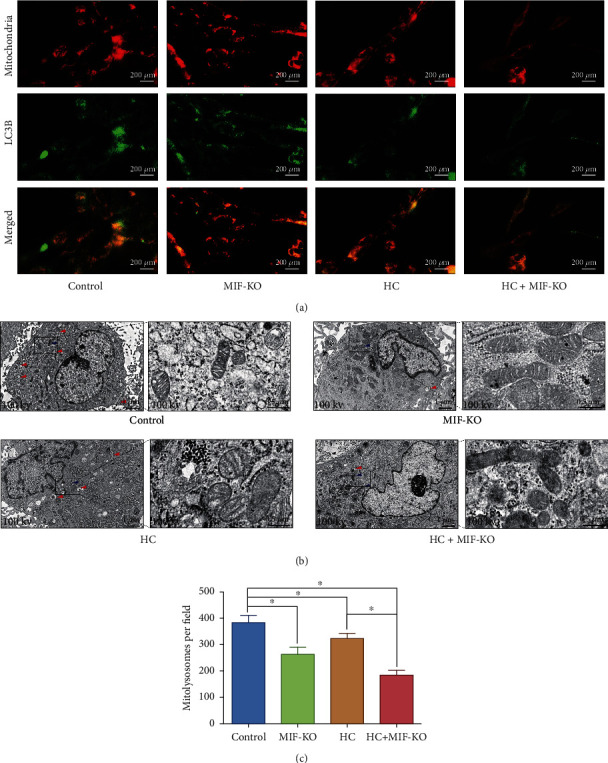
MIF-KO accentuates oxidative stress-induced senescence of the NP cell via depressing mitophagy: (a) mitophagy in NP cells was determined by observation of GFP-LC3 and MitoTracker Red colabeled autophagosomes and mitochondria. (b) The electron micrographs of mitochondria and autophagosomes were observed via TEM, and double-membrane profiles resembling pieces of digested mitochondria were found in some autophagosomes in the high-compression loading group. Blue arrows represent the autophagic vacuole ultrastructural morphology. Red arrows represent the mitochondrial electron micrographs. (c) Statistic analysis of the mitolysosomes per field under optical microscopy (400x). ^∗^*P* < 0.05. ^#^*P* > 0.05.

As the major organelle for ATP biosynthetic process and oxidative phosphorylation, the mitochondrion is also the main ROS generating and attacking component of cells [21]. Our previous studies confirmed that mitophagy could alleviate the oxidative stress-induced senescence of NP cell via recycling the injured mitochondria [5, 22]. In terms of our prior studies and the present GO and KEGG enrichment analysis, we further observed the mitophagy-associated markers in the current study. The western blotting result indicated that both MIF-KO and treatment of HC loading could downregulate the expression of PINK1 and LC3II/I and upregulate the expression of Parkin and P62, which were symbols of the block of mitophagy (Figures 7(a) and 7(b)). Noticeably, knockout of MIF in HC loading-treated NP cells further exacerbated the restraint of mitophagy-related markers (Figures 7(a) and 7(b)). The JC-1 green/red fluorescence ratio is usually used for evaluating the state of the mitochondrion [5]. The JC-1 staining result indicated that knockout of MIF alone did not cause mitochondrial damage, but HC loading aggravated mitochondrial damage in NP cells (Figures 7(c) and 7(d)). Moreover, the MIF-KO NP cells treated with HC loading had the highest JC-1 green/red fluorescence ratio, indicating that deficiency of MIF exacerbated accumulation of the damaged mitochondria induced by the HC loading (Figures 7(c) and 7(d)). To further investigate the mitophagy at various stages, NP cells were transfected with a plasmid encoding GFP-LC3 and incubated with MitoTracker Red to label autophagosomes and mitochondria, respectively. We found that mitochondria in the control group were filamentous and interconnected as a network. Exerting overloaded compression alone enhanced the formation of GFP-LC3 puncta, and a few were costained with mitochondria, which exhibited as short rods or spheres with the network breakdown (Figures 8(a) and 8(c)). Additionally, MIF-KO decreased the amount of colocalizing GFP-LC3 puncta significantly (Figures 8(a) and 8(c)). Furthermore, we observed the amount and morphology of the mitochondria and autophagosomes in the NP cells via TEM. As is shown in Figure 7(e), mitochondria of the NP cells in control and MIF-KO groups maintained the normal morphology and structure, whereas in the HC loading-treated NP cells, the mitochondria appeared shrunken with enhanced membrane density (Figure 8(b)). In addition, the HC loading-treated cell exhibited more autophagosomes, surrounding double-membrane mitochondrion-like segments, in its cytoplasm (Figure 8(b)). However, the MIF-KO NP cells treated with HC loading did not appear typical autophagosome in its cytoplasm (Figure 8(b)). Thus, the results above indicated that deficiency of MIF did retard the mitophagy of NP cells.

## 4. Discussion

Mass studies evaluated that the compressive force applied to the IVD played as a major regulator for the biological behavior of IVD cells, but the particular molecular mechanisms involved in this regulating process are still controversial. Our prior studies elucidated the biological responses of IVD cells to graded mechanical compression loading via establishing the compression-loaded perfusion culture models of IVD cell-encapsulated hydrogels and tissues [5, 6, 9, 10]. In the present study, we analyzed the apoptosis- and senescence-like changes of the human NP cells under graded dynamic compression loading and evaluated that LC loading did not lead to NP cell degenerative changes. In contrast, HC loading did cause cell apoptosis, senescence, and breakdown of ECM. For further exploration of potential regulators and molecular mechanisms involved in this process, we exerted TMT-based proteomics, a popular labeling proteomics method in identifying biomarkers and molecular mechanisms for several diseases.

Depending on the results of our current and prior studies, LC loading compression was beneficial for the NP cell survival and ECM homeostasis, whereas HC loading compression aggravated NP cell senescence, apoptosis, and breakdown of ECM [5, 6, 19]. Hence, we speculated that there might exist protective bioregulators that overexpressed in NP cells under LC loading compression but lost their functions and underexpressed in NP cells that suffered HC loading compression. Based on this hypothesis, we analyzed the DEPs among the NP cells cultured under noncompression, LC, and HC loading compression and predicted eight proteins, which might play as potential protective factors for the NP cell survival under HC loading compression. Among the eight predicted proteins, MIF, a recognized hypoxic stress-associated regulator of innate immunity, was selected for further functional experiments due to its reported similar role in regulating the fate of the human CEP-derived cells [14]. Then, we tested the expression of MIF in both in vitro NP cell 3D-cultured hydrogels and in vivo rat tail IVD compressed models. The results revealed that the expression of MIF was markedly attenuated by HC loading in vitro and overloaded compression in vivo, which were consistent with the proteomics results.

MIF was first identified as a cytokine released from immune or nonimmune cells, including IVD and other types of cartilage cells, which can inhibit the random migration of macrophages and regulate chondroosteogenesis [14, 23, 24]. Despite its wide distribution, MIF secretion is closely related to multiple stresses, such as hypoxic, oxidative, and mechanical stresses [23–26]. MIF is thought as the downstream factor of hypoxia-inducible factor-1 (HIF-1), and the HIF-1 response of the cell is a kind of adaptive response that improves the chances of survival under hypoxic and/or oxidative stress by restoring oxidative homeostasis [25]. In the current study, the analysis of GO and KEGG enrichment also indicated that compression loading affected the fate commitment of NP cells via regulating the ATP biosynthetic process and oxidative phosphorylation. As the prominent organelle for ATP biosynthetic process and oxidative phosphorylation, the mitochondrion is also the main targeting organelle for oxidative stress injury [21]. Noticeably, MIF was reported to play an essential role in regulating many repair/senescence-associated genes via activating autophagy or mitophagy, which is a critical antioxidative stress mechanism to restore intracellular oxygen homeostasis [25, 27]. Our previous studies have evaluated that overloaded compression could trigger oxidative stress-associated mitochondrial dysfunction and induce NP cell senescence, and mitophagy could alleviate oxidative stress-induced senescent changes of NP cell that suffered overloaded compression via clearance of damaged mitochondria. Therefore, we hypothesized that there might exist a relationship between compression-associated mitochondrial damage and mitophagy in NP cell.

The mitochondrion is an essential organelle with multiple functions in the maintenance of intracellular environment homeostasis, not only by regulating ATP production but also by regulating several signal cascades that control cell death, differentiation, and senescence [28]. Mitophagy, a particular form of macroautophagy, can specifically target mitochondria for degradation [29, 30]. In this process, a series of specific receptors or adaptors that mediated pathways were activated to recruit the autophagic machinery towards damaged mitochondria, engulfed and digested by the autophagosomes [29, 30]. The aforementioned results of the current study indicated that HC loading could induce oxidative stress injury and senescence of the human NP cells. Simultaneously, HC loading aggravated mitochondrial dysfunction but retarded activity of mitophagy in NP cells. When we knock down the expression of MIF, the activity of mitophagy was further depressed, which substantially exacerbated the mitochondrial dysfunction, oxidative stress injury, and senescence of the NP cells under overloaded compression. Although mass studies confirmed the mitophagy regulating role of MIF in various types of cells, the specific regulating mechanisms involved in this process were still controversial [27]. The established researches identified and elucidated two signal pathways of mitophagy: ubiquitin- (Ub-) dependent and Ub-independent (receptor-mediated mitophagy) [31]. Some study holds that MIF induces mitophagy via BNIP3-mediated pathway, an Ub-independent manner of mitophagy [32], while some other holds that MIF is the regulator of PINK1/Parkin-mediated mitophagy, the most well-defined Ub-mediated pathway of mitophagy [33]. The results of our prior and current studies elucidated that overload compression-associated mitophagy was related to the PINK1/Parkin-mediated macroautophagy [5]. Noticeably, the other series of studies also evaluated that MIF could protect cardiac cells against overload pressure-induced cardiac hypertrophy via activating Parkin-mediated macroautophagy or mitophagy [26, 34, 35]. Mitophagy-inducing signal cascades are relatively distinct but interconnected [31]. Importantly, mitophagy is dependent on the accumulation of ROS and damaged mitochondria [36]. Deficiency of mitophagy accumulates dysfunctional mitochondria that release ROS into the cytosol and trigger oxidative stress injury [27, 31, 36]. Thus, this is because deficiency of MIF accentuates the oxidative stress damage and senescence of NP cells that suffered overloaded mechanical compression.

There were some limitations in the current study. First, the experimental NP cells in the current study were obtained from the same donor, due to the homogeneity of samples prepared for the proteomics analysis. The collection of clinical patients' IVD samples for the research is ongoing. We have planned to carry out more advanced high-throughput sequencing, such as single-cell sequencing in future studies, which can conclude larger sample capacity and diminish the deviation caused by individual differences. Second, the mechanism behind ROS-induced MIF release remains elusive, and the current study simply focused on its autophagy-inducing role involved in the progress of mechanical compression-related disc degeneration. Hence, more researches on the MIF-associated biological changes in the pathomechanism of disc degeneration will be exerted in our further studies.

## 5. Conclusion

**Figure 9:**
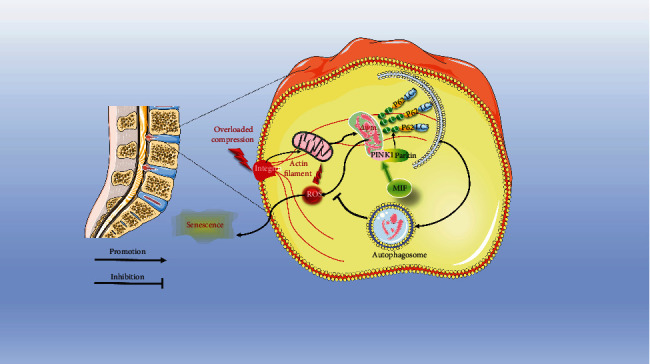
Schematic diagram shows the potential mechanism of MIF in regulating overloaded compression induced NP cell senescence: overloaded compression induces mitochondrial injury and triggers oxidative stress-associated senescence of NP cells. The deficiency of MIF depresses mitophagy in NP cells, which further exacerbates oxidative stress-associated senescence of the NP cells under overloaded compression.

In conclusion, our study reveals that overloaded mechanical compression could induce oxidative stress, mitochondrial dysfunction, and degenerative changes in human NP cells. In contrast, proper physiological pressure is beneficial for maintaining cell viability and ECM homeostasis of the NP. Moreover, there exists a relationship between MIF expression and mechanical compression stress-associated biological changes of NP cells. The deficiency of MIF accentuates the accumulation of ROS, mitochondrial dysfunction, and senescence under overloaded mechanical compression. The potential molecular mechanism involved in this process is related to the mitophagy-inducing role of MIF (Figure 9). This work helps us better understand the regulating function of mechanical compression stress in the progress of disc degeneration and provides more substantial proofs for using MIF-alerting agents in treating disc degenerative diseases.

## Data Availability

The data used to support the findings of this study are available from the corresponding authors upon request.

## Supplementary Materials

Supplementary Materials Supplementary Figure 1: preoperative MRI image of the patient's lumbar IVDs and the gross view of the separated NP tissues. Supplementary Table 1: annotation detail of the DEPs between the LC loading group and the control group. Supplementary Table 2: annotation detail of the DEPs between the HC loading group and the control group. Supplementary Table 3: annotation detail of the DEPs between the HC loading group and the LC loading group.
