# Supplementary material for: Deficiency of MIF Accentuates Overloaded Compression-Induced Nucleus Pulposus Cell Oxidative Damage via Depressing Mitophagy
Source: Oxid Med Cell Longev. 2021 Jul 1;2021:6192498. doi: 10.1155/2021/6192498 (PMC8270705; doi:10.1155/2021/6192498)
Supplement: Supplementary Materials — Supplementary Figure 1: preoperative MRI image of the patient's lumbar IVDs and the gross view of the separated NP tissues. Supplementary Table 1: annotation detail of the DEPs between the LC loading group and the control group. Supplementary Table 2: annotation detail of the DEPs between the HC loading group and the control group. Supplementary Table 3: annotation detail of the DEPs between the HC loading group and the LC loading group. [file 6192498.f1.zip › 6192498.f1.pdf]

Supplemental Material

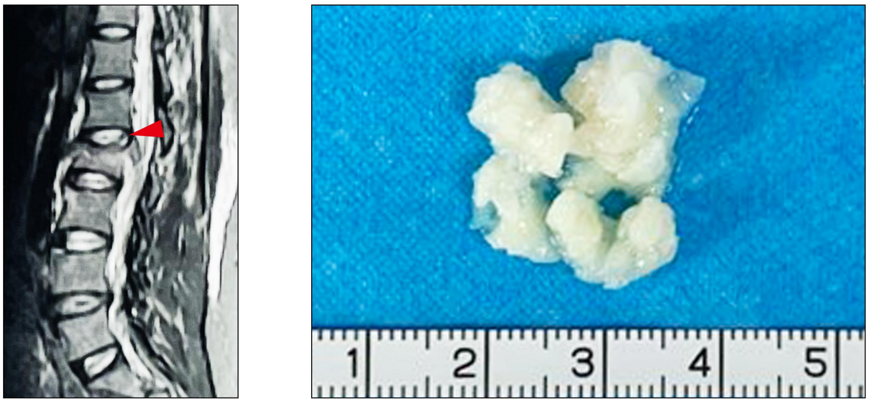

(a) (b)

FIGURE 1: (a) Preoperative MRI image of the donor’s lumbar intervertebral discs. Red arrow represents the segment of the excisional disc. (b) Gross view of the nucleus pulposus tissues separated from the excisional disc.
