# Supplementary material for: Deficiency of MIF Accentuates Overloaded Compression-Induced Nucleus Pulposus Cell Oxidative Damage via Depressing Mitophagy
Source: Oxid Med Cell Longev. 2021 Jul 1;2021:6192498. doi: 10.1155/2021/6192498 (PMC8270705; doi:10.1155/2021/6192498)
Supplement: Supplementary Materials — Supplementary Figure 1: preoperative MRI image of the patient's lumbar IVDs and the gross view of the separated NP tissues. Supplementary Table 1: annotation detail of the DEPs between the LC loading group and the control group. Supplementary Table 2: annotation detail of the DEPs between the HC loading group and the control group. Supplementary Table 3: annotation detail of the DEPs between the HC loading group and the LC loading group. [file 6192498.f1.zip › 6192498.f2.pdf]

Accession_id	Description	KO_id	KO_name
ENSP00000403343.1	angio associated migratory cell protein [Source:HGNC Symbol;Acc:HGNC:18]		
ENSP00000340689.4	glutaminase [Source:HGNC Symbol;Acc:HGNC:4331]	K01425	glsA, GLS
ENSP00000379051.2	purine rich element binding protein B [Source:HGNC Symbol;Acc:HGNC:9702]		
ENSP00000413707.1	leucine rich repeat containing 15 [Source:HGNC Symbol;Acc:HGNC:20818]		
ENSP00000379133.3	APAF1 interacting protein [Source:HGNC Symbol;Acc:HGNC:17581]	K08964	mtnB
ENSP00000376374.2	thymocyte nuclear protein 1 [Source:HGNC Symbol;Acc:HGNC:29560]		
ENSP00000252809.3	growth differentiation factor 15 [Source:HGNC Symbol;Acc:HGNC:30142]	K05504	GDF15
ENSP00000264870.3	coagulation factor XIII A chain [Source:HGNC Symbol;Acc:HGNC:3531]	K03917	F13A1
ENSP00000394898.2	ATP binding cassette subfamily B member 9 [Source:HGNC Symbol;Acc:HGNC:50]	K05656	ABCB9, TAPL
ENSP00000423482.1	calcium/calmodulin dependent protein kinase II delta [Source:HGNC Symbol;Acc:HGNC:1462]		
ENSP00000299633.4	HDGF like 3 [Source:HGNC Symbol;Acc:HGNC:24937]		
ENSP00000353007.4	sulfatase 2 [Source:HGNC Symbol;Acc:HGNC:20392]	K14607	SULF
ENSP00000296280.6	mannan binding lectin serine peptidase 1 [Source:HGNC Symbol;Acc:HGNC:6901]		
ENSP00000464036.2	cyclin dependent kinase 11B [Source:HGNC Symbol;Acc:HGNC:1729]		
ENSP00000468785.1	methyl-CpG binding domain protein 1 [Source:HGNC Symbol;Acc:HGNC:6916]	K11589	MBD1
ENSP00000322061.9	complement C7 [Source:HGNC Symbol;Acc:HGNC:1346]	K03996	C7
ENSP00000499556.1	coiled-coil domain containing 57 [Source:HGNC Symbol;Acc:HGNC:27564]		
ENSP00000386538.3	TBC1 domain family member 10B [Source:HGNC Symbol;Acc:HGNC:24510]	K19944	TBC1D10
ENSP00000499391.1	deleted in malignant brain tumors 1 [Source:HGNC Symbol;Acc:HGNC:2926]		
ENSP00000252252.3	keratin 6B [Source:HGNC Symbol;Acc:HGNC:6444]	K07605	KRT2
ENSP00000361359.3	CD40 molecule [Source:HGNC Symbol;Acc:HGNC:11919]	K03160	TNFRSF5, CD40
ENSP00000395252.1	chromodomain helicase DNA binding protein 3 [Source:HGNC Symbol;Acc:HGNC:1918]	K11642	CHD3, MI2A
ENSP00000306261.4	keratin 78 [Source:HGNC Symbol;Acc:HGNC:28926]	K07605	KRT2
ENSP00000444565.1	transmembrane and ubiquitin like domain containing 2 [Source:HGNC Symbol;Acc:HGNC:28459]		
ENSP00000379430.4	NADH:ubiquinone oxidoreductase complex assembly factor 6 [Source:HGNC Symbol;Acc:H	K18163	NDUFAF6
ENSP00000363891.3	solute carrier family 38 member 10 [Source:HGNC Symbol;Acc:HGNC:28237]	K14996	SLC38A10
ENSP00000232974.6	zinc finger and BTB domain containing 47 [Source:HGNC Symbol;Acc:HGNC:26955]	K10518	ZBTB47
ENSP00000431759.5	cilia and flagella associated protein 54 [Source:HGNC Symbol;Acc:HGNC:26456]		
ENSP00000360519.3	retinol binding protein 4 [Source:HGNC Symbol;Acc:HGNC:9922]	K18271	RBP4
ENSP00000157600.3	LIM and cysteine rich domains 1 [Source:HGNC Symbol;Acc:HGNC:6633]		
ENSP00000343924.2	proline and arginine rich end leucine rich repeat protein [Source:HGNC Symbol;Acc:HGNC	K08125	PRELP
ENSP00000330523.5	collagen triple helix repeat containing 1 [Source:HGNC Symbol;Acc:HGNC:18831]		
ENSP00000486201.1	ADAM metallopeptidase with thrombospondin type 1 motif 13 [Source:HGNC Symbol;Acc:	K08627	ADAMTS13
ENSP00000399168.2	major histocompatibility complex, class I, B [Source:HGNC Symbol;Acc:HGNC:4932]	K06751	MHC1
ENSP00000238044.3	ECRG4 augurin precursor [Source:HGNC Symbol;Acc:HGNC:24642]		
ENSP00000261336.2	PZP alpha-2-macroglobulin like [Source:HGNC Symbol;Acc:HGNC:9750]		
ENSP00000397705.2	major histocompatibility complex, class I, F [Source:HGNC Symbol;Acc:HGNC:4963]	K06751	MHC1

ENSP00000331544.6	fibulin 1 [Source:HGNC Symbol;Acc:HGNC:3600]	K17307	FBLN1_2
ENSP00000358035.5	ADAMTS like 4 [Source:HGNC Symbol;Acc:HGNC:19706]		
ENSP00000360464.3	NFKB activating protein [Source:HGNC Symbol;Acc:HGNC:29873]		
ENSP00000225964.5	collagen type I alpha 1 chain [Source:HGNC Symbol;Acc:HGNC:2197]	K06236	COL1A
ENSP00000432266.1	Yip1 domain family member 1 [Source:HGNC Symbol;Acc:HGNC:25231]		
ENSP00000321983.6	sorbin and SH3 domain containing 2 [Source:HGNC Symbol;Acc:HGNC:24098]		
ENSP00000484804.1	armadillo repeat containing 9 [Source:HGNC Symbol;Acc:HGNC:20730]		
ENSP00000340466.4	glucosidase II alpha subunit [Source:HGNC Symbol;Acc:HGNC:4138]		
ENSP00000460885.1	CORO7-PAM16 readthrough [Source:HGNC Symbol;Acc:HGNC:44424]	K17805;K	PAM16, TIM16;CORO7
ENSP00000359729.4	solute carrier family 9 member A6 [Source:HGNC Symbol;Acc:HGNC:11079]		
ENSP00000296424.4	3-hydroxybutyrate dehydrogenase 2 [Source:HGNC Symbol;Acc:HGNC:32389]	K00019	E1.1.1.30, bdh
ENSP00000354813.2	mitochondrially encoded NADH:ubiquinone oxidoreductase core subunit 5 [Source:HGNC	K03883	ND5
ENSP00000381220.1	HD domain containing 2 [Source:HGNC Symbol;Acc:HGNC:21078]	K07023	K07023
ENSP00000478874.1	calcium/calmodulin dependent protein kinase ID [Source:HGNC Symbol;Acc:HGNC:19341]	K08794	CAMK1
ENSP00000477077.1	PC4 and SFRS1 interacting protein 1 [Source:HGNC Symbol;Acc:HGNC:9527]		
ENSP00000252050.4	cullin 9 [Source:HGNC Symbol;Acc:HGNC:15982]	K11970	CUL9, PARC
ENSP00000359956.5	ELOVL fatty acid elongase 5 [Source:HGNC Symbol;Acc:HGNC:21308]		
ENSP00000399585.2	magnesium transporter MRS2 [Source:HGNC Symbol;Acc:HGNC:13785]		
ENSP00000303057.4	peptidylprolyl isomerase C [Source:HGNC Symbol;Acc:HGNC:9256]	K09563	PPIC, CYPC
ENSP00000222271.2	cartilage oligomeric matrix protein [Source:HGNC Symbol;Acc:HGNC:2227]	K04659	THBS2S
ENSP00000290649.5	autocrine motility factor receptor [Source:HGNC Symbol;Acc:HGNC:463]	K10636	AMFR, GP78
ENSP00000378812.3	coiled-coil-helix-coiled-coil-helix domain containing 2 [Source:HGNC Symbol;Acc:HGNC:21645]		
ENSP00000355994.3	hydroxysteroid 11-beta dehydrogenase 1 [Source:HGNC Symbol;Acc:HGNC:5208]	K15680	HSD11B1
ENSP00000342118.6	HHIP like 2 [Source:HGNC Symbol;Acc:HGNC:25842]		
ENSP00000496842.1	PNN interacting serine and arginine rich protein [Source:HGNC Symbol;Acc:HGNC:21222]	K13170	SFRS18
ENSP00000217133.1	tubulin beta 1 class VI [Source:HGNC Symbol;Acc:HGNC:16257]	K07375	TUBB
ENSP00000483673.1	golgin A6 family like 22 [Source:HGNC Symbol;Acc:HGNC:50289]		
ENSP00000497649.1	tenascin XB [Source:HGNC Symbol;Acc:HGNC:11976]	K06252	TN
ENSP00000347602.3	AT-rich interaction domain 4A [Source:HGNC Symbol;Acc:HGNC:9885]	K19194	ARID4A, RBP1
ENSP00000310170.2	FOS like 1, AP-1 transcription factor subunit [Source:HGNC Symbol;Acc:HGNC:13718]	K04502	FOSL1
ENSP00000354574.3	solute carrier family 9 member B2 [Source:HGNC Symbol;Acc:HGNC:25143]		
ENSP00000364956.3	pleckstrin homology and RUN domain containing M2 [Source:HGNC Symbol;Acc:HGNC:29	K15348	PLEKHM2, SKIP
ENSP00000462172.1	KIAA0040 [Source:HGNC Symbol;Acc:HGNC:28950]		
ENSP00000342805.4	Fas apoptotic inhibitory molecule [Source:HGNC Symbol;Acc:HGNC:18703]		
ENSP00000441858.2	Dmx like 2 [Source:HGNC Symbol;Acc:HGNC:2938]		
ENSP00000343273.4	kelch like family member 7 [Source:HGNC Symbol;Acc:HGNC:15646]	K10445	KLHL7
ENSP00000263277.2	EH domain containing 2 [Source:HGNC Symbol;Acc:HGNC:3243]	K12469	EHD2
ENSP00000316042.4	heterogeneous nuclear ribonucleoprotein A0 [Source:HGNC Symbol;Acc:HGNC:5030]	K12894	HNRNPA0

ENSP00000339723.3	corepressor interacting with RBPJ, 1 [Source:HGNC Symbol;Acc:HGNC:24217]	K06066	CIR
ENSP00000222482.4	carboxypeptidase A4 [Source:HGNC Symbol;Acc:HGNC:15740]	K08637	CPA4
ENSP00000465432.1	chromosome 19 open reading frame 53 [Source:HGNC Symbol;Acc:HGNC:24991]		
ENSP00000419105.1	transgelin 3 [Source:HGNC Symbol;Acc:HGNC:29868]	K20526	TAGLN
ENSP00000406612.2	nudix hydrolase 4 [Source:HGNC Symbol;Acc:HGNC:8051]	K07766	E3.6.1.52
ENSP00000297290.3	brain protein I3 [Source:HGNC Symbol;Acc:HGNC:1109]		
ENSP00000456272.1	TRIM59 and iFT80 readthrough	K12028;K	TRIM59;IFT80
ENSP00000350896.3	EPH receptor B4 [Source:HGNC Symbol;Acc:HGNC:3395]	K05113	EPHB4, HTK
ENSP00000471683.1	solute carrier family 38 member 5 [Source:HGNC Symbol;Acc:HGNC:18070]	K14992	SLC38A5, SNAT5
ENSP00000356037.3	complement component 4 binding protein alpha [Source:HGNC Symbol;Acc:HGNC:1325]	K04002	C4BPA
ENSP00000261405.5	von Willebrand factor [Source:HGNC Symbol;Acc:HGNC:12726]	K03900	VWF
ENSP00000225740.6	aldehyde dehydrogenase 3 family member A1 [Source:HGNC Symbol;Acc:HGNC:405]	K00129	E1.2.1.5
ENSP00000261623.3	cytochrome b-245 alpha chain [Source:HGNC Symbol;Acc:HGNC:2577]	K08009	CYBA, P22PHOX
ENSP00000265978.4	family with sequence similarity 160 member A2 [Source:HGNC Symbol;Acc:HGNC:25378]		
ENSP00000352929.3	casein kinase 1 epsilon [Source:HGNC Symbol;Acc:HGNC:2453]	K08960	CSNK1E
ENSP00000248958.4	stromal cell derived factor 2 like 1 [Source:HGNC Symbol;Acc:HGNC:10676]		
ENSP00000489269.1	synaptotagmin like 2 [Source:HGNC Symbol;Acc:HGNC:15585]		
ENSP00000273853.6	centromere protein C [Source:HGNC Symbol;Acc:HGNC:1854]	K11497	CENPC
ENSP00000324856.6	serine/threonine kinase 11 [Source:HGNC Symbol;Acc:HGNC:11389]	K07298	STK11, LKB1
ENSP00000225577.4	ribosomal protein S6 kinase B1 [Source:HGNC Symbol;Acc:HGNC:10436]	K04688	RPS6KB
ENSP00000354964.2	zinc finger protein 318 [Source:HGNC Symbol;Acc:HGNC:13578]		
ENSP00000276914.2	perilipin 2 [Source:HGNC Symbol;Acc:HGNC:248]	K17284	PLIN2, ADRP
ENSP00000370129.4	aldo-keto reductase family 1 member C2 [Source:HGNC Symbol;Acc:HGNC:385]	K00089	AKR1C2
ENSP00000388446.2	reelin [Source:HGNC Symbol;Acc:HGNC:9957]	K06249	RELN
ENSP00000364979.4	collagen type IV alpha 1 chain [Source:HGNC Symbol;Acc:HGNC:2202]	K06237	COL4A
ENSP00000255030.5	C-reactive protein [Source:HGNC Symbol;Acc:HGNC:2367]		
ENSP00000284268.6	ANKH inorganic pyrophosphate transport regulator [Source:HGNC Symbol;Acc:HGNC:15492]		
ENSP00000323065.1	GADD45G interacting protein 1 [Source:HGNC Symbol;Acc:HGNC:29996]		
ENSP00000356563.4	peroxisomal biogenesis factor 3 [Source:HGNC Symbol;Acc:HGNC:8858]	K13336	PEX3
ENSP00000353654.5	collagen type IV alpha 2 chain [Source:HGNC Symbol;Acc:HGNC:2203]	K06237	COL4A
ENSP00000263408.4	complement C9 [Source:HGNC Symbol;Acc:HGNC:1358]	K04000	C9
ENSP00000450607.1	timeless circadian regulator [Source:HGNC Symbol;Acc:HGNC:11813]	K03155	TIMELESS
ENSP00000297848.3	collagen type XIV alpha 1 chain [Source:HGNC Symbol;Acc:HGNC:2191]	K08133	COL14A
ENSP00000364000.3	collagen type V alpha 2 chain [Source:HGNC Symbol;Acc:HGNC:2210]	K19721	COL5AS
ENSP00000350277.2	plectin [Source:HGNC Symbol;Acc:HGNC:9069]	K10388	PLEC
ENSP00000437673.1	four and a half LIM domains 1 [Source:HGNC Symbol;Acc:HGNC:3702]	K14365	FHL1, SLIM1
ENSP00000333664.8	acetyl-CoA acyltransferase 1 [Source:HGNC Symbol;Acc:HGNC:82]	K07513	ACAA1
ENSP00000346839.4	fibronectin 1 [Source:HGNC Symbol;Acc:HGNC:3778]		

ENSP00000391481.1	transketolase [Source:HGNC Symbol;Acc:HGNC:11834]	K00615	E2.2.1.1, tktA, tktB
ENSP00000355110.3	SPARC related modular calcium binding 1 [Source:HGNC Symbol;Acc:HGNC:20318]		
ENSP00000400759.1	cytochrome c oxidase assembly factor 1 homolog [Source:HGNC Symbol;Acc:HGNC:21868	K18173	COA1
ENSP00000354960.4	collagen beta(1-O)galactosyltransferase 2 [Source:HGNC Symbol;Acc:HGNC:16790]	K11703	GLT25D
ENSP00000231461.4	ST8 alpha-N-acetyl-neuraminide alpha-2,8-sialyltransferase 4 [Source:HGNC Symbol;Acc:	K06614	SIAT8D
ENSP00000223095.4	serpin family E member 1 [Source:HGNC Symbol;Acc:HGNC:8583]	K03982	SERPINE1, PAI1
ENSP00000380159.3	FLYWCH family member 2 [Source:HGNC Symbol;Acc:HGNC:25178]		
ENSP00000379838.2	calcium regulated heat stable protein 1 [Source:HGNC Symbol;Acc:HGNC:17150]		
ENSP00000335620.5	glutathione S-transferase alpha 1 [Source:HGNC Symbol;Acc:HGNC:4626]	K00799	GST, gst
ENSP00000387360.1	multiple coagulation factor deficiency 2 [Source:HGNC Symbol;Acc:HGNC:18451]	K20364	MCFD2
ENSP00000384552.1	fibrinogen gamma chain [Source:HGNC Symbol;Acc:HGNC:3694]	K03905	FGG
ENSP00000414847.3	ADP ribosylation factor like GTPase 6 interacting protein 4 [Source:HGNC Symbol;Acc:HGNC:18076]		
ENSP00000384917.3	sushi, von Willebrand factor type A, EGF and pentraxin domain containing 1 [Source:HGN(	K17495	CSMD
ENSP00000337354.5	lipase A, lysosomal acid type [Source:HGNC Symbol;Acc:HGNC:6617]	K01052	LIPA
ENSP00000263578.5	FAD dependent oxidoreductase domain containing 1 [Source:HGNC Symbol;Acc:HGNC:26	K18166	FOXRED1
ENSP00000280258.4	serine protease 23 [Source:HGNC Symbol;Acc:HGNC:14370]	K09627	PRSS23
ENSP00000286091.5	protein disulfide isomerase family A member 4 [Source:HGNC Symbol;Acc:HGNC:30167]	K09582	PDIA4, ERP72
ENSP00000332449.7	cysteine rich protein 1 [Source:HGNC Symbol;Acc:HGNC:2360]		
ENSP00000419184.2	SET and MYND domain containing 3 [Source:HGNC Symbol;Acc:HGNC:15513]	K11426	SMYD
ENSP00000393887.2	alpha 2-HS glycoprotein [Source:HGNC Symbol;Acc:HGNC:349]		
ENSP00000428115.1	ribonucleotide reductase regulatory TP53 inducible subunit M2B [Source:HGNC Symbol;Acc:HGNC:17296]		
ENSP00000364694.3	asporin [Source:HGNC Symbol;Acc:HGNC:14872]	K08120	ASPN
ENSP00000360718.3	RAB3B, member RAS oncogene family [Source:HGNC Symbol;Acc:HGNC:9778]	K06108	RAB3B
ENSP00000397261.2	selenium binding protein 1 [Source:HGNC Symbol;Acc:HGNC:10719]	K17285	SELENBP1
ENSP00000367817.3	podocalyxin like [Source:HGNC Symbol;Acc:HGNC:9171]	K06817	PODXL
ENSP00000268129.5	abhydrolase domain containing 2 [Source:HGNC Symbol;Acc:HGNC:18717]	K13697	ABHD2
ENSP00000340220.5	chromosome 7 open reading frame 26 [Source:HGNC Symbol;Acc:HGNC:21702]		
ENSP00000267113.4	extended synaptotagmin 1 [Source:HGNC Symbol;Acc:HGNC:29534]		
ENSP00000368242.4	ribonuclease P/MRP subunit p25 like [Source:HGNC Symbol;Acc:HGNC:19909]	K14525	RPP25
ENSP00000364235.3	ring finger protein 5 [Source:HGNC Symbol;Acc:HGNC:10068]	K10666	RNF5
ENSP00000347184.5	huntingtin [Source:HGNC Symbol;Acc:HGNC:4851]	K04533	HD
ENSP00000450758.1	G protein subunit gamma 2 [Source:HGNC Symbol;Acc:HGNC:4404]	K07826	GNG2
ENSP00000360269.2	tumor associated calcium signal transducer 2 [Source:HGNC Symbol;Acc:HGNC:11530]	K17288	TACSTD2
ENSP00000268124.5	DNA polymerase gamma, catalytic subunit [Source:HGNC Symbol;Acc:HGNC:9179]	K02332	POLG1, MIP1
ENSP00000370128.5	solute carrier family 7 member 1 [Source:HGNC Symbol;Acc:HGNC:11057]	K13863	SLC7A1, ATRC1
ENSP00000283752.5	serpin family B member 3 [Source:HGNC Symbol;Acc:HGNC:10569]	K13963	SERPINB
ENSP00000349811.3	FKBP prolyl isomerase 5 [Source:HGNC Symbol;Acc:HGNC:3721]	K09571	FKBP4_5
ENSP00000466897.1	cold inducible RNA binding protein [Source:HGNC Symbol;Acc:HGNC:1982]		

ENSP00000264265.3	latexin [Source:HGNC Symbol;Acc:HGNC:13347]		
ENSP00000261883.4	cartilage intermediate layer protein [Source:HGNC Symbol;Acc:HGNC:1980]		
ENSP00000371434.3	regulatory factor X3 [Source:HGNC Symbol;Acc:HGNC:9984]	K09173	RFX1_2_3
ENSP00000441927.1	sestrin 3 [Source:HGNC Symbol;Acc:HGNC:23060]	K10141	SESN1_3
ENSP00000352696.1	fibronectin 1 [Source:HGNC Symbol;Acc:HGNC:3778]	K05717	FN1
ENSP00000223836.1(	adenylate kinase 1 [Source:HGNC Symbol;Acc:HGNC:361]	K00939	adk, AK
ENSP00000425286.1	transmembrane protein 26 [Source:HGNC Symbol;Acc:HGNC:28550]		
ENSP00000247665.1(	phosphohistidine phosphatase 1 [Source:HGNC Symbol;Acc:HGNC:30033]	K01112	PHPT1
ENSP00000411532.1	DNA topoisomerase II alpha [Source:HGNC Symbol;Acc:HGNC:11989]	K03164	TOP2
ENSP00000263116.2	RAB36, member RAS oncogene family [Source:HGNC Symbol;Acc:HGNC:9775]	K07922	RAB36
ENSP00000304408.3	collagen type III alpha 1 chain [Source:HGNC Symbol;Acc:HGNC:2201]	K19720	COL3A
ENSP00000494022.1	glutamate-ammonia ligase [Source:HGNC Symbol;Acc:HGNC:4341]	K01915	glnA, GLUL
ENSP00000384949.2	pyrroline-5-carboxylate reductase 1 [Source:HGNC Symbol;Acc:HGNC:9721]	K00286	proC
ENSP00000351157.4	thioredoxin domain containing 15 [Source:HGNC Symbol;Acc:HGNC:20652]		
ENSP00000498855.1	calcyphosine [Source:HGNC Symbol;Acc:HGNC:1487]		
ENSP00000477443.1	LSM4 homolog, U6 small nuclear RNA and mRNA degradation associated [Source:HGNC Symbol;Acc:HGNC:17259]		
ENSP00000397908.2	lipin 1 [Source:HGNC Symbol;Acc:HGNC:13345]		
ENSP00000322885.5	deltex E3 ubiquitin ligase 2 [Source:HGNC Symbol;Acc:HGNC:15973]	K06058	DTX
ENSP00000397166.1	LIM domain containing preferred translocation partner in lipoma [Source:HGNC Symbol;Ac	K16676	LPP
ENSP00000377617.2	methylenetetrahydrofolate dehydrogenase (NADP+ dependent) 2, methenyltetrahydrofola	K13403	MTHFD2
ENSP00000297268.6	collagen type I alpha 2 chain [Source:HGNC Symbol;Acc:HGNC:2198]	K06236	COL1A
ENSP00000386043.2	latent transforming growth factor beta binding protein 1 [Source:HGNC Symbol;Acc:HGNC	K19559	LTBP1
ENSP00000360882.3	collagen type V alpha 1 chain [Source:HGNC Symbol;Acc:HGNC:2209]	K19721	COL5AS
ENSP00000476948.1	RNA polymerase II associated protein 2 [Source:HGNC Symbol;Acc:HGNC:25791]	K20827	RPAP2
ENSP00000346874.3	fatty acyl-CoA reductase 1 [Source:HGNC Symbol;Acc:HGNC:26222]	K13356	FAR
ENSP00000216330.3	FKBP prolyl isomerase 3 [Source:HGNC Symbol;Acc:HGNC:3719]	K09570	FKBP3
ENSP00000404464.2	collagen and calcium binding EGF domains 1 [Source:HGNC Symbol;Acc:HGNC:29426]	K19638	CCBE1
ENSP00000297540.4	phosphorylated adaptor for RNA export [Source:HGNC Symbol;Acc:HGNC:10241]	K14291	PHAX
ENSP00000393183.2	DDB1 and CUL4 associated factor 1 [Source:HGNC Symbol;Acc:HGNC:30911]	K11789	VPRBP, DCAF1
ENSP00000373783.3	lysyl oxidase like 2 [Source:HGNC Symbol;Acc:HGNC:6666]	K00280	LOXL2_3_4
ENSP00000348299.4	tripartite motif containing 13 [Source:HGNC Symbol;Acc:HGNC:9976]	K12003	TRIM13
ENSP00000359688.5	ribosome production factor 1 homolog [Source:HGNC Symbol;Acc:HGNC:30350]	K14846	RPF1
ENSP00000481741.1	glutathione S-transferase theta 1 [Source:HGNC Symbol;Acc:HGNC:4641]	K00799	GST, gst
ENSP00000359050.4	armadillo like helical domain containing 3 [Source:HGNC Symbol;Acc:HGNC:25788]		
ENSP00000484472.1	epiplakin 1 [Source:HGNC Symbol;Acc:HGNC:15577]		
ENSP00000498441.1	fibrinogen alpha chain [Source:HGNC Symbol;Acc:HGNC:3661]	K03903	FGA
ENSP00000424571.1	fibrillin 2 [Source:HGNC Symbol;Acc:HGNC:3604]		
ENSP00000264501.4	KIAA1109 [Source:HGNC Symbol;Acc:HGNC:26953]		

ENSP00000361087.3	synuclein gamma [Source:HGNC Symbol;Acc:HGNC:11141]		
ENSP00000298248.7	crystallin lambda 1 [Source:HGNC Symbol;Acc:HGNC:18246]	K13247	CRYL1
ENSP00000240185.3	TAR DNA binding protein [Source:HGNC Symbol;Acc:HGNC:11571]		
ENSP00000451979.1	apurinic/apyrimidinic endodeoxyribonuclease 1 [Source:HGNC Symbol;Acc:HGNC:587]	K10771	APEX1
ENSP00000356382.1	coagulation factor XIII B chain [Source:HGNC Symbol;Acc:HGNC:3534]	K03906	F13B
ENSP00000300086.4	TERF2 interacting protein [Source:HGNC Symbol;Acc:HGNC:19246]	K11113	TERF2IP, RAP1
ENSP00000441434.1	nicotinamide N-methyltransferase [Source:HGNC Symbol;Acc:HGNC:7861]	K00541	NNMT
ENSP00000357721.1	S100 calcium binding protein A8 [Source:HGNC Symbol;Acc:HGNC:10498]	K21127	S100A8
ENSP00000342710.3	keratin 77 [Source:HGNC Symbol;Acc:HGNC:20411]	K07605	KRT2
ENSP00000393312.3	fibroblast growth factor receptor 1 [Source:HGNC Symbol;Acc:HGNC:3688]	K04362	FGFR1, CD331
ENSP00000352798.4	collagen type XVIII alpha 1 chain [Source:HGNC Symbol;Acc:HGNC:2195]	K06823	COL18A
ENSP00000467630.1	coiled-coil domain containing 43 [Source:HGNC Symbol;Acc:HGNC:26472]		
ENSP00000325548.4	carnosine dipeptidase 2 [Source:HGNC Symbol;Acc:HGNC:24437]	K08660	CNDP2
ENSP00000344577.2	apolipoprotein L3 [Source:HGNC Symbol;Acc:HGNC:14868]	K14480	APOL
ENSP00000333666.3	acireductone dioxygenase 1 [Source:HGNC Symbol;Acc:HGNC:30576]	K08967	mtnD, mtnZ, ADI1
ENSP00000295927.3	pentraxin 3 [Source:HGNC Symbol;Acc:HGNC:9692]		
ENSP00000262120.5	twisted gastrulation BMP signaling modulator 1 [Source:HGNC Symbol;Acc:HGNC:12429]		
ENSP00000421315.2	novel protein, MINDY4 and AQP1 readthrough		
ENSP00000233143.4	thymosin beta 10 [Source:HGNC Symbol;Acc:HGNC:11879]	K13785	TMSB10
ENSP00000297785.3	aldehyde dehydrogenase 1 family member A1 [Source:HGNC Symbol;Acc:HGNC:402]	K07249	ALDH1A
ENSP00000409456.1	heterogeneous nuclear ribonucleoprotein K [Source:HGNC Symbol;Acc:HGNC:5044]		
ENSP00000410137.2	neurofilament medium [Source:HGNC Symbol;Acc:HGNC:7734]		
ENSP00000260985.2	isocitrate dehydrogenase (NADP(+)) 1, cytosolic [Source:HGNC Symbol;Acc:HGNC:5382]	K00031	IDH1, IDH2, icd
ENSP00000320447.6	nuclear receptor subfamily 2 group C member 2 [Source:HGNC Symbol;Acc:HGNC:7972]		
ENSP00000386794.2	Rho guanine nucleotide exchange factor 4 [Source:HGNC Symbol;Acc:HGNC:684]		
ENSP00000339328.3	plasminogen activator, urokinase receptor [Source:HGNC Symbol;Acc:HGNC:9053]	K03985	PLAUR, CD87
ENSP00000305839.4	G protein regulated inducer of neurite outgrowth 1 [Source:HGNC Symbol;Acc:HGNC:24835]		
ENSP00000473589.1	S100 calcium binding protein A6 [Source:HGNC Symbol;Acc:HGNC:10496]		
ENSP00000309163.3	Ras converting CAAX endopeptidase 1 [Source:HGNC Symbol;Acc:HGNC:13721]	K08658	RCE1, FACE2
ENSP00000269919.3	pyroglutamyl-peptidase I [Source:HGNC Symbol;Acc:HGNC:13568]	K01304	pcp
ENSP00000369071.4	periostin [Source:HGNC Symbol;Acc:HGNC:16953]		
ENSP00000465759.2	RNA polymerase mitochondrial [Source:HGNC Symbol;Acc:HGNC:9200]	K10908	POLRMT, RPO41
ENSP00000266659.3	GLI pathogenesis related 1 [Source:HGNC Symbol;Acc:HGNC:17001]		
ENSP00000408283.2	profilin 2 [Source:HGNC Symbol;Acc:HGNC:8882]	K05759	PFN
ENSP00000483921.1	single stranded DNA binding protein 2 [Source:HGNC Symbol;Acc:HGNC:15831]		
ENSP00000290705.8	metallothionein 1A [Source:HGNC Symbol;Acc:HGNC:7393]	K14739	MT1_2
ENSP00000288699.6	dihydropyrimidinase like 5 [Source:HGNC Symbol;Acc:HGNC:20637]	K07529	DPYSL5, CRAM
ENSP00000280154.7	programmed cell death 4 [Source:HGNC Symbol;Acc:HGNC:8763]	K16865	PDCD4

ENSP00000378221.1	integrator complex subunit 12 [Source:HGNC Symbol;Acc:HGNC:25067]	K13149	INTS12
ENSP00000410715.2	secreted frizzled related protein 4 [Source:HGNC Symbol;Acc:HGNC:10778]	K02185	SFRP4
ENSP00000481360.1	collagen type V alpha 1 chain [Source:HGNC Symbol;Acc:HGNC:2209]	K19721	COL5AS
ENSP00000319192.5	serine/threonine kinase 17a [Source:HGNC Symbol;Acc:HGNC:11395]	K08804	STK17
ENSP00000342512.2	chromosome 3 open reading frame 33 [Source:HGNC Symbol;Acc:HGNC:26434]		
ENSP00000216286.4	nidogen 2 [Source:HGNC Symbol;Acc:HGNC:13389]	K06826	NID
ENSP00000257192.4	desmoglein 1 [Source:HGNC Symbol;Acc:HGNC:3048]	K07596	DSG1
ENSP00000311572.5	prostaglandin reductase 1 [Source:HGNC Symbol;Acc:HGNC:18429]	K13948	PTGR1, LTB4DH
ENSP00000362929.2	gelsolin [Source:HGNC Symbol;Acc:HGNC:4620]	K05768	GSN
ENSP00000005340.4	dishevelled segment polarity protein 2 [Source:HGNC Symbol;Acc:HGNC:3086]	K02353	DVL
ENSP00000311449.5	RAB6A, member RAS oncogene family [Source:HGNC Symbol;Acc:HGNC:9786]	K07893	RAB6A
ENSP00000431376.1	TatD DNase domain containing 3 [Source:HGNC Symbol;Acc:HGNC:27010]		
ENSP00000370115.5	serpin family B member 1 [Source:HGNC Symbol;Acc:HGNC:3311]	K13963	SERPINB
ENSP00000434657.1	serpin family H member 1 [Source:HGNC Symbol;Acc:HGNC:1546]	K09501	SERPINH1, HSP47
ENSP00000323929.7	alpha-2-macroglobulin [Source:HGNC Symbol;Acc:HGNC:7]	K03910	A2M
ENSP00000445920.1	metabolism of cobalamin associated B [Source:HGNC Symbol;Acc:HGNC:19331]	K00798	MMAB, pduO
ENSP00000358045.4	extracellular matrix protein 1 [Source:HGNC Symbol;Acc:HGNC:3153]		
ENSP00000349878.5	heparin binding growth factor [Source:HGNC Symbol;Acc:HGNC:4856]	K16641	HDGF
ENSP00000331258.5	protein O-mannose kinase [Source:HGNC Symbol;Acc:HGNC:26267]	K17547	SGK196
ENSP00000222390.5	hepatocyte growth factor [Source:HGNC Symbol;Acc:HGNC:4893]	K05460	HGF
ENSP00000158771.4	derlin 2 [Source:HGNC Symbol;Acc:HGNC:17943]	K13989	DERL2_3
ENSP00000371493.4	serpin family F member 2 [Source:HGNC Symbol;Acc:HGNC:9075]	K03983	SERPINF2, AAP
ENSP00000348056.4	ubiquitin conjugating enzyme E2 I [Source:HGNC Symbol;Acc:HGNC:12485]	K10577	UBE2I, UBC9
ENSP00000495507.1	SLIT and NTRK like family member 6 [Source:HGNC Symbol;Acc:HGNC:23503]		
ENSP00000363827.3	heparan sulfate proteoglycan 2 [Source:HGNC Symbol;Acc:HGNC:5273]	K06255	HSPG2
ENSP00000370254.4	aldo-keto reductase family 1 member C1 [Source:HGNC Symbol;Acc:HGNC:384]	K00212	AKR1C1
ENSP00000230048.3	acyl-CoA thioesterase 13 [Source:HGNC Symbol;Acc:HGNC:20999]	K17362	ACOT13
ENSP00000361682.4	leucine rich repeat containing 8 VRAC subunit A [Source:HGNC Symbol;Acc:HGNC:19027]		
ENSP00000478396.1	TELO2 interacting protein 2 [Source:HGNC Symbol;Acc:HGNC:26262]		
ENSP00000285737.4	lon peptidase 2, peroxisomal [Source:HGNC Symbol;Acc:HGNC:20598]	K01338	lon
ENSP00000250784.7	ribosomal protein S4 Y-linked 1 [Source:HGNC Symbol;Acc:HGNC:10425]	K02987	RP-S4e, RPS4
ENSP00000499243.1	cytochrome P450 family 27 subfamily C member 1 [Source:HGNC Symbol;Acc:HGNC:3348(	K17951	CYP27C
ENSP00000339730.2	thrombospondin 4 [Source:HGNC Symbol;Acc:HGNC:11788]	K04659	THBS2S
ENSP00000337746.7	inositol-3-phosphate synthase 1 [Source:HGNC Symbol;Acc:HGNC:29821]	K01858	INO1, ISYNA1
ENSP00000321259.3	transaldolase 1 [Source:HGNC Symbol;Acc:HGNC:11559]	K00616	E2.2.1.2, talA, talB
ENSP00000416951.2	zinc finger CCCH-type containing 18 [Source:HGNC Symbol;Acc:HGNC:25091]		
ENSP00000357980.3	HtrA serine peptidase 1 [Source:HGNC Symbol;Acc:HGNC:9476]	K08784	HTRA1, PRSS11
ENSP00000500914.1	embryonic ectoderm development [Source:HGNC Symbol;Acc:HGNC:3188]	K11462	EED

ENSP00000447000.1	novel protein	K09534;K	SARNP, CIP29, THO1;DNAJ(
ENSP00000310861.3	keratin 2 [Source:HGNC Symbol;Acc:HGNC:6439]	K07605	KRT2
ENSP00000215754.7	macrophage migration inhibitory factor [Source:HGNC Symbol;Acc:HGNC:7097]	K07253	MIF
ENSP00000393896.2	nebulette [Source:HGNC Symbol;Acc:HGNC:16932]		
ENSP00000358719.3	malic enzyme 1 [Source:HGNC Symbol;Acc:HGNC:6983]	K00029	E1.1.1.40, maeB
ENSP00000335255.3	angiopoietin like 5 [Source:HGNC Symbol;Acc:HGNC:19705]		
ENSP00000262962.6	YJU2 splicing factor homolog [Source:HGNC Symbol;Acc:HGNC:25518]		
ENSP00000400839.2	solute carrier family 35 member G2 [Source:HGNC Symbol;Acc:HGNC:28480]		
ENSP00000167586.6	keratin 14 [Source:HGNC Symbol;Acc:HGNC:6416]	K07604	KRT1
ENSP00000296412.8	alcohol dehydrogenase 5 (class III), chi polypeptide [Source:HGNC Symbol;Acc:HGNC:253]	K00121	frmA, ADH5, adhC
ENSP00000356917.2	olfactomedin like 2B [Source:HGNC Symbol;Acc:HGNC:24558]		
ENSP00000357789.1	filaggrin [Source:HGNC Symbol;Acc:HGNC:3748]		
ENSP00000369889.3	collagen type II alpha 1 chain [Source:HGNC Symbol;Acc:HGNC:2200]	K19719	COL2A
ENSP00000333994.3	hemoglobin subunit beta [Source:HGNC Symbol;Acc:HGNC:4827]	K13823	HBB
ENSP00000330075.5	2-oxoglutarate and iron dependent oxygenase domain containing 3 [Source:HGNC Symbol;Acc:HGNC:26174]		
ENSP00000325527.5	fibrillin 1 [Source:HGNC Symbol;Acc:HGNC:3603]	K06825	FBN1
ENSP00000435289.1	platelet activating factor acetylhydrolase 1b catalytic subunit 2 [Source:HGNC Symbol;Acc:I	K16795	PAFAH1B2_3
ENSP00000496785.1	collagen type V alpha 2 chain [Source:HGNC Symbol;Acc:HGNC:2210]	K19721	COL5AS
ENSP00000308938.9	plasminogen [Source:HGNC Symbol;Acc:HGNC:9071]	K01315	PLG
ENSP00000369317.3	keratin 6A [Source:HGNC Symbol;Acc:HGNC:6443]	K07605	KRT2
ENSP00000406293.2	transcription elongation factor A3 [Source:HGNC Symbol;Acc:HGNC:11615]		
ENSP00000372218.1	serpin family B member 12 [Source:HGNC Symbol;Acc:HGNC:14220]		
ENSP00000361608.4	ATP binding cassette subfamily C member 10 [Source:HGNC Symbol;Acc:HGNC:52]	K05674	ABCC10
ENSP00000264187.6	nidogen 1 [Source:HGNC Symbol;Acc:HGNC:7821]	K06826	NID
ENSP00000499582.1	mesencephalic astrocyte derived neurotrophic factor [Source:HGNC Symbol;Acc:HGNC:15461]		
ENSP00000278422.4	thioredoxin related transmembrane protein 2 [Source:HGNC Symbol;Acc:HGNC:30739]		
ENSP00000357643.3	marker of proliferation Ki-67 [Source:HGNC Symbol;Acc:HGNC:7107]	K17582	MKI67
ENSP00000434511.1	selenoprotein H [Source:HGNC Symbol;Acc:HGNC:18251]		
ENSP00000362463.3	glyoxalase I [Source:HGNC Symbol;Acc:HGNC:4323]	K01759	GLO1, gloA
ENSP00000363397.3	UDP-glucose ceramide glucosyltransferase [Source:HGNC Symbol;Acc:HGNC:12524]	K00720	UGCG
ENSP00000378431.1	multimerin 1 [Source:HGNC Symbol;Acc:HGNC:7178]		
ENSP00000497333.1	endothelin converting enzyme 1 [Source:HGNC Symbol;Acc:HGNC:3146]	K01415	ECE
ENSP00000308452.8	keratin 17 [Source:HGNC Symbol;Acc:HGNC:6427]	K07604	KRT1
ENSP00000356671.3	serpin family C member 1 [Source:HGNC Symbol;Acc:HGNC:775]	K03911	SERPINC1, AT3
ENSP00000296721.4	actin filament associated protein 1 like 1 [Source:HGNC Symbol;Acc:HGNC:26714]		
ENSP00000252102.4	NADH:ubiquinone oxidoreductase subunit A2 [Source:HGNC Symbol;Acc:HGNC:7685]	K03946	NDUFA2
ENSP00000332723.2	collectin subfamily member 10 [Source:HGNC Symbol;Acc:HGNC:2220]	K10065	COLEC10
ENSP00000454612.1	cAMP regulated phosphoprotein 19 [Source:HGNC Symbol;Acc:HGNC:16967]		

ENSP00000286234.5	DEP domain containing MTOR interacting protein [Source:HGNC Symbol;Acc:HGNC:22953	K20402	DEPTOR
ENSP00000361850.3	plasminogen activator, urokinase [Source:HGNC Symbol;Acc:HGNC:9052]	K01348	PLAU
ENSP00000463154.1	acidic nuclear phosphoprotein 32 family member E [Source:HGNC Symbol;Acc:HGNC:1667	K18648	ANP32E
ENSP00000394670.1	peptidylprolyl isomerase A [Source:HGNC Symbol;Acc:HGNC:9253]	K03767	PPIA
ENSP00000412922.1	ubiquitin related modifier 1 [Source:HGNC Symbol;Acc:HGNC:28378]	K12161	URM1
ENSP00000497783.1	impact RWD domain protein [Source:HGNC Symbol;Acc:HGNC:20387]		
ENSP00000247470.9	PYD and CARD domain containing [Source:HGNC Symbol;Acc:HGNC:16608]	K12799	PYCARD, ASC
ENSP00000332455.3	karyopherin subunit alpha 2 [Source:HGNC Symbol;Acc:HGNC:6395]	K15043	KPNA2
ENSP00000252242.4	keratin 5 [Source:HGNC Symbol;Acc:HGNC:6442]	K07605	KRT2
ENSP00000481559.1	mitochondrial rRNA methyltransferase 1 [Source:HGNC Symbol;Acc:HGNC:26202]	K15507	MRM1, PET56
ENSP00000459533.1	lipopolysaccharide induced TNF factor [Source:HGNC Symbol;Acc:HGNC:16841]	K19363	LITAF
ENSP00000354718.2	A-kinase anchoring protein 13 [Source:HGNC Symbol;Acc:HGNC:371]	K16529	ARHGEF13
ENSP00000342026.5	peroxiredoxin 6 [Source:HGNC Symbol;Acc:HGNC:16753]	K11188	PRDX6
ENSP00000297313.3	regulator of G protein signaling 20 [Source:HGNC Symbol;Acc:HGNC:14600]	K16449	RGS
ENSP00000247977.3	F-box and leucine rich repeat protein 12 [Source:HGNC Symbol;Acc:HGNC:13611]	K10278	FBXL12
ENSP00000223642.1	complement C5 [Source:HGNC Symbol;Acc:HGNC:1331]	K03994	C5
ENSP00000323780.4	inositol hexakisphosphate kinase 1 [Source:HGNC Symbol;Acc:HGNC:18360]	K07756	IP6K, IHPK
ENSP00000474249.1	NEDD8-MDP1 readthrough [Source:HGNC Symbol;Acc:HGNC:39551]	K12158	NEDD8
ENSP00000292401.4	alpha-2-glycoprotein 1, zinc-binding [Source:HGNC Symbol;Acc:HGNC:910]		
ENSP00000297161.2	BMP binding endothelial regulator [Source:HGNC Symbol;Acc:HGNC:24154]		
ENSP00000250340.3	C-type lectin domain containing 11A [Source:HGNC Symbol;Acc:HGNC:10576]	K17521	CLEC11A
