# Supplementary material for: Deficiency of MIF Accentuates Overloaded Compression-Induced Nucleus Pulposus Cell Oxidative Damage via Depressing Mitophagy
Source: Oxid Med Cell Longev. 2021 Jul 1;2021:6192498. doi: 10.1155/2021/6192498 (PMC8270705; doi:10.1155/2021/6192498)
Supplement: Supplementary Materials — Supplementary Figure 1: preoperative MRI image of the patient's lumbar IVDs and the gross view of the separated NP tissues. Supplementary Table 1: annotation detail of the DEPs between the LC loading group and the control group. Supplementary Table 2: annotation detail of the DEPs between the HC loading group and the control group. Supplementary Table 3: annotation detail of the DEPs between the HC loading group and the LC loading group. [file 6192498.f1.zip › 6192498.f3.pdf]

Accession_id	Description	KO_id	KO_name
ENSP00000269228.4	NPC intracellular cholesterol transporter 1 [Source:HGNC Symbol;Acc:HGNC:7897]	K12385	NPC1
ENSP00000403343.1	angio associated migratory cell protein [Source:HGNC Symbol;Acc:HGNC:18]		
ENSP00000365959.1	F-box protein 44 [Source:HGNC Symbol;Acc:HGNC:24847]	K10103	FBXO44
ENSP00000479636.1	phospholipase C delta 3 [Source:HGNC Symbol;Acc:HGNC:9061]	K05857	PLCD
ENSP00000261407.4	lysophosphatidylcholine acyltransferase 3 [Source:HGNC Symbol;Acc:HGNC:30244]	K13515	LPCAT3, MBOAT5
ENSP00000323889.4	tripartite motif containing 25 [Source:HGNC Symbol;Acc:HGNC:12932]	K10652	TRIM25, EFP
ENSP00000434435.1	family with sequence similarity 111 member A [Source:HGNC Symbol;Acc:HGNC:24725]		
ENSP00000398064.2	HIG1 hypoxia inducible domain family member 1A [Source:HGNC Symbol;Acc:HGNC:29527]		
ENSP00000413707.1	leucine rich repeat containing 15 [Source:HGNC Symbol;Acc:HGNC:20818]		
ENSP00000437955.1	hypoxia inducible factor 1 subunit alpha [Source:HGNC Symbol;Acc:HGNC:4910]		
ENSP00000371388.3	sulfiredoxin 1 [Source:HGNC Symbol;Acc:HGNC:16132]	K12260	SRX1
ENSP00000307705.3	histone cluster 1 H1 family member e [Source:HGNC Symbol;Acc:HGNC:4718]	K11275	H1_5
ENSP00000373073.3	urocanate hydratase 1 [Source:HGNC Symbol;Acc:HGNC:26444]	K01712	hutU, UROC1
ENSP00000486116.1	mitogen-activated protein kinase kinase kinase kinase 4 [Source:HGNC Symbol;Acc:HGNC:6866]		
ENSP00000252809.3	growth differentiation factor 15 [Source:HGNC Symbol;Acc:HGNC:30142]	K05504	GDF15
ENSP00000264870.3	coagulation factor XIII A chain [Source:HGNC Symbol;Acc:HGNC:3531]	K03917	F13A1
ENSP00000387739.2	angiomotin like 1 [Source:HGNC Symbol;Acc:HGNC:17811]	K06104	AMOTL
ENSP00000394898.2	ATP binding cassette subfamily B member 9 [Source:HGNC Symbol;Acc:HGNC:50]	K05656	ABCB9, TAPL
ENSP00000488378.1	paraoxonase 2 [Source:HGNC Symbol;Acc:HGNC:9205]	K01045	PON
ENSP00000312773.4	NFKB inhibitor interacting Ras like 2 [Source:HGNC Symbol;Acc:HGNC:17898]	K17197	NKIRAS
ENSP00000273968.4	PIGY upstream reading frame [Source:HGNC Symbol;Acc:HGNC:44317]		
ENSP00000477781.1	tumor protein, translationally-controlled 1 [Source:HGNC Symbol;Acc:HGNC:12022]		
ENSP00000267884.6	signal recognition particle 14 [Source:HGNC Symbol;Acc:HGNC:11299]	K03104	SRP14
ENSP00000421801.1	HGF activator [Source:HGNC Symbol;Acc:HGNC:4894]	K09631	HGFAC
ENSP00000255194.6	adaptor related protein complex 3 subunit beta 1 [Source:HGNC Symbol;Acc:HGNC:566]	K12397	AP3B
ENSP00000345957.3	ribosomal protein S21 [Source:HGNC Symbol;Acc:HGNC:10409]	K02971	RP-S21e, RPS21
ENSP00000497648.1	minichromosome maintenance complex component 4 [Source:HGNC Symbol;Acc:HGNC:6947]		
ENSP00000338934.7	ezrin [Source:HGNC Symbol;Acc:HGNC:12691]	K08007	VIL2
ENSP00000442057.2	cyclin dependent kinase 2 interacting protein [Source:HGNC Symbol;Acc:HGNC:23789]		
ENSP00000353007.4	sulfatase 2 [Source:HGNC Symbol;Acc:HGNC:20392]	K14607	SULF
ENSP00000260746.4	ADP ribosylation factor like GTPase 3 [Source:HGNC Symbol;Acc:HGNC:694]	K07944	ARL3
ENSP00000272452.2	sulfotransferase family 1C member 4 [Source:HGNC Symbol;Acc:HGNC:11457]	K01025	E2.8.2.-
ENSP00000296280.6	mannan binding lectin serine peptidase 1 [Source:HGNC Symbol;Acc:HGNC:6901]		
ENSP00000245185.5	metallothionein 2A [Source:HGNC Symbol;Acc:HGNC:7406]	K14739	MT1_2
ENSP00000265983.3	hemopexin [Source:HGNC Symbol;Acc:HGNC:5171]	K18977	HPX
ENSP00000453581.2	aggrecan [Source:HGNC Symbol;Acc:HGNC:319]	K06792	AGC1, CSPG1
ENSP00000463574.1	acid phosphatase 6, lysophosphatidic [Source:HGNC Symbol;Acc:HGNC:29609]	K14395	ACP6

ENSP00000482106.1	DEAD-box helicase 24 [Source:HGNC Symbol;Acc:HGNC:13266]	K14805	DDX24, MAK5
ENSP00000322061.9	complement C7 [Source:HGNC Symbol;Acc:HGNC:1346]	K03996	C7
ENSP00000356652.2	calcyclin binding protein [Source:HGNC Symbol;Acc:HGNC:30423]	K04507	CACYBP, SIP
ENSP00000356770.3	coagulation factor V [Source:HGNC Symbol;Acc:HGNC:3542]	K03902	F5
ENSP00000304642.9	reticulophagy regulator 1 [Source:HGNC Symbol;Acc:HGNC:25964]		
ENSP00000234590.4	enolase 1 [Source:HGNC Symbol;Acc:HGNC:3350]	K01689	ENO, eno
ENSP00000231061.4	secreted protein acidic and cysteine rich [Source:HGNC Symbol;Acc:HGNC:11219]		
ENSP00000054950.3	reticulocalbin 1 [Source:HGNC Symbol;Acc:HGNC:9934]		
ENSP00000436759.1	signal peptidase complex subunit 2 [Source:HGNC Symbol;Acc:HGNC:28962]		
ENSP00000499391.1	deleted in malignant brain tumors 1 [Source:HGNC Symbol;Acc:HGNC:2926]		
ENSP00000417806.1	small integral membrane protein 4 [Source:HGNC Symbol;Acc:HGNC:37257]		
ENSP00000292907.3	cytochrome c oxidase subunit 7A1 [Source:HGNC Symbol;Acc:HGNC:2287]	K02270	COX7A
ENSP00000357452.3	phosphomevalonate kinase [Source:HGNC Symbol;Acc:HGNC:9141]	K13273	PMVK
ENSP00000348010.6	pre-mRNA processing factor 39 [Source:HGNC Symbol;Acc:HGNC:20314]	K13217	PRPF39, PRP39
ENSP00000353408.5	moesin [Source:HGNC Symbol;Acc:HGNC:7373]	K05763	MSN
ENSP00000225614.1	galactokinase 1 [Source:HGNC Symbol;Acc:HGNC:4118]	K00849	galK
ENSP00000221233.3	exosome component 5 [Source:HGNC Symbol;Acc:HGNC:24662]	K12590	RRP46, EXOSC5
ENSP00000427722.1	inositol polyphosphate-4-phosphatase type I A [Source:HGNC Symbol;Acc:HGNC:6074]	K01109	INPP4
ENSP00000395252.1	chromodomain helicase DNA binding protein 3 [Source:HGNC Symbol;Acc:HGNC:1918]	K11642	CHD3, MI2A
ENSP00000299492.4	PPFIA binding protein 2 [Source:HGNC Symbol;Acc:HGNC:9250]		
ENSP00000306261.4	keratin 78 [Source:HGNC Symbol;Acc:HGNC:28926]	K07605	KRT2
ENSP00000497532.1	solute carrier family 25 member 38 [Source:HGNC Symbol;Acc:HGNC:26054]	K15118	SLC25A38
ENSP00000379430.4	NADH:ubiquinone oxidoreductase complex assembly factor 6 [Source:HGNC Symbol;Acc:H(	K18163	NDUFAF6
ENSP00000380178.2	ubiquitin conjugating enzyme E2 G1 [Source:HGNC Symbol;Acc:HGNC:12482]	K10575	UBE2G1, UBC7
ENSP00000360519.3	retinol binding protein 4 [Source:HGNC Symbol;Acc:HGNC:9922]	K18271	RBP4
ENSP00000421096.1	phosphoribosylaminoimidazole carboxylase and phosphoribosylaminoimidazolesuccinocarl	K01587	PAICS
ENSP00000259808.3	receptor interacting serine/threonine kinase 1 [Source:HGNC Symbol;Acc:HGNC:10019]	K02861	RIPK1, RIP1
ENSP00000330523.5	collagen triple helix repeat containing 1 [Source:HGNC Symbol;Acc:HGNC:18831]		
ENSP00000306991.4	hyaluronan synthase 2 [Source:HGNC Symbol;Acc:HGNC:4819]	K00752	hasA
ENSP00000486201.1	ADAM metallopeptidase with thrombospondin type 1 motif 13 [Source:HGNC Symbol;Acc:H	K08627	ADAMTS13
ENSP00000399168.2	major histocompatibility complex, class I, B [Source:HGNC Symbol;Acc:HGNC:4932]	K06751	MHC1
ENSP00000374455.4	sequestosome 1 [Source:HGNC Symbol;Acc:HGNC:11280]	K14381	SQSTM1
ENSP00000261336.2	PZP alpha-2-macroglobulin like [Source:HGNC Symbol;Acc:HGNC:9750]		
ENSP00000397705.2	major histocompatibility complex, class I, F [Source:HGNC Symbol;Acc:HGNC:4963]	K06751	MHC1
ENSP00000331544.6	fibulin 1 [Source:HGNC Symbol;Acc:HGNC:3600]	K17307	FBLN1_2
ENSP00000358035.5	ADAMTS like 4 [Source:HGNC Symbol;Acc:HGNC:19706]		
ENSP00000266025.3	transmembrane protein 115 [Source:HGNC Symbol;Acc:HGNC:30055]		
ENSP00000225964.5	collagen type I alpha 1 chain [Source:HGNC Symbol;Acc:HGNC:2197]	K06236	COL1A

ENSP00000451828.1	AKT serine/threonine kinase 1 [Source:HGNC Symbol;Acc:HGNC:391]		
ENSP00000359958.3	zinc finger RANBP2-type containing 2 [Source:HGNC Symbol;Acc:HGNC:13058]		
ENSP00000432266.1	Yip1 domain family member 1 [Source:HGNC Symbol;Acc:HGNC:25231]		
ENSP00000321983.6	sorbin and SH3 domain containing 2 [Source:HGNC Symbol;Acc:HGNC:24098]		
ENSP00000387858.2	stathmin 1 [Source:HGNC Symbol;Acc:HGNC:6510]	K04381	STMN1
ENSP00000496799.1	myosin IC [Source:HGNC Symbol;Acc:HGNC:7597]	K10356	MYO1
ENSP00000484804.1	armadillo repeat containing 9 [Source:HGNC Symbol;Acc:HGNC:20730]		
ENSP00000251595.6	hemoglobin subunit alpha 2 [Source:HGNC Symbol;Acc:HGNC:4824]	K13822	HBA
ENSP00000252486.3	apolipoprotein E [Source:HGNC Symbol;Acc:HGNC:613]		
ENSP00000266085.5	TIMP metallopeptidase inhibitor 3 [Source:HGNC Symbol;Acc:HGNC:11822]	K16866	TIMP3
ENSP00000415998.2	solute carrier family 48 member 1 [Source:HGNC Symbol;Acc:HGNC:26035]	K15380	SLC48A1, HRG1
ENSP00000206423.3	coiled-coil domain containing 80 [Source:HGNC Symbol;Acc:HGNC:30649]		
ENSP00000265056.7	minichromosome maintenance complex component 2 [Source:HGNC Symbol;Acc:HGNC:69	K02540	MCM2
ENSP00000242057.4	aryl hydrocarbon receptor [Source:HGNC Symbol;Acc:HGNC:348]	K09093	AHR
ENSP00000308546.2	methylphosphate capping enzyme [Source:HGNC Symbol;Acc:HGNC:20247]	K15190	MEPCE, BCDIN3
ENSP00000216797.5	NFKB inhibitor alpha [Source:HGNC Symbol;Acc:HGNC:7797]	K04734	NFKBIA
ENSP00000341483.5	RAN binding protein 3 [Source:HGNC Symbol;Acc:HGNC:9850]	K15304	RANBP3
ENSP00000416994.2	alpha tubulin acetyltransferase 1 [Source:HGNC Symbol;Acc:HGNC:21186]		
ENSP00000324422.5	zyxin [Source:HGNC Symbol;Acc:HGNC:13200]	K06273	ZYX
ENSP00000349575.2	MOB kinase activator 3A [Source:HGNC Symbol;Acc:HGNC:29802]		
ENSP00000437109.1	FYVE, RhoGEF and PH domain containing 4 [Source:HGNC Symbol;Acc:HGNC:19125]	K05723	FGD4
ENSP00000478061.1	myristoylated alanine rich protein kinase C substrate [Source:HGNC Symbol;Acc:HGNC:675[illegible]	K12561	MARCKS
ENSP00000364709.3	coagulation factor X [Source:HGNC Symbol;Acc:HGNC:3528]	K01314	F10
ENSP00000260731.3	kinesin family member 11 [Source:HGNC Symbol;Acc:HGNC:6388]	K10398	KIF11, EG5
ENSP00000274276.3	oncostatin M receptor [Source:HGNC Symbol;Acc:HGNC:8507]	K05057	OSMR
ENSP00000379928.1	syntaxin binding protein 6 [Source:HGNC Symbol;Acc:HGNC:19666]	K08519	STXBP6
ENSP00000371798.3	fascin actin-bundling protein 1 [Source:HGNC Symbol;Acc:HGNC:11148]	K17455	FSCN1_2
ENSP00000249700.4	tropomodulin 2 [Source:HGNC Symbol;Acc:HGNC:11872]	K10370	TMOD
ENSP00000484343.1	serpin family B member 6 [Source:HGNC Symbol;Acc:HGNC:8950]	K13963	SERPINB
ENSP00000274008.3	spermatogenesis associated 5 [Source:HGNC Symbol;Acc:HGNC:18119]	K14575	AFG2, DRG1, SPATA5
ENSP00000264896.2	scavenger receptor class B member 2 [Source:HGNC Symbol;Acc:HGNC:1665]	K12384	SCARB2, LIMP2, CD36L2
ENSP00000478874.1	calcium/calmodulin dependent protein kinase ID [Source:HGNC Symbol;Acc:HGNC:19341]	K08794	CAMK1
ENSP00000350893.2	CD46 molecule [Source:HGNC Symbol;Acc:HGNC:6953]	K04007	CD46, MCP
ENSP00000477077.1	PC4 and SFRS1 interacting protein 1 [Source:HGNC Symbol;Acc:HGNC:9527]		
ENSP00000358777.2	ATPase H+ transporting accessory protein 1 [Source:HGNC Symbol;Acc:HGNC:868]	K03662	ATPeVS1, ATP6S1
ENSP00000427976.1	peptidylprolyl isomerase A [Source:HGNC Symbol;Acc:HGNC:9253]	K03767	PPIA
ENSP00000252050.4	cullin 9 [Source:HGNC Symbol;Acc:HGNC:15982]	K11970	CUL9, PARC
ENSP00000336775.7	synemin [Source:HGNC Symbol;Acc:HGNC:24466]	K10376	DMN

ENSP00000484390.1	death associated protein kinase 2 [Source:HGNC Symbol;Acc:HGNC:2675]		
ENSP00000418194.2	poly(ADP-ribose) polymerase family member 14 [Source:HGNC Symbol;Acc:HGNC:29232]	K15261	PARP10_14_15
ENSP00000262948.3	mitogen-activated protein kinase kinase 2 [Source:HGNC Symbol;Acc:HGNC:6842]	K04369	MAP2K2, MEK2
ENSP00000294129.2	NCK interacting protein with SH3 domain [Source:HGNC Symbol;Acc:HGNC:15486]		
ENSP00000332790.3	histone cluster 2 H2A family member b [Source:HGNC Symbol;Acc:HGNC:20508]	K11251	H2A
ENSP00000344937.4	dpy-19 like C-mannosyltransferase 3 [Source:HGNC Symbol;Acc:HGNC:27120]		
ENSP00000468578.1	microfibril associated protein 4 [Source:HGNC Symbol;Acc:HGNC:7035]		
ENSP00000466214.1	programmed cell death 5 [Source:HGNC Symbol;Acc:HGNC:8764]	K06875	PDCD5, TFAR19
ENSP00000362092.3	Ras related GTP binding C [Source:HGNC Symbol;Acc:HGNC:19902]	K16186	RRAGC_D
ENSP00000384164.1	kinesin family member 16B [Source:HGNC Symbol;Acc:HGNC:15869]	K17916	KIF16B, SNX23
ENSP00000419506.1	poly(ADP-ribose) polymerase family member 9 [Source:HGNC Symbol;Acc:HGNC:24118]		
ENSP00000416293.2	solute carrier family 2 member 1 [Source:HGNC Symbol;Acc:HGNC:11005]	K07299	SLC2A1, GLUT1
ENSP00000334754.6	TEA domain transcription factor 1 [Source:HGNC Symbol;Acc:HGNC:11714]	K09448	TEAD
ENSP00000261875.5	3-hydroxyacyl-CoA dehydratase 3 [Source:HGNC Symbol;Acc:HGNC:24175]	K10703	PHS1, PAS2
ENSP00000222271.2	cartilage oligomeric matrix protein [Source:HGNC Symbol;Acc:HGNC:2227]	K04659	THBS2S
ENSP00000265070.6	golgi phosphoprotein 3 [Source:HGNC Symbol;Acc:HGNC:15452]	K15620	GOLPH3, GPP34
ENSP00000378812.3	coiled-coil-helix-coiled-coil-helix domain containing 2 [Source:HGNC Symbol;Acc:HGNC:21645]		
ENSP00000357704.1	S100 calcium binding protein A4 [Source:HGNC Symbol;Acc:HGNC:10494]		
ENSP00000386264.2	transmembrane protein 237 [Source:HGNC Symbol;Acc:HGNC:14432]		
ENSP00000427793.1	WW domain containing E3 ubiquitin protein ligase 1 [Source:HGNC Symbol;Acc:HGNC:170(	K05633	AIP5, WWP1
ENSP00000367044.1	ribosome binding protein 1 [Source:HGNC Symbol;Acc:HGNC:10448]		
ENSP00000343471.2	IKBKB interacting protein [Source:HGNC Symbol;Acc:HGNC:26430]		
ENSP00000356329.2	retinoic acid early transcript 1G [Source:HGNC Symbol;Acc:HGNC:16795]	K07987	RAET1
ENSP00000494544.1	dual specificity tyrosine phosphorylation regulated kinase 1A [Source:HGNC Symbol;Acc:HG	K08825	DYRK1
ENSP00000380921.3	SH3 domain containing kinase binding protein 1 [Source:HGNC Symbol;Acc:HGNC:13867]	K12470	SH3KBP1, CIN85
ENSP00000451932.1	NME1-NME2 readthrough [Source:HGNC Symbol;Acc:HGNC:33531]	K00940	ndk, NME
ENSP00000226218.4	vitronectin [Source:HGNC Symbol;Acc:HGNC:12724]	K06251	VTN
ENSP00000297350.4	TNF receptor superfamily member 11b [Source:HGNC Symbol;Acc:HGNC:11909]	K05148	TNFRSF11B, OPG
ENSP00000361927.2	heterogeneous nuclear ribonucleoprotein H2 [Source:HGNC Symbol;Acc:HGNC:5042]	K12898	HNRNPF_H
ENSP00000370150.4	WRN helicase interacting protein 1 [Source:HGNC Symbol;Acc:HGNC:20876]	K07478	ycaJ
ENSP00000342385.4	prostaglandin E synthase [Source:HGNC Symbol;Acc:HGNC:9599]	K15729	PTGES
ENSP00000406061.2	spartin [Source:HGNC Symbol;Acc:HGNC:18514]	K19366	SPG20
ENSP00000385410.1	nucleolar and coiled-body phosphoprotein 1 [Source:HGNC Symbol;Acc:HGNC:15608]		
ENSP00000299608.2	thioredoxin related transmembrane protein 3 [Source:HGNC Symbol;Acc:HGNC:24718]	K09585	TXNDC10
ENSP00000479889.2	interleukin 1 receptor associated kinase 4 [Source:HGNC Symbol;Acc:HGNC:17967]	K04733	IRAK4
ENSP00000362730.5	mitochondrial carrier 1 [Source:HGNC Symbol;Acc:HGNC:17586]	K17885	MTCH
ENSP00000331849.3	mitochondrial ribosomal protein L54 [Source:HGNC Symbol;Acc:HGNC:16685]	K17435	MRPL54
ENSP00000448228.1	spermatogenesis associated serine rich 2 [Source:HGNC Symbol;Acc:HGNC:18650]		

ENSP00000390968.1	phosphate cytidylyltransferase 1, choline, alpha [Source:HGNC Symbol;Acc:HGNC:8754]	K00968	PCYT1
ENSP00000497649.1	tenascin XB [Source:HGNC Symbol;Acc:HGNC:11976]	K06252	TN
ENSP00000387216.1	protein phosphatase 3 regulatory subunit B, alpha [Source:HGNC Symbol;Acc:HGNC:9317]	K06268	PPP3R, CNB
ENSP00000347602.3	AT-rich interaction domain 4A [Source:HGNC Symbol;Acc:HGNC:9885]	K19194	ARID4A, RBP1
ENSP00000215909.5	galectin 1 [Source:HGNC Symbol;Acc:HGNC:6561]	K06830	LGALS1
ENSP00000376352.2	pyruvate dehydrogenase kinase 1 [Source:HGNC Symbol;Acc:HGNC:8809]		
ENSP00000342951.3	ATPase H+ transporting V0 subunit a1 [Source:HGNC Symbol;Acc:HGNC:865]	K02154	ATPeV0A, ATP6N
ENSP00000365606.5	GRIP1 associated protein 1 [Source:HGNC Symbol;Acc:HGNC:18706]		
ENSP00000309126.8	NOP2/Sun RNA methyltransferase 5 [Source:HGNC Symbol;Acc:HGNC:16385]	K15264	NSUN5, WBSCR20, RCM1
ENSP00000462172.1	KIAA0040 [Source:HGNC Symbol;Acc:HGNC:28950]		
ENSP00000326342.3	ELMO domain containing 2 [Source:HGNC Symbol;Acc:HGNC:28111]		
ENSP00000342805.4	Fas apoptotic inhibitory molecule [Source:HGNC Symbol;Acc:HGNC:18703]		
ENSP00000295797.4	protein kinase C iota [Source:HGNC Symbol;Acc:HGNC:9404]	K06069	PRKCI
ENSP00000312458.2	ARV1 homolog, fatty acid homeostasis modulator [Source:HGNC Symbol;Acc:HGNC:29561]		
ENSP00000263277.2	EH domain containing 2 [Source:HGNC Symbol;Acc:HGNC:3243]	K12469	EHD2
ENSP00000316042.4	heterogeneous nuclear ribonucleoprotein A0 [Source:HGNC Symbol;Acc:HGNC:5030]	K12894	HNRNPA0
ENSP00000261813.4	prefoldin subunit 1 [Source:HGNC Symbol;Acc:HGNC:8866]	K09548	PFDN1
ENSP00000319141.4	cytochrome b reductase 1 [Source:HGNC Symbol;Acc:HGNC:20797]	K08370	CYBRD1, Dcytb
ENSP00000347041.4	fibromodulin [Source:HGNC Symbol;Acc:HGNC:3774]	K08121	FMOD
ENSP00000170447.7	makorin ring finger protein 2 [Source:HGNC Symbol;Acc:HGNC:7113]	K15687	MKRN
ENSP00000352516.3	DNA methyltransferase 1 [Source:HGNC Symbol;Acc:HGNC:2976]	K00558	DNMT1, dcm
ENSP00000362682.3	olfactomedin like 2A [Source:HGNC Symbol;Acc:HGNC:27270]		
ENSP00000222482.4	carboxypeptidase A4 [Source:HGNC Symbol;Acc:HGNC:15740]	K08637	CPA4
ENSP00000358374.3	SR-related CTD associated factor 11 [Source:HGNC Symbol;Acc:HGNC:10784]		
ENSP00000483600.1	leukocyte receptor cluster member 9 [Source:HGNC Symbol;Acc:HGNC:16306]		
ENSP00000377374.3	endoplasmic reticulum-golgi intermediate compartment 1 [Source:HGNC Symbol;Acc:HGN(	K20365	ERGIC1
ENSP00000356954.3	cellular communication network factor 2 [Source:HGNC Symbol;Acc:HGNC:2500]	K06827	CTGF
ENSP00000300177.4	gremlin 1, DAN family BMP antagonist [Source:HGNC Symbol;Acc:HGNC:2001]		
ENSP00000405730.2	apolipoprotein M [Source:HGNC Symbol;Acc:HGNC:13916]		
ENSP00000265074.8	natriuretic peptide receptor 3 [Source:HGNC Symbol;Acc:HGNC:7945]	K12325	NPR3
ENSP00000297290.3	brain protein I3 [Source:HGNC Symbol;Acc:HGNC:1109]		
ENSP00000383178.3	diaphanous related formin 3 [Source:HGNC Symbol;Acc:HGNC:15480]	K05745	DIAPH3, DRF3
ENSP00000421664.1	PDZ and LIM domain 7 [Source:HGNC Symbol;Acc:HGNC:22958]		
ENSP00000498538.1	DIMT1 rRNA methyltransferase and ribosome maturation factor [Source:HGNC Symbol;Acc:	K14191	DIM1
ENSP00000498361.1	transforming acidic coiled-coil containing protein 3 [Source:HGNC Symbol;Acc:HGNC:1152	K14283	TACC3, maskin
ENSP00000350896.3	EPH receptor B4 [Source:HGNC Symbol;Acc:HGNC:3395]	K05113	EPHB4, HTK
ENSP00000433979.1	MAF1 homolog, negative regulator of RNA polymerase III [Source:HGNC Symbol;Acc:HGNC:24966]		
ENSP00000471683.1	solute carrier family 38 member 5 [Source:HGNC Symbol;Acc:HGNC:18070]	K14992	SLC38A5, SNAT5

ENSP00000490312.1	EFR3 homolog A [Source:HGNC Symbol;Acc:HGNC:28970]		
ENSP00000356037.3	complement component 4 binding protein alpha [Source:HGNC Symbol;Acc:HGNC:1325]	K04002	C4BPA
ENSP00000358160.2	histone cluster 1 H3 family member h [Source:HGNC Symbol;Acc:HGNC:4775]	K11253	H3
ENSP00000261405.5	von Willebrand factor [Source:HGNC Symbol;Acc:HGNC:12726]	K03900	VWF
ENSP00000333298.4	lysosomal associated membrane protein 1 [Source:HGNC Symbol;Acc:HGNC:6499]	K06528	LAMP1_2, CD107
ENSP00000403153.2	apolipoprotein L2 [Source:HGNC Symbol;Acc:HGNC:619]	K14480	APOL
ENSP00000339566.2	histone cluster 1 H1 family member c [Source:HGNC Symbol;Acc:HGNC:4716]	K11275	H1_5
ENSP00000356989.3	prefoldin subunit 2 [Source:HGNC Symbol;Acc:HGNC:8867]	K09549	PFDN2
ENSP00000306010.6	ADP ribosylation factor 4 [Source:HGNC Symbol;Acc:HGNC:655]	K07939	ARF4
ENSP00000378699.3	cyclin dependent kinase 1 [Source:HGNC Symbol;Acc:HGNC:1722]	K02087	CDK1, CDC2
ENSP00000349324.3	TNF receptor superfamily member 10c [Source:HGNC Symbol;Acc:HGNC:11906]	K04722	TNFRSF10, TRAILR, CD261,
ENSP00000433240.1	midkine [Source:HGNC Symbol;Acc:HGNC:6972]		
ENSP00000261167.2	WW domain binding protein 11 [Source:HGNC Symbol;Acc:HGNC:16461]	K12866	WBP11, NPWBP
ENSP00000303992.4	transmembrane protein 43 [Source:HGNC Symbol;Acc:HGNC:28472]		
ENSP00000316881.5	prolyl 3-hydroxylase 2 [Source:HGNC Symbol;Acc:HGNC:19317]		
ENSP00000378958.3	RAB, member of RAS oncogene family like 2B [Source:HGNC Symbol;Acc:HGNC:9800]		
ENSP00000425634.1	histidyl-tRNA synthetase [Source:HGNC Symbol;Acc:HGNC:4816]	K01892	HARS, hisS
ENSP00000363349.3	sorting nexin family member 30 [Source:HGNC Symbol;Acc:HGNC:23685]	K17921	SNX7_30
ENSP00000264657.4	signal transducer and activator of transcription 3 [Source:HGNC Symbol;Acc:HGNC:11364]	K04692	STAT3
ENSP00000457703.1	ubiquitin like 7 [Source:HGNC Symbol;Acc:HGNC:28221]		
ENSP00000358022.2	MCL1 apoptosis regulator, BCL2 family member [Source:HGNC Symbol;Acc:HGNC:6943]	K02539	MCL1
ENSP00000273258.3	ADP ribosylation factor like GTPase 6 interacting protein 5 [Source:HGNC Symbol;Acc:HGN(	K20393	ARL6IP5, PRAF3
ENSP00000306124.3	protein kinase C epsilon [Source:HGNC Symbol;Acc:HGNC:9401]	K18050	PRKCE
ENSP00000426083.1	alcohol dehydrogenase 1C (class I), gamma polypeptide [Source:HGNC Symbol;Acc:HGNC:2	K13951	ADH1_7
ENSP00000239440.4	ArfGAP with RhoGAP domain, ankyrin repeat and PH domain 3 [Source:HGNC Symbol;Acc:H	K12490	ARAP3
ENSP00000225577.4	ribosomal protein S6 kinase B1 [Source:HGNC Symbol;Acc:HGNC:10436]	K04688	RPS6KB
ENSP00000317159.4	cytochrome c1 [Source:HGNC Symbol;Acc:HGNC:2579]	K00413	CYC1, CYT1, petC
ENSP00000281938.2	heat shock protein family B (small) member 8 [Source:HGNC Symbol;Acc:HGNC:30171]	K08879	HSPB8
ENSP00000482613.1	high mobility group nucleosomal binding domain 3 [Source:HGNC Symbol;Acc:HGNC:12312]		
ENSP00000315768.4	signal transducer and activator of transcription 2 [Source:HGNC Symbol;Acc:HGNC:11363]	K11221	STAT2
ENSP00000401988.1	caldesmon 1 [Source:HGNC Symbol;Acc:HGNC:1441]	K12327	CALD1
ENSP00000308610.5	glycerol-3-phosphate dehydrogenase 2 [Source:HGNC Symbol;Acc:HGNC:4456]	K00111	glpA, glpD
ENSP00000462880.1	annexin A8 [Source:HGNC Symbol;Acc:HGNC:546]	K17096	ANXA8
ENSP00000376678.4	transgelin [Source:HGNC Symbol;Acc:HGNC:11553]	K20526	TAGLN
ENSP00000333277.1	histone cluster 2 H3 family member d [Source:HGNC Symbol;Acc:HGNC:25311]	K11253	H3
ENSP00000356047.3	6-phosphofructo-2-kinase/fructose-2,6-biphosphatase 2 [Source:HGNC Symbol;Acc:HGNC	K19029	PFKFB2
ENSP00000262030.3	ATP synthase F1 subunit beta [Source:HGNC Symbol;Acc:HGNC:830]	K02133	ATPeF1B, ATP5B, ATP2
ENSP00000356107.3	RAB29, member RAS oncogene family [Source:HGNC Symbol;Acc:HGNC:9789]	K07916	RAB7L1, RAB7L

ENSP00000360540.3	centrosomal protein 55 [Source:HGNC Symbol;Acc:HGNC:1161]	K16456	CEP55
ENSP00000252622.8	LSM7 homolog, U6 small nuclear RNA and mRNA degradation associated [Source:HGNC Sy	K12626	LSM7
ENSP00000370129.4	aldo-keto reductase family 1 member C2 [Source:HGNC Symbol;Acc:HGNC:385]	K00089	AKR1C2
ENSP00000331106.5	peroxisomal biogenesis factor 26 [Source:HGNC Symbol;Acc:HGNC:22965]	K13340	PEX26
ENSP00000388446.2	reelin [Source:HGNC Symbol;Acc:HGNC:9957]	K06249	RELN
ENSP00000294360.4	CXXC motif containing zinc binding protein [Source:HGNC Symbol;Acc:HGNC:26059]		
ENSP00000320650.3	DnaJ heat shock protein family (Hsp40) member C25 [Source:HGNC Symbol;Acc:HGNC:341	K19371	DNAJC25
ENSP00000364979.4	collagen type IV alpha 1 chain [Source:HGNC Symbol;Acc:HGNC:2202]	K06237	COL4A
ENSP00000356853.4	uridine-cytidine kinase 2 [Source:HGNC Symbol;Acc:HGNC:12562]	K00876	udk, UCK
ENSP00000255030.5	C-reactive protein [Source:HGNC Symbol;Acc:HGNC:2367]		
ENSP00000355809.2	ENAH actin regulator [Source:HGNC Symbol;Acc:HGNC:18271]	K05746	ENAH, MENA
ENSP00000323065.1	GADD45G interacting protein 1 [Source:HGNC Symbol;Acc:HGNC:29996]		
ENSP00000310117.3	protein phosphatase 1 regulatory inhibitor subunit 14B [Source:HGNC Symbol;Acc:HGNC:9(	K17555	PPP1R14B
ENSP00000367024.4	N(alpha)-acetyltransferase 40, NatD catalytic subunit [Source:HGNC Symbol;Acc:HGNC:258	K20794	NAA40, NAT4
ENSP00000397580.2	erythrocyte membrane protein band 4.1 like 2 [Source:HGNC Symbol;Acc:HGNC:3379]		
ENSP00000411471.2	ATP binding cassette subfamily F member 3 [Source:HGNC Symbol;Acc:HGNC:72]	K06158	ABCF3
ENSP00000478763.1	signal regulatory protein alpha [Source:HGNC Symbol;Acc:HGNC:9662]	K06551	SIRPA_B1_G, CD172
ENSP00000362524.3	angiopoietin like 2 [Source:HGNC Symbol;Acc:HGNC:490]		
ENSP00000353654.5	collagen type IV alpha 2 chain [Source:HGNC Symbol;Acc:HGNC:2203]	K06237	COL4A
ENSP00000262238.4	YY1 transcription factor [Source:HGNC Symbol;Acc:HGNC:12856]	K09201	YY
ENSP00000263408.4	complement C9 [Source:HGNC Symbol;Acc:HGNC:1358]	K04000	C9
ENSP00000463188.1	tripartite motif containing 16 [Source:HGNC Symbol;Acc:HGNC:17241]	K12006	TRIM16
ENSP00000350509.4	androgen induced 1 [Source:HGNC Symbol;Acc:HGNC:21607]		
ENSP00000327589.1	glutamate dehydrogenase 2 [Source:HGNC Symbol;Acc:HGNC:4336]	K00261	GLUD1_2, gdhA
ENSP00000450607.1	timeless circadian regulator [Source:HGNC Symbol;Acc:HGNC:11813]	K03155	TIMELESS
ENSP00000342023.5	F-box protein 38 [Source:HGNC Symbol;Acc:HGNC:28844]	K10313	FBXO38, MOKA
ENSP00000297848.3	collagen type XIV alpha 1 chain [Source:HGNC Symbol;Acc:HGNC:2191]	K08133	COL14A
ENSP00000354722.2	eukaryotic translation initiation factor 1A Y-linked [Source:HGNC Symbol;Acc:HGNC:3252]	K03236	EIF1A
ENSP00000364000.3	collagen type V alpha 2 chain [Source:HGNC Symbol;Acc:HGNC:2210]	K19721	COL5AS
ENSP00000254810.3	H3 histone family member 3B [Source:HGNC Symbol;Acc:HGNC:4765]	K11253	H3
ENSP00000325421.4	presenilin associated rhomboid like [Source:HGNC Symbol;Acc:HGNC:18253]	K09650	PARL, PSARL, PCP1
ENSP00000353032.7	purinergic receptor P2X 4 [Source:HGNC Symbol;Acc:HGNC:8535]		
ENSP00000225665.7	solute carrier family 25 member 11 [Source:HGNC Symbol;Acc:HGNC:10981]	K15104	SLC25A11, OGC
ENSP00000385499.1	STON1-GTF2A1L readthrough [Source:HGNC Symbol;Acc:HGNC:30651]	K20067;	TFIIA1, GTF2A1, TOA1;STC
ENSP00000498597.1	TSPY like 1 [Source:HGNC Symbol;Acc:HGNC:12382]	K11284	TSPYL1
ENSP00000341779.5	protein phosphatase 1 catalytic subunit gamma [Source:HGNC Symbol;Acc:HGNC:9283]	K06269	PPP1C
ENSP00000391481.1	transketolase [Source:HGNC Symbol;Acc:HGNC:11834]	K00615	E2.2.1.1, tktA, tktB
ENSP00000487049.2	erythrocyte membrane protein band 4.1 like 1 [Source:HGNC Symbol;Acc:HGNC:3378]		

ENSP00000355110.3 SPARC related modular calcium binding 1 [Source:HGNC Symbol;Acc:HGNC:20318]		
ENSP00000311809.4 collagen type XIV alpha 1 chain [Source:HGNC Symbol;Acc:HGNC:2191]	K08133	COL14A
ENSP00000240285.5 retinol dehydrogenase 10 [Source:HGNC Symbol;Acc:HGNC:19975]	K11151	RDH10
ENSP00000378484.3 voltage dependent anion channel 1 [Source:HGNC Symbol;Acc:HGNC:12669]	K05862	VDAC1
ENSP00000400759.1 cytochrome c oxidase assembly factor 1 homolog [Source:HGNC Symbol;Acc:HGNC:21868]	K18173	COA1
ENSP00000452879.1 tropomyosin 1 [Source:HGNC Symbol;Acc:HGNC:12010]		
ENSP00000311713.3 oxidative stress responsive kinase 1 [Source:HGNC Symbol;Acc:HGNC:8508]	K08835	OXSR1, STK39
ENSP00000359393.3 high mobility group box 3 [Source:HGNC Symbol;Acc:HGNC:5004]	K11296	HMGB3
ENSP00000363458.4 low density lipoprotein receptor adaptor protein 1 [Source:HGNC Symbol;Acc:HGNC:18640]	K12474	LDLRAP1, ARH
ENSP00000276416.6 bridging integrator 3 [Source:HGNC Symbol;Acc:HGNC:1054]	K20120	BIN3
ENSP00000430420.1 annexin A6 [Source:HGNC Symbol;Acc:HGNC:544]		
ENSP00000360916.3 vav guanine nucleotide exchange factor 2 [Source:HGNC Symbol;Acc:HGNC:12658]	K05730	VAV
ENSP00000396308.2 dihydrofolate reductase [Source:HGNC Symbol;Acc:HGNC:2861]	K00287	folA
ENSP00000231461.4 ST8 alpha-N-acetyl-neuraminide alpha-2,8-sialyltransferase 4 [Source:HGNC Symbol;Acc:H	K06614	SIAT8D
ENSP00000359305.3 transmembrane p24 trafficking protein 5 [Source:HGNC Symbol;Acc:HGNC:24251]	K14825	TMED5, ERP2, ERP4
ENSP00000416959.2 transformer 2 beta homolog [Source:HGNC Symbol;Acc:HGNC:10781]	K12897	TRA2
ENSP00000363591.3 BCL2 antagonist/killer 1 [Source:HGNC Symbol;Acc:HGNC:949]	K14021	BAK, BAK1
ENSP00000497642.1 lipoprotein lipase [Source:HGNC Symbol;Acc:HGNC:6677]	K01059	LPL
ENSP00000346931.1 chromosome 6 open reading frame 120 [Source:HGNC Symbol;Acc:HGNC:21247]		
ENSP00000376919.2 LUC7 like 3 pre-mRNA splicing factor [Source:HGNC Symbol;Acc:HGNC:24309]		
ENSP00000238256.3 FKBP prolyl isomerase 15 [Source:HGNC Symbol;Acc:HGNC:23397]	K17478	FKBP15, WAFL
ENSP00000380159.3 FLYWCH family member 2 [Source:HGNC Symbol;Acc:HGNC:25178]		
ENSP00000361892.3 serine/threonine kinase 4 [Source:HGNC Symbol;Acc:HGNC:11408]	K04411	STK4, MST1
ENSP00000227525.3 transmembrane protein 109 [Source:HGNC Symbol;Acc:HGNC:28771]		
ENSP00000011473.2 synaptophysin like 1 [Source:HGNC Symbol;Acc:HGNC:11507]		
ENSP00000380413.2 ArfGAP with GTPase domain, ankyrin repeat and PH domain 3 [Source:HGNC Symbol;Acc:H	K12491	AGAP1_3
ENSP00000383898.3 solute carrier family 25 member 3 [Source:HGNC Symbol;Acc:HGNC:10989]		
ENSP00000296591.4 EGF like repeats and discoidin domains 3 [Source:HGNC Symbol;Acc:HGNC:3173]		
ENSP00000484580.1 protein phosphatase 1 regulatory inhibitor subunit 2 [Source:HGNC Symbol;Acc:HGNC:928	K16833	PPP1R2, IPP2
ENSP00000292566.3 alkB homolog 4, lysine demethylase [Source:HGNC Symbol;Acc:HGNC:21900]	K10766	ALKBH4
ENSP00000262094.4 RAB27B, member RAS oncogene family [Source:HGNC Symbol;Acc:HGNC:9767]	K07886	RAB27B
ENSP00000376276.2 serine and arginine rich splicing factor 2 [Source:HGNC Symbol;Acc:HGNC:10783]	K12891	SFRS2
ENSP00000384552.1 fibrinogen gamma chain [Source:HGNC Symbol;Acc:HGNC:3694]	K03905	FGG
ENSP00000361664.1 human immunodeficiency virus type I enhancer binding protein 3 [Source:HGNC Symbol;Ac	K09239	HIVEP
ENSP00000382717.3 WD repeat domain 19 [Source:HGNC Symbol;Acc:HGNC:18340]	K19671	WDR19, IFT144
ENSP00000302229.8 phospholipid phosphatase 1 [Source:HGNC Symbol;Acc:HGNC:9228]	K01080	PPAP2
ENSP00000341874.3 RIO kinase 3 [Source:HGNC Symbol;Acc:HGNC:11451]	K08872	RIOK3, SUDD
ENSP00000286713.2 stomatin [Source:HGNC Symbol;Acc:HGNC:3383]	K17286	STOM

ENSP00000429366.1	antioxidant 1 copper chaperone [Source:HGNC Symbol;Acc:HGNC:798]	K07213	ATOX1, ATX1, copZ, golB
ENSP00000385703.1	LBH regulator of WNT signaling pathway [Source:HGNC Symbol;Acc:HGNC:29532]		
ENSP00000389297.2	coiled-coil-helix-coiled-coil-helix domain containing 3 [Source:HGNC Symbol;Acc:HGNC:21906]		
ENSP00000363162.3	ATPase H+ transporting V1 subunit G1 [Source:HGNC Symbol;Acc:HGNC:864]	K02152	ATPeV1G, ATP6G
ENSP00000230050.3	ribosomal protein S12 [Source:HGNC Symbol;Acc:HGNC:10385]	K02951	RP-S12e, RPS12
ENSP00000360883.4	interferon induced protein with tetratricopeptide repeats 3 [Source:HGNC Symbol;Acc:HGNC:5411]		
ENSP00000435070.1	microtubule actin crosslinking factor 1 [Source:HGNC Symbol;Acc:HGNC:13664]	K19827	MACF1
ENSP00000497605.1	inositol 1,4,5-trisphosphate receptor type 1 [Source:HGNC Symbol;Acc:HGNC:6180]		
ENSP00000400717.2	G protein subunit alpha 13 [Source:HGNC Symbol;Acc:HGNC:4381]	K04639	GNA13
ENSP00000342962.3	serine incorporator 1 [Source:HGNC Symbol;Acc:HGNC:13464]		
ENSP00000455736.1	pyruvate kinase M1/2 [Source:HGNC Symbol;Acc:HGNC:9021]		
ENSP00000419782.1	cyclin dependent kinase 5 [Source:HGNC Symbol;Acc:HGNC:1774]	K02090	CDK5
ENSP00000496166.1	aldolase, fructose-bisphosphate A [Source:HGNC Symbol;Acc:HGNC:414]	K01623	ALDO
ENSP00000447447.1	keratin 74 [Source:HGNC Symbol;Acc:HGNC:28929]		
ENSP00000280258.4	serine protease 23 [Source:HGNC Symbol;Acc:HGNC:14370]	K09627	PRSS23
ENSP00000480987.1	minichromosome maintenance complex component 3 [Source:HGNC Symbol;Acc:HGNC:69	K02541	MCM3
ENSP00000373684.3	serine/threonine kinase 38 like [Source:HGNC Symbol;Acc:HGNC:17848]	K08790	STK38, NDR
ENSP00000419184.2	SET and MYND domain containing 3 [Source:HGNC Symbol;Acc:HGNC:15513]	K11426	SMYD
ENSP00000216259.7	phosphomannomutase 1 [Source:HGNC Symbol;Acc:HGNC:9114]	K17497	PMM
ENSP00000393887.2	alpha 2-HS glycoprotein [Source:HGNC Symbol;Acc:HGNC:349]		
ENSP00000311427.5	sorting nexin 33 [Source:HGNC Symbol;Acc:HGNC:28468]	K17923	SNX9_18_33
ENSP00000369849.4	mesenteric estrogen dependent adipogenesis [Source:HGNC Symbol;Acc:HGNC:25926]		
ENSP00000295897.4	albumin [Source:HGNC Symbol;Acc:HGNC:399]	K16141	ALB
ENSP00000428115.1	ribonucleotide reductase regulatory TP53 inducible subunit M2B [Source:HGNC Symbol;Acc:HGNC:17296]		
ENSP00000441282.1	ATPase H+ transporting V0 subunit d1 [Source:HGNC Symbol;Acc:HGNC:13724]		
ENSP00000378243.3	calcium/calmodulin dependent protein kinase II gamma [Source:HGNC Symbol;Acc:HGNC:1463]		
ENSP00000253792.2	ATP citrate lyase [Source:HGNC Symbol;Acc:HGNC:115]	K01648	ACLY
ENSP00000354511.6	catechol-O-methyltransferase [Source:HGNC Symbol;Acc:HGNC:2228]	K00545	COMT
ENSP00000368100.4	RAD50 double strand break repair protein [Source:HGNC Symbol;Acc:HGNC:9816]	K10866	RAD50
ENSP00000364694.3	asporin [Source:HGNC Symbol;Acc:HGNC:14872]	K08120	ASPN
ENSP00000262225.3	transmembrane p24 trafficking protein 2 [Source:HGNC Symbol;Acc:HGNC:16996]	K20347	TMED2, EMP24
ENSP00000360718.3	RAB3B, member RAS oncogene family [Source:HGNC Symbol;Acc:HGNC:9778]	K06108	RAB3B
ENSP00000367817.3	podocalyxin like [Source:HGNC Symbol;Acc:HGNC:9171]	K06817	PODXL
ENSP00000366007.1	nicotinamide riboside kinase 1 [Source:HGNC Symbol;Acc:HGNC:26057]	K10524	NRK1_2
ENSP00000268129.5	abhydrolase domain containing 2 [Source:HGNC Symbol;Acc:HGNC:18717]	K13697	ABHD2
ENSP00000301788.7	RNA polymerase II subunit G [Source:HGNC Symbol;Acc:HGNC:9194]	K03015	RPB7, POLR2G
ENSP00000330836.5	glutaredoxin 3 [Source:HGNC Symbol;Acc:HGNC:15987]		
ENSP00000472264.1	ubiquitin A-52 residue ribosomal protein fusion product 1 [Source:HGNC Symbol;Acc:HGNC:12458]		

ENSP00000368447.4	GPALPP motifs containing 1 [Source:HGNC Symbol;Acc:HGNC:20298]		
ENSP00000356530.3	ribonuclease L [Source:HGNC Symbol;Acc:HGNC:10050]	K01165	RNASEL
ENSP00000218099.2	coagulation factor IX [Source:HGNC Symbol;Acc:HGNC:3551]	K01321	F9
ENSP00000355968.4	solute carrier family 30 member 1 [Source:HGNC Symbol;Acc:HGNC:11012]	K14688	SLC30A1, ZNT1
ENSP00000345445.4	SAMM50 sorting and assembly machinery component [Source:HGNC Symbol;Acc:HGNC:24	K07277	SAM50, TOB55, bamA
ENSP00000368242.4	ribonuclease P/MRP subunit p25 like [Source:HGNC Symbol;Acc:HGNC:19909]	K14525	RPP25
ENSP00000362111.4	tetraspanin 6 [Source:HGNC Symbol;Acc:HGNC:11858]	K17295	TSPAN6
ENSP00000478274.1	survival of motor neuron 2, centromeric [Source:HGNC Symbol;Acc:HGNC:11118]		
ENSP00000347184.5	huntingtin [Source:HGNC Symbol;Acc:HGNC:4851]	K04533	HD
ENSP00000239449.4	protocadherin beta 14 [Source:HGNC Symbol;Acc:HGNC:8685]	K16494	PCDHB
ENSP00000302886.6	proliferation-associated 2G4 [Source:HGNC Symbol;Acc:HGNC:8550]		
ENSP00000326706.4	pleckstrin homology domain containing O2 [Source:HGNC Symbol;Acc:HGNC:30026]		
ENSP00000370394.3	RUN and FYVE domain containing 3 [Source:HGNC Symbol;Acc:HGNC:30285]		
ENSP00000404190.2	Rac GTPase activating protein 1 [Source:HGNC Symbol;Acc:HGNC:9804]	K16733	RACGAP1, Tum
ENSP00000220003.9	C-terminal Src kinase [Source:HGNC Symbol;Acc:HGNC:2444]	K05728	CSK
ENSP00000308227.4	high mobility group AT-hook 1 [Source:HGNC Symbol;Acc:HGNC:5010]	K09282	HMGA1
ENSP00000361508.3	phospholipid transfer protein [Source:HGNC Symbol;Acc:HGNC:9093]	K08761	PLTP
ENSP00000224073.1	endothelial differentiation related factor 1 [Source:HGNC Symbol;Acc:HGNC:3164]	K03627	MBF1
ENSP00000354610.4	amylase alpha 2B (pancreatic) [Source:HGNC Symbol;Acc:HGNC:478]	K01176	AMY, amyA, malS
ENSP00000440756.1	cytochrome c oxidase assembly factor 4 homolog [Source:HGNC Symbol;Acc:HGNC:24604]	K18177	COA4
ENSP00000450758.1	G protein subunit gamma 2 [Source:HGNC Symbol;Acc:HGNC:4404]	K07826	GNG2
ENSP00000222968.4	PDGFA associated protein 1 [Source:HGNC Symbol;Acc:HGNC:14634]		
ENSP00000360269.2	tumor associated calcium signal transducer 2 [Source:HGNC Symbol;Acc:HGNC:11530]	K17288	TACSTD2
ENSP00000382250.2	mitochondrial ribosomal protein S6 [Source:HGNC Symbol;Acc:HGNC:14051]	K02990	RP-S6, MRPS6, rpsF
ENSP00000494918.1	phosphatase and tensin homolog [Source:HGNC Symbol;Acc:HGNC:9588]		
ENSP00000254816.1	tripartite motif containing 47 [Source:HGNC Symbol;Acc:HGNC:19020]	K12023	TRIM47
ENSP00000424183.1	late endosomal/lysosomal adaptor, MAPK and MTOR activator 3 [Source:HGNC Symbol;Acc	K04370	LAMTOR3, MP1, MAP2K1I
ENSP00000399812.2	collagen type XII alpha 1 chain [Source:HGNC Symbol;Acc:HGNC:2188]	K08132	COL12A
ENSP00000421725.1	GC vitamin D binding protein [Source:HGNC Symbol;Acc:HGNC:4187]	K12258	GC
ENSP00000285379.4	carbonic anhydrase 2 [Source:HGNC Symbol;Acc:HGNC:1373]	K18245	CA2
ENSP00000295666.4	insulin like growth factor binding protein 7 [Source:HGNC Symbol;Acc:HGNC:5476]		
ENSP00000318177.9	far upstream element binding protein 3 [Source:HGNC Symbol;Acc:HGNC:4005]	K13210	FUBP
ENSP00000260363.4	kinesin family member 23 [Source:HGNC Symbol;Acc:HGNC:6392]	K17387	KIF23
ENSP00000476687.1	torsin 1A interacting protein 1 [Source:HGNC Symbol;Acc:HGNC:29456]		
ENSP00000272746.5	WAS/WASL interacting protein family member 1 [Source:HGNC Symbol;Acc:HGNC:12736]	K19475	WIPF
ENSP00000466897.1	cold inducible RNA binding protein [Source:HGNC Symbol;Acc:HGNC:1982]		
ENSP00000325748.4	serine/threonine kinase 25 [Source:HGNC Symbol;Acc:HGNC:11404]	K08838	STK24_25_MST4
ENSP00000385746.2	spermine synthase [Source:HGNC Symbol;Acc:HGNC:11123]	K00802	SMS

ENSP00000461988.1	mitogen-activated protein kinase kinase kinase 3 [Source:HGNC Symbol;Acc:HGNC:6855]		
ENSP00000348278.4	serine/threonine kinase 39 [Source:HGNC Symbol;Acc:HGNC:17717]	K08835	OXSR1, STK39
ENSP00000262428.4	coactosin like F-actin binding protein 1 [Source:HGNC Symbol;Acc:HGNC:18304]		
ENSP00000310670.4	cell cycle and apoptosis regulator 2 [Source:HGNC Symbol;Acc:HGNC:23360]		
ENSP00000356902.1	UDP-N-acetylglucosamine pyrophosphorylase 1 [Source:HGNC Symbol;Acc:HGNC:12457]	K00972	UAP1
ENSP00000261883.4	cartilage intermediate layer protein [Source:HGNC Symbol;Acc:HGNC:1980]		
ENSP00000420588.1	transcription factor A, mitochondrial [Source:HGNC Symbol;Acc:HGNC:11741]	K11830	TFAM, MTTFA
ENSP00000181796.2	family with sequence similarity 107 member B [Source:HGNC Symbol;Acc:HGNC:23726]		
ENSP00000265866.7	heterogeneous nuclear ribonucleoprotein H3 [Source:HGNC Symbol;Acc:HGNC:5043]	K12898	HNRNPF_H
ENSP00000441927.1	sestrin 3 [Source:HGNC Symbol;Acc:HGNC:23060]	K10141	SESN1_3
ENSP00000217195.8	chromosome 20 open reading frame 27 [Source:HGNC Symbol;Acc:HGNC:15873]		
ENSP00000352696.1	fibronectin 1 [Source:HGNC Symbol;Acc:HGNC:3778]	K05717	FN1
ENSP00000245932.5	vasodilator stimulated phosphoprotein [Source:HGNC Symbol;Acc:HGNC:12652]	K06274	VASP
ENSP00000364886.2	Rho family GTPase 3 [Source:HGNC Symbol;Acc:HGNC:671]	K07859	RND3
ENSP00000425286.1	transmembrane protein 26 [Source:HGNC Symbol;Acc:HGNC:28550]		
ENSP00000411532.1	DNA topoisomerase II alpha [Source:HGNC Symbol;Acc:HGNC:11989]	K03164	TOP2
ENSP00000357697.4	S100 calcium binding protein A2 [Source:HGNC Symbol;Acc:HGNC:10492]		
ENSP00000334052.4	legumain [Source:HGNC Symbol;Acc:HGNC:9472]	K01369	LGMN
ENSP00000483879.1	XK related 5 [Source:HGNC Symbol;Acc:HGNC:20782]		
ENSP00000304408.3	collagen type III alpha 1 chain [Source:HGNC Symbol;Acc:HGNC:2201]	K19720	COL3A
ENSP00000337773.2	N-ribosyldihydronicotinamide:quinone reductase 2 [Source:HGNC Symbol;Acc:HGNC:7856]	K08071	NQO2
ENSP00000305138.4	MAPK regulated corepressor interacting protein 2 [Source:HGNC Symbol;Acc:HGNC:14142]		
ENSP00000498855.1	calcyphosine [Source:HGNC Symbol;Acc:HGNC:1487]		
ENSP00000305647.4	vesicle associated membrane protein 5 [Source:HGNC Symbol;Acc:HGNC:12646]	K08514	VAMP5
ENSP00000436864.1	CUGBP Elav-like family member 1 [Source:HGNC Symbol;Acc:HGNC:2549]	K13207	CUGBP, BRUNOL, CELF
ENSP00000346564.4	tumor protein p53 inducible protein 11 [Source:HGNC Symbol;Acc:HGNC:16842]		
ENSP00000468927.1	signal transducing adaptor family member 2 [Source:HGNC Symbol;Acc:HGNC:30430]		
ENSP00000380880.2	solute carrier family 66 member 2 [Source:HGNC Symbol;Acc:HGNC:26188]		
ENSP00000340989.4	stratifin [Source:HGNC Symbol;Acc:HGNC:10773]	K06644	SFN
ENSP00000397908.2	lipin 1 [Source:HGNC Symbol;Acc:HGNC:13345]		
ENSP00000000412.3	mannose-6-phosphate receptor, cation dependent [Source:HGNC Symbol;Acc:HGNC:6752]	K10089	M6PR
ENSP00000280551.6	SEC24 homolog D, COPII coat complex component [Source:HGNC Symbol;Acc:HGNC:1070	K14007	SEC24
ENSP00000225831.4	C-C motif chemokine ligand 2 [Source:HGNC Symbol;Acc:HGNC:10618]	K14624	CCL2, MCP1
ENSP00000478171.1	ubiquitin like with PHD and ring finger domains 1 [Source:HGNC Symbol;Acc:HGNC:12556]	K10638	UHRF1, NP95
ENSP00000322885.5	deltex E3 ubiquitin ligase 2 [Source:HGNC Symbol;Acc:HGNC:15973]	K06058	DTX
ENSP00000397166.1	LIM domain containing preferred translocation partner in lipoma [Source:HGNC Symbol;Acc	K16676	LPP
ENSP00000350757.3	glia maturation factor beta [Source:HGNC Symbol;Acc:HGNC:4373]		
ENSP00000377617.2	methylenetetrahydrofolate dehydrogenase (NADP+ dependent) 2, methenyltetrahydrofolat	K13403	MTHFD2

ENSP00000408827.2	MAPK regulated corepressor interacting protein 1 [Source:HGNC Symbol;Acc:HGNC:28007]		
ENSP00000404381.2	DnaJ heat shock protein family (Hsp40) member B14 [Source:HGNC Symbol;Acc:HGNC:2588	K09520	DNAJB14
ENSP00000348093.5	heterogeneous nuclear ribonucleoprotein A/B [Source:HGNC Symbol;Acc:HGNC:5034]		
ENSP00000345848.4	acidic nuclear phosphoprotein 32 family member B [Source:HGNC Symbol;Acc:HGNC:16677	K18647	ANP32B
ENSP00000484569.1	proteasome activator subunit 2 [Source:HGNC Symbol;Acc:HGNC:9569]		
ENSP00000263036.3	optineurin [Source:HGNC Symbol;Acc:HGNC:17142]	K19946	OPTN, FIP2
ENSP00000265605.2	aldehyde dehydrogenase 8 family member A1 [Source:HGNC Symbol;Acc:HGNC:15471]		
ENSP00000216117.8	heme oxygenase 1 [Source:HGNC Symbol;Acc:HGNC:5013]	K00510	HMOX1
ENSP00000297268.6	collagen type I alpha 2 chain [Source:HGNC Symbol;Acc:HGNC:2198]	K06236	COL1A
ENSP00000353284.4	DEAD-box helicase 3 Y-linked [Source:HGNC Symbol;Acc:HGNC:2699]	K17642	DDX3Y
ENSP00000265748.2	anillin actin binding protein [Source:HGNC Symbol;Acc:HGNC:14082]	K18621	ANLN
ENSP00000386043.2	latent transforming growth factor beta binding protein 1 [Source:HGNC Symbol;Acc:HGNC:[illegible]	K19559	LTBP1
ENSP00000244573.3	histone cluster 1 H1 family member a [Source:HGNC Symbol;Acc:HGNC:4715]	K11275	H1_5
ENSP00000422607.1	acyl-CoA synthetase long chain family member 1 [Source:HGNC Symbol;Acc:HGNC:3569]	K01897	ACSL, fadD
ENSP00000362466.1	secreted phosphoprotein 2 [Source:HGNC Symbol;Acc:HGNC:11256]		
ENSP00000360305.3	PDZ and LIM domain 1 [Source:HGNC Symbol;Acc:HGNC:2067]		
ENSP00000316176.2	ubiquitin conjugating enzyme E2 N [Source:HGNC Symbol;Acc:HGNC:12492]	K10580	UBE2N, BLU, UBC13
ENSP00000360882.3	collagen type V alpha 1 chain [Source:HGNC Symbol;Acc:HGNC:2209]	K19721	COL5AS
ENSP00000364293.4	TSR2 ribosome maturation factor [Source:HGNC Symbol;Acc:HGNC:25455]	K14800	TSR2
ENSP00000476948.1	RNA polymerase II associated protein 2 [Source:HGNC Symbol;Acc:HGNC:25791]	K20827	RPAP2
ENSP00000306614.3	peptidylprolyl isomerase H [Source:HGNC Symbol;Acc:HGNC:14651]	K09567	PPIH, CYPH
ENSP00000471388.1	ribosomal protein S5 [Source:HGNC Symbol;Acc:HGNC:10426]		
ENSP00000306864.3	vasorin [Source:HGNC Symbol;Acc:HGNC:18517]		
ENSP00000404464.2	collagen and calcium binding EGF domains 1 [Source:HGNC Symbol;Acc:HGNC:29426]	K19638	CCBE1
ENSP00000432216.1	serum amyloid A like 1 [Source:HGNC Symbol;Acc:HGNC:25158]		
ENSP00000353846.3	doublecortin like kinase 1 [Source:HGNC Symbol;Acc:HGNC:2700]	K08805	DCLK1_2
ENSP00000372206.1	EEF1A lysine methyltransferase 1 [Source:HGNC Symbol;Acc:HGNC:27351]		
ENSP00000244534.5	histone cluster 1 H1 family member d [Source:HGNC Symbol;Acc:HGNC:4717]	K11275	H1_5
ENSP00000402608.2	carbamoyl-phosphate synthase 1 [Source:HGNC Symbol;Acc:HGNC:2323]	K01948	CPS1
ENSP00000499734.1	novel protein	K12893	SFRS4_5_6
ENSP00000356579.3	centrosomal protein 350 [Source:HGNC Symbol;Acc:HGNC:24238]	K16768	CEP350
ENSP00000367794.3	sushi repeat containing protein X-linked [Source:HGNC Symbol;Acc:HGNC:11309]		
ENSP00000316809.7	phosphatidylinositol transfer protein alpha [Source:HGNC Symbol;Acc:HGNC:9001]		
ENSP00000340454.5	Rap1 GTPase-GDP dissociation stimulator 1 [Source:HGNC Symbol;Acc:HGNC:9859]		
ENSP00000356860.1	microsomal glutathione S-transferase 3 [Source:HGNC Symbol;Acc:HGNC:7064]	K00799	GST, gst
ENSP00000497283.1	gap junction protein alpha 1 [Source:HGNC Symbol;Acc:HGNC:4274]	K07372	GJA1, CX43
ENSP00000428489.1	PDZ binding kinase [Source:HGNC Symbol;Acc:HGNC:18282]		
ENSP00000315130.1	clusterin [Source:HGNC Symbol;Acc:HGNC:2095]	K17252	CLU

ENSP00000265857.3	guided entry of tail-anchored proteins factor 4 [Source:HGNC Symbol;Acc:HGNC:21690]		
ENSP00000370527.4	regulator of calcineurin 1 [Source:HGNC Symbol;Acc:HGNC:3040]	K17901	RCAN1, MCIP1
ENSP00000315644.1	thymidylate synthetase [Source:HGNC Symbol;Acc:HGNC:12441]	K00560	thyA, TYMS
ENSP00000393183.2	DDB1 and CUL4 associated factor 1 [Source:HGNC Symbol;Acc:HGNC:30911]	K11789	VPRBP, DCAF1
ENSP00000263640.3	activin A receptor type 1 [Source:HGNC Symbol;Acc:HGNC:171]	K04675	ACVR1, ALK2
ENSP00000370114.4	PC4 and SFRS1 interacting protein 1 [Source:HGNC Symbol;Acc:HGNC:9527]		
ENSP00000417824.1	inter-alpha-trypsin inhibitor heavy chain 4 [Source:HGNC Symbol;Acc:HGNC:6169]		
ENSP00000387654.2	prenylcysteine oxidase 1 [Source:HGNC Symbol;Acc:HGNC:20588]	K05906	PCYOX1, FCLY
ENSP00000420678.2	TBC1 domain family member 15 [Source:HGNC Symbol;Acc:HGNC:25694]		
ENSP00000248553.6	heat shock protein family B (small) member 1 [Source:HGNC Symbol;Acc:HGNC:5246]	K04455	HSPB1
ENSP00000354451.2	IQ motif containing GTPase activating protein 3 [Source:HGNC Symbol;Acc:HGNC:20669]	K05767	IQGAP2_3
ENSP00000368664.3	regulator of cell cycle [Source:HGNC Symbol;Acc:HGNC:20369]		
ENSP00000484472.1	epiplakin 1 [Source:HGNC Symbol;Acc:HGNC:15577]		
ENSP00000362820.5	serine and arginine rich splicing factor 3 [Source:HGNC Symbol;Acc:HGNC:10785]	K12892	SFRS3
ENSP00000216500.5	dehydrogenase/reductase 7 [Source:HGNC Symbol;Acc:HGNC:21524]	K11165	DHRS7
ENSP00000324248.3	proenkephalin [Source:HGNC Symbol;Acc:HGNC:8831]	K18832	PENK
ENSP00000289371.5	eukaryotic translation initiation factor 5B [Source:HGNC Symbol;Acc:HGNC:30793]	K03243	EIF5B
ENSP00000334329.6	unc-5 netrin receptor B [Source:HGNC Symbol;Acc:HGNC:12568]	K07521	UNC5
ENSP00000418753.1	serine/threonine kinase 26 [Source:HGNC Symbol;Acc:HGNC:18174]		
ENSP00000487640.1	tissue specific transplantation antigen P35B [Source:HGNC Symbol;Acc:HGNC:12390]	K02377	TSTA3, fcl
ENSP00000470972.1	ribosomal protein S19 [Source:HGNC Symbol;Acc:HGNC:10402]	K02966	RP-S19e, RPS19
ENSP00000361635.1	voltage dependent anion channel 2 [Source:HGNC Symbol;Acc:HGNC:12672]	K15040	VDAC2
ENSP00000264156.2	minichromosome maintenance complex component 6 [Source:HGNC Symbol;Acc:HGNC:69	K02542	MCM6
ENSP00000341911.4	paralemmin [Source:HGNC Symbol;Acc:HGNC:8594]	K16519	AKAP2
ENSP00000318176.4	protein activator of interferon induced protein kinase EIF2AK2 [Source:HGNC Symbol;Acc:HGNC:9438]		
ENSP00000498441.1	fibrinogen alpha chain [Source:HGNC Symbol;Acc:HGNC:3661]	K03903	FGA
ENSP00000481886.1	progesterone receptor membrane component 2 [Source:HGNC Symbol;Acc:HGNC:16089]	K17278	PGRMC1_2
ENSP00000248598.5	fibrinogen like 2 [Source:HGNC Symbol;Acc:HGNC:3696]		
ENSP00000386994.1	coiled-coil-helix-coiled-coil-helix domain containing 5 [Source:HGNC Symbol;Acc:HGNC:17840]		
ENSP00000409909.1	Sad1 and UNC84 domain containing 1 [Source:HGNC Symbol;Acc:HGNC:18587]	K19347	SUN1_2
ENSP00000392028.1	chromodomain helicase DNA binding protein 7 [Source:HGNC Symbol;Acc:HGNC:20626]	K14437	CHD7
ENSP00000424571.1	fibrillin 2 [Source:HGNC Symbol;Acc:HGNC:3604]		
ENSP00000388942.1	secernin 1 [Source:HGNC Symbol;Acc:HGNC:22192]	K14358	SCRN
ENSP00000478104.1	vacuolar protein sorting 13 homolog D [Source:HGNC Symbol;Acc:HGNC:23595]	K19527	VPS13D
ENSP00000440222.1	FMC1-LUC7L2 readthrough [Source:HGNC Symbol;Acc:HGNC:44671]	K13212	LUC7L2
ENSP00000377793.3	protein regulator of cytokinesis 1 [Source:HGNC Symbol;Acc:HGNC:9341]	K16732	PRC1
ENSP00000240185.3	TAR DNA binding protein [Source:HGNC Symbol;Acc:HGNC:11571]		
ENSP00000270722.5	PR/SET domain 16 [Source:HGNC Symbol;Acc:HGNC:14000]		

ENSP00000356382.1	coagulation factor XIII B chain [Source:HGNC Symbol;Acc:HGNC:3534]	K03906	F13B
ENSP00000219789.6	CDP-diacylglycerol--inositol 3-phosphatidyltransferase [Source:HGNC Symbol;Acc:HGNC:1	K00999	CDIPT
ENSP00000261313.2	phosphatidylethanolamine binding protein 1 [Source:HGNC Symbol;Acc:HGNC:8630]		
ENSP00000313327.6	heterogeneous nuclear ribonucleoprotein D [Source:HGNC Symbol;Acc:HGNC:5036]		
ENSP00000444711.1	ring finger protein 24 [Source:HGNC Symbol;Acc:HGNC:13779]		
ENSP00000495440.1	tropomyosin 2 [Source:HGNC Symbol;Acc:HGNC:12011]		
ENSP00000309474.6	proteasome 26S subunit, non-ATPase 1 [Source:HGNC Symbol;Acc:HGNC:9554]	K03032	PSMD1, RPN2
ENSP00000357721.1	S100 calcium binding protein A8 [Source:HGNC Symbol;Acc:HGNC:10498]	K21127	S100A8
ENSP00000342710.3	keratin 77 [Source:HGNC Symbol;Acc:HGNC:20411]	K07605	KRT2
ENSP00000361883.3	cyclase associated actin cytoskeleton regulatory protein 1 [Source:HGNC Symbol;Acc:HGNC	K17261	CAP1_2, SRV2
ENSP00000403663.1	adaptor related protein complex 4 subunit mu 1 [Source:HGNC Symbol;Acc:HGNC:574]	K12402	AP4M1
ENSP00000360170.3	angiopoietin like 3 [Source:HGNC Symbol;Acc:HGNC:491]		
ENSP00000265087.4	stanniocalcin 2 [Source:HGNC Symbol;Acc:HGNC:11374]		
ENSP00000225576.3	trans-golgi network vesicle protein 23 homolog C [Source:HGNC Symbol;Acc:HGNC:30453]		
ENSP00000261226.4	transmembrane and coiled-coil domain family 3 [Source:HGNC Symbol;Acc:HGNC:29199]		
ENSP00000280527.2	cysteine rich transmembrane BMP regulator 1 [Source:HGNC Symbol;Acc:HGNC:2359]		
ENSP00000393312.3	fibroblast growth factor receptor 1 [Source:HGNC Symbol;Acc:HGNC:3688]	K04362	FGFR1, CD331
ENSP00000465734.1	solute carrier family 16 member 2 [Source:HGNC Symbol;Acc:HGNC:10923]	K08231	SLC16A2
ENSP00000287020.4	growth differentiation factor 6 [Source:HGNC Symbol;Acc:HGNC:4221]	K20012	GDF6
ENSP00000441875.1	prohibitin 2 [Source:HGNC Symbol;Acc:HGNC:30306]	K17081	PHB2
ENSP00000352798.4	collagen type XVIII alpha 1 chain [Source:HGNC Symbol;Acc:HGNC:2195]	K06823	COL18A
ENSP00000467630.1	coiled-coil domain containing 43 [Source:HGNC Symbol;Acc:HGNC:26472]		
ENSP00000389872.2	oxidoreductase NAD binding domain containing 1 [Source:HGNC Symbol;Acc:HGNC:25128]		
ENSP00000223061.5	procollagen C-endopeptidase enhancer [Source:HGNC Symbol;Acc:HGNC:8738]		
ENSP00000247026.5	nuclear speckle splicing regulatory protein 1 [Source:HGNC Symbol;Acc:HGNC:25305]	K13206	CCDC55
ENSP00000484616.1	cell proliferation regulating inhibitor of protein phosphatase 2A [Source:HGNC Symbol;Acc:HGNC:29302]		
ENSP00000223208.4	centrosomal protein 41 [Source:HGNC Symbol;Acc:HGNC:12370]	K16455	CEP41, TSGA14
ENSP00000357205.3	cellular retinoic acid binding protein 2 [Source:HGNC Symbol;Acc:HGNC:2339]	K17289	CRABP2
ENSP00000339340.3	potassium channel tetramerization domain containing 21 [Source:HGNC Symbol;Acc:HGNC:27452]		
ENSP00000362041.4	DnaJ heat shock protein family (Hsp40) member C9 [Source:HGNC Symbol;Acc:HGNC:1912[illegible]	K09529	DNAJC9
ENSP00000479794.1	histone cluster 1 H4 family member k [Source:HGNC Symbol;Acc:HGNC:4784]	K11254	H4
ENSP00000310263.3	TNF receptor superfamily member 10d [Source:HGNC Symbol;Acc:HGNC:11907]	K04722	TNFRSF10, TRAILR, CD261[illegible]
ENSP00000460236.1	spondin 1 [Source:HGNC Symbol;Acc:HGNC:11252]		
ENSP00000463042.1	serine and arginine rich splicing factor 1 [Source:HGNC Symbol;Acc:HGNC:10780]	K12890	SFRS1, ASF, SF2
ENSP00000410137.2	neurofilament medium [Source:HGNC Symbol;Acc:HGNC:7734]		
ENSP00000368438.5	proliferating cell nuclear antigen [Source:HGNC Symbol;Acc:HGNC:8729]	K04802	PCNA
ENSP00000355896.4	transforming growth factor beta 2 [Source:HGNC Symbol;Acc:HGNC:11768]		
ENSP00000424063.1	calnexin [Source:HGNC Symbol;Acc:HGNC:1473]	K08054	CANX

ENSP00000307863.3	U2 small nuclear RNA auxiliary factor 2 [Source:HGNC Symbol;Acc:HGNC:23156]	K12837	U2AF2
ENSP00000320447.6	nuclear receptor subfamily 2 group C member 2 [Source:HGNC Symbol;Acc:HGNC:7972]		
ENSP00000386794.2	Rho guanine nucleotide exchange factor 4 [Source:HGNC Symbol;Acc:HGNC:684]		
ENSP00000306003.4	ATP synthase membrane subunit e [Source:HGNC Symbol;Acc:HGNC:846]	K02129	ATPeF0E, ATP5I
ENSP00000386541.3	proteasome 26S subunit, non-ATPase 14 [Source:HGNC Symbol;Acc:HGNC:16889]	K03030	PSMD14, RPN11, POH1
ENSP00000301281.5	UBX domain protein 6 [Source:HGNC Symbol;Acc:HGNC:14928]	K14011	UBXN6, UBXD1
ENSP00000367446.2	exostosin glycosyltransferase 1 [Source:HGNC Symbol;Acc:HGNC:3512]	K02366	EXT1
ENSP00000228506.3	malectin [Source:HGNC Symbol;Acc:HGNC:28973]		
ENSP00000324527.5	myosin ID [Source:HGNC Symbol;Acc:HGNC:7598]	K10356	MYO1
ENSP00000300249.4	microtubule associated protein RP/EB family member 2 [Source:HGNC Symbol;Acc:HGNC:68	K10436	MAPRE
ENSP00000472266.1	mitochondrial ribosomal protein L34 [Source:HGNC Symbol;Acc:HGNC:14488]	K02914	RP-L34, MRPL34, rpmH
ENSP00000301761.2	succinate dehydrogenase complex assembly factor 2 [Source:HGNC Symbol;Acc:HGNC:2603	K18168	SDHAF2, SDH5
ENSP00000309163.3	Ras converting CAAX endopeptidase 1 [Source:HGNC Symbol;Acc:HGNC:13721]	K08658	RCE1, FACE2
ENSP00000320168.6	nucleobindin 2 [Source:HGNC Symbol;Acc:HGNC:8044]	K20371	NUCB
ENSP00000377969.3	general transcription factor IIF subunit 1 [Source:HGNC Symbol;Acc:HGNC:4652]	K03138	TFIIF1, GTF2F1, TFG1
ENSP00000462521.1	thioredoxin interacting protein [Source:HGNC Symbol;Acc:HGNC:16952]	K20910	TXNIP
ENSP00000315476.4	exosome component 4 [Source:HGNC Symbol;Acc:HGNC:18189]	K11600	RRP41, EXOSC4, SKI6
ENSP00000496485.1	ribosomal protein S29 [Source:HGNC Symbol;Acc:HGNC:10419]	K02980	RP-S29e, RPS29
ENSP00000237281.3	F-box protein 30 [Source:HGNC Symbol;Acc:HGNC:15600]	K10307	FBXO30
ENSP00000230085.8	sorting nexin 3 [Source:HGNC Symbol;Acc:HGNC:11174]	K17918	SNX3_12
ENSP00000364140.3	collagen type XV alpha 1 chain [Source:HGNC Symbol;Acc:HGNC:2192]	K08135	COL15A
ENSP00000298159.6	cofilin 2 [Source:HGNC Symbol;Acc:HGNC:1875]	K05765	CFL
ENSP00000405405.2	pleckstrin homology like domain family B member 2 [Source:HGNC Symbol;Acc:HGNC:29573]		
ENSP00000259526.3	cellular communication network factor 3 [Source:HGNC Symbol;Acc:HGNC:7885]		
ENSP00000365601.4	IDNK gluconokinase [Source:HGNC Symbol;Acc:HGNC:31367]	K00851	E2.7.1.12, gntK, idnK
ENSP00000408283.2	profilin 2 [Source:HGNC Symbol;Acc:HGNC:8882]	K05759	PFN
ENSP00000266671.5	pleckstrin homology like domain family A member 1 [Source:HGNC Symbol;Acc:HGNC:8933]		
ENSP00000312143.9	tensin 3 [Source:HGNC Symbol;Acc:HGNC:21616]	K18080	TNS
ENSP00000290705.8	metallothionein 1A [Source:HGNC Symbol;Acc:HGNC:7393]	K14739	MT1_2
ENSP00000280154.7	programmed cell death 4 [Source:HGNC Symbol;Acc:HGNC:8763]	K16865	PDCD4
ENSP00000229812.7	serine/threonine kinase 38 [Source:HGNC Symbol;Acc:HGNC:17847]	K08790	STK38, NDR
ENSP00000362702.3	NIMA related kinase 6 [Source:HGNC Symbol;Acc:HGNC:7749]	K20875	NEK6
ENSP00000364469.1	apolipoprotein A1 [Source:HGNC Symbol;Acc:HGNC:600]	K08757	APOA1
ENSP00000317224.5	sterile alpha motif domain containing 4B [Source:HGNC Symbol;Acc:HGNC:25492]		
ENSP00000481360.1	collagen type V alpha 1 chain [Source:HGNC Symbol;Acc:HGNC:2209]	K19721	COL5AS
ENSP00000237530.6	ribophorin II [Source:HGNC Symbol;Acc:HGNC:10382]	K12667	SWP1, RPN2
ENSP00000388806.3	eukaryotic translation initiation factor 4B [Source:HGNC Symbol;Acc:HGNC:3285]	K03258	EIF4B
ENSP00000356607.3	Ral GEF with PH domain and SH3 binding motif 2 [Source:HGNC Symbol;Acc:HGNC:30279]		

ENSP00000301012.3	mevalonate diphosphate decarboxylase [Source:HGNC Symbol;Acc:HGNC:7529]	K01597	MVD, mvaD
ENSP00000500517.1	aldehyde dehydrogenase 3 family member A2 [Source:HGNC Symbol;Acc:HGNC:403]	K00128	ALDH
ENSP00000385060.3	apolipoprotein B mRNA editing enzyme catalytic subunit 3B [Source:HGNC Symbol;Acc:HGI	K18750	APOBEC3
ENSP00000367880.3	phosphatidylinositol glycan anchor biosynthesis class O [Source:HGNC Symbol;Acc:HGNC:2	K05288	PIGO
ENSP00000455282.1	FUS RNA binding protein [Source:HGNC Symbol;Acc:HGNC:4010]	K13098	TLS, FUS
ENSP00000431696.1	cofilin 1 [Source:HGNC Symbol;Acc:HGNC:1874]	K05765	CFL
ENSP00000385609.1	pleckstrin homology domain containing B2 [Source:HGNC Symbol;Acc:HGNC:19236]		
ENSP00000344458.4	protein Z, vitamin K dependent plasma glycoprotein [Source:HGNC Symbol;Acc:HGNC:9460]		
ENSP00000328336.4	cell cycle exit and neuronal differentiation 1 [Source:HGNC Symbol;Acc:HGNC:24153]		
ENSP00000363524.3	kinesin family member 4A [Source:HGNC Symbol;Acc:HGNC:13339]	K10395	KIF4_21_27
ENSP00000484398.1	smoothelin [Source:HGNC Symbol;Acc:HGNC:11126]		
ENSP00000386608.1	small nuclear ribonucleoprotein U4/U6.U5 subunit 27 [Source:HGNC Symbol;Acc:HGNC:302	K12846	SNRNP27
ENSP00000216286.4	nidogen 2 [Source:HGNC Symbol;Acc:HGNC:13389]	K06826	NID
ENSP00000448762.1	poly(rC) binding protein 2 [Source:HGNC Symbol;Acc:HGNC:8648]	K13162	PCBP2
ENSP00000260818.6	DnaJ heat shock protein family (Hsp40) member C13 [Source:HGNC Symbol;Acc:HGNC:303	K09533	DNAJC13
ENSP00000206451.6	proteasome activator subunit 1 [Source:HGNC Symbol;Acc:HGNC:9568]	K06696	PSME1
ENSP00000257192.4	desmoglein 1 [Source:HGNC Symbol;Acc:HGNC:3048]	K07596	DSG1
ENSP00000293872.8	LUC7 like [Source:HGNC Symbol;Acc:HGNC:6723]		
ENSP00000356875.3	NUF2 component of NDC80 kinetochore complex [Source:HGNC Symbol;Acc:HGNC:14621]	K11548	NUF2, CDCA1
ENSP00000378356.3	kinesin family member 20A [Source:HGNC Symbol;Acc:HGNC:9787]	K10402	KIF20
ENSP00000465075.1	SPC24 component of NDC80 kinetochore complex [Source:HGNC Symbol;Acc:HGNC:26913	K11549	SPBC24, SPC24
ENSP00000422078.1	SUB1 regulator of transcription [Source:HGNC Symbol;Acc:HGNC:19985]		
ENSP00000310309.7	actin binding LIM protein family member 3 [Source:HGNC Symbol;Acc:HGNC:29132]	K07520	ABLIM
ENSP00000268150.8	milk fat globule-EGF factor 8 protein [Source:HGNC Symbol;Acc:HGNC:7036]	K17253	MFGE8
ENSP00000262545.2	proprotein convertase subtilisin/kexin type 2 [Source:HGNC Symbol;Acc:HGNC:8744]	K01360	PCSK2
ENSP00000314655.4	TSC22 domain family member 3 [Source:HGNC Symbol;Acc:HGNC:3051]		
ENSP00000466363.1	phosphatidylinositol-4-phosphate 5-kinase type 1 gamma [Source:HGNC Symbol;Acc:HGN	K00889	PIP5K
ENSP00000379513.3	microsomal glutathione S-transferase 1 [Source:HGNC Symbol;Acc:HGNC:7061]	K00799	GST, gst
ENSP00000363973.3	alkaline phosphatase, biomineralization associated [Source:HGNC Symbol;Acc:HGNC:438]	K01077	E3.1.3.1, phoA, phoB
ENSP00000387059.3	Jupiter microtubule associated homolog 1 [Source:HGNC Symbol;Acc:HGNC:14569]		
ENSP00000323929.7	alpha-2-macroglobulin [Source:HGNC Symbol;Acc:HGNC:7]	K03910	A2M
ENSP00000358045.4	extracellular matrix protein 1 [Source:HGNC Symbol;Acc:HGNC:3153]		
ENSP00000300738.5	ribonucleotide reductase catalytic subunit M1 [Source:HGNC Symbol;Acc:HGNC:10451]	K10807	RRM1
ENSP00000344259.4	ubiquitin conjugating enzyme E2 L3 [Source:HGNC Symbol;Acc:HGNC:12488]	K04552	UBE2L3, UBCH7
ENSP00000409382.2	calcium release activated channel regulator 2A [Source:HGNC Symbol;Acc:HGNC:28657]	K17199	RASEF, RAB45
ENSP00000423427.2	DEK proto-oncogene [Source:HGNC Symbol;Acc:HGNC:2768]	K17046	DEK
ENSP00000420608.1	ER membrane protein complex subunit 1 [Source:HGNC Symbol;Acc:HGNC:28957]		
ENSP00000371493.4	serpin family F member 2 [Source:HGNC Symbol;Acc:HGNC:9075]	K03983	SERPINF2, AAP

ENSP00000280612.5 solute carrier family 7 member 11 [Source:HGNC Symbol;Acc:HGNC:11059]	K13869	SLC7A11
ENSP00000266732.4 thymopoietin [Source:HGNC Symbol;Acc:HGNC:11875]		
ENSP00000357625.5 BCL2 interacting protein 3 [Source:HGNC Symbol;Acc:HGNC:1084]	K15464	BNIP3
ENSP00000495507.1 SLIT and NTRK like family member 6 [Source:HGNC Symbol;Acc:HGNC:23503]		
ENSP00000416534.2 DEAD-box helicase 46 [Source:HGNC Symbol;Acc:HGNC:18681]	K12811	DDX46, PRP5
ENSP00000370254.4 aldo-keto reductase family 1 member C1 [Source:HGNC Symbol;Acc:HGNC:384]	K00212	AKR1C1
ENSP00000478396.1 TELO2 interacting protein 2 [Source:HGNC Symbol;Acc:HGNC:26262]		
ENSP00000303222.3 mitochondrial carrier 2 [Source:HGNC Symbol;Acc:HGNC:17587]	K17885	MTCH
ENSP00000255120.5 nuclear autoantigenic sperm protein [Source:HGNC Symbol;Acc:HGNC:7644]	K11291	NASP
ENSP00000248142.6 WD repeat domain 24 [Source:HGNC Symbol;Acc:HGNC:20852]	K20408	WDR24
ENSP00000209875.4 chromobox 5 [Source:HGNC Symbol;Acc:HGNC:1555]	K11587	CBX5, HP1A
ENSP00000419465.1 WW domain containing transcription regulator 1 [Source:HGNC Symbol;Acc:HGNC:24042]	K16820	WWTR1, TAZ
ENSP00000389160.1 NADH:ubiquinone oxidoreductase subunit A11 [Source:HGNC Symbol;Acc:HGNC:20371]	K03956	NDUFA11
ENSP00000220325.4 EH domain containing 4 [Source:HGNC Symbol;Acc:HGNC:3245]	K12477	EHD4
ENSP00000252934.4 ataxin 10 [Source:HGNC Symbol;Acc:HGNC:10549]	K19323	ATXN10
ENSP00000397843.2 translation machinery associated 7 homolog [Source:HGNC Symbol;Acc:HGNC:26932]		
ENSP00000264718.3 GPN-loop GTPase 1 [Source:HGNC Symbol;Acc:HGNC:17030]	K06883	K06883
ENSP00000355802.4 epoxide hydrolase 1 [Source:HGNC Symbol;Acc:HGNC:3401]	K01253	EPHX1
ENSP00000377833.4 annexin A4 [Source:HGNC Symbol;Acc:HGNC:542]	K17093	ANXA4
ENSP00000296255.3 ribophorin I [Source:HGNC Symbol;Acc:HGNC:10381]	K12666	OST1, RPN1
ENSP00000499243.1 cytochrome P450 family 27 subfamily C member 1 [Source:HGNC Symbol;Acc:HGNC:33480]	K17951	CYP27C
ENSP00000376172.1 tissue factor pathway inhibitor [Source:HGNC Symbol;Acc:HGNC:11760]	K03909	TFPI
ENSP00000264170.4 kynureninase [Source:HGNC Symbol;Acc:HGNC:6469]	K01556	KYNU, kynU
ENSP00000299157.4 IKBKB interacting protein [Source:HGNC Symbol;Acc:HGNC:26430]		
ENSP00000318557.3 solute carrier family 12 member 4 [Source:HGNC Symbol;Acc:HGNC:10913]	K14427	SLC12A4_5_6, KCC
ENSP00000321259.3 transaldolase 1 [Source:HGNC Symbol;Acc:HGNC:11559]	K00616	E2.2.1.2, talA, talB
ENSP00000365773.3 phosphoserine aminotransferase 1 [Source:HGNC Symbol;Acc:HGNC:19129]	K00831	serC, PSAT1
ENSP00000297258.6 fatty acid binding protein 5 [Source:HGNC Symbol;Acc:HGNC:3560]	K08754	FABP5
ENSP00000360869.3 interferon induced protein with tetratricopeptide repeats 1 [Source:HGNC Symbol;Acc:HGN	K14217	IFIT1
ENSP00000229270.4 triosephosphate isomerase 1 [Source:HGNC Symbol;Acc:HGNC:12009]	K01803	TPI, tpiA
ENSP00000357980.3 HtrA serine peptidase 1 [Source:HGNC Symbol;Acc:HGNC:9476]	K08784	HTRA1, PRSS11
ENSP00000371188.2 calcium/calmodulin dependent protein kinase kinase 1 [Source:HGNC Symbol;Acc:HGNC:14	K00908	CAMKK1
ENSP00000340274.2 RAP2C, member of RAS oncogene family [Source:HGNC Symbol;Acc:HGNC:21165]	K07839	RAP2C
ENSP00000500914.1 embryonic ectoderm development [Source:HGNC Symbol;Acc:HGNC:3188]	K11462	EED
ENSP00000310861.3 keratin 2 [Source:HGNC Symbol;Acc:HGNC:6439]	K07605	KRT2
ENSP00000377027.2 transforming growth factor beta receptor associated protein 1 [Source:HGNC Symbol;Acc:H	K20177	VPS3, TGFBRAP1
ENSP00000362268.2 apolipoprotein O like [Source:HGNC Symbol;Acc:HGNC:24009]		
ENSP00000497376.1 protein S [Source:HGNC Symbol;Acc:HGNC:9456]		

ENSP00000215754.7	macrophage migration inhibitory factor [Source:HGNC Symbol;Acc:HGNC:7097]	K07253	MIF
ENSP00000233813.4	insulin like growth factor binding protein 5 [Source:HGNC Symbol;Acc:HGNC:5474]		
ENSP00000335636.4	N(alpha)-acetyltransferase 20, NatB catalytic subunit [Source:HGNC Symbol;Acc:HGNC:159(	K17972	NAA20, NAT3
ENSP00000354394.3	signal transducer and activator of transcription 1 [Source:HGNC Symbol;Acc:HGNC:11362]	K11220	STAT1
ENSP00000249014.4	CDC42 effector protein 1 [Source:HGNC Symbol;Acc:HGNC:17014]		
ENSP00000272430.5	rhotekin [Source:HGNC Symbol;Acc:HGNC:10466]		
ENSP00000373411.3	NFKB inhibitor interacting Ras like 1 [Source:HGNC Symbol;Acc:HGNC:17899]	K17197	NKIRAS
ENSP00000326305.4	solute carrier family 25 member 20 [Source:HGNC Symbol;Acc:HGNC:1421]	K15109	SLC25A20_29, CACT, CACI
ENSP00000486361.1	diazepam binding inhibitor, acyl-CoA binding protein [Source:HGNC Symbol;Acc:HGNC:26!	K08762	DBI, ACBP
ENSP00000494125.1	tropomyosin 4 [Source:HGNC Symbol;Acc:HGNC:12013]		
ENSP00000423972.1	nuclear receptor binding SET domain protein 2 [Source:HGNC Symbol;Acc:HGNC:12766]	K11424	WHSC1, MMSET, NSD2
ENSP00000362728.3	karyopherin subunit alpha 6 [Source:HGNC Symbol;Acc:HGNC:6399]		
ENSP00000244061.2	ring finger protein 114 [Source:HGNC Symbol;Acc:HGNC:13094]	K15697	RNF114
ENSP00000356110.4	nuclear casein kinase and cyclin dependent kinase substrate 1 [Source:HGNC Symbol;Acc:HGNC:29923]		
ENSP00000473469.1	DENN domain containing 4C [Source:HGNC Symbol;Acc:HGNC:26079]	K20163	DENND4
ENSP00000167586.6	keratin 14 [Source:HGNC Symbol;Acc:HGNC:6416]	K07604	KRT1
ENSP00000328694.6	MOB kinase activator 2 [Source:HGNC Symbol;Acc:HGNC:24904]		
ENSP00000356917.2	olfactomedin like 2B [Source:HGNC Symbol;Acc:HGNC:24558]		
ENSP00000225655.5	profilin 1 [Source:HGNC Symbol;Acc:HGNC:8881]	K05759	PFN
ENSP00000358857.4	emerin [Source:HGNC Symbol;Acc:HGNC:3331]	K12569	EMD
ENSP00000361411.3	zinc finger CCHC-type containing 24 [Source:HGNC Symbol;Acc:HGNC:26911]		
ENSP00000369889.3	collagen type II alpha 1 chain [Source:HGNC Symbol;Acc:HGNC:2200]	K19719	COL2A
ENSP00000358012.4	endosulfine alpha [Source:HGNC Symbol;Acc:HGNC:3360]		
ENSP00000483345.1	HDGF like 2 [Source:HGNC Symbol;Acc:HGNC:14680]		
ENSP00000268261.4	phosphomannomutase 2 [Source:HGNC Symbol;Acc:HGNC:9115]	K17497	PMM
ENSP00000439606.2	integrin linked kinase [Source:HGNC Symbol;Acc:HGNC:6040]		
ENSP00000361512.4	phosphoribosyl pyrophosphate synthetase 1 [Source:HGNC Symbol;Acc:HGNC:9462]	K00948	PRPS, prsA
ENSP00000457694.1	Jupiter microtubule associated homolog 2 [Source:HGNC Symbol;Acc:HGNC:14137]		
ENSP00000497691.1	amyloid beta precursor like protein 2 [Source:HGNC Symbol;Acc:HGNC:598]	K08117	APLP2
ENSP00000447091.1	solute carrier family 8 member B1 [Source:HGNC Symbol;Acc:HGNC:26175]	K13754	SLC24A6, NCKX6
ENSP00000348886.5	stomatin like 2 [Source:HGNC Symbol;Acc:HGNC:14559]		
ENSP00000499466.1	peptidylprolyl cis/trans isomerase, NIMA-interacting 4 [Source:HGNC Symbol;Acc:HGNC:89!	K09579	PIN4
ENSP00000365950.3	RNA binding motif protein 3 [Source:HGNC Symbol;Acc:HGNC:9900]	K13186	RBM3
ENSP00000333994.3	hemoglobin subunit beta [Source:HGNC Symbol;Acc:HGNC:4827]	K13823	HBB
ENSP00000270776.8	phosphogluconate dehydrogenase [Source:HGNC Symbol;Acc:HGNC:8891]	K00033	PGD, gnd, gntZ
ENSP00000428924.1	LYN proto-oncogene, Src family tyrosine kinase [Source:HGNC Symbol;Acc:HGNC:6735]		
ENSP00000269576.5	keratin 10 [Source:HGNC Symbol;Acc:HGNC:6413]	K07604	KRT1
ENSP00000498772.1	CLN5 intracellular trafficking protein [Source:HGNC Symbol;Acc:HGNC:2076]	K12390	CLN5

ENSP00000330075.5	2-oxoglutarate and iron dependent oxygenase domain containing 3 [Source:HGNC Symbol;Acc:HGNC:26174]		
ENSP00000437301.1	radixin [Source:HGNC Symbol;Acc:HGNC:9944]	K05762	RDX
ENSP00000363255.2	regulator of G protein signaling 3 [Source:HGNC Symbol;Acc:HGNC:9999]	K07524	RGS3
ENSP00000293261.2	transmembrane protein 143 [Source:HGNC Symbol;Acc:HGNC:25603]		
ENSP00000325527.5	fibrillin 1 [Source:HGNC Symbol;Acc:HGNC:3603]	K06825	FBN1
ENSP00000457912.1	sulfotransferase family 1A member 1 [Source:HGNC Symbol;Acc:HGNC:11453]	K01014	SULT1A
ENSP00000335304.4	dihydrolipoamide S-succinyltransferase [Source:HGNC Symbol;Acc:HGNC:2911]	K00658	DLST, sucB
ENSP00000496785.1	collagen type V alpha 2 chain [Source:HGNC Symbol;Acc:HGNC:2210]	K19721	COL5AS
ENSP00000364188.3	dolichyl-diphosphooligosaccharide--protein glycosyltransferase non-catalytic subunit [Sou	K12670	WBP1
ENSP00000353770.2	ribonucleotide reductase regulatory subunit M2 [Source:HGNC Symbol;Acc:HGNC:10452]	K10808	RRM2
ENSP00000410242.2	insulin like growth factor 2 mRNA binding protein 2 [Source:HGNC Symbol;Acc:HGNC:2886	K17392	IGF2BP2
ENSP00000465404.1	Parkinsonism associated deglycase [Source:HGNC Symbol;Acc:HGNC:16369]		
ENSP00000408730.1	coiled-coil domain containing 124 [Source:HGNC Symbol;Acc:HGNC:25171]		
ENSP00000369317.3	keratin 6A [Source:HGNC Symbol;Acc:HGNC:6443]	K07605	KRT2
ENSP00000406293.2	transcription elongation factor A3 [Source:HGNC Symbol;Acc:HGNC:11615]		
ENSP00000263100.2	alpha-1-B glycoprotein [Source:HGNC Symbol;Acc:HGNC:5]		
ENSP00000372218.1	serpin family B member 12 [Source:HGNC Symbol;Acc:HGNC:14220]		
ENSP00000264187.6	nidogen 1 [Source:HGNC Symbol;Acc:HGNC:7821]	K06826	NID
ENSP00000379366.2	MOB kinase activator 1B [Source:HGNC Symbol;Acc:HGNC:29801]	K06685	MOB1, Mats
ENSP00000330389.4	peptidyl-tRNA hydrolase domain containing 1 [Source:HGNC Symbol;Acc:HGNC:33782]		
ENSP00000222005.1	cell division cycle 37 [Source:HGNC Symbol;Acc:HGNC:1735]	K09554	CDC37
ENSP00000357643.3	marker of proliferation Ki-67 [Source:HGNC Symbol;Acc:HGNC:7107]	K17582	MKI67
ENSP00000303366.7	lectin, mannose binding 2 [Source:HGNC Symbol;Acc:HGNC:16986]	K10082	LMAN2, VIP36
ENSP00000304229.5	histidine triad nucleotide binding protein 1 [Source:HGNC Symbol;Acc:HGNC:4912]	K02503	HINT1, hinT, hit
ENSP00000380557.3	A-kinase anchoring protein 8 like [Source:HGNC Symbol;Acc:HGNC:29857]	K15978	AKAP8L, HA95
ENSP00000305494.6	spermatogenesis associated 5 like 1 [Source:HGNC Symbol;Acc:HGNC:28762]		
ENSP00000425906.1	LHFPL tetraspan subfamily member 2 [Source:HGNC Symbol;Acc:HGNC:6588]		
ENSP00000308541.5	coagulation factor II, thrombin [Source:HGNC Symbol;Acc:HGNC:3535]	K01313	F2
ENSP00000443194.1	N-acetyltransferase 1 [Source:HGNC Symbol;Acc:HGNC:7645]	K00622	nat
ENSP00000378431.1	multimerin 1 [Source:HGNC Symbol;Acc:HGNC:7178]		
ENSP00000311747.4	RNA binding motif protein 14 [Source:HGNC Symbol;Acc:HGNC:14219]	K13189	RBM14
ENSP00000452245.1	ribonuclease A family member 4 [Source:HGNC Symbol;Acc:HGNC:10047]	K16632	RNASE4
ENSP00000300584.3	TBC1 domain family member 2B [Source:HGNC Symbol;Acc:HGNC:29183]	K20166	TBC1D2B
ENSP00000362144.3	mitochondrial calcium uniporter [Source:HGNC Symbol;Acc:HGNC:23526]	K20858	MCU
ENSP00000383059.3	arginine and glutamate rich 1 [Source:HGNC Symbol;Acc:HGNC:25482]	K13173	ARGLU1
ENSP00000223357.3	AE binding protein 1 [Source:HGNC Symbol;Acc:HGNC:303]		
ENSP00000351695.4	pancreatic lipase related protein 1 [Source:HGNC Symbol;Acc:HGNC:9156]	K14074	PNLIPRP1, PLRP1
ENSP00000307940.5	eukaryotic translation elongation factor 2 [Source:HGNC Symbol;Acc:HGNC:3214]	K03234	EEF2

ENSP00000480626.1	PR/SET domain 11 [Source:HGNC Symbol;Acc:HGNC:13996]		
ENSP00000359665.3	phosphatidylinositol 4-kinase type 2 alpha [Source:HGNC Symbol;Acc:HGNC:30031]	K13711	PI4K2
ENSP00000233057.4	eukaryotic translation initiation factor 2 alpha kinase 2 [Source:HGNC Symbol;Acc:HGNC:94	K16195	EIF2AK2
ENSP00000303145.4	transmembrane p24 trafficking protein 10 [Source:HGNC Symbol;Acc:HGNC:16998]	K20352	TMED10, ERV25
ENSP00000356280.3	pleckstrin homology like domain family A member 3 [Source:HGNC Symbol;Acc:HGNC:8934]		
ENSP00000247970.4	peptidylprolyl cis/trans isomerase, NIMA-interacting 1 [Source:HGNC Symbol;Acc:HGNC:89	K09578	PIN1
ENSP00000242784.3	telomerase RNA component interacting RNase [Source:HGNC Symbol;Acc:HGNC:28424]		
ENSP00000476176.2	solute carrier family 37 member 4 [Source:HGNC Symbol;Acc:HGNC:4061]		
ENSP00000417864.2	acidic nuclear phosphoprotein 32 family member A [Source:HGNC Symbol;Acc:HGNC:1323	K18646	ANP32A_C_D
ENSP00000274311.2	pelota mRNA surveillance and ribosome rescue factor [Source:HGNC Symbol;Acc:HGNC:882	K06965	PELO, DOM34, pelA
ENSP00000308452.8	keratin 17 [Source:HGNC Symbol;Acc:HGNC:6427]	K07604	KRT1
ENSP00000309166.7	RNA binding motif protein 4 [Source:HGNC Symbol;Acc:HGNC:9901]	K13187	RBM4
ENSP00000373706.4	diaphanous related formin 1 [Source:HGNC Symbol;Acc:HGNC:2876]	K05740	DIAPH1
ENSP00000349748.5	splicing factor proline and glutamine rich [Source:HGNC Symbol;Acc:HGNC:10774]	K13219	SFPQ, PSF
ENSP00000273395.4	BOC cell adhesion associated, oncogene regulated [Source:HGNC Symbol;Acc:HGNC:17173	K20020	BOC
ENSP00000398597.1	exosome component 6 [Source:HGNC Symbol;Acc:HGNC:19055]	K12587	MTR3, EXOSC6
ENSP00000457733.1	solute carrier family 25 member 24 [Source:HGNC Symbol;Acc:HGNC:20662]	K14684	SLC25A23S
ENSP00000341170.2	pleiotrophin [Source:HGNC Symbol;Acc:HGNC:9630]	K16642	PTN
ENSP00000414921.1	polypyrimidine tract binding protein 3 [Source:HGNC Symbol;Acc:HGNC:10253]	K17844	PTBP3, ROD1
ENSP00000499082.1	crystallin alpha B [Source:HGNC Symbol;Acc:HGNC:2389]	K09542	CRYAB
ENSP00000325146.8	collagen type XII alpha 1 chain [Source:HGNC Symbol;Acc:HGNC:2188]	K08132	COL12A
ENSP00000258739.4	KDEL endoplasmic reticulum protein retention receptor 2 [Source:HGNC Symbol;Acc:HGNC	K10949	KDELR
ENSP00000452123.1	serine and arginine rich splicing factor 5 [Source:HGNC Symbol;Acc:HGNC:10787]	K12893	SFRS4_5_6
ENSP00000229794.4	mitogen-activated protein kinase 14 [Source:HGNC Symbol;Acc:HGNC:6876]		
ENSP00000396899.2	inner membrane mitochondrial protein [Source:HGNC Symbol;Acc:HGNC:6047]	K17785	IMMT, FCJ1, MNOS2
ENSP00000332723.2	collectin subfamily member 10 [Source:HGNC Symbol;Acc:HGNC:2220]	K10065	COLEC10
ENSP00000359464.3	opioid growth factor receptor like 1 [Source:HGNC Symbol;Acc:HGNC:21378]		
ENSP00000352167.3	solute carrier family 25 member 29 [Source:HGNC Symbol;Acc:HGNC:20116]	K15109	SLC25A20_29, CACT, CACI
ENSP00000370588.3	CD99 molecule (Xg blood group) [Source:HGNC Symbol;Acc:HGNC:7082]	K06520	CD99, MIC2
ENSP00000493835.1	RING1 and YY1 binding protein [Source:NCBI gene;Acc:23429]	K11469	RYBP
ENSP00000305193.3	HCLS1 binding protein 3 [Source:HGNC Symbol;Acc:HGNC:24979]		
ENSP00000307288.5	minichromosome maintenance complex component 7 [Source:HGNC Symbol;Acc:HGNC:69	K02210	MCM7, CDC47
ENSP00000286234.5	DEP domain containing MTOR interacting protein [Source:HGNC Symbol;Acc:HGNC:22953]	K20402	DEPTOR
ENSP00000418593.1	sorting nexin family member 21 [Source:HGNC Symbol;Acc:HGNC:16154]	K17932	SNX21
ENSP00000345083.4	mitogen-activated protein kinase kinase 3 [Source:HGNC Symbol;Acc:HGNC:6843]	K04432	MAP2K3, MKK3
ENSP00000362014.3	dynamin 1 [Source:HGNC Symbol;Acc:HGNC:2972]	K01528	DNM
ENSP00000419765.1	bromodomain containing 9 [Source:HGNC Symbol;Acc:HGNC:25818]	K11723	BRD7_9
ENSP00000281830.4	potassium voltage-gated channel subfamily E regulatory subunit 4 [Source:HGNC Symbol;A	K04898	KCNE4

ENSP00000394670.1	peptidylprolyl isomerase A [Source:HGNC Symbol;Acc:HGNC:9253]	K03767	PPIA
ENSP00000359380.2	stearoyl-CoA desaturase [Source:HGNC Symbol;Acc:HGNC:10571]	K00507	SCD, desC
ENSP00000354255.4	carboxypeptidase Z [Source:HGNC Symbol;Acc:HGNC:2333]	K13022	CPZ
ENSP00000412922.1	ubiquitin related modifier 1 [Source:HGNC Symbol;Acc:HGNC:28378]	K12161	URM1
ENSP00000461388.1	OVCA2 serine hydrolase domain containing [Source:HGNC Symbol;Acc:HGNC:24203]		
ENSP00000216122.3	minichromosome maintenance complex component 5 [Source:HGNC Symbol;Acc:HGNC:69	K02209	MCM5, CDC46
ENSP00000367123.2	solute carrier family 3 member 2 [Source:HGNC Symbol;Acc:HGNC:11026]		
ENSP00000384886.1	IFI30 lysosomal thiol reductase [Source:HGNC Symbol;Acc:HGNC:5398]	K08059	IFI30, GILT
ENSP00000340691.4	eukaryotic translation initiation factor 4E binding protein 1 [Source:HGNC Symbol;Acc:HGN	K07205	EIF4EBP1
ENSP00000322926.6	FERM, ARH/RhoGEF and pleckstrin domain protein 1 [Source:HGNC Symbol;Acc:HGNC:359	K17477	FARP1
ENSP00000318845.4	synaptogyrin 1 [Source:HGNC Symbol;Acc:HGNC:11498]		
ENSP00000270142.6	superoxide dismutase 1 [Source:HGNC Symbol;Acc:HGNC:11179]	K04565	SOD1
ENSP00000247470.9	PYD and CARD domain containing [Source:HGNC Symbol;Acc:HGNC:16608]	K12799	PYCARD, ASC
ENSP00000332455.3	karyopherin subunit alpha 2 [Source:HGNC Symbol;Acc:HGNC:6395]	K15043	KPNA2
ENSP00000252242.4	keratin 5 [Source:HGNC Symbol;Acc:HGNC:6442]	K07605	KRT2
ENSP00000220772.3	secreted frizzled related protein 1 [Source:HGNC Symbol;Acc:HGNC:10776]		
ENSP00000297632.6	transmembrane protein 65 [Source:HGNC Symbol;Acc:HGNC:25203]		
ENSP00000228938.5	matrix Gla protein [Source:HGNC Symbol;Acc:HGNC:7060]		
ENSP00000377862.3	FAT atypical cadherin 4 [Source:HGNC Symbol;Acc:HGNC:23109]	K16669	FAT4
ENSP00000368876.3	TOG array regulator of axonemal microtubules 2 [Source:HGNC Symbol;Acc:HGNC:33715]		
ENSP00000385714.1	novel protein, AP000351.4-DDT readtrhough	K10028	DDT
ENSP00000261811.4	cysteine rich transmembrane module containing 1 [Source:HGNC Symbol;Acc:HGNC:30239]		
ENSP00000441365.1	MARVEL domain containing 1 [Source:HGNC Symbol;Acc:HGNC:28674]		
ENSP00000459533.1	lipopolysaccharide induced TNF factor [Source:HGNC Symbol;Acc:HGNC:16841]	K19363	LITAF
ENSP00000500157.1	DNA primase subunit 1 [Source:HGNC Symbol;Acc:HGNC:9369]		
ENSP00000370543.3	solute carrier family 5 member 3 [Source:HGNC Symbol;Acc:HGNC:11038]	K14383	SLC5A3, SMIT
ENSP00000230124.3	FIG4 phosphoinositide 5-phosphatase [Source:HGNC Symbol;Acc:HGNC:16873]		
ENSP00000498932.1	chromosome 11 open reading frame 96 [Source:HGNC Symbol;Acc:HGNC:38675]		
ENSP00000367541.1	tropomyosin 2 [Source:HGNC Symbol;Acc:HGNC:12011]		
ENSP00000005558.4	interferon related developmental regulator 1 [Source:HGNC Symbol;Acc:HGNC:5456]		
ENSP00000310572.6	proteasome 26S subunit, ATPase 5 [Source:HGNC Symbol;Acc:HGNC:9552]	K03066	PSMC5, RPT6
ENSP00000462316.2	phosphodiesterase 4D interacting protein [Source:HGNC Symbol;Acc:HGNC:15580]	K16549	PDE4DIP
ENSP00000360034.2	SERPINE1 mRNA binding protein 1 [Source:HGNC Symbol;Acc:HGNC:17860]	K13199	SERBP1
ENSP00000247977.3	F-box and leucine rich repeat protein 12 [Source:HGNC Symbol;Acc:HGNC:13611]	K10278	FBXL12
ENSP00000419970.1	cytochrome p450 oxidoreductase [Source:HGNC Symbol;Acc:HGNC:9208]	K00327	POR
ENSP00000362345.1	apoptosis inducing factor mitochondria associated 2 [Source:HGNC Symbol;Acc:HGNC:21411]		
ENSP00000377372.3	growth associated protein 43 [Source:HGNC Symbol;Acc:HGNC:4140]	K20041	GAP43
ENSP00000295317.3	ring finger protein 149 [Source:HGNC Symbol;Acc:HGNC:23137]	K15704	RNF149

ENSP00000272928.3	atypical chemokine receptor 3 [Source:HGNC Symbol;Acc:HGNC:23692]	K04304	CXCR7, RDC1
ENSP00000387259.2	mitogen-activated protein kinase kinase kinase 20 [Source:HGNC Symbol;Acc:HGNC:17797]	K04424	ZAK, MLTK
ENSP00000319739.5	reticulocalbin 2 [Source:HGNC Symbol;Acc:HGNC:9935]		
ENSP00000354878.5	kinesin family member 21A [Source:HGNC Symbol;Acc:HGNC:19349]	K10395	KIF4_21_27
ENSP00000297161.2	BMP binding endothelial regulator [Source:HGNC Symbol;Acc:HGNC:24154]		
ENSP00000250340.3	C-type lectin domain containing 11A [Source:HGNC Symbol;Acc:HGNC:10576]	K17521	CLEC11A
ENSP00000433138.1	caspase 1 [Source:HGNC Symbol;Acc:HGNC:1499]	K01370	CASP1
