# Supplementary material for: Deficiency of MIF Accentuates Overloaded Compression-Induced Nucleus Pulposus Cell Oxidative Damage via Depressing Mitophagy
Source: Oxid Med Cell Longev. 2021 Jul 1;2021:6192498. doi: 10.1155/2021/6192498 (PMC8270705; doi:10.1155/2021/6192498)
Supplement: Supplementary Materials — Supplementary Figure 1: preoperative MRI image of the patient's lumbar IVDs and the gross view of the separated NP tissues. Supplementary Table 1: annotation detail of the DEPs between the LC loading group and the control group. Supplementary Table 2: annotation detail of the DEPs between the HC loading group and the control group. Supplementary Table 3: annotation detail of the DEPs between the HC loading group and the LC loading group. [file 6192498.f1.zip › 6192498.f4.pdf]

Accession_id	Description	KO_id	KO_name
ENSP00000398370.2	eukaryotic translation initiation factor 4A2 [Source:HGNC Symbol;Acc:HGNC:3284]	K03257	EIF4A
ENSP00000406705.2	histamine receptor H1 [Source:HGNC Symbol;Acc:HGNC:5182]	K04149	HRH1
ENSP00000269228.4	NPC intracellular cholesterol transporter 1 [Source:HGNC Symbol;Acc:HGNC:7897]	K12385	NPC1
ENSP00000367615.3	adenine phosphoribosyltransferase [Source:HGNC Symbol;Acc:HGNC:626]	K00759	APRT, apt
ENSP00000300605.4	haloacid dehalogenase like hydrolase domain containing 2 [Source:HGNC Symbol;Acc:HGNC:25364]		
ENSP00000465894.1	metallophosphoesterase 1 [Source:HGNC Symbol;Acc:HGNC:15988]		
ENSP00000296577.4	ATP binding cassette subfamily E member 1 [Source:HGNC Symbol;Acc:HGNC:69]	K06174	ABCE1, Rli1
ENSP00000490664.1	glutaminyl-tRNA synthetase [Source:HGNC Symbol;Acc:HGNC:9751]	K01886	QARS, glnS
ENSP00000479636.1	phospholipase C delta 3 [Source:HGNC Symbol;Acc:HGNC:9061]	K05857	PLCD
ENSP00000413218.3	peroxiredoxin like 2B [Source:HGNC Symbol;Acc:HGNC:28390]		
ENSP00000340836.6	osteoclast stimulating factor 1 [Source:HGNC Symbol;Acc:HGNC:8510]		
ENSP00000261407.4	lysophosphatidylcholine acyltransferase 3 [Source:HGNC Symbol;Acc:HGNC:30244]	K13515	LPCAT3, MBOAT5
ENSP00000354876.1	mitochondrially encoded cytochrome c oxidase II [Source:HGNC Symbol;Acc:HGNC:7421]	K02261	COX2
ENSP00000361917.3	armadillo repeat containing X-linked 1 [Source:HGNC Symbol;Acc:HGNC:18073]		
ENSP00000288774.3	peroxisomal biogenesis factor 10 [Source:HGNC Symbol;Acc:HGNC:8851]		
ENSP00000221930.4	transforming growth factor beta 1 [Source:HGNC Symbol;Acc:HGNC:11766]		
ENSP00000336988.7	G protein-coupled receptor 107 [Source:HGNC Symbol;Acc:HGNC:17830]		
ENSP00000253332.1	A-kinase anchoring protein 12 [Source:HGNC Symbol;Acc:HGNC:370]	K16528	AKAP12
ENSP00000340684.3	monoamine oxidase A [Source:HGNC Symbol;Acc:HGNC:6833]	K00274	MAO, aofH
ENSP00000370055.4	ubiquitin like 3 [Source:HGNC Symbol;Acc:HGNC:12504]		
ENSP00000425561.1	eukaryotic translation initiation factor 4E [Source:HGNC Symbol;Acc:HGNC:3287]		
ENSP00000398064.2	HIG1 hypoxia inducible domain family member 1A [Source:HGNC Symbol;Acc:HGNC:29527]		
ENSP00000354687.2	mitochondrially encoded NADH:ubiquinone oxidoreductase core subunit 1 [Source:HGNC Syn	K03878	ND1
ENSP00000379051.2	purine rich element binding protein B [Source:HGNC Symbol;Acc:HGNC:9702]		
ENSP00000373620.3	heat shock protein family D (Hsp60) member 1 [Source:HGNC Symbol;Acc:HGNC:5261]	K04077	groEL, HSPD1
ENSP00000305675.4	caveolae associated protein 2 [Source:HGNC Symbol;Acc:HGNC:10690]		
ENSP00000358737.3	ATP synthase peripheral stalk-membrane subunit b [Source:HGNC Symbol;Acc:HGNC:840]	K02127	ATPeF0B, ATP5F1, ATP
ENSP00000421592.2	Aly/REF export factor [Source:HGNC Symbol;Acc:HGNC:19071]	K12881	THOC4, ALY
ENSP00000345719.4	torsin family 1 member A [Source:HGNC Symbol;Acc:HGNC:3098]		
ENSP00000346549.2	regulator of microtubule dynamics 2 [Source:HGNC Symbol;Acc:HGNC:26567]		
ENSP00000044462.7	proteasome subunit alpha 4 [Source:HGNC Symbol;Acc:HGNC:9533]	K02728	PSMA4
ENSP00000258439.2	transmembrane protein 127 [Source:HGNC Symbol;Acc:HGNC:26038]		
ENSP00000385697.1	NADH:ubiquinone oxidoreductase subunit A10 [Source:HGNC Symbol;Acc:HGNC:7684]	K03954	NDUFA10
ENSP00000333551.3	pyridoxal phosphate binding protein [Source:HGNC Symbol;Acc:HGNC:9457]	K06997	yggS, PROSC
ENSP00000442050.2	phosphate cytidylyltransferase 2, ethanolamine [Source:HGNC Symbol;Acc:HGNC:8756]		
ENSP00000245046.2	ER membrane protein complex subunit 3 [Source:HGNC Symbol;Acc:HGNC:23999]		
ENSP00000263275.3	outer mitochondrial membrane lipid metabolism regulator OPA3 [Source:HGNC Symbol;Acc:HGNC:8142]		

ENSP00000257694.8	patatin like phospholipase domain containing 8 [Source:HGNC Symbol;Acc:HGNC:28900]	K16815	PNPLA8
ENSP00000379133.3	APAF1 interacting protein [Source:HGNC Symbol;Acc:HGNC:17581]	K08964	mtnB
ENSP00000362639.3	Rab9 effector protein with kelch motifs [Source:HGNC Symbol;Acc:HGNC:16896]	K20285	RABEPK
ENSP00000371388.3	sulfiredoxin 1 [Source:HGNC Symbol;Acc:HGNC:16132]	K12260	SRX1
ENSP00000385152.3	KIAA1671 [Source:HGNC Symbol;Acc:HGNC:29345]		
ENSP00000362207.4	carbohydrate sulfotransferase 3 [Source:HGNC Symbol;Acc:HGNC:1971]	K01020	CHST3
ENSP00000340211.5	coronin 1B [Source:HGNC Symbol;Acc:HGNC:2253]	K13886	CORO1B_1C_6
ENSP00000405573.3	glucose-6-phosphate isomerase [Source:HGNC Symbol;Acc:HGNC:4458]	K01810	GPI, pgi
ENSP00000424707.1	transmembrane protein 167A [Source:HGNC Symbol;Acc:HGNC:28330]		
ENSP00000363092.4	protein kinase cGMP-dependent 1 [Source:HGNC Symbol;Acc:HGNC:9414]		
ENSP00000265260.3	PEST proteolytic signal containing nuclear protein [Source:HGNC Symbol;Acc:HGNC:30023]		
ENSP00000367050.5	reticulon 3 [Source:HGNC Symbol;Acc:HGNC:10469]	K20723	RTN3
ENSP00000373073.3	urocanate hydratase 1 [Source:HGNC Symbol;Acc:HGNC:26444]	K01712	hutU, UROC1
ENSP00000486116.1	mitogen-activated protein kinase kinase kinase kinase 4 [Source:HGNC Symbol;Acc:HGNC:6866]		
ENSP00000376374.2	thymocyte nuclear protein 1 [Source:HGNC Symbol;Acc:HGNC:29560]		
ENSP00000264993.3	CDV3 homolog [Source:HGNC Symbol;Acc:HGNC:26928]		
ENSP00000449337.1	CD63 molecule [Source:HGNC Symbol;Acc:HGNC:1692]	K06497	CD63, MLA1, TSPAN3C
ENSP00000346694.4	heterogeneous nuclear ribonucleoprotein A2/B1 [Source:HGNC Symbol;Acc:HGNC:5033]	K13158	HNRNPA2B1
ENSP00000252809.3	growth differentiation factor 15 [Source:HGNC Symbol;Acc:HGNC:30142]	K05504	GDF15
ENSP00000387739.2	angiomotin like 1 [Source:HGNC Symbol;Acc:HGNC:17811]	K06104	AMOTL
ENSP00000498205.1	programmed cell death 11 [Source:HGNC Symbol;Acc:HGNC:13408]	K14792	RRP5, PDCD11
ENSP00000355800.3	transmembrane protein 63A [Source:HGNC Symbol;Acc:HGNC:29118]		
ENSP00000488378.1	paraoxonase 2 [Source:HGNC Symbol;Acc:HGNC:9205]	K01045	PON
ENSP00000423482.1	calcium/calmodulin dependent protein kinase II delta [Source:HGNC Symbol;Acc:HGNC:1462]		
ENSP00000262134.5	lysophosphatidylcholine acyltransferase 2 [Source:HGNC Symbol;Acc:HGNC:26032]	K13510	LPCAT1_2
ENSP00000366800.5	DnaJ heat shock protein family (Hsp40) member C11 [Source:HGNC Symbol;Acc:HGNC:25570]	K09531	DNAJC11
ENSP00000354111.4	DnaJ heat shock protein family (Hsp40) member C5 [Source:HGNC Symbol;Acc:HGNC:16235]	K09525	DNAJC5
ENSP00000312773.4	NFKB inhibitor interacting Ras like 2 [Source:HGNC Symbol;Acc:HGNC:17898]	K17197	NKIRAS
ENSP00000339613.5	protein O-fucosyltransferase 2 [Source:HGNC Symbol;Acc:HGNC:14683]	K03691	POFUT
ENSP00000490217.1	negative regulator of P-body association [Source:HGNC Symbol;Acc:HGNC:50713]		
ENSP00000392330.2	B cell receptor associated protein 31 [Source:HGNC Symbol;Acc:HGNC:16695]	K14009	BCAP31, BAP31
ENSP00000473259.1	protein tyrosine phosphatase 4A2 [Source:HGNC Symbol;Acc:HGNC:9635]	K18041	PTP4A
ENSP00000205948.6	apolipoprotein H [Source:HGNC Symbol;Acc:HGNC:616]	K17305	APOH, B2G1
ENSP00000358105.3	aph-1 homolog A, gamma-secretase subunit [Source:HGNC Symbol;Acc:HGNC:29509]	K06172	APH1A
ENSP00000477781.1	tumor protein, translationally-controlled 1 [Source:HGNC Symbol;Acc:HGNC:12022]		
ENSP00000270225.6	SUMO1 activating enzyme subunit 1 [Source:HGNC Symbol;Acc:HGNC:30660]	K10684	UBLE1A, SAE1
ENSP00000216455.4	proteasome subunit alpha 3 [Source:HGNC Symbol;Acc:HGNC:9532]	K02727	PSMA3
ENSP00000267884.6	signal recognition particle 14 [Source:HGNC Symbol;Acc:HGNC:11299]	K03104	SRP14

ENSP00000283882.3	craniofacial development protein 1 [Source:HGNC Symbol;Acc:HGNC:1873]		
ENSP00000305230.7	signal recognition particle 9 [Source:HGNC Symbol;Acc:HGNC:11304]	K03109	SRP9
ENSP00000354554.2	mitochondrially encoded cytochrome b [Source:HGNC Symbol;Acc:HGNC:7427]	K00412	CYTB, petB
ENSP00000277010.4	sigma non-opioid intracellular receptor 1 [Source:HGNC Symbol;Acc:HGNC:8157]	K20719	SIGMAR1
ENSP00000250498.4	defender against cell death 1 [Source:HGNC Symbol;Acc:HGNC:2664]	K12668	OST2, DAD1
ENSP00000327070.5	malate dehydrogenase 2 [Source:HGNC Symbol;Acc:HGNC:6971]	K00026	MDH2
ENSP00000494186.1	gasdermin E [Source:HGNC Symbol;Acc:HGNC:2810]		
ENSP00000226230.6	transmembrane protein 97 [Source:HGNC Symbol;Acc:HGNC:28106]		
ENSP00000498679.1	clustered mitochondria homolog [Source:HGNC Symbol;Acc:HGNC:29094]	K03255	TIF31, CLU1
ENSP00000221957.3	perilipin 3 [Source:HGNC Symbol;Acc:HGNC:16893]	K20287	PLIN3
ENSP00000345957.3	ribosomal protein S21 [Source:HGNC Symbol;Acc:HGNC:10409]	K02971	RP-S21e, RPS21
ENSP00000299633.4	HDGF like 3 [Source:HGNC Symbol;Acc:HGNC:24937]		
ENSP00000373918.3	glycyl-tRNA synthetase [Source:HGNC Symbol;Acc:HGNC:4162]	K01880	GARS, glyS1
ENSP00000497648.1	minichromosome maintenance complex component 4 [Source:HGNC Symbol;Acc:HGNC:6947]		
ENSP00000338934.7	ezrin [Source:HGNC Symbol;Acc:HGNC:12691]	K08007	VIL2
ENSP00000365458.4	heterogeneous nuclear ribonucleoprotein K [Source:HGNC Symbol;Acc:HGNC:5044]	K12886	HNRNPK
ENSP00000337736.3	A-kinase anchoring protein 1 [Source:HGNC Symbol;Acc:HGNC:367]	K16518	AKAP1
ENSP00000363205.3	NFS1 cysteine desulfurase [Source:HGNC Symbol;Acc:HGNC:15910]	K04487	iscS, NFS1
ENSP00000270502.5	translocase of inner mitochondrial membrane 29 [Source:HGNC Symbol;Acc:HGNC:25152]		
ENSP00000312250.8	ADP dependent glucokinase [Source:HGNC Symbol;Acc:HGNC:25250]	K08074	ADPGK
ENSP00000260746.4	ADP ribosylation factor like GTPase 3 [Source:HGNC Symbol;Acc:HGNC:694]	K07944	ARL3
ENSP00000323313.7	protein disulfide isomerase family A member 5 [Source:HGNC Symbol;Acc:HGNC:24811]	K09583	PDIA5
ENSP00000385923.3	nucleobindin 1 [Source:HGNC Symbol;Acc:HGNC:8043]	K20371	NUCB
ENSP00000251047.4	lectin, mannose binding 1 [Source:HGNC Symbol;Acc:HGNC:6631]	K10080	LMAN1, ERGIC53
ENSP00000252595.6	solute carrier family 27 member 1 [Source:HGNC Symbol;Acc:HGNC:10995]	K08745	SLC27A1_4, FATP1, FA
ENSP00000368468.3	mitochondrial calcium uniporter regulator 1 [Source:HGNC Symbol;Acc:HGNC:21097]		
ENSP00000054666.6	vesicle associated membrane protein 3 [Source:HGNC Symbol;Acc:HGNC:12644]	K13505	VAMP3
ENSP00000272452.2	sulfotransferase family 1C member 4 [Source:HGNC Symbol;Acc:HGNC:11457]	K01025	E2.8.2.-
ENSP00000398131.2	G1 to S phase transition 1 [Source:HGNC Symbol;Acc:HGNC:4621]	K03267	ERF3, GSPT
ENSP00000497362.1	glycoprotein nmb [Source:HGNC Symbol;Acc:HGNC:4462]		
ENSP00000293362.1	proteasome activator subunit 3 [Source:HGNC Symbol;Acc:HGNC:9570]		
ENSP00000357998.4	SEC63 homolog, protein translocation regulator [Source:HGNC Symbol;Acc:HGNC:21082]	K09540	SEC63, DNAJC23
ENSP00000384116.1	Sad1 and UNC84 domain containing 1 [Source:HGNC Symbol;Acc:HGNC:18587]		
ENSP00000405926.3	transmembrane p24 trafficking protein 7 [Source:HGNC Symbol;Acc:HGNC:24253]	K20349	TMED7
ENSP00000296417.5	H2A histone family member Z [Source:HGNC Symbol;Acc:HGNC:4741]	K11251	H2A
ENSP00000233893.5	heat shock protein family E (Hsp10) member 1 [Source:HGNC Symbol;Acc:HGNC:5269]	K04078	groES, HSPE1
ENSP00000262613.5	SLC9A3 regulator 1 [Source:HGNC Symbol;Acc:HGNC:11075]	K13365	SLC9A3R1, NHERF1
ENSP00000481714.1	N-ethylmaleimide sensitive factor, vesicle fusing ATPase [Source:HGNC Symbol;Acc:HGNC:801	K06027	NSF, SEC18

ENSP00000245185.5	metallothionein 2A [Source:HGNC Symbol;Acc:HGNC:7406]	K14739	MT1_2
ENSP00000378995.1	ATP synthase F1 subunit delta [Source:HGNC Symbol;Acc:HGNC:837]	K02134	ATPeF1D, ATP5D, ATP[illegible]
ENSP00000417175.1	VAMP associated protein B and C [Source:HGNC Symbol;Acc:HGNC:12649]	K10707	VAPB, ALS8
ENSP00000350848.2	high mobility group nucleosome binding domain 5 [Source:HGNC Symbol;Acc:HGNC:8013]	K17045	HMGN5
ENSP00000453581.2	aggrecan [Source:HGNC Symbol;Acc:HGNC:319]	K06792	AGC1, CSPG1
ENSP00000280346.6	dihydrolipoamide S-acetyltransferase [Source:HGNC Symbol;Acc:HGNC:2896]	K00627	DLAT, aceF, pdhC
ENSP00000358565.4	solute carrier family 35 member A1 [Source:HGNC Symbol;Acc:HGNC:11021]	K15272	SLC35A1_2_3
ENSP00000359130.4	solute carrier family 30 member 7 [Source:HGNC Symbol;Acc:HGNC:19306]	K14692	SLC30A5_7, ZNT5_7, M[illegible]
ENSP00000361140.3	aldo-keto reductase family 1 member A1 [Source:HGNC Symbol;Acc:HGNC:380]	K00002	AKR1A1, adh
ENSP00000417677.1	zinc finger and BTB domain containing 8 opposite strand [Source:HGNC Symbol;Acc:HGNC:24094]		
ENSP00000386226.3	RNA binding motif protein 34 [Source:HGNC Symbol;Acc:HGNC:28965]	K14837	NOP12
ENSP00000389906.1	protein disulfide isomerase family A member 3 [Source:HGNC Symbol;Acc:HGNC:4606]		
ENSP00000417763.1	N(alpha)-acetyltransferase 10, NatA catalytic subunit [Source:HGNC Symbol;Acc:HGNC:18704]	K20791	NAA10_11, ARD1_2
ENSP00000334113.5	dehydrogenase/reductase X-linked [Source:HGNC Symbol;Acc:HGNC:18399]	K11170	DHRSX
ENSP00000482106.1	DEAD-box helicase 24 [Source:HGNC Symbol;Acc:HGNC:13266]	K14805	DDX24, MAK5
ENSP00000380514.3	hydroxyacylglutathione hydrolase [Source:HGNC Symbol;Acc:HGNC:4805]	K01069	E3.1.2.6, gloB
ENSP00000277865.4	glutamate dehydrogenase 1 [Source:HGNC Symbol;Acc:HGNC:4335]	K00261	GLUD1_2, gdhA
ENSP00000285848.5	OXA1L mitochondrial inner membrane protein [Source:HGNC Symbol;Acc:HGNC:8526]	K03217	yidC, spoIIIJ, OXA1, ccf
ENSP00000239878.4	ubiquitin fold modifier 1 [Source:HGNC Symbol;Acc:HGNC:20597]	K12162	UFM1
ENSP00000318066.3	ectonucleotide pyrophosphatase/phosphodiesterase 4 [Source:HGNC Symbol;Acc:HGNC:3359[illegible]	K18424	ENPP4
ENSP00000281828.6	phenylalanyl-tRNA synthetase subunit beta [Source:HGNC Symbol;Acc:HGNC:17800]	K01890	FARSB, pheT
ENSP00000297578.4	solute carrier family 25 member 32 [Source:HGNC Symbol;Acc:HGNC:29683]	K15115	SLC25A32, MFT
ENSP00000356652.2	calcyclin binding protein [Source:HGNC Symbol;Acc:HGNC:30423]	K04507	CACYBP, SIP
ENSP00000254654.3	ILK associated serine/threonine phosphatase [Source:HGNC Symbol;Acc:HGNC:15566]	K17500	ILKAP
ENSP00000354632.2	mitochondrially encoded ATP synthase membrane subunit 6 [Source:HGNC Symbol;Acc:HGNC[illegible]	K02126	ATPeF0A, MTATP6, AT[illegible]
ENSP00000356015.4	acetyl-CoA acetyltransferase 2 [Source:HGNC Symbol;Acc:HGNC:94]	K00626	E2.3.1.9, atoB
ENSP00000356770.3	coagulation factor V [Source:HGNC Symbol;Acc:HGNC:3542]	K03902	F5
ENSP00000348163.3	plastin 3 [Source:HGNC Symbol;Acc:HGNC:9091]	K17336	PLS3
ENSP00000304642.9	reticulophagy regulator 1 [Source:HGNC Symbol;Acc:HGNC:25964]		
ENSP00000499556.1	coiled-coil domain containing 57 [Source:HGNC Symbol;Acc:HGNC:27564]		
ENSP00000262455.6	endoplasmic reticulum protein 44 [Source:HGNC Symbol;Acc:HGNC:18311]	K17264	ERP44, TXNDC4
ENSP00000234590.4	enolase 1 [Source:HGNC Symbol;Acc:HGNC:3350]	K01689	ENO, eno
ENSP00000262435.9	SMAD specific E3 ubiquitin protein ligase 2 [Source:HGNC Symbol;Acc:HGNC:16809]	K04678	SMURF
ENSP00000054950.3	reticulocalbin 1 [Source:HGNC Symbol;Acc:HGNC:9934]		
ENSP00000500707.1	pep chromosome:GRCh38:CHR_HG2499_PATCH:101984125:101986741:1 gene:ENSG00000288336.1 transcript:ENST00000673[illegible]		
ENSP00000436759.1	signal peptidase complex subunit 2 [Source:HGNC Symbol;Acc:HGNC:28962]		
ENSP00000308369.2	LEM domain containing 3 [Source:HGNC Symbol;Acc:HGNC:28887]	K19410	LEMD3
ENSP00000237596.2	polycystin 2, transient receptor potential cation channel [Source:HGNC Symbol;Acc:HGNC:900[illegible]	K04986	PKD2

ENSP00000264932.6	succinate dehydrogenase complex flavoprotein subunit A [Source:HGNC Symbol;Acc:HGNC:1(	K00234	SDHA, SDH1
ENSP00000361812.1	SYS1 golgi trafficking protein [Source:HGNC Symbol;Acc:HGNC:16162]	K20318	SYS1
ENSP00000286448.6	vesicle associated membrane protein 7 [Source:HGNC Symbol;Acc:HGNC:11486]	K08515	VAMP7
ENSP00000499391.1	deleted in malignant brain tumors 1 [Source:HGNC Symbol;Acc:HGNC:2926]		
ENSP00000418690.1	geranylgeranyl diphosphate synthase 1 [Source:HGNC Symbol;Acc:HGNC:4249]	K00804	GGPS1
ENSP00000417806.1	small integral membrane protein 4 [Source:HGNC Symbol;Acc:HGNC:37257]		
ENSP00000292907.3	cytochrome c oxidase subunit 7A1 [Source:HGNC Symbol;Acc:HGNC:2287]	K02270	COX7A
ENSP00000300131.3	NGFI-A binding protein 2 [Source:HGNC Symbol;Acc:HGNC:7627]		
ENSP00000352138.6	kirre like nephrin family adhesion molecule 1 [Source:HGNC Symbol;Acc:HGNC:15734]		
ENSP00000434070.1	eukaryotic translation elongation factor 1 delta [Source:HGNC Symbol;Acc:HGNC:3211]	K15410	EEF1D
ENSP00000261890.2	RAB11A, member RAS oncogene family [Source:HGNC Symbol;Acc:HGNC:9760]	K07904	RAB11A
ENSP00000323068.4	transmembrane protein 168 [Source:HGNC Symbol;Acc:HGNC:25826]		
ENSP00000417573.1	Yip1 domain family member 6 [Source:HGNC Symbol;Acc:HGNC:28304]		
ENSP00000012443.4	protein phosphatase 5 catalytic subunit [Source:HGNC Symbol;Acc:HGNC:9322]	K04460	PPP5C
ENSP00000353408.5	moesin [Source:HGNC Symbol;Acc:HGNC:7373]	K05763	MSN
ENSP00000290299.2	ATP synthase peripheral stalk subunit OSCP [Source:HGNC Symbol;Acc:HGNC:850]	K02137	ATPeF0O, ATP5O, ATP
ENSP00000225614.1	galactokinase 1 [Source:HGNC Symbol;Acc:HGNC:4118]	K00849	galK
ENSP00000221233.3	exosome component 5 [Source:HGNC Symbol;Acc:HGNC:24662]	K12590	RRP46, EXOSC5
ENSP00000320236.2	translocase of inner mitochondrial membrane 22 [Source:HGNC Symbol;Acc:HGNC:17317]	K17790	TIM22
ENSP00000331897.3	isocitrate dehydrogenase (NADP(+)) 2, mitochondrial [Source:HGNC Symbol;Acc:HGNC:5383]	K00031	IDH1, IDH2, icd
ENSP00000364765.3	layilin [Source:HGNC Symbol;Acc:HGNC:29471]	K10077	LAYN
ENSP00000440045.2	ATPase sarcoplasmic/endoplasmic reticulum Ca2+ transporting 2 [Source:HGNC Symbol;Acc:H	K05853	ATP2A
ENSP00000229239.5	glyceraldehyde-3-phosphate dehydrogenase [Source:HGNC Symbol;Acc:HGNC:4141]	K00134	GAPDH, gapA
ENSP00000498365.1	TNF alpha induced protein 8 like 3 [Source:HGNC Symbol;Acc:HGNC:20620]		
ENSP00000360017.4	zinc finger protein 280C [Source:HGNC Symbol;Acc:HGNC:25955]		
ENSP00000264710.4	RAB10, member RAS oncogene family [Source:HGNC Symbol;Acc:HGNC:9759]	K07903	RAB10
ENSP00000299492.4	PPFIA binding protein 2 [Source:HGNC Symbol;Acc:HGNC:9250]		
ENSP00000231887.3	enoyl-CoA hydratase and 3-hydroxyacyl CoA dehydrogenase [Source:HGNC Symbol;Acc:HGN	K07514	EHHADH
ENSP00000444565.1	transmembrane and ubiquitin like domain containing 2 [Source:HGNC Symbol;Acc:HGNC:28459]		
ENSP00000398076.2	ATPase phospholipid transporting 9B (putative) [Source:HGNC Symbol;Acc:HGNC:13541]	K01530	E3.6.3.1
ENSP00000265062.3	RAB7A, member RAS oncogene family [Source:HGNC Symbol;Acc:HGNC:9788]	K07897	RAB7A
ENSP00000320508.5	ring finger protein 185 [Source:HGNC Symbol;Acc:HGNC:26783]	K10666	RNF5
ENSP00000497532.1	solute carrier family 25 member 38 [Source:HGNC Symbol;Acc:HGNC:26054]	K15118	SLC25A38
ENSP00000253719.1	napsin A aspartic peptidase [Source:HGNC Symbol;Acc:HGNC:13395]	K08565	NAPSA
ENSP00000366945.2	solute carrier family 25 member 51 [Source:HGNC Symbol;Acc:HGNC:23323]		
ENSP00000355210.3	transmembrane protein 184B [Source:HGNC Symbol;Acc:HGNC:1310]		
ENSP00000340672.4	armadillo repeat containing X-linked 3 [Source:HGNC Symbol;Acc:HGNC:24065]		
ENSP00000169551.6	translocase of inner mitochondrial membrane 21 [Source:HGNC Symbol;Acc:HGNC:25010]	K17796	TIM21

ENSP00000303315.4	JunB proto-oncogene, AP-1 transcription factor subunit [Source:HGNC Symbol;Acc:HGNC:620	K09028	JUNB
ENSP00000380178.2	ubiquitin conjugating enzyme E2 G1 [Source:HGNC Symbol;Acc:HGNC:12482]	K10575	UBE2G1, UBC7
ENSP00000252699.2	actinin alpha 4 [Source:HGNC Symbol;Acc:HGNC:166]	K05699	ACTN1_4
ENSP00000455744.1	novel PSMA2 and C7orf25 readthrough	K02726	PSMA2
ENSP00000360125.3	phosphoglucomutase 1 [Source:HGNC Symbol;Acc:HGNC:8905]	K01835	pgm
ENSP00000315630.6	switching B cell complex subunit SWAP70 [Source:HGNC Symbol;Acc:HGNC:17070]	K20072	SWAP70
ENSP00000157600.3	LIM and cysteine rich domains 1 [Source:HGNC Symbol;Acc:HGNC:6633]		
ENSP00000243997.3	ATP synthase F1 subunit epsilon [Source:HGNC Symbol;Acc:HGNC:838]	K02135	ATPeF1E, ATP5E, ATP1
ENSP00000421096.1	phosphoribosylaminoimidazole carboxylase and phosphoribosylaminoimidazolesuccinocarbox	K01587	PAICS
ENSP00000368219.3	nudix hydrolase 5 [Source:HGNC Symbol;Acc:HGNC:8052]		
ENSP00000243346.5	N-myc and STAT interactor [Source:HGNC Symbol;Acc:HGNC:7854]		
ENSP00000288245.9	high mobility group AT-hook 1 [Source:HGNC Symbol;Acc:HGNC:5010]		
ENSP00000284116.4	glycerophosphodiester phosphodiesterase domain containing 1 [Source:HGNC Symbol;Acc:HGNC:20883]		
ENSP00000294785.5	nicastrin [Source:HGNC Symbol;Acc:HGNC:17091]	K06171	NCSTN
ENSP00000304102.3	COP9 signalosome subunit 6 [Source:HGNC Symbol;Acc:HGNC:21749]	K12179	COPS6, CSN6
ENSP00000312318.3	zinc ribbon domain containing 2 [Source:HGNC Symbol;Acc:HGNC:11328]		
ENSP00000304051.3	ring finger protein 139 [Source:HGNC Symbol;Acc:HGNC:17023]	K15703	RNF139, TRC8
ENSP00000364494.1	apolipoprotein C3 [Source:HGNC Symbol;Acc:HGNC:610]	K08759	APOC3
ENSP00000407233.2	proteasome subunit beta 9 [Source:HGNC Symbol;Acc:HGNC:9546]	K02741	PSMB9, LMP2
ENSP00000296786.6	ubiquitin like domain containing CTD phosphatase 1 [Source:HGNC Symbol;Acc:HGNC:28110]	K17618	UBLCP1
ENSP00000337834.4	tumor protein p53 inducible protein 3 [Source:HGNC Symbol;Acc:HGNC:19373]	K10133	TP53I3
ENSP00000374455.4	sequestosome 1 [Source:HGNC Symbol;Acc:HGNC:11280]	K14381	SQSTM1
ENSP00000238044.3	ECRG4 augurin precursor [Source:HGNC Symbol;Acc:HGNC:24642]		
ENSP00000248564.4	G protein subunit gamma 11 [Source:HGNC Symbol;Acc:HGNC:4403]	K04546	GNG11
ENSP00000362334.3	proteasome subunit beta 2 [Source:HGNC Symbol;Acc:HGNC:9539]	K02734	PSMB2
ENSP00000362300.4	argonaute RISC component 1 [Source:HGNC Symbol;Acc:HGNC:3262]	K11593	ELF2C, AGO
ENSP00000331544.6	fibulin 1 [Source:HGNC Symbol;Acc:HGNC:3600]	K17307	FBLN1_2
ENSP00000376800.3	myotrophin [Source:HGNC Symbol;Acc:HGNC:15667]		
ENSP00000355228.5	high mobility group nucleosomal binding domain 2 [Source:HGNC Symbol;Acc:HGNC:4986]	K11300	HMGN2
ENSP00000216121.7	nipsnap homolog 1 [Source:HGNC Symbol;Acc:HGNC:7827]		
ENSP00000231004.4	lysyl oxidase [Source:HGNC Symbol;Acc:HGNC:6664]	K00277	LOX
ENSP00000360464.3	NFKB activating protein [Source:HGNC Symbol;Acc:HGNC:29873]		
ENSP00000383365.1	thioredoxin reductase 2 [Source:HGNC Symbol;Acc:HGNC:18155]	K00384	trxB
ENSP00000225964.5	collagen type I alpha 1 chain [Source:HGNC Symbol;Acc:HGNC:2197]	K06236	COL1A
ENSP00000461945.2	RAB31, member RAS oncogene family [Source:HGNC Symbol;Acc:HGNC:9771]	K07891	RAB22
ENSP00000298556.7	hypoxanthine phosphoribosyltransferase 1 [Source:HGNC Symbol;Acc:HGNC:5157]	K00760	hprT, hpt, HPRT1
ENSP00000484175.1	major facilitator superfamily domain containing 1 [Source:HGNC Symbol;Acc:HGNC:25874]		
ENSP00000361083.3	adipogenesis regulatory factor [Source:HGNC Symbol;Acc:HGNC:24043]		

ENSP00000359958.3	zinc finger RANBP2-type containing 2 [Source:HGNC Symbol;Acc:HGNC:13058]		
ENSP00000264080.5	G protein-coupled receptor 108 [Source:HGNC Symbol;Acc:HGNC:17829]		
ENSP00000404049.2	dermatan sulfate epimerase [Source:HGNC Symbol;Acc:HGNC:21144]	K01794	DSE
ENSP00000330918.7	phosphoglycolate phosphatase [Source:HGNC Symbol;Acc:HGNC:8909]	K19269	PGP, PGLP
ENSP00000352584.4	aldo-keto reductase family 1 member B10 [Source:HGNC Symbol;Acc:HGNC:382]	K00011	AKR1B
ENSP00000387858.2	stathmin 1 [Source:HGNC Symbol;Acc:HGNC:6510]	K04381	STMN1
ENSP00000265080.4	Ras protein specific guanine nucleotide releasing factor 2 [Source:HGNC Symbol;Acc:HGNC:98	K12326	RASGRF2
ENSP00000351181.5	glutathione S-transferase kappa 1 [Source:HGNC Symbol;Acc:HGNC:16906]	K13299	GSTK1
ENSP00000251595.6	hemoglobin subunit alpha 2 [Source:HGNC Symbol;Acc:HGNC:4824]	K13822	HBA
ENSP00000347619.4	major facilitator superfamily domain containing 10 [Source:HGNC Symbol;Acc:HGNC:16894]		
ENSP00000449751.1	FKBP prolyl isomerase 11 [Source:HGNC Symbol;Acc:HGNC:18624]	K09576	FKBP11
ENSP00000252486.3	apolipoprotein E [Source:HGNC Symbol;Acc:HGNC:613]		
ENSP00000321455.5	mitochondrial calcium uptake family member 3 [Source:HGNC Symbol;Acc:HGNC:27820]		
ENSP00000340466.4	glucosidase II alpha subunit [Source:HGNC Symbol;Acc:HGNC:4138]		
ENSP00000266085.5	TIMP metallopeptidase inhibitor 3 [Source:HGNC Symbol;Acc:HGNC:11822]	K16866	TIMP3
ENSP00000363641.5	thioredoxin [Source:HGNC Symbol;Acc:HGNC:12435]	K03671	trxA
ENSP00000364613.2	caspase recruitment domain family member 19 [Source:HGNC Symbol;Acc:HGNC:28148]		
ENSP00000460885.1	CORO7-PAM16 readthrough [Source:HGNC Symbol;Acc:HGNC:44424]	K17805;	PAM16, TIM16;CORO7
ENSP00000267750.4	ER membrane protein complex subunit 4 [Source:HGNC Symbol;Acc:HGNC:28032]		
ENSP00000368831.2	sorting nexin 2 [Source:HGNC Symbol;Acc:HGNC:11173]	K17917	SNX1_2
ENSP00000381693.2	zinc finger SWIM-type containing 8 [Source:HGNC Symbol;Acc:HGNC:23528]		
ENSP00000357702.3	S100 calcium binding protein A3 [Source:HGNC Symbol;Acc:HGNC:10493]		
ENSP00000382239.2	RNA binding motif protein 12B [Source:HGNC Symbol;Acc:HGNC:32310]		
ENSP00000301244.5	serine peptidase inhibitor, Kunitz type 2 [Source:HGNC Symbol;Acc:HGNC:11247]		
ENSP00000420641.1	signal sequence receptor subunit 3 [Source:HGNC Symbol;Acc:HGNC:11325]		
ENSP00000443442.1	PPFIA binding protein 1 [Source:HGNC Symbol;Acc:HGNC:9249]		
ENSP00000362409.3	niban apoptosis regulator 2 [Source:HGNC Symbol;Acc:HGNC:25282]		
ENSP00000303570.2	ankyrin repeat and MYND domain containing 2 [Source:HGNC Symbol;Acc:HGNC:25370]		
ENSP00000370194.4	GDP-mannose 4,6-dehydratase [Source:HGNC Symbol;Acc:HGNC:4369]	K01711	gmd, GMDS
ENSP00000415998.2	solute carrier family 48 member 1 [Source:HGNC Symbol;Acc:HGNC:26035]	K15380	SLC48A1, HRG1
ENSP00000471857.1	neuron navigator 1 [Source:HGNC Symbol;Acc:HGNC:15989]		
ENSP00000450627.1	thymopoietin [Source:HGNC Symbol;Acc:HGNC:11875]		
ENSP00000206423.3	coiled-coil domain containing 80 [Source:HGNC Symbol;Acc:HGNC:30649]		
ENSP00000490083.1	ATPase H+ transporting accessory protein 2 [Source:HGNC Symbol;Acc:HGNC:18305]	K19514	ATP6AP2, PRR
ENSP00000265056.7	minichromosome maintenance complex component 2 [Source:HGNC Symbol;Acc:HGNC:6944]	K02540	MCM2
ENSP00000308546.2	methylphosphate capping enzyme [Source:HGNC Symbol;Acc:HGNC:20247]	K15190	MEPCE, BCDIN3
ENSP00000446057.2	SUMO peptidase family member, NEDD8 specific [Source:HGNC Symbol;Acc:HGNC:22992]	K08597	SENP8, NEDP1, DEN1
ENSP00000317232.4	FKBP prolyl isomerase 10 [Source:HGNC Symbol;Acc:HGNC:18169]	K09575	FKBP9_10

ENSP00000341483.5	RAN binding protein 3 [Source:HGNC Symbol;Acc:HGNC:9850]	K15304	RANBP3
ENSP00000296424.4	3-hydroxybutyrate dehydrogenase 2 [Source:HGNC Symbol;Acc:HGNC:32389]	K00019	E1.1.1.30, bdh
ENSP00000286692.4	DNA damage regulated autophagy modulator 2 [Source:HGNC Symbol;Acc:HGNC:28769]		
ENSP00000306397.3	ubiquinol-cytochrome c reductase, Rieske iron-sulfur polypeptide 1 [Source:HGNC Symbol;Ac	K00411	UQCRFS1, RIP1, petA
ENSP00000321195.5	ATPase phospholipid transporting 11B (putative) [Source:HGNC Symbol;Acc:HGNC:13553]	K01530	E3.6.3.1
ENSP00000351737.6	transmembrane protein 126B [Source:HGNC Symbol;Acc:HGNC:30883]	K18165	TMEM126B
ENSP00000457788.1	mannosidase alpha class 2C member 1 [Source:HGNC Symbol;Acc:HGNC:6827]		
ENSP00000290573.2	hexokinase 2 [Source:HGNC Symbol;Acc:HGNC:4923]	K00844	HK
ENSP00000263038.4	phytanoyl-CoA 2-hydroxylase [Source:HGNC Symbol;Acc:HGNC:8940]	K00477	PHYH
ENSP00000357448.3	PBX homeobox interacting protein 1 [Source:HGNC Symbol;Acc:HGNC:21199]		
ENSP00000354499.2	mitochondrially encoded cytochrome c oxidase I [Source:HGNC Symbol;Acc:HGNC:7419]	K02256	COX1
ENSP00000349575.2	MOB kinase activator 3A [Source:HGNC Symbol;Acc:HGNC:29802]		
ENSP00000346032.3	annexin A2 [Source:HGNC Symbol;Acc:HGNC:537]	K17092	ANXA2
ENSP00000437109.1	FYVE, RhoGEF and PH domain containing 4 [Source:HGNC Symbol;Acc:HGNC:19125]	K05723	FGD4
ENSP00000403817.1	nascent polypeptide associated complex subunit alpha [Source:HGNC Symbol;Acc:HGNC:7629]		
ENSP00000478061.1	myristoylated alanine rich protein kinase C substrate [Source:HGNC Symbol;Acc:HGNC:6759]	K12561	MARCKS
ENSP00000364709.3	coagulation factor X [Source:HGNC Symbol;Acc:HGNC:3528]	K01314	F10
ENSP00000359233.4	ATP binding cassette subfamily D member 3 [Source:HGNC Symbol;Acc:HGNC:67]	K05677	ABCD3, PMP70
ENSP00000260731.3	kinesin family member 11 [Source:HGNC Symbol;Acc:HGNC:6388]	K10398	KIF11, EG5
ENSP00000356953.2	succinate dehydrogenase complex subunit C [Source:HGNC Symbol;Acc:HGNC:10682]	K00236	SDHC, SDH3
ENSP00000274276.3	oncostatin M receptor [Source:HGNC Symbol;Acc:HGNC:8507]	K05057	OSMR
ENSP00000235345.5	solute carrier family 35 member D1 [Source:HGNC Symbol;Acc:HGNC:20800]	K15281	SLC35D
ENSP00000371798.3	fascin actin-bundling protein 1 [Source:HGNC Symbol;Acc:HGNC:11148]	K17455	FSCN1_2
ENSP00000381220.1	HD domain containing 2 [Source:HGNC Symbol;Acc:HGNC:21078]	K07023	K07023
ENSP00000425721.1	calpastatin [Source:HGNC Symbol;Acc:HGNC:1515]	K04281	CAST
ENSP00000363054.4	syntaxin 12 [Source:HGNC Symbol;Acc:HGNC:11430]	K13813	STX12, STX13
ENSP00000249700.4	tropomodulin 2 [Source:HGNC Symbol;Acc:HGNC:11872]	K10370	TMOD
ENSP00000264689.6	UFM1 specific peptidase 2 [Source:HGNC Symbol;Acc:HGNC:25640]	K01376	UFSP2
ENSP00000184266.2	NADH:ubiquinone oxidoreductase subunit B4 [Source:HGNC Symbol;Acc:HGNC:7699]	K03960	NDUFB4
ENSP00000381859.3	translocase of outer mitochondrial membrane 6 [Source:HGNC Symbol;Acc:HGNC:34528]	K17772	TOM6
ENSP00000338788.6	zinc finger CCCH-type containing 15 [Source:HGNC Symbol;Acc:HGNC:29528]		
ENSP00000484343.1	serpin family B member 6 [Source:HGNC Symbol;Acc:HGNC:8950]	K13963	SERPINB
ENSP00000264896.2	scavenger receptor class B member 2 [Source:HGNC Symbol;Acc:HGNC:1665]	K12384	SCARB2, LIMP2, CD36L
ENSP00000350893.2	CD46 molecule [Source:HGNC Symbol;Acc:HGNC:6953]	K04007	CD46, MCP
ENSP00000229314.4	golgi transport 1B [Source:HGNC Symbol;Acc:HGNC:20175]		
ENSP00000477077.1	PC4 and SFRS1 interacting protein 1 [Source:HGNC Symbol;Acc:HGNC:9527]		
ENSP00000419851.1	guanine monophosphate synthase [Source:HGNC Symbol;Acc:HGNC:4378]	K01951	guaA, GMPS
ENSP00000358777.2	ATPase H+ transporting accessory protein 1 [Source:HGNC Symbol;Acc:HGNC:868]	K03662	ATPeVS1, ATP6S1

ENSP00000427976.1	peptidylprolyl isomerase A [Source:HGNC Symbol;Acc:HGNC:9253]	K03767	PPIA
ENSP00000336775.7	synemin [Source:HGNC Symbol;Acc:HGNC:24466]	K10376	DMN
ENSP00000253815.3	ubiquitin conjugating enzyme E2 D2 [Source:HGNC Symbol;Acc:HGNC:12475]		
ENSP00000442464.1	neutral cholesterol ester hydrolase 1 [Source:HGNC Symbol;Acc:HGNC:29260]		
ENSP00000379457.2	Fas associated factor 1 [Source:HGNC Symbol;Acc:HGNC:3578]	K20703	FAF1
ENSP00000367721.2	nucleoporin 160 [Source:HGNC Symbol;Acc:HGNC:18017]	K14303	NUP160, NUP120
ENSP00000332790.3	histone cluster 2 H2A family member b [Source:HGNC Symbol;Acc:HGNC:20508]	K11251	H2A
ENSP00000344937.4	dpy-19 like C-mannosyltransferase 3 [Source:HGNC Symbol;Acc:HGNC:27120]		
ENSP00000358151.2	histone cluster 2 H2B family member e [Source:HGNC Symbol;Acc:HGNC:4760]	K11252	H2B
ENSP00000385740.1	Sad1 and UNC84 domain containing 2 [Source:HGNC Symbol;Acc:HGNC:14210]	K19347	SUN1_2
ENSP00000253794.1	vacuolar protein sorting 25 homolog [Source:HGNC Symbol;Acc:HGNC:28122]	K12189	VPS25, EAP20
ENSP00000372582.2	major histocompatibility complex, class II, DP beta 1 [Source:HGNC Symbol;Acc:HGNC:4940]		
ENSP00000368965.4	heparan-alpha-glucosaminide N-acetyltransferase [Source:HGNC Symbol;Acc:HGNC:26527]	K10532	HGSNAT
ENSP00000303057.4	peptidylprolyl isomerase C [Source:HGNC Symbol;Acc:HGNC:9256]	K09563	PPIC, CYPC
ENSP00000468578.1	microfibril associated protein 4 [Source:HGNC Symbol;Acc:HGNC:7035]		
ENSP00000466214.1	programmed cell death 5 [Source:HGNC Symbol;Acc:HGNC:8764]	K06875	PDCD5, TFAR19
ENSP00000420381.1	calumenin [Source:HGNC Symbol;Acc:HGNC:1458]		
ENSP00000420389.1	membrane metalloendopeptidase [Source:HGNC Symbol;Acc:HGNC:7154]	K01389	MME, CD10
ENSP00000498864.1	pyruvate dehydrogenase kinase 3 [Source:HGNC Symbol;Acc:HGNC:8811]	K00898	PDK2_3_4
ENSP00000281456.5	solute carrier family 25 member 4 [Source:HGNC Symbol;Acc:HGNC:10990]	K05863	SLC25A4S, ANT
ENSP00000362092.3	Ras related GTP binding C [Source:HGNC Symbol;Acc:HGNC:19902]	K16186	RRAGC_D
ENSP00000265641.4	carnitine palmitoyltransferase 1A [Source:HGNC Symbol;Acc:HGNC:2328]	K08765	CPT1A
ENSP00000378360.3	triokinase and FMN cyclase [Source:HGNC Symbol;Acc:HGNC:24552]	K00863	DAK, TKFC
ENSP00000383960.3	5'-nucleotidase, cytosolic II [Source:HGNC Symbol;Acc:HGNC:8022]	K01081	E3.1.3.5
ENSP00000296603.4	LMBR1 domain containing 2 [Source:HGNC Symbol;Acc:HGNC:25287]		
ENSP00000383003.4	FKBP prolyl isomerase 1A [Source:HGNC Symbol;Acc:HGNC:3711]	K09568	FKBP1
ENSP00000246548.3	ubiquitin like modifier activating enzyme 2 [Source:HGNC Symbol;Acc:HGNC:30661]	K10685	UBLE1B, SAE2, UBA2
ENSP00000356584.3	torsin 1A interacting protein 2 [Source:HGNC Symbol;Acc:HGNC:24055]		
ENSP00000384164.1	kinesin family member 16B [Source:HGNC Symbol;Acc:HGNC:15869]	K17916	KIF16B, SNX23
ENSP00000498303.1	glutathione synthetase [Source:HGNC Symbol;Acc:HGNC:4624]	K21456	GSS
ENSP00000182527.3	translocation associated membrane protein 2 [Source:HGNC Symbol;Acc:HGNC:16855]		
ENSP00000416293.2	solute carrier family 2 member 1 [Source:HGNC Symbol;Acc:HGNC:11005]	K07299	SLC2A1, GLUT1
ENSP00000497458.1	vitamin K epoxide reductase complex subunit 1 like 1 [Source:HGNC Symbol;Acc:HGNC:21492	K05357	VKORC1
ENSP00000261875.5	3-hydroxyacyl-CoA dehydratase 3 [Source:HGNC Symbol;Acc:HGNC:24175]	K10703	PHS1, PAS2
ENSP00000353720.3	carboxylesterase 1 [Source:HGNC Symbol;Acc:HGNC:1863]	K01044	CES1
ENSP00000226444.3	sulfotransferase family 1E member 1 [Source:HGNC Symbol;Acc:HGNC:11377]	K01016	SULT1E1, STE
ENSP00000247194.4	trans-L-3-hydroxyproline dehydratase [Source:HGNC Symbol;Acc:HGNC:20488]	K18384	L3HYPDH
ENSP00000356918.1	syntaxin 7 [Source:HGNC Symbol;Acc:HGNC:11442]	K08488	STX7

ENSP00000216727.4	poly(A) binding protein nuclear 1 [Source:HGNC Symbol;Acc:HGNC:8565]	K14396	PABPN1, PABP2
ENSP00000448286.1	methyltransferase like 7A [Source:HGNC Symbol;Acc:HGNC:24550]		
ENSP00000363071.3	desmin [Source:HGNC Symbol;Acc:HGNC:2770]	K07610	DES
ENSP00000417052.1	EBP cholestenol delta-isomerase [Source:HGNC Symbol;Acc:HGNC:3133]	K01824	EBP
ENSP00000386893.2	peptidylprolyl isomerase like 3 [Source:HGNC Symbol;Acc:HGNC:9262]	K12734	PPIL3
ENSP00000355994.3	hydroxysteroid 11-beta dehydrogenase 1 [Source:HGNC Symbol;Acc:HGNC:5208]	K15680	HSD11B1
ENSP00000462878.1	F-box and leucine rich repeat protein 20 [Source:HGNC Symbol;Acc:HGNC:24679]	K10268	FBXL2_20
ENSP00000401191.1	BUD23 rRNA methyltransferase and ribosome maturation factor [Source:HGNC Symbol;Acc:HGNC:16405]		
ENSP00000442284.2	metabolism of cobalamin associated A [Source:HGNC Symbol;Acc:HGNC:18871]	K07588	argK
ENSP00000357704.1	S100 calcium binding protein A4 [Source:HGNC Symbol;Acc:HGNC:10494]		
ENSP00000257347.4	cysteinyl-tRNA synthetase 2, mitochondrial [Source:HGNC Symbol;Acc:HGNC:25695]	K01883	CARS, cysS
ENSP00000494352.1	3-ketodihydrosphingosine reductase [Source:HGNC Symbol;Acc:HGNC:4021]	K04708	E1.1.1.102
ENSP00000468656.1	ubiquitin like 5 [Source:HGNC Symbol;Acc:HGNC:13736]	K13113	UBL5, HUB1
ENSP00000243563.2	small nuclear ribonucleoprotein polypeptide A [Source:HGNC Symbol;Acc:HGNC:11151]	K11091	SNRPA
ENSP00000302961.2	heat shock protein family A (Hsp70) member 4 [Source:HGNC Symbol;Acc:HGNC:5237]	K09489	HSPA4
ENSP00000276390.2	ATPase H+ transporting V1 subunit B2 [Source:HGNC Symbol;Acc:HGNC:854]	K02147	ATPeV1B, ATP6B
ENSP00000438115.1	COP9 signalosome subunit 7A [Source:HGNC Symbol;Acc:HGNC:16758]	K12180	COPS7, CSN7
ENSP00000401564.2	RAN binding protein 1 [Source:HGNC Symbol;Acc:HGNC:9847]	K15306	RANBP1
ENSP00000324729.4	salvador family WW domain containing protein 1 [Source:HGNC Symbol;Acc:HGNC:17795]	K16686	SAV1, Sav
ENSP00000327336.4	biglycan [Source:HGNC Symbol;Acc:HGNC:1044]	K08118	BGN
ENSP00000367044.1	ribosome binding protein 1 [Source:HGNC Symbol;Acc:HGNC:10448]		
ENSP00000343471.2	IKBKB interacting protein [Source:HGNC Symbol;Acc:HGNC:26430]		
ENSP00000265686.3	T cell immune regulator 1, ATPase H+ transporting V0 subunit a3 [Source:HGNC Symbol;Acc:H	K02154	ATPeV0A, ATP6N
ENSP00000376161.3	ORMDL sphingolipid biosynthesis regulator 1 [Source:HGNC Symbol;Acc:HGNC:16036]		
ENSP00000451932.1	NME1-NME2 readthrough [Source:HGNC Symbol;Acc:HGNC:33531]	K00940	ndk, NME
ENSP00000361626.3	Y-box binding protein 1 [Source:HGNC Symbol;Acc:HGNC:8014]	K09276	YBX1, NSEP1
ENSP00000361642.3	phosphopantothenoylcysteine synthetase [Source:HGNC Symbol;Acc:HGNC:25686]	K01922	PPCS, coaB
ENSP00000346809.3	post-GPI attachment to proteins 1 [Source:HGNC Symbol;Acc:HGNC:25712]	K05294	PGAP1
ENSP00000473631.1	DnaJ heat shock protein family (Hsp40) member C3 [Source:HGNC Symbol;Acc:HGNC:9439]	K09523	DNAJC3
ENSP00000480515.1	heme oxygenase 2 [Source:HGNC Symbol;Acc:HGNC:5014]	K21418	HMOX2
ENSP00000438873.1	enolase 2 [Source:HGNC Symbol;Acc:HGNC:3353]	K01689	ENO, eno
ENSP00000371740.1	KN motif and ankyrin repeat domains 1 [Source:HGNC Symbol;Acc:HGNC:19309]		
ENSP00000297350.4	TNF receptor superfamily member 11b [Source:HGNC Symbol;Acc:HGNC:11909]	K05148	TNFRSF11B, OPG
ENSP00000359170.4	deleted in primary ciliary dyskinesia homolog (mouse) [Source:HGNC Symbol;Acc:HGNC:2454	K20800	DPCD
ENSP00000325448.5	lysyl-tRNA synthetase [Source:HGNC Symbol;Acc:HGNC:6215]	K04567	KARS, lysS
ENSP00000379217.3	ubiquitin family domain containing 1 [Source:HGNC Symbol;Acc:HGNC:30565]		
ENSP00000337040.3	unc-119 lipid binding chaperone [Source:HGNC Symbol;Acc:HGNC:12565]		
ENSP00000354649.5	magnesium transporter 1 [Source:HGNC Symbol;Acc:HGNC:28880]	K19478	MAGT1

ENSP00000380820.1	minichromosome maintenance complex component 3 associated protein [Source:HGNC Symbol;Acc:HGNC:6946]		
ENSP00000340969.5	testis expressed 264, ER-phagy receptor [Source:HGNC Symbol;Acc:HGNC:30247]		
ENSP00000370150.4	WRN helicase interacting protein 1 [Source:HGNC Symbol;Acc:HGNC:20876]	K07478	ycaJ
ENSP00000366240.2	abhydrolase domain containing 17B [Source:HGNC Symbol;Acc:HGNC:24278]		
ENSP00000342385.4	prostaglandin E synthase [Source:HGNC Symbol;Acc:HGNC:9599]	K15729	PTGES
ENSP00000406061.2	spartin [Source:HGNC Symbol;Acc:HGNC:18514]	K19366	SPG20
ENSP00000301329.6	glyoxalase domain containing 4 [Source:HGNC Symbol;Acc:HGNC:14111]		
ENSP00000250124.6	mannose-P-dolichol utilization defect 1 [Source:HGNC Symbol;Acc:HGNC:7207]	K09660	MPDU1
ENSP00000217133.1	tubulin beta 1 class VI [Source:HGNC Symbol;Acc:HGNC:16257]	K07375	TUBB
ENSP00000345494.4	phospholipid scramblase 1 [Source:HGNC Symbol;Acc:HGNC:9092]		
ENSP00000359225.4	calponin 3 [Source:HGNC Symbol;Acc:HGNC:2157]		
ENSP00000247020.3	stromal cell derived factor 2 [Source:HGNC Symbol;Acc:HGNC:10675]		
ENSP00000299608.2	thioredoxin related transmembrane protein 3 [Source:HGNC Symbol;Acc:HGNC:24718]	K09585	TXNDC10
ENSP00000414359.2	proteasome subunit alpha 1 [Source:HGNC Symbol;Acc:HGNC:9530]	K02725	PSMA1
ENSP00000268058.3	promyelocytic leukemia [Source:HGNC Symbol;Acc:HGNC:9113]		
ENSP00000305069.4	transmembrane protein 192 [Source:HGNC Symbol;Acc:HGNC:26775]		
ENSP00000362730.5	mitochondrial carrier 1 [Source:HGNC Symbol;Acc:HGNC:17586]	K17885	MTCH
ENSP00000345347.5	high mobility group box 1 [Source:HGNC Symbol;Acc:HGNC:4983]		
ENSP00000497574.1	glutamate-cysteine ligase catalytic subunit [Source:HGNC Symbol;Acc:HGNC:4311]	K11204	GCLC
ENSP00000335808.6	family with sequence similarity 192 member A [Source:HGNC Symbol;Acc:HGNC:29856]		
ENSP00000225737.6	A-kinase anchoring protein 10 [Source:HGNC Symbol;Acc:HGNC:368]	K16526	AKAP10
ENSP00000495240.1	chromodomain helicase DNA binding protein 8 [Source:HGNC Symbol;Acc:HGNC:20153]	K04494	CHD8, HELSNF1
ENSP00000290810.3	NEDD8 activating enzyme E1 subunit 1 [Source:HGNC Symbol;Acc:HGNC:621]	K04532	NAE1, APPBP1
ENSP00000403400.3	cell cycle progression 1 [Source:HGNC Symbol;Acc:HGNC:24227]		
ENSP00000387216.1	protein phosphatase 3 regulatory subunit B, alpha [Source:HGNC Symbol;Acc:HGNC:9317]	K06268	PPP3R, CNB
ENSP00000467600.1	synaptogyrin 2 [Source:HGNC Symbol;Acc:HGNC:11499]		
ENSP00000310170.2	FOS like 1, AP-1 transcription factor subunit [Source:HGNC Symbol;Acc:HGNC:13718]	K04502	FOSL1
ENSP00000215909.5	galectin 1 [Source:HGNC Symbol;Acc:HGNC:6561]	K06830	LGALS1
ENSP00000382021.3	leucine carboxyl methyltransferase 1 [Source:HGNC Symbol;Acc:HGNC:17557]	K18203	LCMT1
ENSP00000432280.1	TBC1 domain family member 20 [Source:HGNC Symbol;Acc:HGNC:16133]	K20372	TBC1D20, GYP8
ENSP00000354574.3	solute carrier family 9 member B2 [Source:HGNC Symbol;Acc:HGNC:25143]		
ENSP00000217446.3	phosphatidylinositol glycan anchor biosynthesis class U [Source:HGNC Symbol;Acc:HGNC:157	K05293	PIGU
ENSP00000303987.7	transmembrane protein 223 [Source:HGNC Symbol;Acc:HGNC:28464]		
ENSP00000364940.3	chloride intracellular channel 1 [Source:HGNC Symbol;Acc:HGNC:2062]	K05021	CLIC1
ENSP00000406878.2	proteasome subunit beta 8 [Source:HGNC Symbol;Acc:HGNC:9545]	K02740	PSMB8, LMP7
ENSP00000434024.1	H2A histone family member X [Source:HGNC Symbol;Acc:HGNC:4739]	K11251	H2A
ENSP00000386377.1	alpha-1,6-mannosylglycoprotein 6-beta-N-acetylglucosaminyltransferase [Source:HGNC Sym	K00744	MGAT5
ENSP00000265498.1	microsomal glutathione S-transferase 2 [Source:HGNC Symbol;Acc:HGNC:7063]	K00799	GST, gst

ENSP00000429083.1	tubulin folding cofactor A [Source:HGNC Symbol;Acc:HGNC:11579]	K17292	TBCA
ENSP00000342951.3	ATPase H+ transporting V0 subunit a1 [Source:HGNC Symbol;Acc:HGNC:865]	K02154	ATPeV0A, ATP6N
ENSP00000445929.1	solute carrier family 2 member 14 [Source:HGNC Symbol;Acc:HGNC:18301]		
ENSP00000419503.1	3-hydroxyacyl-CoA dehydratase 4 [Source:HGNC Symbol;Acc:HGNC:20920]	K10703	PHS1, PAS2
ENSP00000326342.3	ELMO domain containing 2 [Source:HGNC Symbol;Acc:HGNC:28111]		
ENSP00000337688.4	SEC62 homolog, preprotein translocation factor [Source:HGNC Symbol;Acc:HGNC:11846]	K12275	SEC62
ENSP00000318195.4	nucleolin [Source:HGNC Symbol;Acc:HGNC:7667]	K11294	NCL, NSR1
ENSP00000464522.3	translocase of inner mitochondrial membrane 23 [Source:HGNC Symbol;Acc:HGNC:17312]	K17794	TIM23
ENSP00000369853.3	C2 calcium dependent domain containing 2 [Source:HGNC Symbol;Acc:HGNC:1266]		
ENSP00000396127.3	RAN, member RAS oncogene family [Source:HGNC Symbol;Acc:HGNC:9846]	K07936	RAN
ENSP00000263383.3	ilvB acetolactate synthase like [Source:HGNC Symbol;Acc:HGNC:6041]	K11259	ILVBL
ENSP00000296824.2	coiled-coil domain containing 127 [Source:HGNC Symbol;Acc:HGNC:30520]		
ENSP00000472998.1	small nuclear ribonucleoprotein U1 subunit 70 [Source:HGNC Symbol;Acc:HGNC:11150]	K11093	SNRP70
ENSP00000312458.2	ARV1 homolog, fatty acid homeostasis modulator [Source:HGNC Symbol;Acc:HGNC:29561]		
ENSP00000269601.4	thioredoxin like 4A [Source:HGNC Symbol;Acc:HGNC:30551]	K12859	TXNL4A, DIB1
ENSP00000319673.9	G protein-coupled receptor 89A [Source:HGNC Symbol;Acc:HGNC:31984]		
ENSP00000261813.4	prefoldin subunit 1 [Source:HGNC Symbol;Acc:HGNC:8866]	K09548	PFDN1
ENSP00000478310.2	signal peptidase complex subunit 1 [Source:HGNC Symbol;Acc:HGNC:23401]	K12946	SPCS1
ENSP00000319141.4	cytochrome b reductase 1 [Source:HGNC Symbol;Acc:HGNC:20797]	K08370	CYBRD1, Dcytb
ENSP00000310668.5	nucleoporin 93 [Source:HGNC Symbol;Acc:HGNC:28958]	K14309	NUP93, NIC96
ENSP00000347041.4	fibromodulin [Source:HGNC Symbol;Acc:HGNC:3774]	K08121	FMOD
ENSP00000358633.2	glucose-6-phosphate dehydrogenase [Source:HGNC Symbol;Acc:HGNC:4057]		
ENSP00000431391.1	apoptosis inhibitor 5 [Source:HGNC Symbol;Acc:HGNC:594]		
ENSP00000498922.1	peptidase D [Source:HGNC Symbol;Acc:HGNC:8840]		
ENSP00000493302.1	pyridoxamine 5'-phosphate oxidase [Source:HGNC Symbol;Acc:HGNC:30260]	K00275	pdxH, PNPO
ENSP00000425882.1	NADH:ubiquinone oxidoreductase subunit C1 [Source:HGNC Symbol;Acc:HGNC:7705]	K03967	NDUFC1
ENSP00000366157.4	G protein-coupled receptor 180 [Source:HGNC Symbol;Acc:HGNC:28899]		
ENSP00000499458.1	sarcolemma associated protein [Source:HGNC Symbol;Acc:HGNC:16643]		
ENSP00000352516.3	DNA methyltransferase 1 [Source:HGNC Symbol;Acc:HGNC:2976]	K00558	DNMT1, dcm
ENSP00000339723.3	corepressor interacting with RBPJ, 1 [Source:HGNC Symbol;Acc:HGNC:24217]	K06066	CIR
ENSP00000237642.6	starch binding domain 1 [Source:HGNC Symbol;Acc:HGNC:24854]		
ENSP00000362682.3	olfactomedin like 2A [Source:HGNC Symbol;Acc:HGNC:27270]		
ENSP00000433999.1	ribonuclease/angiogenin inhibitor 1 [Source:HGNC Symbol;Acc:HGNC:10074]	K16634	RNH1
ENSP00000499472.1	steroid sulfatase [Source:HGNC Symbol;Acc:HGNC:11425]	K01131	STS
ENSP00000278483.3	heat shock protein nuclear import factor hikeshi [Source:HGNC Symbol;Acc:HGNC:26938]		
ENSP00000223215.4	mesoderm specific transcript [Source:HGNC Symbol;Acc:HGNC:7028]		
ENSP00000465432.1	chromosome 19 open reading frame 53 [Source:HGNC Symbol;Acc:HGNC:24991]		
ENSP00000314067.3	p21 (RAC1) activated kinase 2 [Source:HGNC Symbol;Acc:HGNC:8591]	K04410	PAK2

ENSP00000393507.1	methylthioadenosine phosphorylase [Source:HGNC Symbol;Acc:HGNC:7413]	
ENSP00000263579.4	decapping enzyme, scavenger [Source:HGNC Symbol;Acc:HGNC:29812]	K12584 DCPS, DCS
ENSP00000452697.1	SEC11 homolog A, signal peptidase complex subunit [Source:HGNC Symbol;Acc:HGNC:17718]	K13280 SEC11, sipW
ENSP00000367938.1	cytochrome c oxidase subunit 7A2 like [Source:HGNC Symbol;Acc:HGNC:2289]	K02270 COX7A
ENSP00000377374.3	endoplasmic reticulum-golgi intermediate compartment 1 [Source:HGNC Symbol;Acc:HGNC:2	K20365 ERGIC1
ENSP00000390475.1	CXXC finger protein 1 [Source:HGNC Symbol;Acc:HGNC:24343]	K14960 CXXC1, SPP1, CPS40
ENSP00000356954.3	cellular communication network factor 2 [Source:HGNC Symbol;Acc:HGNC:2500]	K06827 CTGF
ENSP00000407788.2	inner membrane mitochondrial protein [Source:HGNC Symbol;Acc:HGNC:6047]	K17785 IMMT, FCJ1, MNOS2
ENSP00000343899.5	FERM domain containing 6 [Source:HGNC Symbol;Acc:HGNC:19839]	K16822 FRMD6
ENSP00000363191.1	reactive oxygen species modulator 1 [Source:HGNC Symbol;Acc:HGNC:16185]	
ENSP00000348019.5	solute carrier family 17 member 5 [Source:HGNC Symbol;Acc:HGNC:10933]	K12301 SLC17A5
ENSP00000258761.3	basic leucine zipper and W2 domains 2 [Source:HGNC Symbol;Acc:HGNC:18808]	
ENSP00000419105.1	transgelin 3 [Source:HGNC Symbol;Acc:HGNC:29868]	K20526 TAGLN
ENSP00000259746.9	transmembrane protein 63B [Source:HGNC Symbol;Acc:HGNC:17735]	
ENSP00000261733.2	aldehyde dehydrogenase 2 family member [Source:HGNC Symbol;Acc:HGNC:404]	K00128 ALDH
ENSP00000354340.4	ubiquitin like modifier activating enzyme 3 [Source:HGNC Symbol;Acc:HGNC:12470]	K10686 UBE1C, UBA3
ENSP00000433919.1	chloride nucleotide-sensitive channel 1A [Source:HGNC Symbol;Acc:HGNC:2080]	K05019 CLNS1A
ENSP00000236957.5	eukaryotic translation elongation factor 1 beta 2 [Source:HGNC Symbol;Acc:HGNC:3208]	K03232 EEF1B
ENSP00000360671.4	solute carrier family 25 member 5 [Source:HGNC Symbol;Acc:HGNC:10991]	K05863 SLC25A4S, ANT
ENSP00000405730.2	apolipoprotein M [Source:HGNC Symbol;Acc:HGNC:13916]	
ENSP00000355467.3	saccharopine dehydrogenase (putative) [Source:HGNC Symbol;Acc:HGNC:24275]	
ENSP00000383178.3	diaphanous related formin 3 [Source:HGNC Symbol;Acc:HGNC:15480]	K05745 DIAPH3, DRF3
ENSP00000370750.4	retinol dehydrogenase 11 [Source:HGNC Symbol;Acc:HGNC:17964]	K11152 RDH11
ENSP00000421664.1	PDZ and LIM domain 7 [Source:HGNC Symbol;Acc:HGNC:22958]	
ENSP00000481625.1	solute carrier family 6 member 6 [Source:HGNC Symbol;Acc:HGNC:11052]	K05039 SLC6A6S
ENSP00000498538.1	DIMT1 rRNA methyltransferase and ribosome maturation factor [Source:HGNC Symbol;Acc:HG	K14191 DIM1
ENSP00000296978.3	transmembrane protein 200A [Source:HGNC Symbol;Acc:HGNC:21075]	
ENSP00000498361.1	transforming acidic coiled-coil containing protein 3 [Source:HGNC Symbol;Acc:HGNC:11524]	K14283 TACC3, maskin
ENSP00000498786.1	hydroxymethylbilane synthase [Source:HGNC Symbol;Acc:HGNC:4982]	K01749 hemC, HMBS
ENSP00000366672.3	D-aminoacyl-tRNA deacylase 1 [Source:HGNC Symbol;Acc:HGNC:16219]	K07560 dtd, DTD1
ENSP00000490312.1	EFR3 homolog A [Source:HGNC Symbol;Acc:HGNC:28970]	
ENSP00000383866.4	SUZ RNA binding domain containing 1 [Source:HGNC Symbol;Acc:HGNC:30232]	
ENSP00000356037.3	complement component 4 binding protein alpha [Source:HGNC Symbol;Acc:HGNC:1325]	K04002 C4BPA
ENSP00000382779.1	SEH1 like nucleoporin [Source:HGNC Symbol;Acc:HGNC:30379]	K14299 SEH1
ENSP00000275428.4	gamma-glutamylcyclotransferase [Source:HGNC Symbol;Acc:HGNC:21705]	K00682 GGCT
ENSP00000373153.5	3-hydroxyacyl-CoA dehydratase 2 [Source:HGNC Symbol;Acc:HGNC:9640]	K10703 PHS1, PAS2
ENSP00000413937.2	inositol polyphosphate-5-phosphatase K [Source:HGNC Symbol;Acc:HGNC:33882]	K01106 E3.1.3.56
ENSP00000266679.8	cleavage and polyadenylation specific factor 6 [Source:HGNC Symbol;Acc:HGNC:13871]	

ENSP00000393580.2	transporter 2, ATP binding cassette subfamily B member [Source:HGNC Symbol;Acc:HGNC:44]	K05654	ABCB3, TAP2
ENSP00000257910.3	tetraspanin 31 [Source:HGNC Symbol;Acc:HGNC:10539]	K17356	TSPAN13_31
ENSP00000362576.4	tyrosyl-tRNA synthetase [Source:HGNC Symbol;Acc:HGNC:12840]	K01866	YARS, tyrS
ENSP00000354201.5	ubiquitin conjugating enzyme E2 Z [Source:HGNC Symbol;Acc:HGNC:25847]	K10585	UBE2Z
ENSP00000499020.1	extended synaptotagmin 2 [Source:HGNC Symbol;Acc:HGNC:22211]		
ENSP00000358160.2	histone cluster 1 H3 family member h [Source:HGNC Symbol;Acc:HGNC:4775]	K11253	H3
ENSP00000346671.3	FYN proto-oncogene, Src family tyrosine kinase [Source:HGNC Symbol;Acc:HGNC:4037]	K05703	FYN
ENSP00000333298.4	lysosomal associated membrane protein 1 [Source:HGNC Symbol;Acc:HGNC:6499]	K06528	LAMP1_2, CD107
ENSP00000225740.6	aldehyde dehydrogenase 3 family member A1 [Source:HGNC Symbol;Acc:HGNC:405]	K00129	E1.2.1.5
ENSP00000403153.2	apolipoprotein L2 [Source:HGNC Symbol;Acc:HGNC:619]	K14480	APOL
ENSP00000339566.2	histone cluster 1 H1 family member c [Source:HGNC Symbol;Acc:HGNC:4716]	K11275	H1_5
ENSP00000356989.3	prefoldin subunit 2 [Source:HGNC Symbol;Acc:HGNC:8867]	K09549	PFDN2
ENSP00000365159.4	iron-sulfur cluster assembly 1 [Source:HGNC Symbol;Acc:HGNC:28660]	K13628	iscA, ISCA1
ENSP00000299198.2	creatine kinase B [Source:HGNC Symbol;Acc:HGNC:1991]	K00933	E2.7.3.2
ENSP00000357474.5	centrosomal protein 85 like [Source:HGNC Symbol;Acc:HGNC:21638]	K16766	CEP85
ENSP00000432441.1	prefoldin subunit 4 [Source:HGNC Symbol;Acc:HGNC:8868]	K09550	PFDN4
ENSP00000261623.3	cytochrome b-245 alpha chain [Source:HGNC Symbol;Acc:HGNC:2577]	K08009	CYBA, P22PHOX
ENSP00000413483.1	dephospho-CoA kinase domain containing [Source:HGNC Symbol;Acc:HGNC:26238]		
ENSP00000379203.3	ATPase H+ transporting V1 subunit C1 [Source:HGNC Symbol;Acc:HGNC:856]	K02148	ATPeV1C, ATP6C
ENSP00000378699.3	cyclin dependent kinase 1 [Source:HGNC Symbol;Acc:HGNC:1722]	K02087	CDK1, CDC2
ENSP00000305860.6	heterogeneous nuclear ribonucleoprotein D [Source:HGNC Symbol;Acc:HGNC:5036]		
ENSP00000320866.4	calreticulin [Source:HGNC Symbol;Acc:HGNC:1455]	K08057	CALR
ENSP00000349324.3	TNF receptor superfamily member 10c [Source:HGNC Symbol;Acc:HGNC:11906]	K04722	TNFRSF10, TRAILR, CD
ENSP00000433240.1	midkine [Source:HGNC Symbol;Acc:HGNC:6972]		
ENSP00000372005.2	DnaJ heat shock protein family (Hsp40) member C19 [Source:HGNC Symbol;Acc:HGNC:30528]	K09539	DNAJC19
ENSP00000261167.2	WW domain binding protein 11 [Source:HGNC Symbol;Acc:HGNC:16461]	K12866	WBP11, NPWBP
ENSP00000223122.2	core 1 synthase, glycoprotein-N-acetylgalactosamine 3-beta-galactosyltransferase 1 [Source:H	K00731	C1GALT1
ENSP00000230895.6	death associated protein [Source:HGNC Symbol;Acc:HGNC:2672]		
ENSP00000323811.5	2-hydroxyacyl-CoA lyase 1 [Source:HGNC Symbol;Acc:HGNC:17856]	K12261	HACL1
ENSP00000265523.4	biliverdin reductase A [Source:HGNC Symbol;Acc:HGNC:1062]	K00214	BLVRA, bvdR
ENSP00000303992.4	transmembrane protein 43 [Source:HGNC Symbol;Acc:HGNC:28472]		
ENSP00000352929.3	casein kinase 1 epsilon [Source:HGNC Symbol;Acc:HGNC:2453]	K08960	CSNK1E
ENSP00000259400.6	syntaxin 17 [Source:HGNC Symbol;Acc:HGNC:11432]	K08491	STX17
ENSP00000494440.1	ribosomal protein S10 [Source:HGNC Symbol;Acc:HGNC:10383]	K02947	RP-S10e, RPS10
ENSP00000248958.4	stromal cell derived factor 2 like 1 [Source:HGNC Symbol;Acc:HGNC:10676]		
ENSP00000387729.2	poly(A) binding protein interacting protein 1 [Source:HGNC Symbol;Acc:HGNC:16945]	K14322	PAIP1
ENSP00000378958.3	RAB, member of RAS oncogene family like 2B [Source:HGNC Symbol;Acc:HGNC:9800]		
ENSP00000425634.1	histidyl-tRNA synthetase [Source:HGNC Symbol;Acc:HGNC:4816]	K01892	HARS, hisS

ENSP00000280557.6	density regulated re-initiation and release factor [Source:HGNC Symbol;Acc:HGNC:2769]		
ENSP00000280871.4	solute carrier family 2 member 13 [Source:HGNC Symbol;Acc:HGNC:15956]	K08150	SLC2A13, ITR
ENSP00000366190.2	DR1 associated protein 1 [Source:HGNC Symbol;Acc:HGNC:3019]		
ENSP00000362480.1	VPS26, retromer complex component A [Source:HGNC Symbol;Acc:HGNC:12711]	K18466	VPS26
ENSP00000363944.3	LAS1 like, ribosome biogenesis factor [Source:HGNC Symbol;Acc:HGNC:25726]	K16912	LAS1
ENSP00000339017.4	ATPase family AAA domain containing 1 [Source:HGNC Symbol;Acc:HGNC:25903]		
ENSP00000361524.3	chromosome 9 open reading frame 78 [Source:HGNC Symbol;Acc:HGNC:24932]		
ENSP00000273853.6	centromere protein C [Source:HGNC Symbol;Acc:HGNC:1854]	K11497	CENPC
ENSP00000358022.2	MCL1 apoptosis regulator, BCL2 family member [Source:HGNC Symbol;Acc:HGNC:6943]	K02539	MCL1
ENSP00000273258.3	ADP ribosylation factor like GTPase 6 interacting protein 5 [Source:HGNC Symbol;Acc:HGNC:1	K20393	ARL6IP5, PRAF3
ENSP00000489597.1	protein tyrosine phosphatase non-receptor type 11 [Source:HGNC Symbol;Acc:HGNC:9644]	K07293	PTPN11
ENSP00000378938.2	zinc fingers and homeoboxes 1 [Source:HGNC Symbol;Acc:HGNC:12871]		
ENSP00000485216.1	mitoregulin [Source:HGNC Symbol;Acc:HGNC:27339]		
ENSP00000325240.6	LIM and SH3 protein 1 [Source:HGNC Symbol;Acc:HGNC:6513]		
ENSP00000342322.4	transmembrane 7 superfamily member 3 [Source:HGNC Symbol;Acc:HGNC:23049]		
ENSP00000426083.1	alcohol dehydrogenase 1C (class I), gamma polypeptide [Source:HGNC Symbol;Acc:HGNC:251	K13951	ADH1_7
ENSP00000225726.5	coiled-coil domain containing 47 [Source:HGNC Symbol;Acc:HGNC:24856]		
ENSP00000480040.1	TATA-box binding protein associated factor 15 [Source:HGNC Symbol;Acc:HGNC:11547]	K14651	TAF15, NPL3
ENSP00000295682.4	keratinocyte associated protein 2 [Source:HGNC Symbol;Acc:HGNC:28942]		
ENSP00000317159.4	cytochrome c1 [Source:HGNC Symbol;Acc:HGNC:2579]	K00413	CYC1, CYT1, petC
ENSP00000400101.2	solute carrier family 25 member 13 [Source:HGNC Symbol;Acc:HGNC:10983]	K15105	SLC25A12_13, AGC
ENSP00000354964.2	zinc finger protein 318 [Source:HGNC Symbol;Acc:HGNC:13578]		
ENSP00000281938.2	heat shock protein family B (small) member 8 [Source:HGNC Symbol;Acc:HGNC:30171]	K08879	HSPB8
ENSP00000468733.1	transmembrane protein 205 [Source:HGNC Symbol;Acc:HGNC:29631]		
ENSP00000407727.2	abhydrolase domain containing 16A [Source:HGNC Symbol;Acc:HGNC:13921]		
ENSP00000241337.4	glutathione S-transferase mu 2 [Source:HGNC Symbol;Acc:HGNC:4634]	K00799	GST, gst
ENSP00000482613.1	high mobility group nucleosomal binding domain 3 [Source:HGNC Symbol;Acc:HGNC:12312]		
ENSP00000321856.5	cysteine rich with EGF like domains 1 [Source:HGNC Symbol;Acc:HGNC:14630]		
ENSP00000290597.5	aldehyde dehydrogenase 4 family member A1 [Source:HGNC Symbol;Acc:HGNC:406]	K00294	E1.2.1.88
ENSP00000308208.6	matrix metallopeptidase 14 [Source:HGNC Symbol;Acc:HGNC:7160]	K07763	MMP14
ENSP00000401988.1	caldesmon 1 [Source:HGNC Symbol;Acc:HGNC:1441]	K12327	CALD1
ENSP00000435777.1	ribosomal protein S13 [Source:HGNC Symbol;Acc:HGNC:10386]	K02953	RP-S13e, RPS13
ENSP00000276914.2	perilipin 2 [Source:HGNC Symbol;Acc:HGNC:248]	K17284	PLIN2, ADRP
ENSP00000308610.5	glycerol-3-phosphate dehydrogenase 2 [Source:HGNC Symbol;Acc:HGNC:4456]	K00111	glpA, glpD
ENSP00000376472.3	STT3 oligosaccharyltransferase complex catalytic subunit A [Source:HGNC Symbol;Acc:HGNC:6	K07151	STT3
ENSP00000350937.4	testin LIM domain protein [Source:HGNC Symbol;Acc:HGNC:14620]		
ENSP00000462880.1	annexin A8 [Source:HGNC Symbol;Acc:HGNC:546]	K17096	ANXA8
ENSP00000484893.1	PIGB opposite strand 1 [Source:HGNC Symbol;Acc:HGNC:50696]		

ENSP00000412708.2	required for meiotic nuclear division 1 homolog [Source:HGNC Symbol;Acc:HGNC:21176]		
ENSP00000359504.4	guanylate binding protein 1 [Source:HGNC Symbol;Acc:HGNC:4182]	K20899	GBP1_3_4_7
ENSP00000356057.3	transmembrane protein 181 [Source:HGNC Symbol;Acc:HGNC:20958]		
ENSP00000378886.2	twinfilin actin binding protein 1 [Source:HGNC Symbol;Acc:HGNC:9620]	K08870	TWF
ENSP00000333277.1	histone cluster 2 H3 family member d [Source:HGNC Symbol;Acc:HGNC:25311]	K11253	H3
ENSP00000262030.3	ATP synthase F1 subunit beta [Source:HGNC Symbol;Acc:HGNC:830]	K02133	ATPeF1B, ATP5B, ATP2
ENSP00000356107.3	RAB29, member RAS oncogene family [Source:HGNC Symbol;Acc:HGNC:9789]	K07916	RAB7L1, RAB7L
ENSP00000360540.3	centrosomal protein 55 [Source:HGNC Symbol;Acc:HGNC:1161]	K16456	CEP55
ENSP00000252622.8	LSM7 homolog, U6 small nuclear RNA and mRNA degradation associated [Source:HGNC Symb	K12626	LSM7
ENSP00000499136.1	aldehyde dehydrogenase 1 family member L2 [Source:HGNC Symbol;Acc:HGNC:26777]	K00289	E1.5.1.6, FTHFD
ENSP00000281031.4	NADH:ubiquinone oxidoreductase subunit C2 [Source:HGNC Symbol;Acc:HGNC:7706]	K03968	NDUFC2
ENSP00000308430.8	cytochrome b5 type B [Source:HGNC Symbol;Acc:HGNC:24374]		
ENSP00000349576.3	dCMP deaminase [Source:HGNC Symbol;Acc:HGNC:2710]	K01493	comEB
ENSP00000294360.4	CXXC motif containing zinc binding protein [Source:HGNC Symbol;Acc:HGNC:26059]		
ENSP00000414376.2	thymosin beta 4 X-linked [Source:HGNC Symbol;Acc:HGNC:11881]	K05764	TMSB4
ENSP00000320650.3	DnaJ heat shock protein family (Hsp40) member C25 [Source:HGNC Symbol;Acc:HGNC:34187]	K19371	DNAJC25
ENSP00000356853.4	uridine-cytidine kinase 2 [Source:HGNC Symbol;Acc:HGNC:12562]	K00876	udk, UCK
ENSP00000439485.1	phosphotriesterase related [Source:HGNC Symbol;Acc:HGNC:9590]	K07048	PTER, php
ENSP00000424387.1	threonyl-tRNA synthetase [Source:HGNC Symbol;Acc:HGNC:11572]	K01868	TARS, thrS
ENSP00000371362.4	NAD kinase 2, mitochondrial [Source:HGNC Symbol;Acc:HGNC:26404]	K00858	ppnK, NADK
ENSP00000255030.5	C-reactive protein [Source:HGNC Symbol;Acc:HGNC:2367]		
ENSP00000284268.6	ANKH inorganic pyrophosphate transport regulator [Source:HGNC Symbol;Acc:HGNC:15492]		
ENSP00000355809.2	ENAH actin regulator [Source:HGNC Symbol;Acc:HGNC:18271]	K05746	ENAH, MENA
ENSP00000498353.1	hypoxia up-regulated 1 [Source:HGNC Symbol;Acc:HGNC:16931]	K09486	HYOU1
ENSP00000364945.2	dimethylarginine dimethylaminohydrolase 2 [Source:HGNC Symbol;Acc:HGNC:2716]	K01482	DDAH, ddaH
ENSP00000356563.4	peroxisomal biogenesis factor 3 [Source:HGNC Symbol;Acc:HGNC:8858]	K13336	PEX3
ENSP00000344779.3	rhomboid domain containing 1 [Source:HGNC Symbol;Acc:HGNC:23081]	K09651	RHBDD1
ENSP00000310117.3	protein phosphatase 1 regulatory inhibitor subunit 14B [Source:HGNC Symbol;Acc:HGNC:9057	K17555	PPP1R14B
ENSP00000362873.3	NADH:ubiquinone oxidoreductase subunit A8 [Source:HGNC Symbol;Acc:HGNC:7692]	K03952	NDUFA8
ENSP00000478763.1	signal regulatory protein alpha [Source:HGNC Symbol;Acc:HGNC:9662]	K06551	SIRPA_B1_G, CD172
ENSP00000362524.3	angiopoietin like 2 [Source:HGNC Symbol;Acc:HGNC:490]		
ENSP00000263408.4	complement C9 [Source:HGNC Symbol;Acc:HGNC:1358]	K04000	C9
ENSP00000355889.2	isoleucyl-tRNA synthetase 2, mitochondrial [Source:HGNC Symbol;Acc:HGNC:29685]	K01870	IARS, ileS
ENSP00000295770.2	STT3 oligosaccharyltransferase complex catalytic subunit B [Source:HGNC Symbol;Acc:HGNC:3	K07151	STT3
ENSP00000390722.2	solute carrier family 25 member 17 [Source:HGNC Symbol;Acc:HGNC:10987]	K13354	SLC25A17, PMP34
ENSP00000350509.4	androgen induced 1 [Source:HGNC Symbol;Acc:HGNC:21607]		
ENSP00000356591.3	sterol O-acyltransferase 1 [Source:HGNC Symbol;Acc:HGNC:11177]	K00637	SOAT
ENSP00000376309.2	heterogeneous nuclear ribonucleoprotein A3 [Source:HGNC Symbol;Acc:HGNC:24941]	K12741	HNRNPA1_3

ENSP00000327589.1	glutamate dehydrogenase 2 [Source:HGNC Symbol;Acc:HGNC:4336]	K00261	GLUD1_2, gdhA
ENSP00000423321.1	CLPTM1 like [Source:HGNC Symbol;Acc:HGNC:24308]		
ENSP00000342023.5	F-box protein 38 [Source:HGNC Symbol;Acc:HGNC:28844]	K10313	FBXO38, MOKA
ENSP00000339191.2	caveolin 1 [Source:HGNC Symbol;Acc:HGNC:1527]		
ENSP00000435726.1	WD repeat domain 74 [Source:HGNC Symbol;Acc:HGNC:25529]	K14841	NSA1, WDR74
ENSP00000297848.3	collagen type XIV alpha 1 chain [Source:HGNC Symbol;Acc:HGNC:2191]	K08133	COL14A
ENSP00000357288.4	late endosomal/lysosomal adaptor, MAPK and MTOR activator 2 [Source:HGNC Symbol;Acc:H	K20398	LAMTOR2
ENSP00000378363.2	DnaJ heat shock protein family (Hsp40) member B12 [Source:HGNC Symbol;Acc:HGNC:14891]	K09518	DNAJB12
ENSP00000310565.7	dermatan sulfate epimerase like [Source:HGNC Symbol;Acc:HGNC:18144]		
ENSP00000354722.2	eukaryotic translation initiation factor 1A Y-linked [Source:HGNC Symbol;Acc:HGNC:3252]	K03236	EIF1A
ENSP00000360483.3	NDC1 transmembrane nucleoporin [Source:HGNC Symbol;Acc:HGNC:25525]	K14315	NDC1, TMEM48
ENSP00000380747.2	transmembrane protein 209 [Source:HGNC Symbol;Acc:HGNC:21898]		
ENSP00000362724.5	ribophorin II [Source:HGNC Symbol;Acc:HGNC:10382]		
ENSP00000329867.8	protein phosphatase methylesterase 1 [Source:HGNC Symbol;Acc:HGNC:30178]	K13617	PPME1
ENSP00000421669.1	UTP15 small subunit processome component [Source:HGNC Symbol;Acc:HGNC:25758]	K14549	UTP15
ENSP00000254810.3	H3 histone family member 3B [Source:HGNC Symbol;Acc:HGNC:4765]	K11253	H3
ENSP00000325421.4	presenilin associated rhomboid like [Source:HGNC Symbol;Acc:HGNC:18253]	K09650	PARL, PSARL, PCP1
ENSP00000334308.4	spectrin repeat containing nuclear envelope family member 3 [Source:HGNC Symbol;Acc:HGNC:19861]		
ENSP00000276431.4	TNF receptor superfamily member 10b [Source:HGNC Symbol;Acc:HGNC:11905]	K04722	TNFRSF10, TRAILR, CD
ENSP00000353032.7	purinergic receptor P2X 4 [Source:HGNC Symbol;Acc:HGNC:8535]		
ENSP00000350277.2	plectin [Source:HGNC Symbol;Acc:HGNC:9069]	K10388	PLEC
ENSP00000225665.7	solute carrier family 25 member 11 [Source:HGNC Symbol;Acc:HGNC:10981]	K15104	SLC25A11, OGC
ENSP00000347988.2	NADH:ubiquinone oxidoreductase subunit A5 [Source:HGNC Symbol;Acc:HGNC:7688]	K03949	NDUFA5
ENSP00000437673.1	four and a half LIM domains 1 [Source:HGNC Symbol;Acc:HGNC:3702]	K14365	FHL1, SLIM1
ENSP00000498597.1	TSPY like 1 [Source:HGNC Symbol;Acc:HGNC:12382]	K11284	TSPYL1
ENSP00000263665.6	contactin 3 [Source:HGNC Symbol;Acc:HGNC:2173]	K06761	CNTN3
ENSP00000333664.8	acetyl-CoA acyltransferase 1 [Source:HGNC Symbol;Acc:HGNC:82]	K07513	ACAA1
ENSP00000377060.3	chromobox 1 [Source:HGNC Symbol;Acc:HGNC:1551]	K11585	CBX1, HP1B, SWI6
ENSP00000359539.5	glutamic-oxaloacetic transaminase 1 [Source:HGNC Symbol;Acc:HGNC:4432]	K14454	GOT1
ENSP00000353741.5	eukaryotic translation termination factor 1 [Source:HGNC Symbol;Acc:HGNC:3477]	K03265	ETF1, ERF1
ENSP00000388731.2	ITPR interacting domain containing 2 [Source:HGNC Symbol;Acc:HGNC:11319]		
ENSP00000228495.6	potassium channel tetramerization domain containing 10 [Source:HGNC Symbol;Acc:HGNC:23	K15074	BACURD
ENSP00000358510.3	FUN14 domain containing 2 [Source:HGNC Symbol;Acc:HGNC:24925]		
ENSP00000383115.3	collectin subfamily member 12 [Source:HGNC Symbol;Acc:HGNC:16016]	K10062	COLEC12
ENSP00000215567.4	trans-2,3-enoyl-CoA reductase [Source:HGNC Symbol;Acc:HGNC:4551]	K10258	TER, TSC13, CER10
ENSP00000391481.1	transketolase [Source:HGNC Symbol;Acc:HGNC:11834]	K00615	E2.2.1.1, tktA, tktB
ENSP00000262213.2	translocation associated membrane protein 1 [Source:HGNC Symbol;Acc:HGNC:20568]	K14010	TRAM1
ENSP00000268613.1	cadherin 13 [Source:HGNC Symbol;Acc:HGNC:1753]	K06808	CDH13

ENSP00000269097.3	glucose-6-phosphatase catalytic subunit 3 [Source:HGNC Symbol;Acc:HGNC:24861]	K01084	G6PC
ENSP00000496526.2	ATP binding cassette subfamily B member 7 [Source:HGNC Symbol;Acc:HGNC:48]	K05662	ABCB7
ENSP00000360682.3	PAXX non-homologous end joining factor [Source:HGNC Symbol;Acc:HGNC:27849]		
ENSP00000355110.3	SPARC related modular calcium binding 1 [Source:HGNC Symbol;Acc:HGNC:20318]		
ENSP00000239891.3	ALG5 dolichyl-phosphate beta-glucosyltransferase [Source:HGNC Symbol;Acc:HGNC:20266]	K00729	ALG5
ENSP00000261601.6	ubiquitin specific peptidase 14 [Source:HGNC Symbol;Acc:HGNC:12612]	K11843	USP14, UBP6
ENSP00000418401.1	SRP receptor subunit beta [Source:HGNC Symbol;Acc:HGNC:24085]	K12272	SRPRB, SRP102
ENSP00000419088.1	family with sequence similarity 162 member A [Source:HGNC Symbol;Acc:HGNC:17865]		
ENSP00000240285.5	retinol dehydrogenase 10 [Source:HGNC Symbol;Acc:HGNC:19975]	K11151	RDH10
ENSP00000291572.8	1-acylglycerol-3-phosphate O-acyltransferase 3 [Source:HGNC Symbol;Acc:HGNC:326]	K13523	AGPAT3_4
ENSP00000309092.6	GDP-mannose pyrophosphorylase B [Source:HGNC Symbol;Acc:HGNC:22932]		
ENSP00000417653.1	drebrin like [Source:HGNC Symbol;Acc:HGNC:2696]		
ENSP00000320246.6	solute carrier family 9 member A9 [Source:HGNC Symbol;Acc:HGNC:20653]	K14725	SLC9A9, NHE9
ENSP00000378484.3	voltage dependent anion channel 1 [Source:HGNC Symbol;Acc:HGNC:12669]	K05862	VDAC1
ENSP00000256644.4	late endosomal/lysosomal adaptor, MAPK and MTOR activator 5 [Source:HGNC Symbol;Acc:H	K16344	LAMTOR5, HBXIP
ENSP00000275730.4	solute carrier family 12 member 9 [Source:HGNC Symbol;Acc:HGNC:17435]	K14429	SLC12A9, CCC6
ENSP00000452879.1	tropomyosin 1 [Source:HGNC Symbol;Acc:HGNC:12010]		
ENSP00000361777.4	SET nuclear proto-oncogene [Source:HGNC Symbol;Acc:HGNC:10760]	K11290	SET, TAF1, I2PP2A
ENSP00000317985.6	Rho associated coiled-coil containing protein kinase 2 [Source:HGNC Symbol;Acc:HGNC:1025	K17388	ROCK2
ENSP00000385385.2	protein disulfide isomerase family A member 6 [Source:HGNC Symbol;Acc:HGNC:30168]	K09584	PDIA6, TXNDC7
ENSP00000291568.5	cystatin B [Source:HGNC Symbol;Acc:HGNC:2482]	K13907	CSTA_B
ENSP00000265734.4	cyclin dependent kinase 6 [Source:HGNC Symbol;Acc:HGNC:1777]	K02091	CDK6
ENSP00000482455.1	mesoderm development LRP chaperone [Source:HGNC Symbol;Acc:HGNC:13520]		
ENSP00000344503.6	transmembrane protein 201 [Source:HGNC Symbol;Acc:HGNC:33719]		
ENSP00000297156.2	calcium modulating ligand [Source:HGNC Symbol;Acc:HGNC:1471]		
ENSP00000338703.5	UDP-glucose pyrophosphorylase 2 [Source:HGNC Symbol;Acc:HGNC:12527]	K00963	UGP2, galU, galF
ENSP00000359393.3	high mobility group box 3 [Source:HGNC Symbol;Acc:HGNC:5004]	K11296	HMGB3
ENSP00000370849.3	ITPRIP like 2 [Source:HGNC Symbol;Acc:HGNC:27257]		
ENSP00000354960.4	collagen beta(1-O)galactosyltransferase 2 [Source:HGNC Symbol;Acc:HGNC:16790]	K11703	GLT25D
ENSP00000343690.5	dihydropyrimidinase like 3 [Source:HGNC Symbol;Acc:HGNC:3015]		
ENSP00000319977.7	N-myc downstream regulated 1 [Source:HGNC Symbol;Acc:HGNC:7679]	K18266	NDRG1
ENSP00000363458.4	low density lipoprotein receptor adaptor protein 1 [Source:HGNC Symbol;Acc:HGNC:18640]	K12474	LDLRAP1, ARH
ENSP00000338004.3	translocator protein [Source:HGNC Symbol;Acc:HGNC:1158]	K05770	TSPO, BZRP
ENSP00000320084.5	CD276 molecule [Source:HGNC Symbol;Acc:HGNC:19137]	K06746	B7H3, CD276
ENSP00000295148.4	WD repeat and coiled coil containing [Source:HGNC Symbol;Acc:HGNC:26157]		
ENSP00000312017.7	TLC domain containing 3A [Source:HGNC Symbol;Acc:HGNC:29646]		
ENSP00000396308.2	dihydrofolate reductase [Source:HGNC Symbol;Acc:HGNC:2861]	K00287	folA
ENSP00000320324.4	aminopeptidase puromycin sensitive [Source:HGNC Symbol;Acc:HGNC:7900]	K08776	NPEPPS

ENSP00000359305.3	transmembrane p24 trafficking protein 5 [Source:HGNC Symbol;Acc:HGNC:24251]	K14825	TMED5, ERP2, ERP4
ENSP00000389649.2	ATP synthase peripheral stalk subunit F6 [Source:HGNC Symbol;Acc:HGNC:847]	K02131	ATPeF0F6, ATP5J
ENSP00000363591.3	BCL2 antagonist/killer 1 [Source:HGNC Symbol;Acc:HGNC:949]	K14021	BAK, BAK1
ENSP00000344166.2	TMEM189-UBE2V1 readthrough [Source:HGNC Symbol;Acc:HGNC:33521]	K10704;	TMEM189;UBE2V
ENSP00000356602.3	vesicle trafficking 1 [Source:HGNC Symbol;Acc:HGNC:20954]	K12199	VTA1, LIP5
ENSP00000346931.1	chromosome 6 open reading frame 120 [Source:HGNC Symbol;Acc:HGNC:21247]		
ENSP00000223095.4	serpin family E member 1 [Source:HGNC Symbol;Acc:HGNC:8583]	K03982	SERPINE1, PAI1
ENSP00000379629.1	cyclin dependent kinase inhibitor 1B [Source:HGNC Symbol;Acc:HGNC:1785]	K06624	CDKN1B, P27, KIP1
ENSP00000263697.4	DnaJ heat shock protein family (Hsp40) member C8 [Source:HGNC Symbol;Acc:HGNC:15470]	K09528	DNAJC8
ENSP00000331901.4	eukaryotic translation elongation factor 1 gamma [Source:HGNC Symbol;Acc:HGNC:3213]	K03233	EEF1G
ENSP00000211372.5	ribosomal protein S18 [Source:HGNC Symbol;Acc:HGNC:10401]	K02964	RP-S18e, RPS18
ENSP00000437218.1	cysteine and glycine rich protein 1 [Source:HGNC Symbol;Acc:HGNC:2469]	K09377	CSRP
ENSP00000270538.2	translocase of inner mitochondrial membrane 44 [Source:HGNC Symbol;Acc:HGNC:17316]	K17804	TIM44
ENSP00000398053.2	fumarylacetoacetate hydrolase domain containing 1 [Source:HGNC Symbol;Acc:HGNC:14169]	K01557	FAHD1
ENSP00000262890.2	platelet activating factor acetylhydrolase 1b catalytic subunit 3 [Source:HGNC Symbol;Acc:HGN	K16795	PAFAH1B2_3
ENSP00000376919.2	LUC7 like 3 pre-mRNA splicing factor [Source:HGNC Symbol;Acc:HGNC:24309]		
ENSP00000491014.1	MIA SH3 domain ER export factor 2 [Source:HGNC Symbol;Acc:HGNC:18432]		
ENSP00000285419.3	phosphatidylinositol-4,5-bisphosphate 4-phosphatase 2 [Source:HGNC Symbol;Acc:HGNC:25	K13084	TMEM55
ENSP00000370125.5	high mobility group nucleosome binding domain 1 [Source:HGNC Symbol;Acc:HGNC:4984]	K11299	HMGN1
ENSP00000347924.2	sideroflexin 4 [Source:HGNC Symbol;Acc:HGNC:16088]		
ENSP00000227525.3	transmembrane protein 109 [Source:HGNC Symbol;Acc:HGNC:28771]		
ENSP00000318602.5	phosphoribosyl transferase domain containing 1 [Source:HGNC Symbol;Acc:HGNC:23333]		
ENSP00000011473.2	synaptophysin like 1 [Source:HGNC Symbol;Acc:HGNC:11507]		
ENSP00000379838.2	calcium regulated heat stable protein 1 [Source:HGNC Symbol;Acc:HGNC:17150]		
ENSP00000428585.1	small integral membrane protein 12 [Source:HGNC Symbol;Acc:HGNC:25154]		
ENSP00000252029.3	thymidine phosphorylase [Source:HGNC Symbol;Acc:HGNC:3148]	K00758	deoA, TYMP
ENSP00000478532.1	catenin beta like 1 [Source:HGNC Symbol;Acc:HGNC:15879]	K12864	CTNNBL1
ENSP00000307697.5	acyl-CoA oxidase 2 [Source:HGNC Symbol;Acc:HGNC:120]	K10214	ACOX2
ENSP00000394056.2	protein phosphatase 1 regulatory inhibitor subunit 11 [Source:HGNC Symbol;Acc:HGNC:9285]	K17553	PPP1R11
ENSP00000383898.3	solute carrier family 25 member 3 [Source:HGNC Symbol;Acc:HGNC:10989]		
ENSP00000354532.5	purine nucleoside phosphorylase [Source:HGNC Symbol;Acc:HGNC:7892]	K03783	punA, PNP
ENSP00000245552.2	5', 3'-nucleotidase, cytosolic [Source:HGNC Symbol;Acc:HGNC:17144]	K01081	E3.1.3.5
ENSP00000296591.4	EGF like repeats and discoidin domains 3 [Source:HGNC Symbol;Acc:HGNC:3173]		
ENSP00000484580.1	protein phosphatase 1 regulatory inhibitor subunit 2 [Source:HGNC Symbol;Acc:HGNC:9288]	K16833	PPP1R2, IPP2
ENSP00000342676.3	aurora kinase A interacting protein 1 [Source:HGNC Symbol;Acc:HGNC:24114]	K16830	AURKAIP1
ENSP00000262094.4	RAB27B, member RAS oncogene family [Source:HGNC Symbol;Acc:HGNC:9767]	K07886	RAB27B
ENSP00000233114.8	malate dehydrogenase 1 [Source:HGNC Symbol;Acc:HGNC:6970]	K00025	MDH1
ENSP00000376276.2	serine and arginine rich splicing factor 2 [Source:HGNC Symbol;Acc:HGNC:10783]	K12891	SFRS2

ENSP00000361664.1	human immunodeficiency virus type I enhancer binding protein 3 [Source:HGNC Symbol;Acc:H	K09239	HIVEP
ENSP00000302229.8	phospholipid phosphatase 1 [Source:HGNC Symbol;Acc:HGNC:9228]	K01080	PPAP2
ENSP00000341874.3	RIO kinase 3 [Source:HGNC Symbol;Acc:HGNC:11451]	K08872	RIOK3, SUDD
ENSP00000355308.3	3-hydroxyacyl-CoA dehydratase 1 [Source:HGNC Symbol;Acc:HGNC:9639]	K10703	PHS1, PAS2
ENSP00000293971.6	amidohydrolase domain containing 2 [Source:HGNC Symbol;Acc:HGNC:24262]	K01443	nagA, AMDHD2
ENSP00000286713.2	stomatin [Source:HGNC Symbol;Acc:HGNC:3383]	K17286	STOM
ENSP00000222553.3	nicotinamide phosphoribosyltransferase [Source:HGNC Symbol;Acc:HGNC:30092]	K03462	NAMPT
ENSP00000272233.4	ras homolog family member B [Source:HGNC Symbol;Acc:HGNC:668]	K07856	RHOB
ENSP00000256545.4	ER membrane protein complex subunit 7 [Source:HGNC Symbol;Acc:HGNC:24301]		
ENSP00000367770.4	solute carrier family 35 member B4 [Source:HGNC Symbol;Acc:HGNC:20584]	K15278	SLC35B4, YEA4
ENSP00000429366.1	antioxidant 1 copper chaperone [Source:HGNC Symbol;Acc:HGNC:798]	K07213	ATOX1, ATX1, copZ, g(
ENSP00000346725.6	pescadillo ribosomal biogenesis factor 1 [Source:HGNC Symbol;Acc:HGNC:8848]	K14843	PES1, NOP7
ENSP00000385703.1	LBH regulator of WNT signaling pathway [Source:HGNC Symbol;Acc:HGNC:29532]		
ENSP00000400142.2	EWS RNA binding protein 1 [Source:HGNC Symbol;Acc:HGNC:3508]	K13209	EWSR1
ENSP00000389297.2	coiled-coil-helix-coiled-coil-helix domain containing 3 [Source:HGNC Symbol;Acc:HGNC:21906]		
ENSP00000363162.3	ATPase H+ transporting V1 subunit G1 [Source:HGNC Symbol;Acc:HGNC:864]	K02152	ATPeV1G, ATP6G
ENSP00000366430.1	leucine zipper and CTNNBIP1 domain containing [Source:HGNC Symbol;Acc:HGNC:17497]		
ENSP00000299299.3	pterin-4 alpha-carbinolamine dehydratase 1 [Source:HGNC Symbol;Acc:HGNC:8646]	K01724	PCBD, phhB
ENSP00000230050.3	ribosomal protein S12 [Source:HGNC Symbol;Acc:HGNC:10385]	K02951	RP-S12e, RPS12
ENSP00000264335.8	tyrosine 3-monooxygenase/tryptophan 5-monooxygenase activation protein epsilon [Source:I	K06630	YWHAE
ENSP00000299213.7	La ribonucleoprotein domain family member 6 [Source:HGNC Symbol;Acc:HGNC:24012]	K18733	LARP6
ENSP00000299626.5	ALG8 alpha-1,3-glucosyltransferase [Source:HGNC Symbol;Acc:HGNC:23161]	K03849	ALG8
ENSP00000360883.4	interferon induced protein with tetratricopeptide repeats 3 [Source:HGNC Symbol;Acc:HGNC:5411]		
ENSP00000359727.4	BCL2 associated athanogene 2 [Source:HGNC Symbol;Acc:HGNC:938]	K09556	BAG2
ENSP00000498170.1	histocompatibility minor 13 [Source:HGNC Symbol;Acc:HGNC:16435]		
ENSP00000261381.6	xylosyltransferase 1 [Source:HGNC Symbol;Acc:HGNC:15516]	K00771	XYLT
ENSP00000483820.1	pleckstrin homology and RUN domain containing M1 [Source:HGNC Symbol;Acc:HGNC:29017]		
ENSP00000361711.1	transcription elongation factor A like 3 [Source:HGNC Symbol;Acc:HGNC:28247]		
ENSP00000497605.1	inositol 1,4,5-trisphosphate receptor type 1 [Source:HGNC Symbol;Acc:HGNC:6180]		
ENSP00000400717.2	G protein subunit alpha 13 [Source:HGNC Symbol;Acc:HGNC:4381]	K04639	GNA13
ENSP00000342962.3	serine incorporator 1 [Source:HGNC Symbol;Acc:HGNC:13464]		
ENSP00000455736.1	pyruvate kinase M1/2 [Source:HGNC Symbol;Acc:HGNC:9021]		
ENSP00000216392.7	glycogen phosphorylase L [Source:HGNC Symbol;Acc:HGNC:9725]	K00688	PYG, glgP
ENSP00000355880.3	mitochondrial amidoxime reducing component 2 [Source:HGNC Symbol;Acc:HGNC:26064]		
ENSP00000496166.1	aldolase, fructose-bisphosphate A [Source:HGNC Symbol;Acc:HGNC:414]	K01623	ALDO
ENSP00000299767.4	heat shock protein 90 beta family member 1 [Source:HGNC Symbol;Acc:HGNC:12028]	K09487	HSP90B, TRA1
ENSP00000328521.4	cysteine rich protein 2 [Source:HGNC Symbol;Acc:HGNC:2361]		
ENSP00000362352.3	H2A histone family member Y2 [Source:HGNC Symbol;Acc:HGNC:14453]	K11251	H2A

ENSP00000376111.1	matrix AAA peptidase interacting protein 1 [Source:HGNC Symbol;Acc:HGNC:26198]		
ENSP00000300456.3	solute carrier family 27 member 4 [Source:HGNC Symbol;Acc:HGNC:10998]	K08745	SLC27A1_4, FATP1, FAT
ENSP00000360664.4	basic transcription factor 3 like 4 [Source:HGNC Symbol;Acc:HGNC:30547]	K01527	EGD1, BTF3
ENSP00000264220.2	phosphoribosyl pyrophosphate amidotransferase [Source:HGNC Symbol;Acc:HGNC:9238]	K00764	purF, PPAT
ENSP00000253457.3	ER membrane protein complex subunit 8 [Source:HGNC Symbol;Acc:HGNC:7864]		
ENSP00000440905.1	lunapark, ER junction formation factor [Source:HGNC Symbol;Acc:HGNC:21610]		
ENSP00000286091.5	protein disulfide isomerase family A member 4 [Source:HGNC Symbol;Acc:HGNC:30167]	K09582	PDIA4, ERP72
ENSP00000332449.7	cysteine rich protein 1 [Source:HGNC Symbol;Acc:HGNC:2360]		
ENSP00000363590.3	chondroitin sulfate N-acetylgalactosaminyltransferase 2 [Source:HGNC Symbol;Acc:HGNC:242	K00746	CSGALNACT1_2
ENSP00000281187.5	VPS26, retromer complex component B [Source:HGNC Symbol;Acc:HGNC:28119]	K18466	VPS26
ENSP00000370745.1	DEAD-box helicase 1 [Source:HGNC Symbol;Acc:HGNC:2734]	K13177	DDX1
ENSP00000001008.4	FKBP prolyl isomerase 4 [Source:HGNC Symbol;Acc:HGNC:3720]	K09571	FKBP4_5
ENSP00000216259.7	phosphomannomutase 1 [Source:HGNC Symbol;Acc:HGNC:9114]	K17497	PMM
ENSP00000324204.8	maturin, neural progenitor differentiation regulator homolog [Source:HGNC Symbol;Acc:HGNC:25457]		
ENSP00000281282.5	cingulin like 1 [Source:HGNC Symbol;Acc:HGNC:25931]	K21110	CGNL1
ENSP00000369849.4	mesenteric estrogen dependent adipogenesis [Source:HGNC Symbol;Acc:HGNC:25926]		
ENSP00000210444.5	N-acetylneuraminate synthase [Source:HGNC Symbol;Acc:HGNC:19237]	K05304	NANS, SAS
ENSP00000441282.1	ATPase H+ transporting V0 subunit d1 [Source:HGNC Symbol;Acc:HGNC:13724]		
ENSP00000378243.3	calcium/calmodulin dependent protein kinase II gamma [Source:HGNC Symbol;Acc:HGNC:1463]		
ENSP00000253792.2	ATP citrate lyase [Source:HGNC Symbol;Acc:HGNC:115]	K01648	ACLY
ENSP00000358635.3	synaptotagmin binding cytoplasmic RNA interacting protein [Source:HGNC Symbol;Acc:HGNC	K13160	SYNCRIP, HNRPQ
ENSP00000378731.2	aldolase, fructose-bisphosphate C [Source:HGNC Symbol;Acc:HGNC:418]	K01623	ALDO
ENSP00000476117.2	WD repeat domain 18 [Source:HGNC Symbol;Acc:HGNC:17956]	K14829	IPI3
ENSP00000285930.3	aldo-keto reductase family 1 member B [Source:HGNC Symbol;Acc:HGNC:381]	K00011	AKR1B
ENSP00000354511.6	catechol-O-methyltransferase [Source:HGNC Symbol;Acc:HGNC:2228]	K00545	COMT
ENSP00000305892.9	transmembrane protein 208 [Source:HGNC Symbol;Acc:HGNC:25015]		
ENSP00000233242.1	apolipoprotein B [Source:HGNC Symbol;Acc:HGNC:603]	K14462	APOB
ENSP00000454746.2	novel protein	K02957	RP-S15Ae, RPS15A
ENSP00000262225.3	transmembrane p24 trafficking protein 2 [Source:HGNC Symbol;Acc:HGNC:16996]	K20347	TMED2, EMP24
ENSP00000442688.1	major facilitator superfamily domain containing 5 [Source:HGNC Symbol;Acc:HGNC:28156]		
ENSP00000354742.4	importin 9 [Source:HGNC Symbol;Acc:HGNC:19425]	K20224	IPO9, RANBP9
ENSP00000382204.2	jumonji domain containing 1C [Source:HGNC Symbol;Acc:HGNC:12313]	K11449	JMJD1C
ENSP00000006777.6	rhomboid domain containing 2 [Source:HGNC Symbol;Acc:HGNC:23082]	K09652	RHBDD2, RHBDL7
ENSP00000397261.2	selenium binding protein 1 [Source:HGNC Symbol;Acc:HGNC:10719]	K17285	SELENBP1
ENSP00000256383.4	eukaryotic translation initiation factor 2 subunit alpha [Source:HGNC Symbol;Acc:HGNC:3265]	K03237	EIF2S1
ENSP00000378366.2	flotillin 2 [Source:HGNC Symbol;Acc:HGNC:3758]		
ENSP00000356988.2	nitrilase 1 [Source:HGNC Symbol;Acc:HGNC:7828]		
ENSP00000276689.3	NADH:ubiquinone oxidoreductase subunit B9 [Source:HGNC Symbol;Acc:HGNC:7704]	K03965	NDUFB9

ENSP00000268129.5	abhydrolase domain containing 2 [Source:HGNC Symbol;Acc:HGNC:18717]	K13697	ABHD2
ENSP00000331288.4	transmembrane protein 173 [Source:HGNC Symbol;Acc:HGNC:27962]	K12654	TMEM173, MITA
ENSP00000359151.3	dihydrolipoamide branched chain transacylase E2 [Source:HGNC Symbol;Acc:HGNC:2698]	K09699	DBT, bkdB
ENSP00000259963.3	family with sequence similarity 8 member A1 [Source:HGNC Symbol;Acc:HGNC:16372]		
ENSP00000218652.7	Nedd4 family interacting protein 2 [Source:HGNC Symbol;Acc:HGNC:18537]		
ENSP00000363832.2	aldehyde oxidase 1 [Source:HGNC Symbol;Acc:HGNC:553]	K00157	AOX
ENSP00000352522.2	ATPase H+ transporting V1 subunit H [Source:HGNC Symbol;Acc:HGNC:18303]	K02144	ATPeV1H
ENSP00000472264.1	ubiquitin A-52 residue ribosomal protein fusion product 1 [Source:HGNC Symbol;Acc:HGNC:12458]		
ENSP00000368447.4	GPALPP motifs containing 1 [Source:HGNC Symbol;Acc:HGNC:20298]		
ENSP00000430548.1	phosphatidylserine synthase 1 [Source:HGNC Symbol;Acc:HGNC:9587]	K08729	PTDSS1
ENSP00000360613.2	ubiquitin conjugating enzyme E2 A [Source:HGNC Symbol;Acc:HGNC:12472]	K10573	UBE2A, UBC2, RAD6A
ENSP00000218099.2	coagulation factor IX [Source:HGNC Symbol;Acc:HGNC:3551]	K01321	F9
ENSP00000250101.5	thioredoxin domain containing 17 [Source:HGNC Symbol;Acc:HGNC:28218]		
ENSP00000426909.1	receptor for activated C kinase 1 [Source:HGNC Symbol;Acc:HGNC:4399]	K14753	RACK1
ENSP00000426638.1	electron transfer flavoprotein dehydrogenase [Source:HGNC Symbol;Acc:HGNC:3483]	K00311	ETFDH
ENSP00000355968.4	solute carrier family 30 member 1 [Source:HGNC Symbol;Acc:HGNC:11012]	K14688	SLC30A1, ZNT1
ENSP00000433112.1	dickkopf WNT signaling pathway inhibitor 3 [Source:HGNC Symbol;Acc:HGNC:2893]		
ENSP00000267113.4	extended synaptotagmin 1 [Source:HGNC Symbol;Acc:HGNC:29534]		
ENSP00000364649.3	succinate dehydrogenase complex iron sulfur subunit B [Source:HGNC Symbol;Acc:HGNC:1068	K00235	SDHB, SDH2
ENSP00000481455.1	membrane bound O-acyltransferase domain containing 7 [Source:HGNC Symbol;Acc:HGNC:1	K13516	MBOAT7
ENSP00000345445.4	SAMM50 sorting and assembly machinery component [Source:HGNC Symbol;Acc:HGNC:2427	K07277	SAM50, TOB55, bamA
ENSP00000417972.1	SEC22 homolog A, vesicle trafficking protein [Source:HGNC Symbol;Acc:HGNC:20260]	K08520	SEC22A_C
ENSP00000295448.3	glucosamine-6-phosphate deaminase 2 [Source:HGNC Symbol;Acc:HGNC:21526]	K02564	nagB, GNPDA
ENSP00000428112.1	ER lipid raft associated 2 [Source:HGNC Symbol;Acc:HGNC:1356]		
ENSP00000318318.6	mannose phosphate isomerase [Source:HGNC Symbol;Acc:HGNC:7216]	K01809	manA, MPI
ENSP00000327801.4	prolyl 4-hydroxylase subunit beta [Source:HGNC Symbol;Acc:HGNC:8548]	K09580	PDIA1, P4HB
ENSP00000364235.3	ring finger protein 5 [Source:HGNC Symbol;Acc:HGNC:10068]	K10666	RNF5
ENSP00000427463.1	signal peptidase complex subunit 3 [Source:HGNC Symbol;Acc:HGNC:26212]	K12948	SPCS3, SPC3
ENSP00000379983.2	autophagy related 9A [Source:HGNC Symbol;Acc:HGNC:22408]	K17907	ATG9
ENSP00000362111.4	tetraspanin 6 [Source:HGNC Symbol;Acc:HGNC:11858]	K17295	TSPAN6
ENSP00000478274.1	survival of motor neuron 2, centromeric [Source:HGNC Symbol;Acc:HGNC:11118]		
ENSP00000338607.3	thioesterase superfamily member 6 [Source:HGNC Symbol;Acc:HGNC:29656]		
ENSP00000263440.5	ALG6 alpha-1,3-glucosyltransferase [Source:HGNC Symbol;Acc:HGNC:23157]	K03848	ALG6
ENSP00000400175.1	ras homolog family member A [Source:HGNC Symbol;Acc:HGNC:667]		
ENSP00000347184.5	huntingtin [Source:HGNC Symbol;Acc:HGNC:4851]	K04533	HD
ENSP00000260385.6	regulator of microtubule dynamics 3 [Source:HGNC Symbol;Acc:HGNC:25550]		
ENSP00000239449.4	protocadherin beta 14 [Source:HGNC Symbol;Acc:HGNC:8685]	K16494	PCDHB
ENSP00000499349.1	potassium voltage-gated channel subfamily A regulatory beta subunit 2 [Source:HGNC Symbo	K04883	KCNAB2

ENSP00000302886.6	proliferation-associated 2G4 [Source:HGNC Symbol;Acc:HGNC:8550]		
ENSP00000294623.4	far upstream element binding protein 1 [Source:HGNC Symbol;Acc:HGNC:4004]	K13210	FUBP
ENSP00000490747.1	MIEF1 upstream open reading frame protein [Source:UniProtKB/Swiss-Prot;Acc:L0R8F8]		
ENSP00000315263.3	glycolipid transfer protein [Source:HGNC Symbol;Acc:HGNC:24867]		
ENSP00000424341.1	calnexin [Source:HGNC Symbol;Acc:HGNC:1473]	K08054	CANX
ENSP00000404190.2	Rac GTPase activating protein 1 [Source:HGNC Symbol;Acc:HGNC:9804]	K16733	RACGAP1, Tum
ENSP00000353192.3	X-ray repair cross complementing 6 [Source:HGNC Symbol;Acc:HGNC:4055]	K10884	XRCC6, KU70, G22P1
ENSP00000305653.2	leucine zipper and EF-hand containing transmembrane protein 1 [Source:HGNC Symbol;Acc:H	K17800	LETM1, MDM38
ENSP00000263035.4	dehydrogenase E1 and transketolase domain containing 1 [Source:HGNC Symbol;Acc:HGNC:2	K15791	DHKTD1
ENSP00000308227.4	high mobility group AT-hook 1 [Source:HGNC Symbol;Acc:HGNC:5010]	K09282	HMGA1
ENSP00000361508.3	phospholipid transfer protein [Source:HGNC Symbol;Acc:HGNC:9093]	K08761	PLTP
ENSP00000372873.4	flotillin 1 [Source:HGNC Symbol;Acc:HGNC:3757]	K07192	FLOT
ENSP00000224073.1	endothelial differentiation related factor 1 [Source:HGNC Symbol;Acc:HGNC:3164]	K03627	MBF1
ENSP00000359297.3	NAD(P) dependent steroid dehydrogenase-like [Source:HGNC Symbol;Acc:HGNC:13398]	K07748	E1.1.1.170, NSDHL, ER(
ENSP00000296003.4	myotubularin related protein 14 [Source:HGNC Symbol;Acc:HGNC:26190]	K18086	MTMR14
ENSP00000258796.7	tweety family member 3 [Source:HGNC Symbol;Acc:HGNC:22222]		
ENSP00000256854.4	asparaginyl-tRNA synthetase [Source:HGNC Symbol;Acc:HGNC:7643]	K01893	NARS, asnS
ENSP00000354610.4	amylase alpha 2B (pancreatic) [Source:HGNC Symbol;Acc:HGNC:478]	K01176	AMY, amyA, malS
ENSP00000440756.1	cytochrome c oxidase assembly factor 4 homolog [Source:HGNC Symbol;Acc:HGNC:24604]	K18177	COA4
ENSP00000285518.6	1-acylglycerol-3-phosphate O-acyltransferase 5 [Source:HGNC Symbol;Acc:HGNC:20886]	K19007	AGPAT5
ENSP00000389630.2	dpy-19 like 4 [Source:HGNC Symbol;Acc:HGNC:27829]		
ENSP00000265192.4	poly(A) binding protein interacting protein 2 [Source:HGNC Symbol;Acc:HGNC:17970]		
ENSP00000307508.4	calbindin 2 [Source:HGNC Symbol;Acc:HGNC:1435]		
ENSP00000222968.4	PDGFA associated protein 1 [Source:HGNC Symbol;Acc:HGNC:14634]		
ENSP00000372701.4	solute carrier family 39 member 7 [Source:HGNC Symbol;Acc:HGNC:4927]	K14713	SLC39A7, KE4, ZIP7
ENSP00000382250.2	mitochondrial ribosomal protein S6 [Source:HGNC Symbol;Acc:HGNC:14051]	K02990	RP-S6, MRPS6, rpsF
ENSP00000014930.4	heme binding protein 1 [Source:HGNC Symbol;Acc:HGNC:17176]		
ENSP00000290354.5	carbonyl reductase 3 [Source:HGNC Symbol;Acc:HGNC:1549]	K00084	CBR3
ENSP00000496942.1	jagunal homolog 1 [Source:HGNC Symbol;Acc:HGNC:26926]		
ENSP00000278951.7	SID1 transmembrane family member 2 [Source:HGNC Symbol;Acc:HGNC:24272]		
ENSP00000378887.3	dehydrogenase/reductase 7B [Source:HGNC Symbol;Acc:HGNC:24547]	K11166	DHRS7B
ENSP00000387239.1	polypeptide N-acetylgalactosaminyltransferase 13 [Source:HGNC Symbol;Acc:HGNC:23242]		
ENSP00000254816.1	tripartite motif containing 47 [Source:HGNC Symbol;Acc:HGNC:19020]	K12023	TRIM47
ENSP00000424183.1	late endosomal/lysosomal adaptor, MAPK and MTOR activator 3 [Source:HGNC Symbol;Acc:H(	K04370	LAMTOR3, MP1, MAP2
ENSP00000358727.5	glutathione S-transferase omega 1 [Source:HGNC Symbol;Acc:HGNC:13312]	K00799	GST, gst
ENSP00000053468.3	mitochondrial ribosomal protein S10 [Source:HGNC Symbol;Acc:HGNC:14502]	K02946	RP-S10, MRPS10, rpsJ
ENSP00000285379.4	carbonic anhydrase 2 [Source:HGNC Symbol;Acc:HGNC:1373]	K18245	CA2
ENSP00000438248.1	calumenin [Source:HGNC Symbol;Acc:HGNC:1458]		

ENSP00000379287.2	tyrosine 3-monooxygenase/tryptophan 5-monooxygenase activation protein zeta [Source:HG	K16197	YWHAB_Q_Z
ENSP00000356465.3	RAB32, member RAS oncogene family [Source:HGNC Symbol;Acc:HGNC:9772]	K07918	RAB32
ENSP00000349811.3	FKBP prolyl isomerase 5 [Source:HGNC Symbol;Acc:HGNC:3721]	K09571	FKBP4_5
ENSP00000416583.2	deoxyribose-phosphate aldolase [Source:HGNC Symbol;Acc:HGNC:24269]	K01619	deoC, DERA
ENSP00000214869.1	transmembrane p24 trafficking protein 1 [Source:HGNC Symbol;Acc:HGNC:17291]	K20348	TMED1
ENSP00000260363.4	kinesin family member 23 [Source:HGNC Symbol;Acc:HGNC:6392]	K17387	KIF23
ENSP00000404042.2	transmembrane p24 trafficking protein 4 [Source:HGNC Symbol;Acc:HGNC:22301]	K20346	TMED4_9_11
ENSP00000256496.3	ADP ribosylation factor like GTPase 8B [Source:HGNC Symbol;Acc:HGNC:25564]	K07955	ARL8
ENSP00000452603.1	proteasome subunit alpha 6 [Source:HGNC Symbol;Acc:HGNC:9535]		
ENSP00000432043.1	mitochondrial carrier 2 [Source:HGNC Symbol;Acc:HGNC:17587]		
ENSP00000476687.1	torsin 1A interacting protein 1 [Source:HGNC Symbol;Acc:HGNC:29456]		
ENSP00000272746.5	WAS/WASL interacting protein family member 1 [Source:HGNC Symbol;Acc:HGNC:12736]	K19475	WIPF
ENSP00000273986.4	CDGSH iron sulfur domain 2 [Source:HGNC Symbol;Acc:HGNC:24212]		
ENSP00000367299.3	vacuolar protein sorting 36 homolog [Source:HGNC Symbol;Acc:HGNC:20312]	K12190	VPS36, EAP45
ENSP00000304467.5	thimet oligopeptidase 1 [Source:HGNC Symbol;Acc:HGNC:11793]	K01392	THOP1
ENSP00000346340.2	COP9 signalosome subunit 8 [Source:HGNC Symbol;Acc:HGNC:24335]	K12181	COPS8, CSN8
ENSP00000425809.1	phosphoglucomutase 3 [Source:HGNC Symbol;Acc:HGNC:8907]	K01836	PGM3
ENSP00000358089.2	TIA1 cytotoxic granule associated RNA binding protein like 1 [Source:HGNC Symbol;Acc:HGNC:11804]		
ENSP00000307765.5	cytochrome b-245 chaperone 1 [Source:HGNC Symbol;Acc:HGNC:28672]		
ENSP00000311876.6	glucosamine-6-phosphate deaminase 1 [Source:HGNC Symbol;Acc:HGNC:4417]	K02564	nagB, GNPDA
ENSP00000466897.1	cold inducible RNA binding protein [Source:HGNC Symbol;Acc:HGNC:1982]		
ENSP00000325748.4	serine/threonine kinase 25 [Source:HGNC Symbol;Acc:HGNC:11404]	K08838	STK24_25_MST4
ENSP00000264265.3	latexin [Source:HGNC Symbol;Acc:HGNC:13347]		
ENSP00000443459.1	branched chain amino acid transaminase 1 [Source:HGNC Symbol;Acc:HGNC:976]	K00826	E2.6.1.42, ilvE
ENSP00000240922.2	N(alpha)-acetyltransferase 50, NatE catalytic subunit [Source:HGNC Symbol;Acc:HGNC:29533]	K20793	NAA50, NAT5
ENSP00000385746.2	spermine synthase [Source:HGNC Symbol;Acc:HGNC:11123]	K00802	SMS
ENSP00000382341.3	Rab geranylgeranyltransferase subunit alpha [Source:HGNC Symbol;Acc:HGNC:9795]	K14050	RABGGTA
ENSP00000406885.1	transmembrane protein 41A [Source:HGNC Symbol;Acc:HGNC:30544]		
ENSP00000316905.5	sideroflexin 1 [Source:HGNC Symbol;Acc:HGNC:16085]		
ENSP00000229179.4	nucleoporin 107 [Source:HGNC Symbol;Acc:HGNC:29914]	K14301	NUP107, NUP84
ENSP00000357283.4	lamin A/C [Source:HGNC Symbol;Acc:HGNC:6636]	K12641	LMNA
ENSP00000257895.5	retinol dehydrogenase 5 [Source:HGNC Symbol;Acc:HGNC:9940]	K00061	RDH5
ENSP00000216034.4	translocase of outer mitochondrial membrane 22 [Source:HGNC Symbol;Acc:HGNC:18002]	K17769	TOM22
ENSP00000354762.5	cytochrome c oxidase assembly factor 3 [Source:HGNC Symbol;Acc:HGNC:24990]	K18175	CCDC56, COA3
ENSP00000351646.5	stress induced phosphoprotein 1 [Source:HGNC Symbol;Acc:HGNC:11387]	K09553	STIP1
ENSP00000484686.1	glutathione S-transferase pi 1 [Source:HGNC Symbol;Acc:HGNC:4638]		
ENSP00000262428.4	coactosin like F-actin binding protein 1 [Source:HGNC Symbol;Acc:HGNC:18304]		
ENSP00000281513.5	neuroblastoma amplified sequence [Source:HGNC Symbol;Acc:HGNC:15625]	K20473	NBAS

ENSP00000361712.4	transcription elongation factor A like 4 [Source:HGNC Symbol;Acc:HGNC:26121]		
ENSP00000310670.4	cell cycle and apoptosis regulator 2 [Source:HGNC Symbol;Acc:HGNC:23360]		
ENSP00000401371.2	TIA1 cytotoxic granule associated RNA binding protein [Source:HGNC Symbol;Acc:HGNC:1180	K13201	TIA1, TIAL1
ENSP00000364037.4	testis expressed 10 [Source:HGNC Symbol;Acc:HGNC:25988]	K14827	IPI1, TEX10
ENSP00000253039.4	eukaryotic translation initiation factor 2 subunit gamma [Source:HGNC Symbol;Acc:HGNC:326	K03242	EIF2S3
ENSP00000348211.3	solute carrier family 25 member 46 [Source:HGNC Symbol;Acc:HGNC:25198]	K03454	SLC25A46
ENSP00000312599.5	transmembrane protein 70 [Source:HGNC Symbol;Acc:HGNC:26050]	K17966	TMEM70
ENSP00000352208.4	myoferlin [Source:HGNC Symbol;Acc:HGNC:3656]		
ENSP00000276062.8	NADH:ubiquinone oxidoreductase subunit B11 [Source:HGNC Symbol;Acc:HGNC:20372]		
ENSP00000388598.2	TAM41 mitochondrial translocator assembly and maintenance homolog [Source:HGNC Symbo	K17807	TAM41, MMP37
ENSP00000296292.3	RFT1 homolog [Source:HGNC Symbol;Acc:HGNC:30220]	K06316	RFT1
ENSP00000356902.1	UDP-N-acetylglucosamine pyrophosphorylase 1 [Source:HGNC Symbol;Acc:HGNC:12457]	K00972	UAP1
ENSP00000414398.1	DnaJ heat shock protein family (Hsp40) member B11 [Source:HGNC Symbol;Acc:HGNC:14889]	K09517	DNAJB11
ENSP00000313584.6	translocase of outer mitochondrial membrane 5 [Source:HGNC Symbol;Acc:HGNC:31369]	K17773	TOM5
ENSP00000218104.3	ATP binding cassette subfamily D member 1 [Source:HGNC Symbol;Acc:HGNC:61]	K05675	ABCD1, ALD
ENSP00000287295.3	apoptosis inducing factor mitochondria associated 1 [Source:HGNC Symbol;Acc:HGNC:8768]	K04727	PDCD8, AIF
ENSP00000499778.1	microtubule associated monooxygenase, calponin and LIM domain containing 2 [Source:HGN	K19947	MICAL
ENSP00000426120.1	GRAM domain containing 2B [Source:HGNC Symbol;Acc:HGNC:24911]		
ENSP00000420588.1	transcription factor A, mitochondrial [Source:HGNC Symbol;Acc:HGNC:11741]	K11830	TFAM, MTTFA
ENSP00000231512.3	chromosome 5 open reading frame 15 [Source:HGNC Symbol;Acc:HGNC:20656]		
ENSP00000181796.2	family with sequence similarity 107 member B [Source:HGNC Symbol;Acc:HGNC:23726]		
ENSP00000410992.2	mannosyl-oligosaccharide glucosidase [Source:HGNC Symbol;Acc:HGNC:24862]	K01228	GCS1
ENSP00000354468.4	cytochrome b5 reductase 3 [Source:HGNC Symbol;Acc:HGNC:2873]	K00326	E1.6.2.2
ENSP00000358039.3	arginyltransferase 1 [Source:HGNC Symbol;Acc:HGNC:782]		
ENSP00000287859.6	odr-4 GPCR localization factor homolog [Source:HGNC Symbol;Acc:HGNC:24299]		
ENSP00000315411.3	bridging integrator 1 [Source:HGNC Symbol;Acc:HGNC:1052]		
ENSP00000248975.5	tyrosine 3-monooxygenase/tryptophan 5-monooxygenase activation protein eta [Source:HGN	K16198	YWHAG_H
ENSP00000408005.2	SLC9A3 regulator 2 [Source:HGNC Symbol;Acc:HGNC:11076]	K13358	SLC9A3R2, NHERF2
ENSP00000371783.3	bone marrow stromal cell antigen 1 [Source:HGNC Symbol;Acc:HGNC:1118]		
ENSP00000223836.1	adenylate kinase 1 [Source:HGNC Symbol;Acc:HGNC:361]	K00939	adk, AK
ENSP00000496241.1	solute carrier family 33 member 1 [Source:HGNC Symbol;Acc:HGNC:95]	K03372	ACATN, SLC33A1
ENSP00000346762.3	staphylococcal nuclease and tudor domain containing 1 [Source:HGNC Symbol;Acc:HGNC:306	K15979	SND1
ENSP00000364886.2	Rho family GTPase 3 [Source:HGNC Symbol;Acc:HGNC:671]	K07859	RND3
ENSP00000450975.1	prostaglandin reductase 2 [Source:HGNC Symbol;Acc:HGNC:20149]	K13949	PTGR2, ZADH1
ENSP00000401639.2	phosphoserine phosphatase [Source:HGNC Symbol;Acc:HGNC:9577]	K01079	serB, PSPH
ENSP00000247665.1	phosphohistidine phosphatase 1 [Source:HGNC Symbol;Acc:HGNC:30033]	K01112	PHPT1
ENSP00000411532.1	DNA topoisomerase II alpha [Source:HGNC Symbol;Acc:HGNC:11989]	K03164	TOP2
ENSP00000300291.5	nudix hydrolase 21 [Source:HGNC Symbol;Acc:HGNC:13870]	K14397	NUDT21, CPSF5, CFIM

ENSP00000216602.6	zinc finger FYVE-type containing 21 [Source:HGNC Symbol;Acc:HGNC:20760]		
ENSP00000381104.2	transmembrane 9 superfamily member 4 [Source:HGNC Symbol;Acc:HGNC:30797]	K17086	TM9SF2_4
ENSP00000357697.4	S100 calcium binding protein A2 [Source:HGNC Symbol;Acc:HGNC:10492]		
ENSP00000334052.4	legumain [Source:HGNC Symbol;Acc:HGNC:9472]	K01369	LGMN
ENSP00000253023.2	ubiquitin conjugating enzyme E2 M [Source:HGNC Symbol;Acc:HGNC:12491]	K10579	UBE2M, UBC12
ENSP00000361602.4	zinc finger protein 503 [Source:HGNC Symbol;Acc:HGNC:23589]		
ENSP00000361600.1	small vasohibin binding protein [Source:HGNC Symbol;Acc:HGNC:29204]		
ENSP00000496470.1	novel protein		
ENSP00000483879.1	XK related 5 [Source:HGNC Symbol;Acc:HGNC:20782]		
ENSP00000302160.3	LSM3 homolog, U6 small nuclear RNA and mRNA degradation associated [Source:HGNC Symb	K12622	LSM3
ENSP00000261700.3	RNA transcription, translation and transport factor [Source:HGNC Symbol;Acc:HGNC:23169]	K15433	CGI99, CLE7, RLLM1
ENSP00000396673.2	FtsJ RNA 2'-O-methyltransferase 3 [Source:HGNC Symbol;Acc:HGNC:17136]	K14857	SPB1, FTSJ3
ENSP00000484736.1	transmembrane protein 120A [Source:HGNC Symbol;Acc:HGNC:21697]		
ENSP00000480941.1	chromosome 18 open reading frame 32 [Source:HGNC Symbol;Acc:HGNC:31690]		
ENSP00000495612.1	male-enhanced antigen 1 [Source:HGNC Symbol;Acc:HGNC:6986]		
ENSP00000304408.3	collagen type III alpha 1 chain [Source:HGNC Symbol;Acc:HGNC:2201]	K19720	COL3A
ENSP00000222693.4	caveolin 2 [Source:HGNC Symbol;Acc:HGNC:1528]	K12958	CAV2
ENSP00000248378.4	ER membrane protein complex subunit 6 [Source:HGNC Symbol;Acc:HGNC:28430]		
ENSP00000367265.4	cytoskeleton associated protein 4 [Source:HGNC Symbol;Acc:HGNC:16991]	K13999	CKAP4, CLIMP63
ENSP00000317780.6	cytochrome c oxidase subunit 5A [Source:HGNC Symbol;Acc:HGNC:2267]	K02264	COX5A
ENSP00000316029.9	talin 1 [Source:HGNC Symbol;Acc:HGNC:11845]	K06271	TLN
ENSP00000262139.4	WD repeat domain, phosphoinositide interacting 1 [Source:HGNC Symbol;Acc:HGNC:25471]	K17908	WIPI, ATG18
ENSP00000319992.5	transmembrane protein 11 [Source:HGNC Symbol;Acc:HGNC:16823]		
ENSP00000337773.2	N-ribosyldihydronicotinamide:quinone reductase 2 [Source:HGNC Symbol;Acc:HGNC:7856]	K08071	NQO2
ENSP00000494022.1	glutamate-ammonia ligase [Source:HGNC Symbol;Acc:HGNC:4341]	K01915	glnA, GLUL
ENSP00000384949.2	pyrroline-5-carboxylate reductase 1 [Source:HGNC Symbol;Acc:HGNC:9721]	K00286	proC
ENSP00000351157.4	thioredoxin domain containing 15 [Source:HGNC Symbol;Acc:HGNC:20652]		
ENSP00000305138.4	MAPK regulated corepressor interacting protein 2 [Source:HGNC Symbol;Acc:HGNC:14142]		
ENSP00000498855.1	calcyphosine [Source:HGNC Symbol;Acc:HGNC:1487]		
ENSP00000263655.3	cannabinoid receptor interacting protein 1 [Source:HGNC Symbol;Acc:HGNC:24546]		
ENSP00000305647.4	vesicle associated membrane protein 5 [Source:HGNC Symbol;Acc:HGNC:12646]	K08514	VAMP5
ENSP00000382281.3	death domain associated protein [Source:HGNC Symbol;Acc:HGNC:2681]	K02308	DAXX
ENSP00000346564.4	tumor protein p53 inducible protein 11 [Source:HGNC Symbol;Acc:HGNC:16842]		
ENSP00000231368.5	leucyl and cystinyl aminopeptidase [Source:HGNC Symbol;Acc:HGNC:6656]	K01257	LNPEP
ENSP00000363714.3	transmembrane protein 245 [Source:HGNC Symbol;Acc:HGNC:1363]		
ENSP00000498110.1	forkhead box P1 [Source:HGNC Symbol;Acc:HGNC:3823]		
ENSP00000477443.1	LSM4 homolog, U6 small nuclear RNA and mRNA degradation associated [Source:HGNC Symbol;Acc:HGNC:17259]		
ENSP00000314193.4	WD repeat domain 75 [Source:HGNC Symbol;Acc:HGNC:25725]	K14552	NAN1, UTP17, WDR75

ENSP00000380880.2	solute carrier family 66 member 2 [Source:HGNC Symbol;Acc:HGNC:26188]		
ENSP00000340989.4	stratifin [Source:HGNC Symbol;Acc:HGNC:10773]	K06644	SFN
ENSP00000422942.1	retinoic acid induced 14 [Source:HGNC Symbol;Acc:HGNC:14873]		
ENSP00000000412.3	mannose-6-phosphate receptor, cation dependent [Source:HGNC Symbol;Acc:HGNC:6752]	K10089	M6PR
ENSP00000280551.6	SEC24 homolog D, COPII coat complex component [Source:HGNC Symbol;Acc:HGNC:10706]	K14007	SEC24
ENSP00000423822.1	glutathione peroxidase 8 (putative) [Source:HGNC Symbol;Acc:HGNC:33100]	K00432	gpx
ENSP00000446252.1	superoxide dismutase 2 [Source:HGNC Symbol;Acc:HGNC:11180]	K04564	SOD2
ENSP00000352091.5	nucleoporin 98 [Source:HGNC Symbol;Acc:HGNC:8068]	K14297	NUP98, ADAR2, NUP1[illegible]
ENSP00000257177.4	terminal uridylyl transferase 4 [Source:HGNC Symbol;Acc:HGNC:28981]	K13291	TUT
ENSP00000363435.4	inositol 1,4,5-trisphosphate receptor type 3 [Source:HGNC Symbol;Acc:HGNC:6182]	K04960	ITPR3
ENSP00000200639.4	lysosomal associated membrane protein 2 [Source:HGNC Symbol;Acc:HGNC:6501]	K06528	LAMP1_2, CD107
ENSP00000478171.1	ubiquitin like with PHD and ring finger domains 1 [Source:HGNC Symbol;Acc:HGNC:12556]	K10638	UHRF1, NP95
ENSP00000266544.5	NADH:ubiquinone oxidoreductase subunit A9 [Source:HGNC Symbol;Acc:HGNC:7693]	K03953	NDUFA9
ENSP00000361331.2	ERI1 exoribonuclease family member 3 [Source:HGNC Symbol;Acc:HGNC:17276]	K18418	ERI3, PINT1
ENSP00000386597.2	dolichyl-phosphate N-acetylglucosaminephosphotransferase 1 [Source:HGNC Symbol;Acc:HG	K01001	ALG7
ENSP00000337513.5	steroid receptor RNA activator 1 [Source:HGNC Symbol;Acc:HGNC:11281]		
ENSP00000350757.3	glia maturation factor beta [Source:HGNC Symbol;Acc:HGNC:4373]		
ENSP00000366927.3	aldehyde dehydrogenase 1 family member B1 [Source:HGNC Symbol;Acc:HGNC:407]	K00128	ALDH
ENSP00000223864.2	plasminogen receptor with a C-terminal lysine [Source:HGNC Symbol;Acc:HGNC:23633]		
ENSP00000495347.1	tropomyosin 4 [Source:HGNC Symbol;Acc:HGNC:12013]	K10375	TPM4
ENSP00000500484.1	lactate dehydrogenase B [Source:HGNC Symbol;Acc:HGNC:6541]	K00016	LDH, ldh
ENSP00000364119.2	eukaryotic translation initiation factor 2 subunit beta [Source:HGNC Symbol;Acc:HGNC:3266]	K03238	EIF2S2
ENSP00000314036.4	1-acylglycerol-3-phosphate O-acyltransferase 4 [Source:HGNC Symbol;Acc:HGNC:20885]	K13523	AGPAT3_4
ENSP00000353224.4	transferrin receptor [Source:HGNC Symbol;Acc:HGNC:11763]	K06503	TFRC, CD71
ENSP00000411701.1	drebrin like [Source:HGNC Symbol;Acc:HGNC:2696]	K20520	DBNL, ABP1
ENSP00000292114.3	transmembrane protein 199 [Source:HGNC Symbol;Acc:HGNC:18085]		
ENSP00000408827.2	MAPK regulated corepressor interacting protein 1 [Source:HGNC Symbol;Acc:HGNC:28007]		
ENSP00000404381.2	DnaJ heat shock protein family (Hsp40) member B14 [Source:HGNC Symbol;Acc:HGNC:25881]	K09520	DNAJB14
ENSP00000371532.2	very low density lipoprotein receptor [Source:HGNC Symbol;Acc:HGNC:12698]	K20053	VLDLR
ENSP00000369785.5	pirin [Source:HGNC Symbol;Acc:HGNC:30048]	K06911	K06911
ENSP00000498872.1	sorting nexin 6 [Source:HGNC Symbol;Acc:HGNC:14970]	K17920	SNX5_6_32
ENSP00000350012.3	acyl-CoA synthetase long chain family member 3 [Source:HGNC Symbol;Acc:HGNC:3570]	K01897	ACSL, fadD
ENSP00000348093.5	heterogeneous nuclear ribonucleoprotein A/B [Source:HGNC Symbol;Acc:HGNC:5034]		
ENSP00000355746.1	presenilin 2 [Source:HGNC Symbol;Acc:HGNC:9509]	K04522	PSEN2, PS2
ENSP00000345848.4	acidic nuclear phosphoprotein 32 family member B [Source:HGNC Symbol;Acc:HGNC:16677]	K18647	ANP32B
ENSP00000484569.1	proteasome activator subunit 2 [Source:HGNC Symbol;Acc:HGNC:9569]		
ENSP00000263036.3	optineurin [Source:HGNC Symbol;Acc:HGNC:17142]	K19946	OPTN, FIP2
ENSP00000265605.2	aldehyde dehydrogenase 8 family member A1 [Source:HGNC Symbol;Acc:HGNC:15471]		

ENSP00000490149.1	aminoacylase 1 [Source:HGNC Symbol;Acc:HGNC:177]	K14677	ACY1
ENSP00000220764.2	2,4-dienoyl-CoA reductase 1 [Source:HGNC Symbol;Acc:HGNC:2753]	K13236	DECR1
ENSP00000216117.8	heme oxygenase 1 [Source:HGNC Symbol;Acc:HGNC:5013]	K00510	HMOX1
ENSP00000373364.3	golgi membrane protein 1 [Source:HGNC Symbol;Acc:HGNC:15451]		
ENSP00000395590.2	interferon induced protein 35 [Source:HGNC Symbol;Acc:HGNC:5399]		
ENSP00000297268.6	collagen type I alpha 2 chain [Source:HGNC Symbol;Acc:HGNC:2198]	K06236	COL1A
ENSP00000255324.5	ring finger protein 17 [Source:HGNC Symbol;Acc:HGNC:10060]	K18405	TDRD1_4_6_7
ENSP00000357835.5	phospholysine phosphohistidine inorganic pyrophosphate phosphatase [Source:HGNC Symbo	K11725	LHPP
ENSP00000369538.4	GDP dissociation inhibitor 2 [Source:HGNC Symbol;Acc:HGNC:4227]	K17255	GDI1_2
ENSP00000353284.4	DEAD-box helicase 3 Y-linked [Source:HGNC Symbol;Acc:HGNC:2699]	K17642	DDX3Y
ENSP00000427687.1	WD repeat domain 1 [Source:HGNC Symbol;Acc:HGNC:12754]		
ENSP00000265748.2	anillin actin binding protein [Source:HGNC Symbol;Acc:HGNC:14082]	K18621	ANLN
ENSP00000422607.1	acyl-CoA synthetase long chain family member 1 [Source:HGNC Symbol;Acc:HGNC:3569]	K01897	ACSL, fadD
ENSP00000409581.2	arrestin beta 1 [Source:HGNC Symbol;Acc:HGNC:711]	K04439	ARRB
ENSP00000362466.1	secreted phosphoprotein 2 [Source:HGNC Symbol;Acc:HGNC:11256]		
ENSP00000360305.3	PDZ and LIM domain 1 [Source:HGNC Symbol;Acc:HGNC:2067]		
ENSP00000345282.4	glutamic--pyruvic transaminase 2 [Source:HGNC Symbol;Acc:HGNC:18062]	K00814	GPT, ALT
ENSP00000316176.2	ubiquitin conjugating enzyme E2 N [Source:HGNC Symbol;Acc:HGNC:12492]	K10580	UBE2N, BLU, UBC13
ENSP00000366179.3	DnaJ heat shock protein family (Hsp40) member C1 [Source:HGNC Symbol;Acc:HGNC:20090]	K09521	DNAJC1
ENSP00000478813.1	GTP binding protein 8 (putative) [Source:HGNC Symbol;Acc:HGNC:25007]		
ENSP00000366798.1	high mobility group nucleosomal binding domain 4 [Source:HGNC Symbol;Acc:HGNC:4989]	K11302	HMGN4
ENSP00000266304.4	TEF transcription factor, PAR bZIP family member [Source:HGNC Symbol;Acc:HGNC:11722]	K09058	TEF
ENSP00000430236.1	ALG11 alpha-1,2-mannosyltransferase [Source:HGNC Symbol;Acc:HGNC:32456]	K03844	ALG11
ENSP00000256441.4	mitochondrial ribosomal protein S36 [Source:HGNC Symbol;Acc:HGNC:16631]	K17414	MRPS36
ENSP00000367309.4	monoamine oxidase B [Source:HGNC Symbol;Acc:HGNC:6834]	K00274	MAO, aofH
ENSP00000252599.3	collagen beta(1-O)galactosyltransferase 1 [Source:HGNC Symbol;Acc:HGNC:26182]	K11703	GLT25D
ENSP00000265174.4	3'-phosphoadenosine 5'-phosphosulfate synthase 1 [Source:HGNC Symbol;Acc:HGNC:8603]	K13811	PAPSS
ENSP00000263246.3	protein kinase C and casein kinase substrate in neurons 2 [Source:HGNC Symbol;Acc:HGNC:85	K20123	PACSIN
ENSP00000362060.3	NADH:ubiquinone oxidoreductase subunit S5 [Source:HGNC Symbol;Acc:HGNC:7712]	K03938	NDUFS5
ENSP00000464383.1	novel protein	K13504	VAMP2
ENSP00000216330.3	FKBP prolyl isomerase 3 [Source:HGNC Symbol;Acc:HGNC:3719]	K09570	FKBP3
ENSP00000369176.3	NADH:ubiquinone oxidoreductase subunit B6 [Source:HGNC Symbol;Acc:HGNC:7701]	K03962	NDUFB6
ENSP00000367939.3	ubiquinol-cytochrome c reductase complex III subunit VII [Source:HGNC Symbol;Acc:HGNC:2[illegible]	K00418	QCR8, UQCRQ
ENSP00000478755.1	SEC14 like lipid binding 2 [Source:HGNC Symbol;Acc:HGNC:10699]		
ENSP00000334983.5	pyruvate kinase M1/2 [Source:HGNC Symbol;Acc:HGNC:9021]	K00873	PK, pyk
ENSP00000262374.4	ALG1 chitobiosyldiphosphodolichol beta-mannosyltransferase [Source:HGNC Symbol;Acc:HG[illegible]	K03842	ALG1
ENSP00000308021.7	centrosomal protein 290 [Source:HGNC Symbol;Acc:HGNC:29021]	K16533	CEP290, NPHP6
ENSP00000446828.1	3'(2'), 5'-bisphosphate nucleotidase 1 [Source:HGNC Symbol;Acc:HGNC:1096]	K01082	cysQ, MET22, BPNT1

ENSP00000353846.3	doublecortin like kinase 1 [Source:HGNC Symbol;Acc:HGNC:2700]	K08805	DCLK1_2
ENSP00000478863.1	GON7 subunit of KEOPS complex [Source:HGNC Symbol;Acc:HGNC:20356]		
ENSP00000244534.5	histone cluster 1 H1 family member d [Source:HGNC Symbol;Acc:HGNC:4717]	K11275	H1_5
ENSP00000342492.6	parvin beta [Source:HGNC Symbol;Acc:HGNC:14653]	K06275	PARV
ENSP00000402608.2	carbamoyl-phosphate synthase 1 [Source:HGNC Symbol;Acc:HGNC:2323]	K01948	CPS1
ENSP00000499734.1	novel protein	K12893	SFRS4_5_6
ENSP00000269321.7	Rho GDP dissociation inhibitor alpha [Source:HGNC Symbol;Acc:HGNC:678]	K12462	ARHGDI, RHOGDI
ENSP00000300289.5	protein disulfide isomerase family A member 3 [Source:HGNC Symbol;Acc:HGNC:4606]	K08056	PDIA3, GRP58
ENSP00000370744.3	inositol 1,4,5-trisphosphate receptor type 2 [Source:HGNC Symbol;Acc:HGNC:6181]	K04959	ITPR2
ENSP00000321389.5	histone cluster 1 H2A family member c [Source:HGNC Symbol;Acc:HGNC:4733]	K11251	H2A
ENSP00000317379.3	glutaminase [Source:HGNC Symbol;Acc:HGNC:4331]	K01425	glsA, GLS
ENSP00000420682.1	queuine tRNA-ribosyltransferase accessory subunit 2 [Source:HGNC Symbol;Acc:HGNC:25771]	K15407	QTRTD1
ENSP00000249442.6	metaxin 2 [Source:HGNC Symbol;Acc:HGNC:7506]	K17776	MTX
ENSP00000362110.4	splicing factor 3a subunit 3 [Source:HGNC Symbol;Acc:HGNC:10767]	K12827	SF3A3, SAP61, PRP9
ENSP00000316809.7	phosphatidylinositol transfer protein alpha [Source:HGNC Symbol;Acc:HGNC:9001]		
ENSP00000400168.1	ATP5MF-PTCD1 readthrough [Source:HGNC Symbol;Acc:HGNC:38844]	K02130;	ATPeF0F, ATP5J2;PTCD
ENSP00000340454.5	Rap1 GTPase-GDP dissociation stimulator 1 [Source:HGNC Symbol;Acc:HGNC:9859]		
ENSP00000358939.4	seryl-tRNA synthetase [Source:HGNC Symbol;Acc:HGNC:10537]		
ENSP00000293760.5	LEM domain containing 2 [Source:HGNC Symbol;Acc:HGNC:21244]		
ENSP00000356860.1	microsomal glutathione S-transferase 3 [Source:HGNC Symbol;Acc:HGNC:7064]	K00799	GST, gst
ENSP00000359859.3	mitochondrial ribosome associated GTPase 2 [Source:HGNC Symbol;Acc:HGNC:16239]	K03979	obgE, cgtA
ENSP00000497283.1	gap junction protein alpha 1 [Source:HGNC Symbol;Acc:HGNC:4274]	K07372	GJA1, CX43
ENSP00000482396.1	novel protein	K03846	ALG9
ENSP00000428489.1	PDZ binding kinase [Source:HGNC Symbol;Acc:HGNC:18282]		
ENSP00000306330.3	tyrosine 3-monooxygenase/tryptophan 5-monooxygenase activation protein gamma [Source:	K16198	YWHAG_H
ENSP00000370192.4	solute carrier family 46 member 3 [Source:HGNC Symbol;Acc:HGNC:27501]	K20840	SLC46A3
ENSP00000258301.5	syntaxin 6 [Source:HGNC Symbol;Acc:HGNC:11441]	K08498	STX6
ENSP00000297540.4	phosphorylated adaptor for RNA export [Source:HGNC Symbol;Acc:HGNC:10241]	K14291	PHAX
ENSP00000357838.5	ornithine aminotransferase [Source:HGNC Symbol;Acc:HGNC:8091]	K00819	rocD, OAT
ENSP00000379902.3	transmembrane protein 106B [Source:HGNC Symbol;Acc:HGNC:22407]		
ENSP00000318147.7	ER degradation enhancing alpha-mannosidase like protein 3 [Source:HGNC Symbol;Acc:HGN(	K10086	EDEM3
ENSP00000349428.4	polypyrimidine tract binding protein 1 [Source:HGNC Symbol;Acc:HGNC:9583]		
ENSP00000370527.4	regulator of calcineurin 1 [Source:HGNC Symbol;Acc:HGNC:3040]	K17901	RCAN1, MCIP1
ENSP00000399124.1	ERGIC and golgi 3 [Source:HGNC Symbol;Acc:HGNC:15927]		
ENSP00000418493.1	SEC61 translocon alpha 1 subunit [Source:HGNC Symbol;Acc:HGNC:18276]	K10956	SEC61A
ENSP00000296503.5	high mobility group box 2 [Source:HGNC Symbol;Acc:HGNC:5000]	K11295	HMGB2
ENSP00000246166.2	farnesyltransferase, CAAX box, beta [Source:HGNC Symbol;Acc:HGNC:3785]	K05954	FNTB
ENSP00000315644.1	thymidylate synthetase [Source:HGNC Symbol;Acc:HGNC:12441]	K00560	thyA, TYMS

ENSP00000263640.3	activin A receptor type 1 [Source:HGNC Symbol;Acc:HGNC:171]	K04675	ACVR1, ALK2
ENSP00000373783.3	lysyl oxidase like 2 [Source:HGNC Symbol;Acc:HGNC:6666]	K00280	LOXL2_3_4
ENSP00000274938.7	signal peptide, CUB domain and EGF like domain containing 3 [Source:HGNC Symbol;Acc:HGNC:13655]		
ENSP00000370114.4	PC4 and SFRS1 interacting protein 1 [Source:HGNC Symbol;Acc:HGNC:9527]		
ENSP00000306887.2	transmembrane protein 126A [Source:HGNC Symbol;Acc:HGNC:25382]	K18157	TMEM126A
ENSP00000426387.1	zinc finger and BTB domain containing 38 [Source:HGNC Symbol;Acc:HGNC:26636]	K10510	ZBTB38
ENSP00000343742.5	BRO1 domain and CAAX motif containing [Source:HGNC Symbol;Acc:HGNC:26512]		
ENSP00000056217.5	Rho guanine nucleotide exchange factor 5 [Source:HGNC Symbol;Acc:HGNC:13209]	K20684	ARHGEF5
ENSP00000387654.2	prenylcysteine oxidase 1 [Source:HGNC Symbol;Acc:HGNC:20588]	K05906	PCYOX1, FCLY
ENSP00000458504.1	solute carrier family 27 member 3 [Source:HGNC Symbol;Acc:HGNC:10997]	K08772	SLC27A3, FATP3
ENSP00000219439.4	hydroxysteroid dehydrogenase like 1 [Source:HGNC Symbol;Acc:HGNC:16475]		
ENSP00000442318.1	cullin associated and neddylation dissociated 1 [Source:HGNC Symbol;Acc:HGNC:30688]	K17263	CAND1
ENSP00000236192.7	vesicle associated membrane protein 4 [Source:HGNC Symbol;Acc:HGNC:12645]	K08513	VAMP4
ENSP00000281623.3	F-box protein 4 [Source:HGNC Symbol;Acc:HGNC:13583]	K10291	FBXO4
ENSP00000248553.6	heat shock protein family B (small) member 1 [Source:HGNC Symbol;Acc:HGNC:5246]	K04455	HSPB1
ENSP00000330945.6	transmembrane p24 trafficking protein 9 [Source:HGNC Symbol;Acc:HGNC:24878]	K20346	TMED4_9_11
ENSP00000348299.4	tripartite motif containing 13 [Source:HGNC Symbol;Acc:HGNC:9976]	K12003	TRIM13
ENSP00000303659.8	RANBP2 like and GRIP domain containing 3 [Source:HGNC Symbol;Acc:HGNC:32416]	K12172	RANBP2, NUP358
ENSP00000315386.5	protein O-glucosyltransferase 3 [Source:HGNC Symbol;Acc:HGNC:28496]		
ENSP00000229922.2	cyclase associated actin cytoskeleton regulatory protein 2 [Source:HGNC Symbol;Acc:HGNC:2(	K17261	CAP1_2, SRV2
ENSP00000215565.1	NADH:ubiquinone oxidoreductase subunit B7 [Source:HGNC Symbol;Acc:HGNC:7702]	K03963	NDUFB7
ENSP00000425952.2	hydroxyacyl-CoA dehydrogenase [Source:HGNC Symbol;Acc:HGNC:4799]		
ENSP00000388658.2	solute carrier family 25 member 12 [Source:HGNC Symbol;Acc:HGNC:10982]	K15105	SLC25A12_13, AGC
ENSP00000493515.1	transmembrane 9 superfamily member 2 [Source:HGNC Symbol;Acc:HGNC:11865]	K17086	TM9SF2_4
ENSP00000497016.1	signal sequence receptor subunit 1 [Source:HGNC Symbol;Acc:HGNC:11323]	K13249	SSR1
ENSP00000481741.1	glutathione S-transferase theta 1 [Source:HGNC Symbol;Acc:HGNC:4641]	K00799	GST, gst
ENSP00000359050.4	armadillo like helical domain containing 3 [Source:HGNC Symbol;Acc:HGNC:25788]		
ENSP00000287022.5	ubiquinol-cytochrome c reductase binding protein [Source:HGNC Symbol;Acc:HGNC:12582]	K00417	QCR7, UQCRB
ENSP00000253004.6	argininosuccinate synthase 1 [Source:HGNC Symbol;Acc:HGNC:758]	K01940	argG, ASS1
ENSP00000402756.1	nuclear receptor 2C2 associated protein [Source:HGNC Symbol;Acc:HGNC:30763]		
ENSP00000368664.3	regulator of cell cycle [Source:HGNC Symbol;Acc:HGNC:20369]		
ENSP00000398350.2	chromosome 11 open reading frame 68 [Source:HGNC Symbol;Acc:HGNC:28801]		
ENSP00000253110.1	shiftless antiviral inhibitor of ribosomal frameshifting [Source:HGNC Symbol;Acc:HGNC:25649]		
ENSP00000262982.2	chromosome segregation 1 like [Source:HGNC Symbol;Acc:HGNC:2431]	K18423	CSE1, CAS, XPO2
ENSP00000362820.5	serine and arginine rich splicing factor 3 [Source:HGNC Symbol;Acc:HGNC:10785]	K12892	SFRS3
ENSP00000216500.5	dehydrogenase/reductase 7 [Source:HGNC Symbol;Acc:HGNC:21524]	K11165	DHRS7
ENSP00000272252.5	galactose mutarotase [Source:HGNC Symbol;Acc:HGNC:24063]	K01785	galM, GALM
ENSP00000324248.3	proenkephalin [Source:HGNC Symbol;Acc:HGNC:8831]	K18832	PENK

ENSP00000253413.5	ATPase H+ transporting V1 subunit E1 [Source:HGNC Symbol;Acc:HGNC:857]	K02150	ATPeV1E, ATP6E
ENSP00000289371.5	eukaryotic translation initiation factor 5B [Source:HGNC Symbol;Acc:HGNC:30793]	K03243	EIF5B
ENSP00000259253.6	UDP-glucose glycoprotein glucosyltransferase 1 [Source:HGNC Symbol;Acc:HGNC:15663]	K11718	HUGT
ENSP00000435295.1	EGF containing fibulin extracellular matrix protein 2 [Source:HGNC Symbol;Acc:HGNC:3219]	K19866	EFEMP2
ENSP00000487640.1	tissue specific transplantation antigen P35B [Source:HGNC Symbol;Acc:HGNC:12390]	K02377	TSTA3, fcl
ENSP00000470972.1	ribosomal protein S19 [Source:HGNC Symbol;Acc:HGNC:10402]	K02966	RP-S19e, RPS19
ENSP00000268379.4	ubiquinol-cytochrome c reductase core protein 2 [Source:HGNC Symbol;Acc:HGNC:12586]	K00415	QCR2, UQCRC2
ENSP00000467414.1	mahogunin ring finger 1 [Source:HGNC Symbol;Acc:HGNC:20254]	K10604	MGRN1
ENSP00000422440.1	2'-deoxynucleoside 5'-phosphate N-hydrolase 1 [Source:HGNC Symbol;Acc:HGNC:21218]		
ENSP00000361635.1	voltage dependent anion channel 2 [Source:HGNC Symbol;Acc:HGNC:12672]	K15040	VDAC2
ENSP00000264156.2	minichromosome maintenance complex component 6 [Source:HGNC Symbol;Acc:HGNC:6949]	K02542	MCM6
ENSP00000261942.6	Fas associated factor family member 2 [Source:HGNC Symbol;Acc:HGNC:24666]	K18726	FAF2, UBXD8
ENSP00000365049.1	acyl-CoA binding domain containing 5 [Source:HGNC Symbol;Acc:HGNC:23338]		
ENSP00000315774.5	NADH:ubiquinone oxidoreductase core subunit S8 [Source:HGNC Symbol;Acc:HGNC:7715]	K03941	NDUFS8
ENSP00000288680.4	signal peptide peptidase like 3 [Source:HGNC Symbol;Acc:HGNC:30424]	K09598	SPPL3
ENSP00000391509.2	cutA divalent cation tolerance homolog [Source:HGNC Symbol;Acc:HGNC:21101]	K03926	cutA
ENSP00000481886.1	progesterone receptor membrane component 2 [Source:HGNC Symbol;Acc:HGNC:16089]	K17278	PGRMC1_2
ENSP00000248598.5	fibrinogen like 2 [Source:HGNC Symbol;Acc:HGNC:3696]		
ENSP00000356433.4	uronyl 2-sulfotransferase [Source:HGNC Symbol;Acc:HGNC:17223]	K03193	UST
ENSP00000340900.5	MIA SH3 domain ER export factor 3 [Source:HGNC Symbol;Acc:HGNC:24008]		
ENSP00000482336.1	cysteinyl-tRNA synthetase [Source:HGNC Symbol;Acc:HGNC:1493]	K01883	CARS, cysS
ENSP00000409909.1	Sad1 and UNC84 domain containing 1 [Source:HGNC Symbol;Acc:HGNC:18587]	K19347	SUN1_2
ENSP00000392028.1	chromodomain helicase DNA binding protein 7 [Source:HGNC Symbol;Acc:HGNC:20626]	K14437	CHD7
ENSP00000339324.3	small integral membrane protein 15 [Source:HGNC Symbol;Acc:HGNC:33861]		
ENSP00000376620.2	tryptophanyl-tRNA synthetase [Source:HGNC Symbol;Acc:HGNC:12729]	K01867	WARS, trpS
ENSP00000388942.1	secernin 1 [Source:HGNC Symbol;Acc:HGNC:22192]	K14358	SCRN
ENSP00000360268.2	aldehyde dehydrogenase 18 family member A1 [Source:HGNC Symbol;Acc:HGNC:9722]	K12657	ALDH18A1, P5CS
ENSP00000454646.1	solute carrier family 38 member 7 [Source:HGNC Symbol;Acc:HGNC:25582]	K14994	SLC38A7_8
ENSP00000442266.1	DEAD-box helicase 6 [Source:HGNC Symbol;Acc:HGNC:2747]	K12614	DDX6, RCK, DHH1
ENSP00000343552.5	isoprenylcysteine carboxyl methyltransferase [Source:HGNC Symbol;Acc:HGNC:5350]	K00587	ICMT, STE14
ENSP00000278505.4	endonuclease domain containing 1 [Source:HGNC Symbol;Acc:HGNC:29129]	K15049	ENDOD1
ENSP00000370808.5	solute carrier family 25 member 6 [Source:HGNC Symbol;Acc:HGNC:10992]	K05863	SLC25A4S, ANT
ENSP00000264501.4	KIAA1109 [Source:HGNC Symbol;Acc:HGNC:26953]		
ENSP00000440222.1	FMC1-LUC7L2 readthrough [Source:HGNC Symbol;Acc:HGNC:44671]	K13212	LUC7L2
ENSP00000273375.3	RAB, member of RAS oncogene family like 3 [Source:HGNC Symbol;Acc:HGNC:18072]	K07933	RABL3
ENSP00000448083.1	two pore segment channel 1 [Source:HGNC Symbol;Acc:HGNC:18182]	K16896	TPCN1
ENSP00000293350.3	aldehyde dehydrogenase 16 family member A1 [Source:HGNC Symbol;Acc:HGNC:28114]		
ENSP00000385332.1	BSCL2 lipid droplet biogenesis associated, seipin [Source:HGNC Symbol;Acc:HGNC:15832]	K19365	BSCL2

ENSP00000333255.6	vacuolar ATPase assembly factor VMA21 [Source:HGNC Symbol;Acc:HGNC:22082]		
ENSP00000271843.4	jumping translocation breakpoint [Source:HGNC Symbol;Acc:HGNC:6201]		
ENSP00000361087.3	synuclein gamma [Source:HGNC Symbol;Acc:HGNC:11141]		
ENSP00000387263.1	translin [Source:HGNC Symbol;Acc:HGNC:12379]		
ENSP00000297185.3	heat shock protein family A (Hsp70) member 9 [Source:HGNC Symbol;Acc:HGNC:5244]	K04043	dnaK
ENSP00000346890.7	nardilysin convertase [Source:HGNC Symbol;Acc:HGNC:7995]	K01411	NRD1
ENSP00000259339.2	torsin family 1 member B [Source:HGNC Symbol;Acc:HGNC:11995]		
ENSP00000366280.4	lectin, mannose binding 2 like [Source:HGNC Symbol;Acc:HGNC:19263]		
ENSP00000341625.4	cytochrome b5 type A [Source:HGNC Symbol;Acc:HGNC:2570]		
ENSP00000377793.3	protein regulator of cytokinesis 1 [Source:HGNC Symbol;Acc:HGNC:9341]	K16732	PRC1
ENSP00000346437.3	autophagy related 7 [Source:HGNC Symbol;Acc:HGNC:16935]	K08337	ATG7
ENSP00000237889.4	NADH:ubiquinone oxidoreductase subunit B3 [Source:HGNC Symbol;Acc:HGNC:7698]	K03959	NDUFB3
ENSP00000285667.3	heat shock protein family A (Hsp70) member 13 [Source:HGNC Symbol;Acc:HGNC:11375]	K09491	STCH
ENSP00000387065.1	calmodulin 2 [Source:HGNC Symbol;Acc:HGNC:1445]	K02183	CALM
ENSP00000305810.2	syntaxin 18 [Source:HGNC Symbol;Acc:HGNC:15942]	K08492	STX18
ENSP00000246337.4	uroporphyrinogen decarboxylase [Source:HGNC Symbol;Acc:HGNC:12591]	K01599	hemE, UROD
ENSP00000434724.1	coiled-coil domain containing 90B [Source:HGNC Symbol;Acc:HGNC:28108]		
ENSP00000451979.1	apurinic/apyrimidinic endodeoxyribonuclease 1 [Source:HGNC Symbol;Acc:HGNC:587]	K10771	APEX1
ENSP00000064778.4	family with sequence similarity 168 member A [Source:HGNC Symbol;Acc:HGNC:28999]		
ENSP00000253452.2	cytochrome c oxidase subunit 4I1 [Source:HGNC Symbol;Acc:HGNC:2265]	K02263	COX4
ENSP00000374113.4	parathymosin [Source:HGNC Symbol;Acc:HGNC:9629]		
ENSP00000227638.3	pannexin 1 [Source:HGNC Symbol;Acc:HGNC:8599]	K03443	PANX1
ENSP00000367992.3	esterase D [Source:HGNC Symbol;Acc:HGNC:3465]	K01070	frmB, ESD, fghA
ENSP00000429150.1	family with sequence similarity 49 member B [Source:HGNC Symbol;Acc:HGNC:25216]		
ENSP00000219789.6	CDP-diacylglycerol--inositol 3-phosphatidyltransferase [Source:HGNC Symbol;Acc:HGNC:176	K00999	CDIPT
ENSP00000487984.1	nucleosome assembly protein 1 like 4 [Source:HGNC Symbol;Acc:HGNC:7640]	K11282	NAP1L4, NAP2
ENSP00000386651.1	multiple coagulation factor deficiency 2 [Source:HGNC Symbol;Acc:HGNC:18451]	K20364	MCFD2
ENSP00000366819.3	ubiquitin C-terminal hydrolase L3 [Source:HGNC Symbol;Acc:HGNC:12515]	K05609	UCHL3, YUH1
ENSP00000441434.1	nicotinamide N-methyltransferase [Source:HGNC Symbol;Acc:HGNC:7861]	K00541	NNMT
ENSP00000259512.3	derlin 1 [Source:HGNC Symbol;Acc:HGNC:28454]	K11519	DERL1
ENSP00000468051.1	phenylalanyl-tRNA synthetase subunit alpha [Source:HGNC Symbol;Acc:HGNC:3592]	K01889	FARSA, pheS
ENSP00000261313.2	phosphatidylethanolamine binding protein 1 [Source:HGNC Symbol;Acc:HGNC:8630]		
ENSP00000313327.6	heterogeneous nuclear ribonucleoprotein D [Source:HGNC Symbol;Acc:HGNC:5036]		
ENSP00000427985.2	dihydropyrimidinase like 2 [Source:HGNC Symbol;Acc:HGNC:3014]	K07528	DPYSL2, CRMP2
ENSP00000444711.1	ring finger protein 24 [Source:HGNC Symbol;Acc:HGNC:13779]		
ENSP00000348395.6	aminopeptidase like 1 [Source:HGNC Symbol;Acc:HGNC:16244]	K09611	NPEPL1
ENSP00000358814.5	adenosylhomocysteinase like 1 [Source:HGNC Symbol;Acc:HGNC:344]	K01251	E3.3.1.1, ahcY
ENSP00000329471.2	KDEL endoplasmic reticulum protein retention receptor 1 [Source:HGNC Symbol;Acc:HGNC:63	K10949	KDELR

ENSP00000495440.1	tropomyosin 2 [Source:HGNC Symbol;Acc:HGNC:12011]		
ENSP00000303908.4	twinfilin actin binding protein 2 [Source:HGNC Symbol;Acc:HGNC:9621]	K08870	TWF
ENSP00000362413.4	phosphoglycerate kinase 1 [Source:HGNC Symbol;Acc:HGNC:8896]	K00927	PGK, pgk
ENSP00000422473.1	transmembrane protein 33 [Source:HGNC Symbol;Acc:HGNC:25541]	K20724	TMEM33
ENSP00000262160.6	SMAD family member 2 [Source:HGNC Symbol;Acc:HGNC:6768]	K04500	SMAD2_3
ENSP00000299138.7	VPS35 retromer complex component [Source:HGNC Symbol;Acc:HGNC:13487]	K18468	VPS35
ENSP00000386350.1	Obg like ATPase 1 [Source:HGNC Symbol;Acc:HGNC:28833]	K19788	OLA1
ENSP00000329757.4	ATPase H+ transporting V0 subunit c [Source:HGNC Symbol;Acc:HGNC:855]	K02155	ATPeV0C, ATP6L
ENSP00000361883.3	cyclase associated actin cytoskeleton regulatory protein 1 [Source:HGNC Symbol;Acc:HGNC:20	K17261	CAP1_2, SRV2
ENSP00000332887.6	ubiquinol-cytochrome c reductase, complex III subunit X [Source:HGNC Symbol;Acc:HGNC:30	K00419	QCR9, UCRC
ENSP00000260956.4	small RNA binding exonuclease protection factor La [Source:HGNC Symbol;Acc:HGNC:11316]	K11090	LA, SSB
ENSP00000360170.3	angiopoietin like 3 [Source:HGNC Symbol;Acc:HGNC:491]		
ENSP00000265087.4	stanniocalcin 2 [Source:HGNC Symbol;Acc:HGNC:11374]		
ENSP00000225576.3	trans-golgi network vesicle protein 23 homolog C [Source:HGNC Symbol;Acc:HGNC:30453]		
ENSP00000261226.4	transmembrane and coiled-coil domain family 3 [Source:HGNC Symbol;Acc:HGNC:29199]		
ENSP00000292246.3	anoctamin 10 [Source:HGNC Symbol;Acc:HGNC:25519]		
ENSP00000357384.1	dolichyl-phosphate mannosyltransferase subunit 3, regulatory [Source:HGNC Symbol;Acc:HGN	K09659	DPM3
ENSP00000432830.1	adipogenesis associated Mth938 domain containing [Source:HGNC Symbol;Acc:HGNC:30205]		
ENSP00000295802.4	retinol saturase [Source:HGNC Symbol;Acc:HGNC:25991]	K09516	RETSAT
ENSP00000356256.4	translocase of inner mitochondrial membrane 17A [Source:HGNC Symbol;Acc:HGNC:17315]	K17795	TIM17
ENSP00000441875.1	prohibitin 2 [Source:HGNC Symbol;Acc:HGNC:30306]	K17081	PHB2
ENSP00000461922.1	transmembrane protein 104 [Source:HGNC Symbol;Acc:HGNC:25984]		
ENSP00000360640.4	dolichyl-phosphate mannosyltransferase subunit 1, catalytic [Source:HGNC Symbol;Acc:HGNC:3005]		
ENSP00000389872.2	oxidoreductase NAD binding domain containing 1 [Source:HGNC Symbol;Acc:HGNC:25128]		
ENSP00000223061.5	procollagen C-endopeptidase enhancer [Source:HGNC Symbol;Acc:HGNC:8738]		
ENSP00000247026.5	nuclear speckle splicing regulatory protein 1 [Source:HGNC Symbol;Acc:HGNC:25305]	K13206	CCDC55
ENSP00000484616.1	cell proliferation regulating inhibitor of protein phosphatase 2A [Source:HGNC Symbol;Acc:HGNC:29302]		
ENSP00000361262.1	peroxiredoxin like 2A [Source:HGNC Symbol;Acc:HGNC:28651]		
ENSP00000362160.5	solute carrier family 25 member 25 [Source:HGNC Symbol;Acc:HGNC:20663]		
ENSP00000406870.1	chromosome 6 open reading frame 47 [Source:HGNC Symbol;Acc:HGNC:19076]		
ENSP00000325548.4	carnosine dipeptidase 2 [Source:HGNC Symbol;Acc:HGNC:24437]	K08660	CNDP2
ENSP00000233078.3	DAZ associated protein 1 [Source:HGNC Symbol;Acc:HGNC:2683]	K14411	MSI
ENSP00000223208.4	centrosomal protein 41 [Source:HGNC Symbol;Acc:HGNC:12370]	K16455	CEP41, TSGA14
ENSP00000295927.3	pentraxin 3 [Source:HGNC Symbol;Acc:HGNC:9692]		
ENSP00000354040.4	GTP binding protein 4 [Source:HGNC Symbol;Acc:HGNC:21535]	K06943	NOG1
ENSP00000265339.2	ubiquitin conjugating enzyme E2 B [Source:HGNC Symbol;Acc:HGNC:12473]	K10574	UBE2B, RAD6B
ENSP00000357205.3	cellular retinoic acid binding protein 2 [Source:HGNC Symbol;Acc:HGNC:2339]	K17289	CRABP2
ENSP00000225698.4	complement C1q binding protein [Source:HGNC Symbol;Acc:HGNC:1243]	K15414	C1QBP

ENSP00000435820.1	solute carrier family 52 member 2 [Source:HGNC Symbol;Acc:HGNC:30224]		
ENSP00000385594.1	CAP-Gly domain containing linker protein family member 4 [Source:HGNC Symbol;Acc:HGNC:	K10423	CLIP3_4
ENSP00000256255.6	store-operated calcium entry associated regulatory factor [Source:HGNC Symbol;Acc:HGNC:28789]		
ENSP00000402316.2	transporter 1, ATP binding cassette subfamily B member [Source:HGNC Symbol;Acc:HGNC:43]	K05653	ABCB2, TAP1
ENSP00000421315.2	novel protein, MINDY4 and AQP1 readthrough		
ENSP00000362041.4	DnaJ heat shock protein family (Hsp40) member C9 [Source:HGNC Symbol;Acc:HGNC:19123]	K09529	DNAJC9
ENSP00000380378.4	platelet activating factor acetylhydrolase 1b regulatory subunit 1 [Source:HGNC Symbol;Acc:H(	K16794	PAFAH1B1, LIS1
ENSP00000297785.3	aldehyde dehydrogenase 1 family member A1 [Source:HGNC Symbol;Acc:HGNC:402]	K07249	ALDH1A
ENSP00000486049.1	surfeit 4 [Source:HGNC Symbol;Acc:HGNC:11476]	K20369	ERV29, SURF4
ENSP00000407860.2	cytochrome P450 family 20 subfamily A member 1 [Source:HGNC Symbol;Acc:HGNC:20576]	K07435	CYP20A
ENSP00000380037.3	fat storage inducing transmembrane protein 2 [Source:HGNC Symbol;Acc:HGNC:16135]		
ENSP00000497280.1	acylglycerol kinase [Source:HGNC Symbol;Acc:HGNC:21869]	K09881	AGK
ENSP00000479794.1	histone cluster 1 H4 family member k [Source:HGNC Symbol;Acc:HGNC:4784]	K11254	H4
ENSP00000310263.3	TNF receptor superfamily member 10d [Source:HGNC Symbol;Acc:HGNC:11907]	K04722	TNFRSF10, TRAILR, CD
ENSP00000185150.4	endoplasmic reticulum lectin 1 [Source:HGNC Symbol;Acc:HGNC:25222]	K14008	ERLEC1, XTP3B
ENSP00000246024.2	thioredoxin related transmembrane protein 4 [Source:HGNC Symbol;Acc:HGNC:25237]		
ENSP00000463042.1	serine and arginine rich splicing factor 1 [Source:HGNC Symbol;Acc:HGNC:10780]	K12890	SFRS1, ASF, SF2
ENSP00000409456.1	heterogeneous nuclear ribonucleoprotein K [Source:HGNC Symbol;Acc:HGNC:5044]		
ENSP00000264079.5	mucolipin 1 [Source:HGNC Symbol;Acc:HGNC:13356]	K04992	MCOLN1
ENSP00000368438.5	proliferating cell nuclear antigen [Source:HGNC Symbol;Acc:HGNC:8729]	K04802	PCNA
ENSP00000264065.6	DnaJ heat shock protein family (Hsp40) member C10 [Source:HGNC Symbol;Acc:HGNC:24637]	K09530	DNAJC10
ENSP00000335200.6	protein phosphatase 2 phosphatase activator [Source:HGNC Symbol;Acc:HGNC:9308]		
ENSP00000260985.2	isocitrate dehydrogenase (NADP(+)) 1, cytosolic [Source:HGNC Symbol;Acc:HGNC:5382]	K00031	IDH1, IDH2, icd
ENSP00000355896.4	transforming growth factor beta 2 [Source:HGNC Symbol;Acc:HGNC:11768]		
ENSP00000424063.1	calnexin [Source:HGNC Symbol;Acc:HGNC:1473]	K08054	CANX
ENSP00000350512.4	COP9 signalosome subunit 5 [Source:HGNC Symbol;Acc:HGNC:2240]	K09613	COPS5, CSN5
ENSP00000355493.3	adenylosuccinate synthase [Source:HGNC Symbol;Acc:HGNC:292]	K01939	purA, ADSS
ENSP00000323678.3	zinc binding alcohol dehydrogenase domain containing 2 [Source:HGNC Symbol;Acc:HGNC:2(	K07119	K07119
ENSP00000368646.4	peroxiredoxin 4 [Source:HGNC Symbol;Acc:HGNC:17169]	K03386	PRDX2_4, ahpC
ENSP00000306003.4	ATP synthase membrane subunit e [Source:HGNC Symbol;Acc:HGNC:846]	K02129	ATPeF0E, ATP5I
ENSP00000229379.2	cytochrome c oxidase subunit 6A1 [Source:HGNC Symbol;Acc:HGNC:2277]	K02266	COX6A
ENSP00000420200.1	ADP-ribosylarginine hydrolase [Source:HGNC Symbol;Acc:HGNC:269]	K01245	ADPRH
ENSP00000348933.4	coiled-coil domain containing 25 [Source:HGNC Symbol;Acc:HGNC:25591]		
ENSP00000481022.1	secretory carrier membrane protein 1 [Source:HGNC Symbol;Acc:HGNC:10563]	K19995	SCAMP
ENSP00000228506.3	malectin [Source:HGNC Symbol;Acc:HGNC:28973]		
ENSP00000445175.1	lactate dehydrogenase A [Source:HGNC Symbol;Acc:HGNC:6535]	K00016	LDH, ldh
ENSP00000324527.5	myosin ID [Source:HGNC Symbol;Acc:HGNC:7598]	K10356	MYO1
ENSP00000341364.5	transmembrane protein 230 [Source:HGNC Symbol;Acc:HGNC:15876]		

ENSP00000371393.4	phosphoglucomutase 2 [Source:HGNC Symbol;Acc:HGNC:8906]	K15779	PGM2
ENSP00000499501.1	histone cluster 2 H3 pseudogene 2 [Source:HGNC Symbol;Acc:HGNC:32060]		
ENSP00000339328.3	plasminogen activator, urokinase receptor [Source:HGNC Symbol;Acc:HGNC:9053]	K03985	PLAUR, CD87
ENSP00000300249.4	microtubule associated protein RP/EB family member 2 [Source:HGNC Symbol;Acc:HGNC:6891	K10436	MAPRE
ENSP00000367893.3	selenophosphate synthetase 1 [Source:HGNC Symbol;Acc:HGNC:19685]	K01008	selD, SEPHS
ENSP00000370718.3	acetylserotonin O-methyltransferase like [Source:HGNC Symbol;Acc:HGNC:751]		
ENSP00000473589.1	S100 calcium binding protein A6 [Source:HGNC Symbol;Acc:HGNC:10496]		
ENSP00000381504.2	phosphoribosyl pyrophosphate synthetase 2 [Source:HGNC Symbol;Acc:HGNC:9465]	K00948	PRPS, prsA
ENSP00000336702.7	eukaryotic translation initiation factor 5A [Source:HGNC Symbol;Acc:HGNC:3300]	K03263	EIF5A
ENSP00000366098.4	Yip1 interacting factor homolog A, membrane trafficking protein [Source:HGNC Symbol;Acc:H	K20362	YIF1
ENSP00000301761.2	succinate dehydrogenase complex assembly factor 2 [Source:HGNC Symbol;Acc:HGNC:26034]	K18168	SDHAF2, SDH5
ENSP00000351035.4	hydroxysteroid 17-beta dehydrogenase 11 [Source:HGNC Symbol;Acc:HGNC:22960]		
ENSP00000216218.3	ST13 Hsp70 interacting protein [Source:HGNC Symbol;Acc:HGNC:11343]	K09560	ST13
ENSP00000269919.3	pyroglutamyl-peptidase I [Source:HGNC Symbol;Acc:HGNC:13568]	K01304	pcp
ENSP00000468312.1	EMAP like 2 [Source:HGNC Symbol;Acc:HGNC:18035]	K18595	EML1_2
ENSP00000273398.3	ATPase H+ transporting V1 subunit A [Source:HGNC Symbol;Acc:HGNC:851]	K02145	ATPeV1A, ATP6A
ENSP00000378040.3	BCL2 associated agonist of cell death [Source:HGNC Symbol;Acc:HGNC:936]	K02158	BAD
ENSP00000377969.3	general transcription factor IIF subunit 1 [Source:HGNC Symbol;Acc:HGNC:4652]	K03138	TFIIF1, GTF2F1, TFG1
ENSP00000483688.1	proteasome subunit beta 3 [Source:HGNC Symbol;Acc:HGNC:9540]	K02735	PSMB3
ENSP00000462521.1	thioredoxin interacting protein [Source:HGNC Symbol;Acc:HGNC:16952]	K20910	TXNIP
ENSP00000349456.2	chloride channel CLIC like 1 [Source:HGNC Symbol;Acc:HGNC:29675]		
ENSP00000336866.4	RB binding protein 9, serine hydrolase [Source:HGNC Symbol;Acc:HGNC:9892]	K07002	K07002
ENSP00000363979.3	dynein light chain roadblock-type 1 [Source:HGNC Symbol;Acc:HGNC:15468]	K10419	DYNLRB, DNCL2
ENSP00000299596.4	transmembrane protein 41B [Source:HGNC Symbol;Acc:HGNC:28948]		
ENSP00000486167.1	unc-79 homolog, NALCN channel complex subunit [Source:HGNC Symbol;Acc:HGNC:19966]		
ENSP00000345344.5	cathepsin L [Source:HGNC Symbol;Acc:HGNC:2537]	K01365	CTSL
ENSP00000356263.3	acidic residue methyltransferase 1 [Source:HGNC Symbol;Acc:HGNC:17872]		
ENSP00000336693.2	abhydrolase domain containing 12B [Source:HGNC Symbol;Acc:HGNC:19837]	K13705	ABHD12B
ENSP00000465759.2	RNA polymerase mitochondrial [Source:HGNC Symbol;Acc:HGNC:9200]	K10908	POLRMT, RPO41
ENSP00000291565.4	pyridoxal kinase [Source:HGNC Symbol;Acc:HGNC:8819]	K00868	pdxK, pdxY
ENSP00000496485.1	ribosomal protein S29 [Source:HGNC Symbol;Acc:HGNC:10419]	K02980	RP-S29e, RPS29
ENSP00000317331.3	signal sequence receptor subunit 4 [Source:HGNC Symbol;Acc:HGNC:11326]	K04571	SSR4
ENSP00000381607.3	glutathione S-transferase pi 1 [Source:HGNC Symbol;Acc:HGNC:4638]	K00799	GST, gst
ENSP00000237281.3	F-box protein 30 [Source:HGNC Symbol;Acc:HGNC:15600]	K10307	FBXO30
ENSP00000306220.2	membrane magnesium transporter 1 [Source:HGNC Symbol;Acc:HGNC:28100]		
ENSP00000459719.1	capicua transcriptional repressor [Source:HGNC Symbol;Acc:HGNC:14214]	K20225	CIC
ENSP00000496757.1	ribosomal protein S7 [Source:HGNC Symbol;Acc:HGNC:10440]	K02993	RP-S7e, RPS7
ENSP00000298159.6	cofilin 2 [Source:HGNC Symbol;Acc:HGNC:1875]	K05765	CFL

ENSP00000264167.4	alkylglycerone phosphate synthase [Source:HGNC Symbol;Acc:HGNC:327]	K00803	AGPS, agpS
ENSP00000380734.2	family with sequence similarity 98 member B [Source:HGNC Symbol;Acc:HGNC:26773]	K15434	FAM98B
ENSP00000405405.2	pleckstrin homology like domain family B member 2 [Source:HGNC Symbol;Acc:HGNC:29573]		
ENSP00000259526.3	cellular communication network factor 3 [Source:HGNC Symbol;Acc:HGNC:7885]		
ENSP00000417764.1	ALG2 alpha-1,3/1,6-mannosyltransferase [Source:HGNC Symbol;Acc:HGNC:23159]	K03843	ALG2
ENSP00000266671.5	pleckstrin homology like domain family A member 1 [Source:HGNC Symbol;Acc:HGNC:8933]		
ENSP00000483921.1	single stranded DNA binding protein 2 [Source:HGNC Symbol;Acc:HGNC:15831]		
ENSP00000323635.3	family with sequence similarity 210 member A [Source:HGNC Symbol;Acc:HGNC:28346]		
ENSP00000283206.4	transmembrane protein 87B [Source:HGNC Symbol;Acc:HGNC:25913]		
ENSP00000235835.3	aldo-keto reductase family 7 member A2 [Source:HGNC Symbol;Acc:HGNC:389]	K15303	AKR7
ENSP00000356820.4	mitochondrial pyruvate carrier 2 [Source:HGNC Symbol;Acc:HGNC:24515]		
ENSP00000274787.2	HIG1 hypoxia inducible domain family member 2A [Source:HGNC Symbol;Acc:HGNC:28311]		
ENSP00000265395.2	3-hydroxyisobutyrate dehydrogenase [Source:HGNC Symbol;Acc:HGNC:4907]	K00020	mmsB, HIBADH
ENSP00000389351.1	ribosomal protein SA [Source:HGNC Symbol;Acc:HGNC:6502]	K02998	RP-SAe, RPSA
ENSP00000405424.2	oxidation resistance 1 [Source:HGNC Symbol;Acc:HGNC:15822]		
ENSP00000328729.6	selenoprotein F [Source:HGNC Symbol;Acc:HGNC:17705]		
ENSP00000312143.9	tensin 3 [Source:HGNC Symbol;Acc:HGNC:21616]	K18080	TNS
ENSP00000288699.6	dihydropyrimidinase like 5 [Source:HGNC Symbol;Acc:HGNC:20637]	K07529	DPYSL5, CRAM
ENSP00000364839.4	ASXL transcriptional regulator 1 [Source:HGNC Symbol;Acc:HGNC:18318]	K11471	ASXL
ENSP00000280154.7	programmed cell death 4 [Source:HGNC Symbol;Acc:HGNC:8763]	K16865	PDCD4
ENSP00000296543.4	N(alpha)-acetyltransferase 15, NatA auxiliary subunit [Source:HGNC Symbol;Acc:HGNC:30782]	K20792	NAA15_16
ENSP00000299705.5	transmembrane p24 trafficking protein 3 [Source:HGNC Symbol;Acc:HGNC:28889]	K20350	TMED3
ENSP00000298510.2	peroxiredoxin 3 [Source:HGNC Symbol;Acc:HGNC:9354]	K20011	PRDX3
ENSP00000329715.4	developmentally regulated GTP binding protein 1 [Source:HGNC Symbol;Acc:HGNC:3029]		
ENSP00000498466.1	Fas cell surface death receptor [Source:HGNC Symbol;Acc:HGNC:11920]	K04390	TNFRSF6, FAS, CD95
ENSP00000364469.1	apolipoprotein A1 [Source:HGNC Symbol;Acc:HGNC:600]	K08757	APOA1
ENSP00000410715.2	secreted frizzled related protein 4 [Source:HGNC Symbol;Acc:HGNC:10778]	K02185	SFRP4
ENSP00000400482.2	CKLF like MARVEL transmembrane domain containing 3 [Source:HGNC Symbol;Acc:HGNC:19174]		
ENSP00000331209.4	chromosome 11 open reading frame 54 [Source:HGNC Symbol;Acc:HGNC:30204]		
ENSP00000457243.1	nucleolar protein 3 [Source:HGNC Symbol;Acc:HGNC:7869]		
ENSP00000477532.1	UBX domain protein 8 [Source:HGNC Symbol;Acc:HGNC:30307]		
ENSP00000428702.1	cytochrome c oxidase subunit 6C [Source:HGNC Symbol;Acc:HGNC:2285]	K02268	COX6C
ENSP00000481360.1	collagen type V alpha 1 chain [Source:HGNC Symbol;Acc:HGNC:2209]	K19721	COL5AS
ENSP00000351314.4	proteasome subunit beta 10 [Source:HGNC Symbol;Acc:HGNC:9538]	K02733	PSMB10, MECL1
ENSP00000319192.5	serine/threonine kinase 17a [Source:HGNC Symbol;Acc:HGNC:11395]	K08804	STK17
ENSP00000357360.3	metaxin 1 [Source:HGNC Symbol;Acc:HGNC:7504]	K17776	MTX
ENSP00000237530.6	ribophorin II [Source:HGNC Symbol;Acc:HGNC:10382]	K12667	SWP1, RPN2
ENSP00000309432.4	reticulophagy regulator family member 3 [Source:HGNC Symbol;Acc:HGNC:27258]		

ENSP00000388806.3	eukaryotic translation initiation factor 4B [Source:HGNC Symbol;Acc:HGNC:3285]	K03258	EIF4B
ENSP00000325919.6	proteasome assembly chaperone 2 [Source:HGNC Symbol;Acc:HGNC:24929]	K11876	PSMG2, PAC2
ENSP00000301012.3	mevalonate diphosphate decarboxylase [Source:HGNC Symbol;Acc:HGNC:7529]	K01597	MVD, mvaD
ENSP00000500517.1	aldehyde dehydrogenase 3 family member A2 [Source:HGNC Symbol;Acc:HGNC:403]	K00128	ALDH
ENSP00000448617.1	heterogeneous nuclear ribonucleoprotein A1 [Source:HGNC Symbol;Acc:HGNC:5031]		
ENSP00000385060.3	apolipoprotein B mRNA editing enzyme catalytic subunit 3B [Source:HGNC Symbol;Acc:HGNC	K18750	APOBEC3
ENSP00000461521.1	secretory carrier membrane protein 3 [Source:HGNC Symbol;Acc:HGNC:10565]	K19995	SCAMP
ENSP00000367880.3	phosphatidylinositol glycan anchor biosynthesis class O [Source:HGNC Symbol;Acc:HGNC:232	K05288	PIGO
ENSP00000306817.3	ribokinase [Source:HGNC Symbol;Acc:HGNC:30325]	K00852	rbsK, RBKS
ENSP00000369757.4	ribosomal protein S6 [Source:HGNC Symbol;Acc:HGNC:10429]	K02991	RP-S6e, RPS6
ENSP00000455282.1	FUS RNA binding protein [Source:HGNC Symbol;Acc:HGNC:4010]	K13098	TLS, FUS
ENSP00000431696.1	cofilin 1 [Source:HGNC Symbol;Acc:HGNC:1874]	K05765	CFL
ENSP00000454786.2	novel protein	K02602;	CPEB, ORB;RP-S17e, R
ENSP00000385609.1	pleckstrin homology domain containing B2 [Source:HGNC Symbol;Acc:HGNC:19236]		
ENSP00000344458.4	protein Z, vitamin K dependent plasma glycoprotein [Source:HGNC Symbol;Acc:HGNC:9460]		
ENSP00000216832.4	pinin, desmosome associated protein [Source:HGNC Symbol;Acc:HGNC:9162]	K13114	PNN
ENSP00000484398.1	smoothelin [Source:HGNC Symbol;Acc:HGNC:11126]		
ENSP00000499883.1	ATP synthase membrane subunit c locus 2 [Source:HGNC Symbol;Acc:HGNC:842]	K02128	ATPeF0C, ATP5G, ATP
ENSP00000216294.4	small nuclear RNA activating complex polypeptide 1 [Source:HGNC Symbol;Acc:HGNC:11134]	K15208	SNAPC1
ENSP00000485698.1	mitochondrial transcription rescue factor 1 [Source:HGNC Symbol;Acc:HGNC:17971]		
ENSP00000326699.5	calmegin [Source:HGNC Symbol;Acc:HGNC:2060]	K09551	CLGN
ENSP00000352401.6	ribulose-5-phosphate-3-epimerase [Source:HGNC Symbol;Acc:HGNC:10293]	K01783	rpe, RPE
ENSP00000342512.2	chromosome 3 open reading frame 33 [Source:HGNC Symbol;Acc:HGNC:26434]		
ENSP00000247225.6	sphingosine-1-phosphate phosphatase 1 [Source:HGNC Symbol;Acc:HGNC:17720]	K04716	SGPP1
ENSP00000448762.1	poly(rC) binding protein 2 [Source:HGNC Symbol;Acc:HGNC:8648]	K13162	PCBP2
ENSP00000260818.6	DnaJ heat shock protein family (Hsp40) member C13 [Source:HGNC Symbol;Acc:HGNC:30343]	K09533	DNAJC13
ENSP00000206451.6	proteasome activator subunit 1 [Source:HGNC Symbol;Acc:HGNC:9568]	K06696	PSME1
ENSP00000366568.4	ubiquitin like modifier activating enzyme 1 [Source:HGNC Symbol;Acc:HGNC:12469]	K03178	UBE1, UBA1
ENSP00000311572.5	prostaglandin reductase 1 [Source:HGNC Symbol;Acc:HGNC:18429]	K13948	PTGR1, LTB4DH
ENSP00000293872.8	LUC7 like [Source:HGNC Symbol;Acc:HGNC:6723]		
ENSP00000273920.3	enolase-phosphatase 1 [Source:HGNC Symbol;Acc:HGNC:24599]	K09880	mtnC, ENOPH1
ENSP00000258418.5	calcium binding protein 39 [Source:HGNC Symbol;Acc:HGNC:20292]	K08272	CAB39, MO25
ENSP00000328287.6	ring finger protein 123 [Source:HGNC Symbol;Acc:HGNC:21148]	K12169	KPC1, RNF123
ENSP00000496813.1	guided entry of tail-anchored proteins factor 1 [Source:HGNC Symbol;Acc:HGNC:12790]		
ENSP00000477352.1	short coiled-coil protein [Source:HGNC Symbol;Acc:HGNC:20335]	K20316	SCOC
ENSP00000310169.4	H2A histone family member Y [Source:HGNC Symbol;Acc:HGNC:4740]		
ENSP00000378356.3	kinesin family member 20A [Source:HGNC Symbol;Acc:HGNC:9787]	K10402	KIF20
ENSP00000307423.2	mannosyl (alpha-1,6-)-glycoprotein beta-1,2-N-acetylglucosaminyltransferase [Source:HGNC	K00736	MGAT2

ENSP00000311449.5	RAB6A, member RAS oncogene family [Source:HGNC Symbol;Acc:HGNC:9786]	K07893	RAB6A
ENSP00000465075.1	SPC24 component of NDC80 kinetochore complex [Source:HGNC Symbol;Acc:HGNC:26913]	K11549	SPBC24, SPC24
ENSP00000422078.1	SUB1 regulator of transcription [Source:HGNC Symbol;Acc:HGNC:19985]		
ENSP00000356982.5	ubiquitin-fold modifier conjugating enzyme 1 [Source:HGNC Symbol;Acc:HGNC:26941]	K12165	UFC1
ENSP00000359098.2	cytochrome c oxidase subunit 7A2 [Source:HGNC Symbol;Acc:HGNC:2288]	K02270	COX7A
ENSP00000383152.1	DExD-box helicase 39B [Source:HGNC Symbol;Acc:HGNC:13917]	K12812	UAP56, BAT1, SUB2
ENSP00000479383.1	meiosis regulator and mRNA stability factor 1 [Source:HGNC Symbol;Acc:HGNC:29562]	K17573	LKAP, MARF1
ENSP00000499089.1	prolyl endopeptidase [Source:HGNC Symbol;Acc:HGNC:9358]	K01322	PREP
ENSP00000310309.7	actin binding LIM protein family member 3 [Source:HGNC Symbol;Acc:HGNC:29132]	K07520	ABLIM
ENSP00000475979.1	COP9 signalosome subunit 9 [Source:HGNC Symbol;Acc:HGNC:21314]		
ENSP00000349016.4	peroxisomal biogenesis factor 14 [Source:HGNC Symbol;Acc:HGNC:8856]	K13343	PEX14
ENSP00000370115.5	serpin family B member 1 [Source:HGNC Symbol;Acc:HGNC:3311]	K13963	SERPINB
ENSP00000268150.8	milk fat globule-EGF factor 8 protein [Source:HGNC Symbol;Acc:HGNC:7036]	K17253	MFGE8
ENSP00000262545.2	proprotein convertase subtilisin/kexin type 2 [Source:HGNC Symbol;Acc:HGNC:8744]	K01360	PCSK2
ENSP00000421130.1	calpastatin [Source:HGNC Symbol;Acc:HGNC:1515]	K04281	CAST
ENSP00000428426.2	transcription elongation factor A1 [Source:HGNC Symbol;Acc:HGNC:11612]	K03145	TFIIS
ENSP00000365522.3	chromosome 9 open reading frame 64 [Source:HGNC Symbol;Acc:HGNC:28144]		
ENSP00000314649.3	aldehyde dehydrogenase 5 family member A1 [Source:HGNC Symbol;Acc:HGNC:408]		
ENSP00000357950.5	ceramide synthase 2 [Source:HGNC Symbol;Acc:HGNC:14076]	K04710	CERS
ENSP00000347206.4	glycosylphosphatidylinositol anchor attachment 1 [Source:HGNC Symbol;Acc:HGNC:4446]	K05289	GAA1
ENSP00000278353.4	hydroxysteroid 17-beta dehydrogenase 12 [Source:HGNC Symbol;Acc:HGNC:18646]	K10251	HSD17B12, KAR, IFA38
ENSP00000499140.1	carboxylesterase 2 [Source:HGNC Symbol;Acc:HGNC:1864]		
ENSP00000307292.3	caveolae associated protein 3 [Source:HGNC Symbol;Acc:HGNC:9400]		
ENSP00000379513.3	microsomal glutathione S-transferase 1 [Source:HGNC Symbol;Acc:HGNC:7061]	K00799	GST, gst
ENSP00000363973.3	alkaline phosphatase, biomineralization associated [Source:HGNC Symbol;Acc:HGNC:438]	K01077	E3.1.3.1, phoA, phoB
ENSP00000438588.2	methylenetetrahydrofolate dehydrogenase, cyclohydrolase and formyltetrahydrofolate synthet	K00288	MTHFD
ENSP00000271850.7	tropomyosin 3 [Source:HGNC Symbol;Acc:HGNC:12012]		
ENSP00000500542.1	SAC1 like phosphatidylinositide phosphatase [Source:HGNC Symbol;Acc:HGNC:17059]		
ENSP00000387059.3	Jupiter microtubule associated homolog 1 [Source:HGNC Symbol;Acc:HGNC:14569]		
ENSP00000434657.1	serpin family H member 1 [Source:HGNC Symbol;Acc:HGNC:1546]	K09501	SERPINH1, HSP47
ENSP00000445920.1	metabolism of cobalamin associated B [Source:HGNC Symbol;Acc:HGNC:19331]	K00798	MMAB, pduO
ENSP00000418529.1	NSFL1 cofactor [Source:HGNC Symbol;Acc:HGNC:15912]	K14012	SHP1, UBX1, NSFL1C
ENSP00000349878.5	heparin binding growth factor [Source:HGNC Symbol;Acc:HGNC:4856]	K16641	HDGF
ENSP00000381102.4	phosphatidylinositol-4,5-bisphosphate 4-phosphatase 1 [Source:HGNC Symbol;Acc:HGNC:19299]		
ENSP00000300738.5	ribonucleotide reductase catalytic subunit M1 [Source:HGNC Symbol;Acc:HGNC:10451]	K10807	RRM1
ENSP00000468424.1	N-myristoyltransferase 1 [Source:HGNC Symbol;Acc:HGNC:7857]	K00671	E2.3.1.97, NMT
ENSP00000488808.1	strawberry notch homolog 2 [Source:HGNC Symbol;Acc:HGNC:29158]		
ENSP00000337572.4	diacylglycerol kinase eta [Source:HGNC Symbol;Acc:HGNC:2854]	K00901	dgkA, DGK

ENSP00000331258.5	protein O-mannose kinase [Source:HGNC Symbol;Acc:HGNC:26267]	K17547	SGK196
ENSP00000344259.4	ubiquitin conjugating enzyme E2 L3 [Source:HGNC Symbol;Acc:HGNC:12488]	K04552	UBE2L3, UBCH7
ENSP00000265316.3	ATP binding cassette subfamily B member 6 (Langereis blood group) [Source:HGNC Symbol;A	K05661	ABCB6
ENSP00000222390.5	hepatocyte growth factor [Source:HGNC Symbol;Acc:HGNC:4893]	K05460	HGF
ENSP00000278671.5	late endosomal/lysosomal adaptor, MAPK and MTOR activator 1 [Source:HGNC Symbol;Acc:H	K20397	LAMTOR1
ENSP00000409382.2	calcium release activated channel regulator 2A [Source:HGNC Symbol;Acc:HGNC:28657]	K17199	RASEF, RAB45
ENSP00000458954.1	nucleoporin 88 [Source:HGNC Symbol;Acc:HGNC:8067]	K14318	NUP88
ENSP00000369965.3	basic transcription factor 3 [Source:HGNC Symbol;Acc:HGNC:1125]	K01527	EGD1, BTF3
ENSP00000272348.2	small nuclear ribonucleoprotein polypeptide G [Source:HGNC Symbol;Acc:HGNC:11163]	K11099	SNRPG, SMG
ENSP00000366395.3	synoviolin 1 [Source:HGNC Symbol;Acc:HGNC:20738]	K10601	SYVN1, HRD1
ENSP00000295702.4	signal sequence receptor subunit 2 [Source:HGNC Symbol;Acc:HGNC:11324]	K13250	SSR2
ENSP00000254301.9	galectin 3 [Source:HGNC Symbol;Acc:HGNC:6563]	K06831	LGALS3
ENSP00000296795.2	toll like receptor 3 [Source:HGNC Symbol;Acc:HGNC:11849]	K05401	TLR3, CD283
ENSP00000158771.4	derlin 2 [Source:HGNC Symbol;Acc:HGNC:17943]	K13989	DERL2_3
ENSP00000423427.2	DEK proto-oncogene [Source:HGNC Symbol;Acc:HGNC:2768]	K17046	DEK
ENSP00000355486.3	cytochrome c oxidase assembly factor COX20 [Source:HGNC Symbol;Acc:HGNC:26970]		
ENSP00000420608.1	ER membrane protein complex subunit 1 [Source:HGNC Symbol;Acc:HGNC:28957]		
ENSP00000449535.1	acyl-CoA synthetase short chain family member 3 [Source:HGNC Symbol;Acc:HGNC:24723]	K01908	prpE
ENSP00000459368.1	single-pass membrane protein with aspartate rich tail 1 [Source:HGNC Symbol;Acc:HGNC:25055]		
ENSP00000499465.1	adenosine monophosphate deaminase 2 [Source:HGNC Symbol;Acc:HGNC:469]	K01490	AMPD
ENSP00000363506.5	ubiquinol-cytochrome c reductase complex assembly factor 1 [Source:HGNC Symbol;Acc:HGN	K17662	CBP3, UQCC
ENSP00000280612.5	solute carrier family 7 member 11 [Source:HGNC Symbol;Acc:HGNC:11059]	K13869	SLC7A11
ENSP00000239215.7	BCL2 interacting protein 1 [Source:HGNC Symbol;Acc:HGNC:1082]	K08497	SEC20
ENSP00000348056.4	ubiquitin conjugating enzyme E2 I [Source:HGNC Symbol;Acc:HGNC:12485]	K10577	UBE2I, UBC9
ENSP00000357625.5	BCL2 interacting protein 3 [Source:HGNC Symbol;Acc:HGNC:1084]	K15464	BNIP3
ENSP00000500775.1	BRI3 binding protein [Source:HGNC Symbol;Acc:HGNC:14251]		
ENSP00000300107.3	caseinolytic mitochondrial matrix peptidase chaperone subunit [Source:HGNC Symbol;Acc:HGI	K03544	clpX, CLPX
ENSP00000360184.4	transmembrane 9 superfamily member 3 [Source:HGNC Symbol;Acc:HGNC:21529]	K17087	TM9SF3
ENSP00000261868.5	eukaryotic translation initiation factor 3 subunit J [Source:HGNC Symbol;Acc:HGNC:3270]	K03245	EIF3J
ENSP00000361682.4	leucine rich repeat containing 8 VRAC subunit A [Source:HGNC Symbol;Acc:HGNC:19027]		
ENSP00000303222.3	mitochondrial carrier 2 [Source:HGNC Symbol;Acc:HGNC:17587]	K17885	MTCH
ENSP00000222284.4	transmembrane protein 147 [Source:HGNC Symbol;Acc:HGNC:30414]		
ENSP00000255120.5	nuclear autoantigenic sperm protein [Source:HGNC Symbol;Acc:HGNC:7644]	K11291	NASP
ENSP00000371193.4	calcineurin like phosphoesterase domain containing 1 [Source:HGNC Symbol;Acc:HGNC:25632]		
ENSP00000248633.4	peroxisomal biogenesis factor 1 [Source:HGNC Symbol;Acc:HGNC:8850]	K13338	PEX1
ENSP00000209875.4	chromobox 5 [Source:HGNC Symbol;Acc:HGNC:1555]	K11587	CBX5, HP1A
ENSP00000267970.4	tetraspanin 3 [Source:HGNC Symbol;Acc:HGNC:17752]	K17293	TSPAN3
ENSP00000420357.2	RWD domain containing 1 [Source:HGNC Symbol;Acc:HGNC:20993]		

ENSP00000358307.3	NHL repeat containing 2 [Source:HGNC Symbol;Acc:HGNC:24731]		
ENSP00000389160.1	NADH:ubiquinone oxidoreductase subunit A11 [Source:HGNC Symbol;Acc:HGNC:20371]	K03956	NDUFA11
ENSP00000220853.3	ER membrane protein complex subunit 2 [Source:HGNC Symbol;Acc:HGNC:28963]		
ENSP00000373553.5	COP9 signalosome subunit 2 [Source:HGNC Symbol;Acc:HGNC:30747]	K12176	COPS2, CSN2, TRIP15
ENSP00000319664.5	nuclear distribution C, dynein complex regulator [Source:HGNC Symbol;Acc:HGNC:8045]		
ENSP00000397843.2	translation machinery associated 7 homolog [Source:HGNC Symbol;Acc:HGNC:26932]		
ENSP00000244217.5	methylmalonyl-CoA epimerase [Source:HGNC Symbol;Acc:HGNC:16732]	K05606	MCEE, epi
ENSP00000286428.5	VHL binding protein 1 [Source:HGNC Symbol;Acc:HGNC:12662]		
ENSP00000356275.1	cysteine and glycine rich protein 1 [Source:HGNC Symbol;Acc:HGNC:2469]	K09377	CSRP
ENSP00000250784.7	ribosomal protein S4 Y-linked 1 [Source:HGNC Symbol;Acc:HGNC:10425]	K02987	RP-S4e, RPS4
ENSP00000264228.4	steroid 5 alpha-reductase 3 [Source:HGNC Symbol;Acc:HGNC:25812]	K12345	SRD5A3
ENSP00000322175.7	adenylate kinase 4 [Source:HGNC Symbol;Acc:HGNC:363]	K00939	adk, AK
ENSP00000235329.5	mitofusin 2 [Source:HGNC Symbol;Acc:HGNC:16877]	K06030	MFN2
ENSP00000355802.4	epoxide hydrolase 1 [Source:HGNC Symbol;Acc:HGNC:3401]	K01253	EPHX1
ENSP00000385347.2	transmembrane anterior posterior transformation 1 [Source:HGNC Symbol;Acc:HGNC:26887]		
ENSP00000296255.3	ribophorin I [Source:HGNC Symbol;Acc:HGNC:10381]	K12666	OST1, RPN1
ENSP00000337746.7	inositol-3-phosphate synthase 1 [Source:HGNC Symbol;Acc:HGNC:29821]	K01858	INO1, ISYNA1
ENSP00000366237.3	DNA fragmentation factor subunit alpha [Source:HGNC Symbol;Acc:HGNC:2772]	K02310	DFFA, DFF45
ENSP00000475750.1	heme binding protein 2 [Source:HGNC Symbol;Acc:HGNC:15716]		
ENSP00000423673.1	NADH:ubiquinone oxidoreductase subunit A13 [Source:HGNC Symbol;Acc:HGNC:17194]	K11353	NDUFA13
ENSP00000264170.4	kynureninase [Source:HGNC Symbol;Acc:HGNC:6469]	K01556	KYNU, kynU
ENSP00000313050.3	nipsnap homolog 2 [Source:HGNC Symbol;Acc:HGNC:4179]		
ENSP00000299157.4	IKBKB interacting protein [Source:HGNC Symbol;Acc:HGNC:26430]		
ENSP00000264663.5	nicotinamide nucleotide transhydrogenase [Source:HGNC Symbol;Acc:HGNC:7863]	K00323	NNT
ENSP00000217456.2	adipocyte plasma membrane associated protein [Source:HGNC Symbol;Acc:HGNC:13238]		
ENSP00000318557.3	solute carrier family 12 member 4 [Source:HGNC Symbol;Acc:HGNC:10913]	K14427	SLC12A4_5_6, KCC
ENSP00000481545.1	NADH:ubiquinone oxidoreductase subunit A3 [Source:NCBI gene;Acc:4696]	K03947	NDUFA3
ENSP00000321259.3	transaldolase 1 [Source:HGNC Symbol;Acc:HGNC:11559]	K00616	E2.2.1.2, talA, talB
ENSP00000365773.3	phosphoserine aminotransferase 1 [Source:HGNC Symbol;Acc:HGNC:19129]	K00831	serC, PSAT1
ENSP00000337053.4	SEL1L adaptor subunit of ERAD E3 ubiquitin ligase [Source:HGNC Symbol;Acc:HGNC:10717]	K14026	SEL1, SEL1L
ENSP00000363824.3	transmembrane protein 38B [Source:HGNC Symbol;Acc:HGNC:25535]		
ENSP00000428184.1	syndecan binding protein [Source:HGNC Symbol;Acc:HGNC:10662]	K17254	SDCBP
ENSP00000379156.4	myosin phosphatase Rho interacting protein [Source:HGNC Symbol;Acc:HGNC:30321]		
ENSP00000422912.1	coiled-coil-helix-coiled-coil-helix domain containing 6 [Source:HGNC Symbol;Acc:HGNC:281	K17564	CHCHD6
ENSP00000281741.4	thioredoxin domain containing 16 [Source:HGNC Symbol;Acc:HGNC:19965]		
ENSP00000297258.6	fatty acid binding protein 5 [Source:HGNC Symbol;Acc:HGNC:3560]	K08754	FABP5
ENSP00000361888.1	cyclase associated actin cytoskeleton regulatory protein 1 [Source:HGNC Symbol;Acc:HGNC:2	K17261	CAP1_2, SRV2
ENSP00000252603.1	6-phosphogluconolactonase [Source:HGNC Symbol;Acc:HGNC:8903]	K01057	PGLS, pgl, devB

ENSP00000229270.4	triosephosphate isomerase 1 [Source:HGNC Symbol;Acc:HGNC:12009]	K01803	TPI, tpiA
ENSP00000355632.4	polypeptide N-acetylgalactosaminyltransferase 2 [Source:HGNC Symbol;Acc:HGNC:4124]	K00710	GALNT
ENSP00000369677.4	elastin microfibril interfacer 1 [Source:HGNC Symbol;Acc:HGNC:19880]		
ENSP00000362153.3	G protein nucleolar 2 [Source:HGNC Symbol;Acc:HGNC:29925]	K14537	NUG2, GNL2
ENSP00000371811.4	mitochondrial calcium uptake 2 [Source:HGNC Symbol;Acc:HGNC:31830]		
ENSP00000272732.6	secernin 3 [Source:HGNC Symbol;Acc:HGNC:30382]	K14358	SCRN
ENSP00000344322.1	solute carrier family 23 member 2 [Source:HGNC Symbol;Acc:HGNC:10973]	K14611	SLC23A1_2, SVCT1_2
ENSP00000357980.3	HtrA serine peptidase 1 [Source:HGNC Symbol;Acc:HGNC:9476]	K08784	HTRA1, PRSS11
ENSP00000340274.2	RAP2C, member of RAS oncogene family [Source:HGNC Symbol;Acc:HGNC:21165]	K07839	RAP2C
ENSP00000357801.3	S100 calcium binding protein A10 [Source:HGNC Symbol;Acc:HGNC:10487]	K17274	S100A10
ENSP00000447000.1	novel protein	K09534;	SARNP, CIP29, THO1;D
ENSP00000362268.2	apolipoprotein O like [Source:HGNC Symbol;Acc:HGNC:24009]		
ENSP00000257966.8	OS9 endoplasmic reticulum lectin [Source:HGNC Symbol;Acc:HGNC:16994]	K10088	OS9
ENSP00000497376.1	protein S [Source:HGNC Symbol;Acc:HGNC:9456]		
ENSP00000263372.2	potassium two pore domain channel subfamily K member 6 [Source:HGNC Symbol;Acc:HGNC:	K04917	KCNK6
ENSP00000215754.7	macrophage migration inhibitory factor [Source:HGNC Symbol;Acc:HGNC:7097]	K07253	MIF
ENSP00000335636.4	N(alpha)-acetyltransferase 20, NatB catalytic subunit [Source:HGNC Symbol;Acc:HGNC:15908]	K17972	NAA20, NAT3
ENSP00000304364.5	C1GALT1 specific chaperone 1 [Source:HGNC Symbol;Acc:HGNC:24338]	K09653	C1GALT2, C1GALT1C1
ENSP00000434643.1	ribosomal protein S3 [Source:HGNC Symbol;Acc:HGNC:10420]	K02985	RP-S3e, RPS3
ENSP00000387180.2	peptidyl-tRNA hydrolase 2 [Source:HGNC Symbol;Acc:HGNC:24265]	K04794	PTH2
ENSP00000287600.4	phosphodiesterase 6D [Source:HGNC Symbol;Acc:HGNC:8788]	K13758	PDE6D
ENSP00000358312.3	peroxisomal biogenesis factor 11 beta [Source:HGNC Symbol;Acc:HGNC:8853]	K13352	PEX11B
ENSP00000393896.2	nebulette [Source:HGNC Symbol;Acc:HGNC:16932]		
ENSP00000360761.2	1-acylglycerol-3-phosphate O-acyltransferase 2 [Source:HGNC Symbol;Acc:HGNC:325]	K13509	AGPAT1_2
ENSP00000490841.1	THUMP domain containing 1 [Source:HGNC Symbol;Acc:HGNC:23807]	K06963	TAN1, THUMPD1
ENSP00000358719.3	malic enzyme 1 [Source:HGNC Symbol;Acc:HGNC:6983]	K00029	E1.1.1.40, maeB
ENSP00000488704.1	nicotinate phosphoribosyltransferase [Source:HGNC Symbol;Acc:HGNC:30450]	K00763	pncB, NAPRT1
ENSP00000343657.6	methylcrotonoyl-CoA carboxylase 2 [Source:HGNC Symbol;Acc:HGNC:6937]	K01969	E6.4.1.4B
ENSP00000238875.4	galectin like [Source:HGNC Symbol;Acc:HGNC:25012]		
ENSP00000464050.1	DEAD-box helicase 42 [Source:HGNC Symbol;Acc:HGNC:18676]	K12835	DDX42, SF3B125
ENSP00000413675.2	atlastin GTPase 1 [Source:HGNC Symbol;Acc:HGNC:11231]	K17339	ATL
ENSP00000386799.1	DIS3 like 3'-5' exoribonuclease 2 [Source:HGNC Symbol;Acc:HGNC:28648]	K18758	DIS3L2
ENSP00000326305.4	solute carrier family 25 member 20 [Source:HGNC Symbol;Acc:HGNC:1421]	K15109	SLC25A20_29, CACT, C
ENSP00000345988.7	phospholipase C eta 1 [Source:HGNC Symbol;Acc:HGNC:29185]	K19006	PLCH
ENSP00000486361.1	diazepam binding inhibitor, acyl-CoA binding protein [Source:HGNC Symbol;Acc:HGNC:2690]	K08762	DBI, ACBP
ENSP00000494125.1	tropomyosin 4 [Source:HGNC Symbol;Acc:HGNC:12013]		
ENSP00000424940.2	hydroxysteroid 17-beta dehydrogenase 4 [Source:HGNC Symbol;Acc:HGNC:5213]	K12405	HSD17B4
ENSP00000295119.4	nucleoporin 35 [Source:HGNC Symbol;Acc:HGNC:29797]	K14313	NUP35, NUP53

ENSP00000419370.1	mitochondrial assembly of ribosomal large subunit 1 [Source:HGNC Symbol;Acc:HGNC:21721]		
ENSP00000493232.1	mitochondrial calcium uptake 1 [Source:HGNC Symbol;Acc:HGNC:1530]		
ENSP00000261427.5	ubiquitin conjugating enzyme E2 K [Source:HGNC Symbol;Acc:HGNC:4914]	K04649	HIP2, UBC1
ENSP00000022615.4	voltage dependent anion channel 3 [Source:HGNC Symbol;Acc:HGNC:12674]	K15041	VDAC3
ENSP00000330433.3	TM2 domain containing 3 [Source:HGNC Symbol;Acc:HGNC:24128]		
ENSP00000400839.2	solute carrier family 35 member G2 [Source:HGNC Symbol;Acc:HGNC:28480]		
ENSP00000381736.5	ATP synthase F1 subunit alpha [Source:HGNC Symbol;Acc:HGNC:823]	K02132	ATPeF1A, ATP5A1, ATF
ENSP00000312042.4	high density lipoprotein binding protein [Source:HGNC Symbol;Acc:HGNC:4857]		
ENSP00000261714.6	bleomycin hydrolase [Source:HGNC Symbol;Acc:HGNC:1059]	K01372	BLMH, pepC
ENSP00000356110.4	nuclear casein kinase and cyclin dependent kinase substrate 1 [Source:HGNC Symbol;Acc:HGNC:29923]		
ENSP00000266712.6	transmembrane O-mannosyltransferase targeting cadherins 3 [Source:HGNC Symbol;Acc:HGNC:26899]		
ENSP00000253108.3	eukaryotic translation initiation factor 3 subunit G [Source:HGNC Symbol;Acc:HGNC:3274]	K03248	EIF3G
ENSP00000325425.6	methionine adenosyltransferase 2B [Source:HGNC Symbol;Acc:HGNC:6905]	K00789	metK
ENSP00000376139.4	WD repeat domain 45B [Source:HGNC Symbol;Acc:HGNC:25072]		
ENSP00000302037.4	mitochondrial fission factor [Source:HGNC Symbol;Acc:HGNC:24858]		
ENSP00000363899.4	nipsnap homolog 3A [Source:HGNC Symbol;Acc:HGNC:23619]		
ENSP00000296412.8	alcohol dehydrogenase 5 (class III), chi polypeptide [Source:HGNC Symbol;Acc:HGNC:253]	K00121	frmA, ADH5, adhC
ENSP00000205194.3	N-acetyltransferase 14 (putative) [Source:HGNC Symbol;Acc:HGNC:28918]		
ENSP00000225655.5	profilin 1 [Source:HGNC Symbol;Acc:HGNC:8881]	K05759	PFN
ENSP00000358857.4	emerin [Source:HGNC Symbol;Acc:HGNC:3331]	K12569	EMD
ENSP00000325562.6	mitochondrial contact site and cristae organizing system subunit 10 [Source:HGNC Symbol;Acc	K17784	MINOS1, MOS1
ENSP00000246868.2	SBDS ribosome maturation factor [Source:HGNC Symbol;Acc:HGNC:19440]	K14574	SDO1, SBDS
ENSP00000384169.3	fibulin 2 [Source:HGNC Symbol;Acc:HGNC:3601]		
ENSP00000434797.1	ring finger protein 170 [Source:HGNC Symbol;Acc:HGNC:25358]	K15707	RNF170
ENSP00000215832.6	mitogen-activated protein kinase 1 [Source:HGNC Symbol;Acc:HGNC:6871]	K04371	MAPK1_3
ENSP00000270586.3	proteasome subunit beta 6 [Source:HGNC Symbol;Acc:HGNC:9543]	K02738	PSMB6
ENSP00000433361.1	TMEM9 domain family member B [Source:HGNC Symbol;Acc:HGNC:1168]		
ENSP00000358012.4	endosulfine alpha [Source:HGNC Symbol;Acc:HGNC:3360]		
ENSP00000483345.1	HDGF like 2 [Source:HGNC Symbol;Acc:HGNC:14680]		
ENSP00000268261.4	phosphomannomutase 2 [Source:HGNC Symbol;Acc:HGNC:9115]	K17497	PMM
ENSP00000487567.1	PWWP domain containing 3A, DNA repair factor [Source:HGNC Symbol;Acc:HGNC:29641]		
ENSP00000380793.3	ALG3 alpha-1,3- mannosyltransferase [Source:HGNC Symbol;Acc:HGNC:23056]	K03845	ALG3
ENSP00000357340.4	farnesyl diphosphate synthase [Source:HGNC Symbol;Acc:HGNC:3631]	K00787	FDPS
ENSP00000439606.2	integrin linked kinase [Source:HGNC Symbol;Acc:HGNC:6040]		
ENSP00000398269.2	myeloid associated differentiation marker [Source:HGNC Symbol;Acc:HGNC:7544]		
ENSP00000417190.2	ATP synthase mitochondrial F1 complex assembly factor 2 [Source:HGNC Symbol;Acc:HGNC:1	K07556	ATPeAF2, ATPAF2, ATF
ENSP00000363129.3	small nuclear ribonucleoprotein polypeptide C [Source:HGNC Symbol;Acc:HGNC:11157]	K11095	SNRPC
ENSP00000315931.3	adenosylhomocysteinase like 2 [Source:HGNC Symbol;Acc:HGNC:22204]	K01251	E3.3.1.1, ahcY

ENSP00000475489.1	M-phase phosphoprotein 9 [Source:HGNC Symbol;Acc:HGNC:7215]	K16775	MPHOSPH9, MPP9
ENSP00000364883.4	AU RNA binding methylglutaconyl-CoA hydratase [Source:HGNC Symbol;Acc:HGNC:890]	K05607	AUH
ENSP00000370839.5	sarcoglycan beta [Source:HGNC Symbol;Acc:HGNC:10806]	K12566	SGCB
ENSP00000300408.3	prohibitin [Source:HGNC Symbol;Acc:HGNC:8912]	K17080	PHB1
ENSP00000361512.4	phosphoribosyl pyrophosphate synthetase 1 [Source:HGNC Symbol;Acc:HGNC:9462]	K00948	PRPS, prsA
ENSP00000295640.4	arginyl aminopeptidase [Source:HGNC Symbol;Acc:HGNC:10078]	K01260	RNPEP
ENSP00000457694.1	Jupiter microtubule associated homolog 2 [Source:HGNC Symbol;Acc:HGNC:14137]		
ENSP00000447091.1	solute carrier family 8 member B1 [Source:HGNC Symbol;Acc:HGNC:26175]	K13754	SLC24A6, NCKX6
ENSP00000415862.1	acylaminoacyl-peptide hydrolase [Source:HGNC Symbol;Acc:HGNC:586]	K01303	APEH
ENSP00000251453.2	ribosomal protein S16 [Source:HGNC Symbol;Acc:HGNC:10396]	K02960	RP-S16e, RPS16
ENSP00000344822.3	S100 calcium binding protein A13 [Source:HGNC Symbol;Acc:HGNC:10490]		
ENSP00000410964.2	ER lipid raft associated 1 [Source:HGNC Symbol;Acc:HGNC:16947]		
ENSP00000497423.1	solute carrier family 35 member B1 [Source:HGNC Symbol;Acc:HGNC:20798]	K15275	SLC35B1
ENSP00000454168.1	solute carrier family 12 member 6 [Source:HGNC Symbol;Acc:HGNC:10914]	K14427	SLC12A4_5_6, KCC
ENSP00000366988.3	sorting nexin 5 [Source:HGNC Symbol;Acc:HGNC:14969]	K17920	SNX5_6_32
ENSP00000348886.5	stomatin like 2 [Source:HGNC Symbol;Acc:HGNC:14559]		
ENSP00000499466.1	peptidylprolyl cis/trans isomerase, NIMA-interacting 4 [Source:HGNC Symbol;Acc:HGNC:8992]	K09579	PIN4
ENSP00000365950.3	RNA binding motif protein 3 [Source:HGNC Symbol;Acc:HGNC:9900]	K13186	RBM3
ENSP00000333994.3	hemoglobin subunit beta [Source:HGNC Symbol;Acc:HGNC:4827]	K13823	HBB
ENSP00000463411.1	secernin 2 [Source:HGNC Symbol;Acc:HGNC:30381]	K14358	SCRN
ENSP00000324173.6	heat shock protein family A (Hsp70) member 5 [Source:HGNC Symbol;Acc:HGNC:5238]	K09490	HSPA5, BIP
ENSP00000475402.1	NADH:ubiquinone oxidoreductase subunit A6 [Source:HGNC Symbol;Acc:HGNC:7690]	K03950	NDUFA6
ENSP00000270776.8	phosphogluconate dehydrogenase [Source:HGNC Symbol;Acc:HGNC:8891]	K00033	PGD, gnd, gntZ
ENSP00000303525.3	carbonyl reductase 4 [Source:HGNC Symbol;Acc:HGNC:25891]	K11539	CBR4
ENSP00000300128.4	nuclear envelope integral membrane protein 1 [Source:HGNC Symbol;Acc:HGNC:29001]		
ENSP00000428924.1	LYN proto-oncogene, Src family tyrosine kinase [Source:HGNC Symbol;Acc:HGNC:6735]		
ENSP00000418354.1	NADH:ubiquinone oxidoreductase subunit B5 [Source:HGNC Symbol;Acc:HGNC:7700]		
ENSP00000349541.4	caveolae associated protein 1 [Source:HGNC Symbol;Acc:HGNC:9688]	K19387	PTRF
ENSP00000482868.2	bolA family member 2 [Source:HGNC Symbol;Acc:HGNC:29488]		
ENSP00000437301.1	radixin [Source:HGNC Symbol;Acc:HGNC:9944]	K05762	RDX
ENSP00000430858.2	novel protein, TPD52-MRPS28 readthrough		
ENSP00000318158.5	mitochondrial ribosomal protein S24 [Source:HGNC Symbol;Acc:HGNC:14510]	K17403	MRPS24
ENSP00000293261.2	transmembrane protein 143 [Source:HGNC Symbol;Acc:HGNC:25603]		
ENSP00000497225.1	protein-L-isoaspartate (D-aspartate) O-methyltransferase [Source:HGNC Symbol;Acc:HGNC:8[illegible]	K00573	E2.1.1.77, pcm
ENSP00000300574.2	CRK proto-oncogene, adaptor protein [Source:HGNC Symbol;Acc:HGNC:2362]	K04438	CRK, CRKII
ENSP00000435289.1	platelet activating factor acetylhydrolase 1b catalytic subunit 2 [Source:HGNC Symbol;Acc:HGN[illegible]	K16795	PAFAH1B2_3
ENSP00000335304.4	dihydrolipoamide S-succinyltransferase [Source:HGNC Symbol;Acc:HGNC:2911]	K00658	DLST, sucB
ENSP00000364188.3	dolichyl-diphosphooligosaccharide--protein glycosyltransferase non-catalytic subunit [Source[illegible]	K12670	WBP1

ENSP00000353770.2	ribonucleotide reductase regulatory subunit M2 [Source:HGNC Symbol;Acc:HGNC:10452]	K10808	RRM2
ENSP00000424381.1	lipase maturation factor 2 [Source:HGNC Symbol;Acc:HGNC:25096]		
ENSP00000365817.4	major histocompatibility complex, class I, E [Source:HGNC Symbol;Acc:HGNC:4962]	K06751	MHC1
ENSP00000465404.1	Parkinsonism associated deglycase [Source:HGNC Symbol;Acc:HGNC:16369]		
ENSP00000363041.4	CDGSH iron sulfur domain 1 [Source:HGNC Symbol;Acc:HGNC:30880]		
ENSP00000355046.4	mitochondrially encoded NADH:ubiquinone oxidoreductase core subunit 2 [Source:HGNC Sym	K03879	ND2
ENSP00000405562.2	POM121 transmembrane nucleoporin [Source:HGNC Symbol;Acc:HGNC:19702]	K14316	POM121, NUP121
ENSP00000454360.1	sphingolipid transporter 1 (putative) [Source:HGNC Symbol;Acc:HGNC:30621]		
ENSP00000408730.1	coiled-coil domain containing 124 [Source:HGNC Symbol;Acc:HGNC:25171]		
ENSP00000475924.2	DNA polymerase delta interacting protein 2 [Source:HGNC Symbol;Acc:HGNC:23781]	K17809	POLDIP2
ENSP00000376347.1	growth factor receptor bound protein 2 [Source:HGNC Symbol;Acc:HGNC:4566]	K04364	GRB2
ENSP00000377612.5	extended synaptotagmin 1 [Source:HGNC Symbol;Acc:HGNC:29534]		
ENSP00000361608.4	ATP binding cassette subfamily C member 10 [Source:HGNC Symbol;Acc:HGNC:52]	K05674	ABCC10
ENSP00000219169.4	nuclear transport factor 2 [Source:HGNC Symbol;Acc:HGNC:13722]		
ENSP00000295470.5	heterogeneous nuclear ribonucleoprotein D like [Source:HGNC Symbol;Acc:HGNC:5037]	K13044	HNRNPABD
ENSP00000264187.6	nidogen 1 [Source:HGNC Symbol;Acc:HGNC:7821]	K06826	NID
ENSP00000278422.4	thioredoxin related transmembrane protein 2 [Source:HGNC Symbol;Acc:HGNC:30739]		
ENSP00000379366.2	MOB kinase activator 1B [Source:HGNC Symbol;Acc:HGNC:29801]	K06685	MOB1, Mats
ENSP00000292782.4	defective in cullin neddylation 1 domain containing 1 [Source:HGNC Symbol;Acc:HGNC:18184	K17822	DCUN1D1_2
ENSP00000330389.4	peptidyl-tRNA hydrolase domain containing 1 [Source:HGNC Symbol;Acc:HGNC:33782]		
ENSP00000222005.1	cell division cycle 37 [Source:HGNC Symbol;Acc:HGNC:1735]	K09554	CDC37
ENSP00000472420.1	ER membrane protein complex subunit 10 [Source:HGNC Symbol;Acc:HGNC:27609]		
ENSP00000357643.3	marker of proliferation Ki-67 [Source:HGNC Symbol;Acc:HGNC:7107]	K17582	MKI67
ENSP00000303366.7	lectin, mannose binding 2 [Source:HGNC Symbol;Acc:HGNC:16986]	K10082	LMAN2, VIP36
ENSP00000263368.3	biliverdin reductase B [Source:HGNC Symbol;Acc:HGNC:1063]	K05901	BLVRB
ENSP00000348775.4	acyl-CoA oxidase 3, pristanoyl [Source:HGNC Symbol;Acc:HGNC:121]	K00232	E1.3.3.6, ACOX1, ACOX
ENSP00000304229.5	histidine triad nucleotide binding protein 1 [Source:HGNC Symbol;Acc:HGNC:4912]	K02503	HINT1, hinT, hit
ENSP00000274376.6	RAS p21 protein activator 1 [Source:HGNC Symbol;Acc:HGNC:9871]	K04352	RASA1, RASGAP
ENSP00000349087.2	transmembrane protein 259 [Source:HGNC Symbol;Acc:HGNC:17039]		
ENSP00000380557.3	A-kinase anchoring protein 8 like [Source:HGNC Symbol;Acc:HGNC:29857]	K15978	AKAP8L, HA95
ENSP00000434511.1	selenoprotein H [Source:HGNC Symbol;Acc:HGNC:18251]		
ENSP00000498621.1	ribosomal protein S23 [Source:HGNC Symbol;Acc:HGNC:10410]	K02973	RP-S23e, RPS23
ENSP00000296581.5	LSM6 homolog, U6 small nuclear RNA and mRNA degradation associated [Source:HGNC Symb	K12625	LSM6
ENSP00000299166.4	NADH:ubiquinone oxidoreductase subunit B8 [Source:HGNC Symbol;Acc:HGNC:7703]	K03964	NDUFB8
ENSP00000216962.3	glycogen phosphorylase B [Source:HGNC Symbol;Acc:HGNC:9723]	K00688	PYG, glgP
ENSP00000321845.6	SEC24 homolog C, COPII coat complex component [Source:HGNC Symbol;Acc:HGNC:10705]	K14007	SEC24
ENSP00000228740.2	leukotriene A4 hydrolase [Source:HGNC Symbol;Acc:HGNC:6710]	K01254	LTA4H
ENSP00000362463.3	glyoxalase I [Source:HGNC Symbol;Acc:HGNC:4323]	K01759	GLO1, gloA

ENSP00000308541.5	coagulation factor II, thrombin [Source:HGNC Symbol;Acc:HGNC:3535]	K01313	F2
ENSP00000368119.3	galactose-1-phosphate uridylyltransferase [Source:HGNC Symbol;Acc:HGNC:4135]	K00965	galT, GALT
ENSP00000378431.1	multimerin 1 [Source:HGNC Symbol;Acc:HGNC:7178]		
ENSP00000326858.3	5'-nucleotidase domain containing 1 [Source:HGNC Symbol;Acc:HGNC:21556]		
ENSP00000414624.2	phosphoribosyl pyrophosphate synthetase associated protein 1 [Source:HGNC Symbol;Acc:HGNC:9466]		
ENSP00000311747.4	RNA binding motif protein 14 [Source:HGNC Symbol;Acc:HGNC:14219]	K13189	RBM14
ENSP00000452245.1	ribonuclease A family member 4 [Source:HGNC Symbol;Acc:HGNC:10047]	K16632	RNASE4
ENSP00000400718.2	B cell receptor associated protein 29 [Source:HGNC Symbol;Acc:HGNC:24131]		
ENSP00000362144.3	mitochondrial calcium uniporter [Source:HGNC Symbol;Acc:HGNC:23526]	K20858	MCU
ENSP00000424791.1	COP9 signalosome subunit 4 [Source:HGNC Symbol;Acc:HGNC:16702]		
ENSP00000301587.4	ATP synthase peripheral stalk subunit d [Source:HGNC Symbol;Acc:HGNC:845]	K02138	ATPeF0D, ATP5H, ATP[illegible]
ENSP00000383059.3	arginine and glutamate rich 1 [Source:HGNC Symbol;Acc:HGNC:25482]	K13173	ARGLU1
ENSP00000385858.1	bromodomain containing 1 [Source:HGNC Symbol;Acc:HGNC:1102]	K11349	BRD1, BRPF2
ENSP00000246069.6	destrin, actin depolymerizing factor [Source:HGNC Symbol;Acc:HGNC:15750]	K10363	DSTN
ENSP00000438455.1	translocase of inner mitochondrial membrane 8 homolog B [Source:HGNC Symbol;Acc:HGNC:[illegible]	K17780	TIM8
ENSP00000406903.1	prothymosin alpha [Source:HGNC Symbol;Acc:HGNC:9623]		
ENSP00000481593.1	hydroxyacyl-thioester dehydratase type 2 [Source:HGNC Symbol;Acc:HGNC:53111]		
ENSP00000307940.5	eukaryotic translation elongation factor 2 [Source:HGNC Symbol;Acc:HGNC:3214]	K03234	EEF2
ENSP00000310551.4	lysocardiolipin acyltransferase 1 [Source:HGNC Symbol;Acc:HGNC:26756]	K13513	LCLAT1, AGPAT8
ENSP00000359665.3	phosphatidylinositol 4-kinase type 2 alpha [Source:HGNC Symbol;Acc:HGNC:30031]	K13711	PI4K2
ENSP00000360569.3	sterol carrier protein 2 [Source:HGNC Symbol;Acc:HGNC:10606]	K08764	SCP2, SCPX
ENSP00000304592.2	fatty acid synthase [Source:HGNC Symbol;Acc:HGNC:3594]	K00665	FASN
ENSP00000303145.4	transmembrane p24 trafficking protein 10 [Source:HGNC Symbol;Acc:HGNC:16998]	K20352	TMED10, ERV25
ENSP00000263867.4	capping actin protein, gelsolin like [Source:HGNC Symbol;Acc:HGNC:1474]	K10368	CAPG
ENSP00000356280.3	pleckstrin homology like domain family A member 3 [Source:HGNC Symbol;Acc:HGNC:8934]		
ENSP00000463424.1	methylthioadenosine phosphorylase [Source:HGNC Symbol;Acc:HGNC:7413]		
ENSP00000247970.4	peptidylprolyl cis/trans isomerase, NIMA-interacting 1 [Source:HGNC Symbol;Acc:HGNC:8988]	K09578	PIN1
ENSP00000242784.3	telomerase RNA component interacting RNase [Source:HGNC Symbol;Acc:HGNC:28424]		
ENSP00000476176.2	solute carrier family 37 member 4 [Source:HGNC Symbol;Acc:HGNC:4061]		
ENSP00000378322.4	protein phosphatase 3 catalytic subunit alpha [Source:HGNC Symbol;Acc:HGNC:9314]	K04348	PPP3C, CNA
ENSP00000287474.4	ferric chelate reductase 1 [Source:HGNC Symbol;Acc:HGNC:27622]		
ENSP00000417864.2	acidic nuclear phosphoprotein 32 family member A [Source:HGNC Symbol;Acc:HGNC:13233]	K18646	ANP32A_C_D
ENSP00000215882.5	solute carrier family 25 member 1 [Source:HGNC Symbol;Acc:HGNC:10979]	K15100	SLC25A1, CTP
ENSP00000349748.5	splicing factor proline and glutamine rich [Source:HGNC Symbol;Acc:HGNC:10774]	K13219	SFPQ, PSF
ENSP00000273395.4	BOC cell adhesion associated, oncogene regulated [Source:HGNC Symbol;Acc:HGNC:17173]	K20020	BOC
ENSP00000337452.4	NIPA magnesium transporter 1 [Source:HGNC Symbol;Acc:HGNC:17043]	K19364	NIPA1
ENSP00000385657.1	protein phosphatase 1 regulatory subunit 7 [Source:HGNC Symbol;Acc:HGNC:9295]	K17550	PPP1R7, SDS22
ENSP00000321498.9	solute carrier family 37 member 3 [Source:HGNC Symbol;Acc:HGNC:20651]	K03447	SLC37A3

ENSP00000296721.4	actin filament associated protein 1 like 1 [Source:HGNC Symbol;Acc:HGNC:26714]		
ENSP00000355325.6	proteasome subunit beta 5 [Source:HGNC Symbol;Acc:HGNC:9542]	K02737	PSMB5
ENSP00000296135.6	leucine zipper transcription factor like 1 [Source:HGNC Symbol;Acc:HGNC:6741]	K19400	LZTFL1
ENSP00000438963.2	phosphatidylinositol glycan anchor biosynthesis class B [Source:HGNC Symbol;Acc:HGNC:895	K05286	PIGB
ENSP00000457733.1	solute carrier family 25 member 24 [Source:HGNC Symbol;Acc:HGNC:20662]	K14684	SLC25A23S
ENSP00000301522.2	peroxiredoxin 2 [Source:HGNC Symbol;Acc:HGNC:9353]	K03386	PRDX2_4, ahpC
ENSP00000390279.2	ribosomal protein L38 [Source:HGNC Symbol;Acc:HGNC:10349]	K02923	RP-L38e, RPL38
ENSP00000354774.3	SPRY domain containing 7 [Source:HGNC Symbol;Acc:HGNC:14297]		
ENSP00000365725.3	abhydrolase domain containing 12 [Source:HGNC Symbol;Acc:HGNC:15868]	K13704	ABHD12
ENSP00000341170.2	pleiotrophin [Source:HGNC Symbol;Acc:HGNC:9630]	K16642	PTN
ENSP00000414921.1	polypyrimidine tract binding protein 3 [Source:HGNC Symbol;Acc:HGNC:10253]	K17844	PTBP3, ROD1
ENSP00000499082.1	crystallin alpha B [Source:HGNC Symbol;Acc:HGNC:2389]	K09542	CRYAB
ENSP00000394071.2	GDP dissociation inhibitor 1 [Source:HGNC Symbol;Acc:HGNC:4226]	K17255	GDI1_2
ENSP00000325146.8	collagen type XII alpha 1 chain [Source:HGNC Symbol;Acc:HGNC:2188]	K08132	COL12A
ENSP00000349696.4	choline/ethanolamine phosphotransferase 1 [Source:HGNC Symbol;Acc:HGNC:24289]	K13644	CEPT1
ENSP00000258739.4	KDEL endoplasmic reticulum protein retention receptor 2 [Source:HGNC Symbol;Acc:HGNC:63	K10949	KDELR
ENSP00000246117.3	nicalin [Source:HGNC Symbol;Acc:HGNC:26923]		
ENSP00000452123.1	serine and arginine rich splicing factor 5 [Source:HGNC Symbol;Acc:HGNC:10787]	K12893	SFRS4_5_6
ENSP00000396899.2	inner membrane mitochondrial protein [Source:HGNC Symbol;Acc:HGNC:6047]	K17785	IMMT, FCJ1, MNOS2
ENSP00000362308.5	SH3 domain binding glutamate rich protein like [Source:HGNC Symbol;Acc:HGNC:10823]		
ENSP00000242209.4	FKBP prolyl isomerase 9 [Source:HGNC Symbol;Acc:HGNC:3725]	K09575	FKBP9_10
ENSP00000359848.3	phosphatidylinositol glycan anchor biosynthesis class K [Source:HGNC Symbol;Acc:HGNC:896	K05290	PIGK
ENSP00000491074.1	phosphatidylinositol glycan anchor biosynthesis class T [Source:HGNC Symbol;Acc:HGNC:14938]		
ENSP00000217971.7	progesterone receptor membrane component 1 [Source:HGNC Symbol;Acc:HGNC:16090]	K17278	PGRMC1_2
ENSP00000379042.3	endoplasmic reticulum oxidoreductase 1 alpha [Source:HGNC Symbol;Acc:HGNC:13280]	K10950	ERO1L
ENSP00000417580.1	isoamyl acetate hydrolyzing esterase 1 (putative) [Source:HGNC Symbol;Acc:HGNC:27696]		
ENSP00000261893.4	lactamase beta [Source:HGNC Symbol;Acc:HGNC:16468]	K17382	LACTB
ENSP00000492745.1	novel protein		
ENSP00000261735.3	endoplasmic reticulum protein 29 [Source:HGNC Symbol;Acc:HGNC:13799]	K09586	ERP29
ENSP00000344868.7	septin 7 [Source:HGNC Symbol;Acc:HGNC:1717]	K16944	SEPT7, CDC3
ENSP00000454612.1	cAMP regulated phosphoprotein 19 [Source:HGNC Symbol;Acc:HGNC:16967]		
ENSP00000352167.3	solute carrier family 25 member 29 [Source:HGNC Symbol;Acc:HGNC:20116]	K15109	SLC25A20_29, CACT, C
ENSP00000358840.1	ATP synthase membrane subunit DAPIT [Source:HGNC Symbol;Acc:HGNC:30889]	K18194	USMG5, DAPIT
ENSP00000274031.3	SET domain containing 7, histone lysine methyltransferase [Source:HGNC Symbol;Acc:HGNC:3	K11431	SETD7
ENSP00000215574.2	cell division cycle 34 [Source:HGNC Symbol;Acc:HGNC:1734]	K02207	UBE2R, UBC3, CDC34
ENSP00000242607.8	hydrogen voltage gated channel 1 [Source:HGNC Symbol;Acc:HGNC:28240]		
ENSP00000360365.3	MCTS1 re-initiation and release factor [Source:HGNC Symbol;Acc:HGNC:23357]	K07575	K07575
ENSP00000168216.6	hydroxysteroid 17-beta dehydrogenase 10 [Source:HGNC Symbol;Acc:HGNC:4800]	K08683	HSD17B10

ENSP00000410833.2	1,4-alpha-glucan branching enzyme 1 [Source:HGNC Symbol;Acc:HGNC:4180]	K00700	GBE1, glgB
ENSP00000418593.1	sorting nexin family member 21 [Source:HGNC Symbol;Acc:HGNC:16154]	K17932	SNX21
ENSP00000376335.2	G protein-coupled receptor 155 [Source:HGNC Symbol;Acc:HGNC:22951]		
ENSP00000353826.2	lon peptidase 1, mitochondrial [Source:HGNC Symbol;Acc:HGNC:9479]	K08675	PRSS15, PIM1
ENSP00000343479.5	NBR1 autophagy cargo receptor [Source:HGNC Symbol;Acc:HGNC:6746]	K17987	NBR1
ENSP00000435096.1	ribosomal protein S25 [Source:HGNC Symbol;Acc:HGNC:10413]	K02975	RP-S25e, RPS25
ENSP00000424603.1	chloride voltage-gated channel 3 [Source:HGNC Symbol;Acc:HGNC:2021]	K05012	CLCN3_4_5
ENSP00000296658.3	carboxymethylenebutenolidase homolog [Source:HGNC Symbol;Acc:HGNC:25090]	K01061	E3.1.1.45
ENSP00000288050.4	pyruvate dehydrogenase phosphatase regulatory subunit [Source:HGNC Symbol;Acc:HGNC:30	K17509	PDPR
ENSP00000382314.3	prefoldin subunit 6 [Source:HGNC Symbol;Acc:HGNC:4926]	K04798	pfdB, PFDN6
ENSP00000301396.5	proline, glutamate and leucine rich protein 1 [Source:HGNC Symbol;Acc:HGNC:30134]	K16913	PELP1, MNAR
ENSP00000285071.4	folliculin [Source:HGNC Symbol;Acc:HGNC:27310]	K09594	FLCN, BHD
ENSP00000362014.3	dynamin 1 [Source:HGNC Symbol;Acc:HGNC:2972]	K01528	DNM
ENSP00000367570.3	PHD finger protein 11 [Source:HGNC Symbol;Acc:HGNC:17024]		
ENSP00000243167.8	fatty acid amide hydrolase [Source:HGNC Symbol;Acc:HGNC:3553]	K15528	FAAH
ENSP00000281830.4	potassium voltage-gated channel subfamily E regulatory subunit 4 [Source:HGNC Symbol;Acc:	K04898	KCNE4
ENSP00000394670.1	peptidylprolyl isomerase A [Source:HGNC Symbol;Acc:HGNC:9253]	K03767	PPIA
ENSP00000216194.8	adenylosuccinate lyase [Source:HGNC Symbol;Acc:HGNC:291]		
ENSP00000359380.2	stearoyl-CoA desaturase [Source:HGNC Symbol;Acc:HGNC:10571]	K00507	SCD, desC
ENSP00000354255.4	carboxypeptidase Z [Source:HGNC Symbol;Acc:HGNC:2333]	K13022	CPZ
ENSP00000361152.5	peroxiredoxin 1 [Source:HGNC Symbol;Acc:HGNC:9352]	K13279	PRDX1
ENSP00000288607.2	proteasome assembly chaperone 3 [Source:HGNC Symbol;Acc:HGNC:22420]	K11877	PSMG3, PAC3
ENSP00000363500.4	chloride intracellular channel 4 [Source:HGNC Symbol;Acc:HGNC:13518]	K05024	CLIC4
ENSP00000216122.3	minichromosome maintenance complex component 5 [Source:HGNC Symbol;Acc:HGNC:6948]	K02209	MCM5, CDC46
ENSP00000228136.4	chromosome 11 open reading frame 58 [Source:HGNC Symbol;Acc:HGNC:16990]		
ENSP00000402346.2	coatomer protein complex subunit gamma 2 [Source:HGNC Symbol;Acc:HGNC:2237]	K17267	COPG
ENSP00000253382.5	acyl-CoA synthetase short chain family member 2 [Source:HGNC Symbol;Acc:HGNC:15814]	K01895	ACSS, acs
ENSP00000305255.2	syntaxin 8 [Source:HGNC Symbol;Acc:HGNC:11443]	K08501	STX8
ENSP00000216446.4	pleckstrin 2 [Source:HGNC Symbol;Acc:HGNC:19238]	K19993	PLEK
ENSP00000367123.2	solute carrier family 3 member 2 [Source:HGNC Symbol;Acc:HGNC:11026]		
ENSP00000217121.5	TPD52 like 2 [Source:HGNC Symbol;Acc:HGNC:12007]		
ENSP00000262193.6	proteasome subunit beta 1 [Source:HGNC Symbol;Acc:HGNC:9537]	K02732	PSMB1
ENSP00000349053.3	glucosidase II alpha subunit [Source:HGNC Symbol;Acc:HGNC:4138]	K05546	GANAB
ENSP00000365505.5	proteolipid protein 2 [Source:HGNC Symbol;Acc:HGNC:9087]		
ENSP00000258415.4	cytochrome P450 family 27 subfamily A member 1 [Source:HGNC Symbol;Acc:HGNC:2605]	K00488	CYP27A
ENSP00000483613.1	sorting nexin 12 [Source:HGNC Symbol;Acc:HGNC:14976]		
ENSP00000268182.5	IQ motif containing GTPase activating protein 1 [Source:HGNC Symbol;Acc:HGNC:6110]	K16848	IQGAP1
ENSP00000263370.1	inositol-trisphosphate 3-kinase C [Source:HGNC Symbol;Acc:HGNC:14897]	K00911	ITPK

ENSP00000362329.2	pyrophosphatase (inorganic) 1 [Source:HGNC Symbol;Acc:HGNC:9226]	K01507	ppa
ENSP00000313318.3	solute carrier family 35 member C1 [Source:HGNC Symbol;Acc:HGNC:20197]	K15279	SLC35C1, FUCT1
ENSP00000360316.3	24-dehydrocholesterol reductase [Source:HGNC Symbol;Acc:HGNC:2859]	K09828	DHCR24, DWF1
ENSP00000420065.1	abhydrolase domain containing 14B [Source:HGNC Symbol;Acc:HGNC:28235]	K13706	ABHD14
ENSP00000384739.2	7-dehydrocholesterol reductase [Source:HGNC Symbol;Acc:HGNC:2860]	K00213	DHCR7
ENSP00000318845.4	synaptogyrin 1 [Source:HGNC Symbol;Acc:HGNC:11498]		
ENSP00000312615.2	transmembrane protein 134 [Source:HGNC Symbol;Acc:HGNC:26142]		
ENSP00000371267.4	tyrosine 3-monooxygenase/tryptophan 5-monooxygenase activation protein theta [Source:H(	K16197	YWHAB_Q_Z
ENSP00000451084.1	fermitin family member 2 [Source:HGNC Symbol;Acc:HGNC:15767]	K17083	FERMT2, KIND2
ENSP00000409879.2	transmembrane protein 159 [Source:HGNC Symbol;Acc:HGNC:30136]		
ENSP00000270142.6	superoxide dismutase 1 [Source:HGNC Symbol;Acc:HGNC:11179]	K04565	SOD1
ENSP00000261208.3	histidine ammonia-lyase [Source:HGNC Symbol;Acc:HGNC:4806]	K01745	hutH, HAL
ENSP00000266556.7	TAP binding protein like [Source:HGNC Symbol;Acc:HGNC:30683]		
ENSP00000247470.9	PYD and CARD domain containing [Source:HGNC Symbol;Acc:HGNC:16608]	K12799	PYCARD, ASC
ENSP00000381216.2	KH-type splicing regulatory protein [Source:HGNC Symbol;Acc:HGNC:6316]	K13210	FUBP
ENSP00000378405.1	branched chain keto acid dehydrogenase kinase [Source:HGNC Symbol;Acc:HGNC:16902]	K00905	BCKDK
ENSP00000252487.4	translocase of outer mitochondrial membrane 40 [Source:HGNC Symbol;Acc:HGNC:18001]	K11518	TOM40
ENSP00000370258.4	eukaryotic translation initiation factor 3 subunit C like [Source:HGNC Symbol;Acc:HGNC:26347	K03252	EIF3C
ENSP00000339720.5	NDUFA4 mitochondrial complex associated [Source:HGNC Symbol;Acc:HGNC:7687]	K03948	NDUFA4
ENSP00000356856.5	transmembrane and coiled-coil domains 1 [Source:HGNC Symbol;Acc:HGNC:18188]		
ENSP00000203407.5	ubiquinol-cytochrome c reductase core protein 1 [Source:HGNC Symbol;Acc:HGNC:12585]	K00414	QCR1, UQCRC1
ENSP00000471700.1	FKBP prolyl isomerase 8 [Source:HGNC Symbol;Acc:HGNC:3724]	K09574	FKBP8
ENSP00000224807.5	sideroflexin 3 [Source:HGNC Symbol;Acc:HGNC:16087]		
ENSP00000296582.3	transmembrane protein 184C [Source:HGNC Symbol;Acc:HGNC:25587]		
ENSP00000205402.3	dihydrolipoamide dehydrogenase [Source:HGNC Symbol;Acc:HGNC:2898]	K00382	DLD, lpd, pdhD
ENSP00000009589.3	ribosomal protein S20 [Source:HGNC Symbol;Acc:HGNC:10405]	K02969	RP-S20e, RPS20
ENSP00000332455.3	karyopherin subunit alpha 2 [Source:HGNC Symbol;Acc:HGNC:6395]	K15043	KPNA2
ENSP00000381377.3	ARPIN-AP3S2 readthrough [Source:HGNC Symbol;Acc:HGNC:38824]	K12399	AP3S
ENSP00000389903.2	LETM1 domain containing 1 [Source:HGNC Symbol;Acc:HGNC:24241]		
ENSP00000359991.4	phosphoglycerate mutase 1 [Source:HGNC Symbol;Acc:HGNC:8888]	K01834	PGAM, gpmA
ENSP00000436318.1	copper chaperone for superoxide dismutase [Source:HGNC Symbol;Acc:HGNC:1613]	K04569	CCS
ENSP00000297632.6	transmembrane protein 65 [Source:HGNC Symbol;Acc:HGNC:25203]		
ENSP00000408526.2	inositol monophosphatase 1 [Source:HGNC Symbol;Acc:HGNC:6050]	K01092	E3.1.3.25, IMPA, suhB
ENSP00000251363.5	ceramide synthase 4 [Source:HGNC Symbol;Acc:HGNC:23747]	K04710	CERS
ENSP00000228938.5	matrix Gla protein [Source:HGNC Symbol;Acc:HGNC:7060]		
ENSP00000294244.3	spindlin interactor and repressor of chromatin binding [Source:HGNC Symbol;Acc:HGNC:25115]		
ENSP00000377862.3	FAT atypical cadherin 4 [Source:HGNC Symbol;Acc:HGNC:23109]	K16669	FAT4
ENSP00000272065.5	acid phosphatase 1 [Source:HGNC Symbol;Acc:HGNC:122]	K14394	ACP1

ENSP00000386156.2	PYM homolog 1, exon junction complex associated factor [Source:HGNC Symbol;Acc:HGNC:3(	K14294	WIBG, PYM
ENSP00000368876.3	TOG array regulator of axonemal microtubules 2 [Source:HGNC Symbol;Acc:HGNC:33715]		
ENSP00000385714.1	novel protein, AP000351.4-DDT readtrhough	K10028	DDT
ENSP00000261811.4	cysteine rich transmembrane module containing 1 [Source:HGNC Symbol;Acc:HGNC:30239]		
ENSP00000441365.1	MARVEL domain containing 1 [Source:HGNC Symbol;Acc:HGNC:28674]		
ENSP00000459533.1	lipopolysaccharide induced TNF factor [Source:HGNC Symbol;Acc:HGNC:16841]	K19363	LITAF
ENSP00000333920.2	transcription termination factor 1 [Source:HGNC Symbol;Acc:HGNC:12397]	K15225	TTF1
ENSP00000363189.4	transmembrane protein 222 [Source:HGNC Symbol;Acc:HGNC:25363]	K20726	TMEM222
ENSP00000465517.1	chromosome 18 open reading frame 21 [Source:HGNC Symbol;Acc:HGNC:28802]		
ENSP00000261183.3	oxysterol binding protein like 8 [Source:HGNC Symbol;Acc:HGNC:16396]	K20464	OSBPL5_8, ORP5_8
ENSP00000229563.5	transmembrane protein 14C [Source:HGNC Symbol;Acc:HGNC:20952]		
ENSP00000370543.3	solute carrier family 5 member 3 [Source:HGNC Symbol;Acc:HGNC:11038]	K14383	SLC5A3, SMIT
ENSP00000230124.3	FIG4 phosphoinositide 5-phosphatase [Source:HGNC Symbol;Acc:HGNC:16873]		
ENSP00000498932.1	chromosome 11 open reading frame 96 [Source:HGNC Symbol;Acc:HGNC:38675]		
ENSP00000367541.1	tropomyosin 2 [Source:HGNC Symbol;Acc:HGNC:12011]		
ENSP00000447297.1	transforming growth factor beta receptor 1 [Source:HGNC Symbol;Acc:HGNC:11772]	K04674	TGFBR1, ALK5
ENSP00000264027.4	sterol-C5-desaturase [Source:HGNC Symbol;Acc:HGNC:10547]	K00227	SC5DL, ERG3
ENSP00000374484.4	transmembrane protein 87A [Source:HGNC Symbol;Acc:HGNC:24522]		
ENSP00000355361.5	CD47 molecule [Source:HGNC Symbol;Acc:HGNC:1682]	K06266	CD47
ENSP00000359910.4	proteasome subunit alpha 7 [Source:HGNC Symbol;Acc:HGNC:9536]	K02731	PSMA7
ENSP00000342026.5	peroxiredoxin 6 [Source:HGNC Symbol;Acc:HGNC:16753]	K11188	PRDX6
ENSP00000311677.5	protein phosphatase 1 regulatory subunit 8 [Source:HGNC Symbol;Acc:HGNC:9296]	K13216	PPP1R8, NIPP1
ENSP00000009041.7	STARD3 N-terminal like [Source:HGNC Symbol;Acc:HGNC:19169]		
ENSP00000216038.5	RNA 2',3'-cyclic phosphate and 5'-OH ligase [Source:HGNC Symbol;Acc:HGNC:26935]	K14415	RTCB, rtcB
ENSP00000336994.4	CLPTM1 regulator of GABA type A receptor forward trafficking [Source:HGNC Symbol;Acc:HGNC:2087]		
ENSP00000343054.3	RNA binding motif protein 5 [Source:HGNC Symbol;Acc:HGNC:9902]	K13094	RBM5_10
ENSP00000170564.1	G-patch domain containing 1 [Source:HGNC Symbol;Acc:HGNC:24658]	K13123	GPATCH1
ENSP00000466613.1	major facilitator superfamily domain containing 11 [Source:HGNC Symbol;Acc:HGNC:25458]		
ENSP00000397818.2	solute carrier family 25 member 19 [Source:HGNC Symbol;Acc:HGNC:14409]	K15108	SLC25A19, DNC, TPC1
ENSP00000360034.2	SERPINE1 mRNA binding protein 1 [Source:HGNC Symbol;Acc:HGNC:17860]	K13199	SERBP1
ENSP00000401053.1	ubiquitin conjugating enzyme E2 E2 [Source:HGNC Symbol;Acc:HGNC:12478]	K20217	UBE2E
ENSP00000419038.1	G elongation factor mitochondrial 1 [Source:HGNC Symbol;Acc:HGNC:13780]	K02355	fusA, GFM, EFG
ENSP00000456019.1	sulfide quinone oxidoreductase [Source:HGNC Symbol;Acc:HGNC:20390]	K17218	sqr
ENSP00000357076.1	transgelin 2 [Source:HGNC Symbol;Acc:HGNC:11554]	K20526	TAGLN
ENSP00000419970.1	cytochrome p450 oxidoreductase [Source:HGNC Symbol;Acc:HGNC:9208]	K00327	POR
ENSP00000381844.3	atlastin GTPase 3 [Source:HGNC Symbol;Acc:HGNC:24526]	K17339	ATL
ENSP00000377372.3	growth associated protein 43 [Source:HGNC Symbol;Acc:HGNC:4140]	K20041	GAP43
ENSP00000401260.1	Bet1 golgi vesicular membrane trafficking protein [Source:HGNC Symbol;Acc:HGNC:14562]	K08504	BET1

ENSP00000385080.1	fumarylacetoacetate hydrolase [Source:HGNC Symbol;Acc:HGNC:3579]	K01555	FAH, fahA
ENSP00000300161.4	tyrosine 3-monooxygenase/tryptophan 5-monooxygenase activation protein beta [Source:HG	K16197	YWHAB_Q_Z
ENSP00000211998.4	vinculin [Source:HGNC Symbol;Acc:HGNC:12665]	K05700	VCL
ENSP00000261396.3	nucleoporin 133 [Source:HGNC Symbol;Acc:HGNC:18016]	K14300	NUP133
ENSP00000314608.3	kelch like family member 11 [Source:HGNC Symbol;Acc:HGNC:19008]	K10449	KLHL11
ENSP00000295317.3	ring finger protein 149 [Source:HGNC Symbol;Acc:HGNC:23137]	K15704	RNF149
ENSP00000357357.3	glucosylceramidase beta [Source:HGNC Symbol;Acc:HGNC:4177]	K01201	GBA, srfJ
ENSP00000272928.3	atypical chemokine receptor 3 [Source:HGNC Symbol;Acc:HGNC:23692]	K04304	CXCR7, RDC1
ENSP00000455313.1	secretory carrier membrane protein 5 [Source:HGNC Symbol;Acc:HGNC:30386]		
ENSP00000265896.5	squalene epoxidase [Source:HGNC Symbol;Acc:HGNC:11279]	K00511	SQLE, ERG1
ENSP00000320081.3	divergent protein kinase domain 2A [Source:HGNC Symbol;Acc:HGNC:28490]		
ENSP00000256689.5	solute carrier family 38 member 2 [Source:HGNC Symbol;Acc:HGNC:13448]	K14207	SLC38A2, SNAT2
ENSP00000431482.1	NEDD8-MDP1 readthrough [Source:HGNC Symbol;Acc:HGNC:39551]	K17619	MDP1
ENSP00000259457.3	proteasome subunit beta 7 [Source:HGNC Symbol;Acc:HGNC:9544]	K02739	PSMB7
ENSP00000355599.3	translin associated factor X [Source:HGNC Symbol;Acc:HGNC:12380]		
ENSP00000319739.5	reticulocalbin 2 [Source:HGNC Symbol;Acc:HGNC:9935]		
ENSP00000422872.1	ATPase secretory pathway Ca2+ transporting 1 [Source:HGNC Symbol;Acc:HGNC:13211]	K01537	E3.6.3.8
ENSP00000474249.1	NEDD8-MDP1 readthrough [Source:HGNC Symbol;Acc:HGNC:39551]	K12158	NEDD8
ENSP00000292401.4	alpha-2-glycoprotein 1, zinc-binding [Source:HGNC Symbol;Acc:HGNC:910]		
ENSP00000356218.4	cytochrome b5 reductase 1 [Source:HGNC Symbol;Acc:HGNC:13397]	K00326	E1.6.2.2
ENSP00000290349.6	carbonyl reductase 1 [Source:HGNC Symbol;Acc:HGNC:1548]	K00079	CBR1
ENSP00000432782.1	dipeptidyl peptidase 3 [Source:HGNC Symbol;Acc:HGNC:3008]	K01277	DPP3
ENSP00000263388.1	notch receptor 3 [Source:HGNC Symbol;Acc:HGNC:7883]	K20995	NOTCH3
ENSP00000265753.8	eukaryotic translation initiation factor 4H [Source:HGNC Symbol;Acc:HGNC:12741]		
ENSP00000416683.3	insulin like growth factor binding protein acid labile subunit [Source:HGNC Symbol;Acc:HGNC:	K17256	IGFALS, ALS
ENSP00000254759.3	coenzyme Q3, methyltransferase [Source:HGNC Symbol;Acc:HGNC:18175]	K00591	COQ3
ENSP00000433138.1	caspase 1 [Source:HGNC Symbol;Acc:HGNC:1499]	K01370	CASP1
